# Supplementary material for: Genome-Based Analysis of Verticillium Polyketide Synthase Gene Clusters
Source: Biology (Basel). 2022 Aug 23;11(9):1252. doi: 10.3390/biology11091252 (PMC9495618; doi:10.3390/biology11091252)
Supplement: Supplementary file 1 [file biology-11-01252-s001.zip › biology-1827821-supplementary.pdf]

Supplementary Figure S1. Neighbor-joining tree inferred from nucleotide sequences of Polyketide Synthase-III coding regions of Verticillium species as well as PKS-III sequences obtained from other Ascomycetes. GenBank accession numbers for each sequence are provided. Similar groups were obtained with the maximum likelihood analysis. In this analysis we used 1000 bootstrap repeats as indicated at the internodes

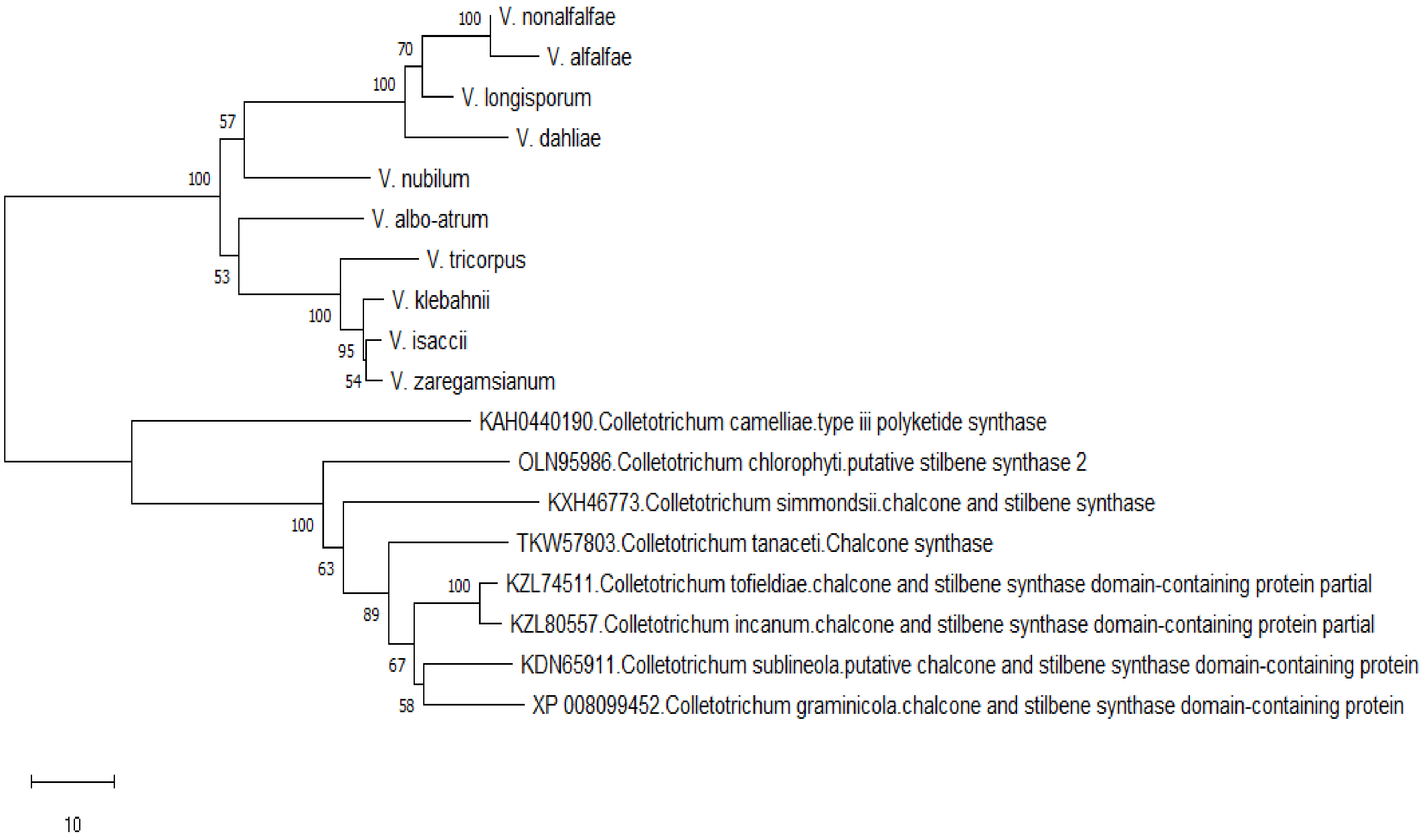

Supplementary Figure S2. Comparison of putative cluster genes for Verticillium VRPKS-I-21 found in *V. zaregamsianum*, *V. klebahnii* and *V. nubilum* and the alternapyrone biosynthesis gene cluster of *Alternaria alternata*. The arrowheads indicate direction of transcription and types of genes common between the two clusters being compared are displayed in the same color. Shaded lines represent similarities between nucleotide sequences.

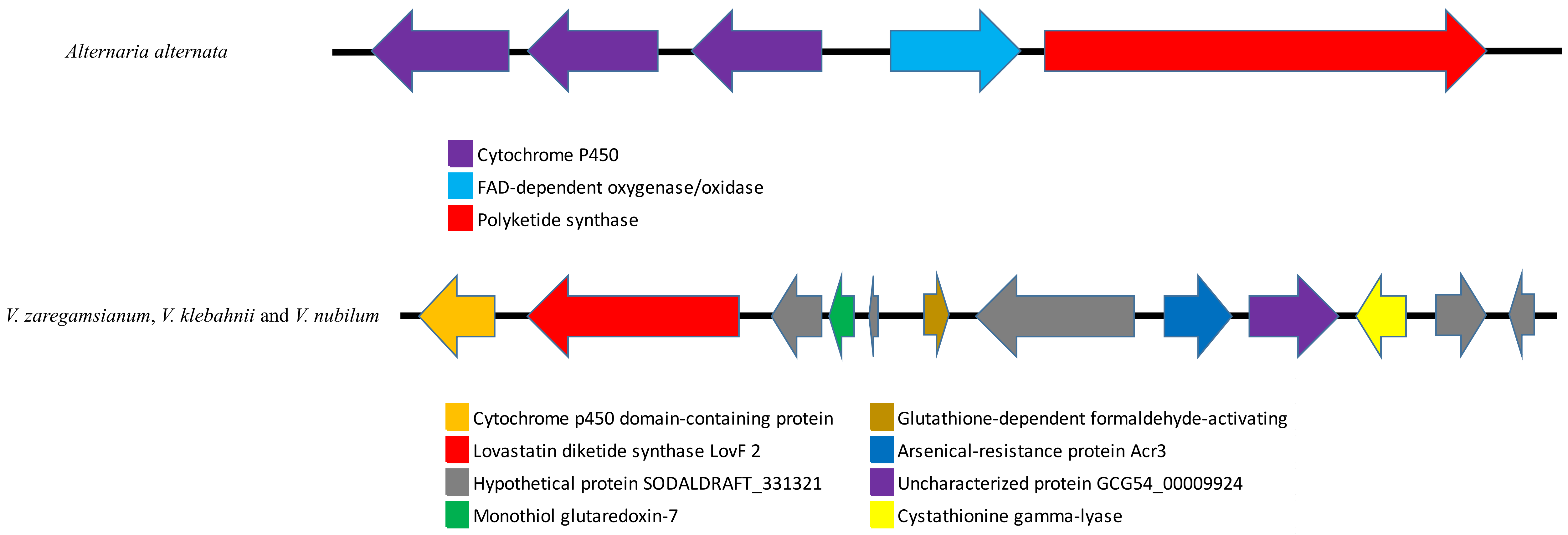

Supplementary Figure S3. Comparison of putative cluster genes for VRPKS-I-22 locus found in the genomes of *V. dahliae*, *V. longisporum*, *V. alfalfa* and *V. nonalfalfae* and fusaric acid gene cluster. The arrowheads indicate direction of transcription and types of genes common between the two clusters being compared are displayed in the same color. Shaded lines represent similarities between nucleotide sequences.

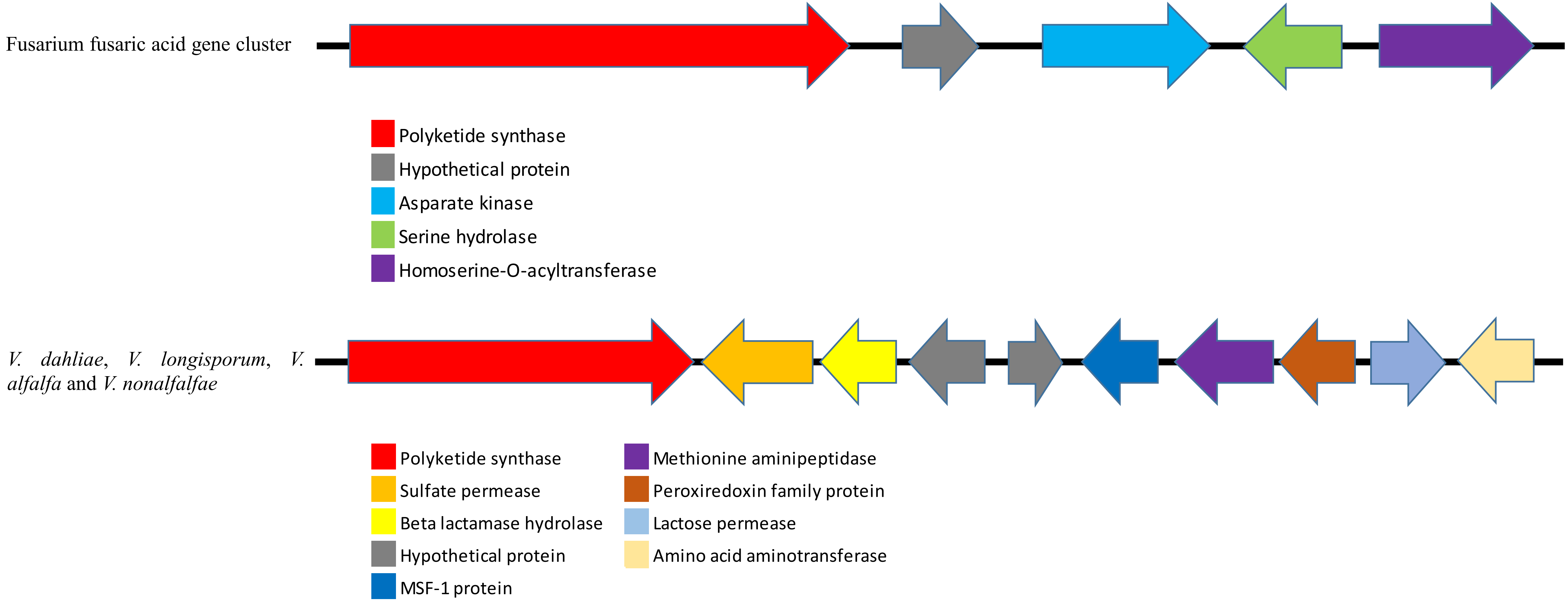

SUPPLEMENTARY FILE S1. The tables below show the predicted Polyketide synthase (PKS) gene clusters in all examined Verticillium genomes.

1) *Verticillium dahliae* contig NW_009276940, VRPKS-I-22 cluster

Gene name	Size	E value	% Coverage	% identity	Top blast hit	Accession number of top blast hit
RNA polymerase associated protein	610	0	100	60.39	*Colletotrichum chlorophyti*	OLN85623
Uniquinone biosynthesis protein	244	0	100	76.42	*Colletotrichum tofieldiae*	KZL76982
Phospholipase D1	1819	0	100	72.86	*Sodiomyces alkalinus*	XP_028463305
Tubulin gamma chain	461	0	100	94.14	*Fusarium oxysporum*	EGU82165
Gamma tubulin	93	4e-37	94	73.86	*Pseudomassariella vexata*	XP_040719305
Hypothetical protein	851	2e-10	14	37.88	*Sodiomyces alkalinus*	XP_028463302
Putative uridine kinase	192	2e-78	100	53.97	*Colletotrichum camelliae*	KAG2083351
Oxidoreductase	345	9e-132	98	56.10	*Fusarium proliferatum*	RBA11051
Major facilitator transporter (MFS)	229	1e-110	99	74.45	*Colletotrichum graminicola*	XP_008093595
FAD dependent monooxygenase	404	1e-177	89	68.06	*Bimuria novae-zelandiae*	KAF1970089
DUF341 domain containing protein	207	1e-98	100	63.76	*Fusarium napiforme*	KAF5540875
UDP glucosyl transferase	553	0	98	59.19	*Fusarium austroafricanum*	KAF4443747
Polyketide synthase	2635	0	99	70.93	*Fusarium miscanthi*	ALQ32877
Sulfate permease	763	0	98	51.05	*Trematosphaeria pertusa*	XP_033684343
Beta lactamase hydrolase	300	1e-137	100	62.33	*Monosporascus sp.*	RYP55738
Hypothetical protein	371	1e-28	87	60	*Fusarium graminearum*	CZS74879
Hypothetical protein	153	2e-80	99	79.22	*Colletotrichum musicola*	KAF6840530
MSF-1 protein	192	2e-121	98	85.94	*Sodiomyces alkalinus*	XP_028463299
Methionine aminipeptidase	441	0	99	82.99	*Colletotrichum sidae*	TEA17142
Peroxiredoxin family protein	425	2e-154	70	67.44	*Sodiomyces alkalinus*	XP_028463297
Lactose permease	306	1e-07	76	26.05	*Cadophora sp.*	KAG4430930
Amino acid aminotransferase	425	0	100	75.81	*Sodiomyces alkalinus*	XP_028463295

2) *Verticillium dahliae* contig NW_009276940, VRPKS-I-23 cluster

Gene name	Size	E value	% Coverage	% identity	Top blast hit	Accession number of top blast hit
Hypothetical protein	351	1e-51	99	34.59	*Sodiomyces alkalinus*	XP_028463289
Homoserine kinase	355	0	99	90.73	*Colletotrichum tofieldiae*	KZL71078
Hypothetical protein	146	1e-96	100	100	*Verticillium dahliae*	XP_009656175
SnodProt1	138	1e-64	100	64.49	*Hirsutella minnesotensis*	KJZ76905
Hypothetical protein	242	2e-5	64	30.65	*Sodiomyces alkalinus*	XP_028462488
Cocaine esterase	600	0	94	60.27	*Sodiomyces alkalinus*	XP_028462489
Hypothetical protein	363	1e-109	100	48.44	*Sodiomyces alkalinus*	XP_028462491
PKS	2402	0	99	49.71	*Sodiomyces alkalinus*	XP_028462492
Drug resistance transporter	485	0	98	53.12	*Stylonectria norvegica*	KAF7555861
Virulence sensor protein/Heat shock protein 90 (Hsp90)	1660	0	83	67.05	*Sodiomyces alkalinus*	XP_028465417
PXMP 2/4 family protein	255	4e-97	100	57.25	*Colletotrichum musicola*	KAF6806760

Transport protein	327	0	99	85.54	*Colletotrichum simmondsii*	KXH42740
Signal recognition receptor	568	0	98	75.79	*Sodiomyces alkalinus*	XP_028465413
Myosin class II	2591	0	82	61.33	*Sodiomyces alkalinus*	XP_028465411

3) *Verticillium dahliae* contig NW_009276935, VRPKS-I-26 cluster

**Gene name**	**Size**	**E value**	**% Coverage**	**% identity**	**Top blast hit**	**Accession number of top blast hit**
Short chain dehydrogenase	292	4e-122	99	58.42	*Colletotrichum siamense*	XP_036493403
Transcriptional regulatory protein	399	8e-79	89	40.22	*Colletotrichum orchidophilum*	XP_022471000
Ankyrin repeat protein	778	1e-61	81	28.59	*Colletotrichum truncatum*	XP_036575058
Putative thiol methyltransferase	77	2e-48	100	100	*Verticillium dahliae*	KAF3349318
Hypothetical protein	1017	0	99	54.45	*Microbacterium sp*	WP_144806847
Hypothetical protein	577	0	100	86.70	*Colletotrichum higginsianum*	XP_018157666
Integral membrane protein	365	7e-177	98	68.89	*Colletotrichum incanum*	KZL85847
Dehydrogenase/oxidoreductase	616	0	93	78.86	*Colletotrichum sublineola*	KDN72067
Hypothetical protein	1269	0	78	70.13	*Colletotrichum fioriniae*	EXF76932
Hypothetical protein	162	6e-59	97	57.86	*Colletotrichum viniferum*	KAF4921290
PKS	1427	0	98	62.71	*Monosporascus sp*	RYP50633
Tubulin tyrosine ligase protein	472	0	99	70.10	*Coniochaeta sp.*	KAB5518934
Short chain dehydrogenase	312	6e-166	98	71.75	*Stachybotrys chlor*	KFA66828
Leucine amidopeptidase	373	0	100	72.65	*Cylindrodendrum hubeiense*	KAF7555029
Midasin	324	1e-24	83	38.74	*Colletotrichum siamense*	KAF4871344
Hypothetical protein	200	1e-90	99	56.36	*Sodiomyces alkalinus*	XP_028470403
Hypothetical protein	126	8e-22	56	69.01	*Hypoxylon sp*	OTA94229
Hypothetical protein	452	2e-93	92	45.92	*Colletotrichum chlorophyti*	OLN97947
LEA domain containing protein	1146	0	97	43	*Colletotrichum orchidophilum*	XP_022467882

4) *Verticillium dahliae* contig NW_009276940, PKS-III cluster

**Gene name**	**Size**	**E value**	**% Coverage**	**% identity**	**Top blast hit**	**Accession number of top blast hit**
Carbonic anhydrase	271	1e-141	98	70.41	*Colletotrichum simmondsii*	KXH49803
Acetyltransferase	279	7e-146	86	79.75	*Colletotrichum higginsianum*	XP_018159255
Oxidoreductase	451	0	98	60.14	*Colletotrichum higginsianum*	XP_018159254
Feruloyl esterase	294	1e-146	96	96.23	*Colletotrichum orbiculare*	TDZ25527
Fungal specific transcription factor	608	0	99	72.88	*Colletotrichum incanum*	KZL80546
BFR2 protein	598	0	100	62.42	*Colletotrichum plurivorum*	KAF6823613
PKS-III	440	0	100	72.01	*Colletotrichum chlorophyti*	OLN95986
Fructose bisphosphate aldolase	199	4e-85	100	93.84	*Sodiomyces alkalinus*	XP_028470318
Specific exonuclease	1531	0	66	73.98	*Sodiomyces alkalinus*	XP_028470311
Mitochondrial 2 oxoglutarate carrier	335	0	100	85.97	*Colletotrichum chlorophyti*	OLN95994
Hypothetical protein	651	3e-168	98	53.53	*Colletotrichum truncatum*	XP_036586171
Splicing factor spf30	320	2e-148	100	72.81	*Colletotrichum truncatum*	XP_036586172
Iron sulfur protein	82	4e-29	95	75.64	*Colletotrichum plurivorum*	KAF6823606
Cell division control protein	191	4e-95	98	70.37	*Sodiomyces alkalinus*	XP_028470317

Short chain dehydrogenase	69	3e-23	76	84.91	*Sodiomyces alkalinus*	XP_028470310
Cellular morphogenesis protein	256	3e-51	97	46.15	*Colletotrichum sojae*	KAF6809931

5) *Verticillium dahliae* contig NW_009276948, VRPKS-I-19 cluster

Gene name	Size	E value	% Coverage	% identity	Top blast hit	Accession number of top blast hit
Extracellular serine rich protein	216	7e-85	86	69.84	*Colletotrichum higginsianum*	XP_018161331
Hypothetical protein	77	1e-21	97	55.13	*Sodiomyces alkalinus*	XP_028465568
Hypothetical protein	290	7e-70	95	44.24	*Acremonium chrysogenum*	KFH42767
NADH ubiquinone oxidoreductase	209	2e-58	84	55.21	*Thermothielavioides terrestris*	SPQ17832
Mitochondrial inner membrane translocase	542	0	100	74.91	*Sodiomyces alkalinu*	XP_028468965
Ribosomal protein	248	2e-98	100	58.47	*Colletotrichum fioriniae*	EXF75199.
DeSI like protein sdu 1	257	2e-136	97	76	*Colletotrichum chlorophyti*	OLN97068
Obg like ATPase	394	0	100	88.83	*Claviceps africana*	KAG5929667
Exopolygalacturonase/Thioredoxin domain	509	1e-180	76	57.01	*Monosporascus sp*	RYP54678
Ammonium transporter/drug resistance protein	576	0	98	69.80	*Sodiomyces alkalinus*	XP_028468956
R-PKS-I	2160	0	99	61.92	*Claviceps purpurea*	KAG6168136
Esterase	274	1e-89	97	47.84	*Claviceps citrina*	KAG6041354
L-theronate dehydrogenase	1134	0	99	72.90	*Colletotrichum graminicola*	XP_008095607
Fungal specific transcription factor	633	0	100	61.80	*Colletotrichum salicis*	KXH47283
MFS transporter	488	0	99	76.18	*Colletotrichum chlorophyti*	OLN87319
Hypothetical protein	205	9e-132	94	96.89	*Verticillium longisporum*	KAG7105862
Alpha/beta hydrolase	196	6e-54	96	44.58	*Zopfia rhizophila*	KAF2180147
Cutinase	224	9e-116	91	80.39	*Sodiomyces alkalinus*	XP_028468955
Vacuolar protein sorting associate protein 9	770	0	98	69.64	*Colletotrichum orbiculare*	TDZ20835
Hypothetical protein	482	3e-34	65	45.02	*Colletotrichum tofieldiae*	KZL68686
Acetylxylan esterase 2 like protein	284	3e-93	89	58.36	*Colletotrichum siamense*	KAF4811263
Ankyrin repeat protein	381	4e-16	24	47.37	*Colletotrichum tofieldiae*	KZL70525

6) *Verticillium dahliae* contig NW_009276923, VRPKS-I-1 cluster

Gene name	Size	E value	% Coverage	% identity	Top blast hit	Accession number of top blast hit
AP-3 complex subunit beta-2 like protein	659	0	92	78.49	*Colletotrichum truncatum*	XP_036580440
Hypothetical protein	135	8e-35	98	46.41	*Sodiomyces alkalinus*	XP_028470755
Kinetochore protein nuf2	331	8e-178	90	79.05	*Sodiomyces alkalinus*	XP_028470756
Hypothetical protein	328	4e-32	77	34.53	*Fusarium graminum*	KAF5000361
Hypothetical protein	195	1e-58	97	52.63	*Fusarium oxysporum f. sp. rapae*	KAG7415270
Efflux pump/MFS-type efflux transporter	585	0	91	68.96	*Torrubiella hemipte*	CEJ81615
Transcription factor (Isoflavipucine cluster transcription factor)	413	2e-56	50	44.05	*Torrubiella hemipterig*	CEJ81614
Hypothetical protein	195	2e-42	96	38.62	*Lasiodiplodia theobromae*	KAB2573329
Enoyl reductase	317	1e-79	96	41.64	*Aspergillus turcosus*	RHZ60657

Hydrolase/aminopeptidase	359	7e-98	96	49.71	*Tothia fuscella*	KAF2431630
Hypothetical protein	371	4e-94	99	42.20	*Phialocephala subalpina*	CZR58350
PKS-NRPS	4123	0	99	44.83	*Massariosphaeria phaeosp*	KAF2874833
Hypothetical protein	286	3e-71	78	50	*Diaporthe helianthi*	POS73388
Hypothetical protein	154	3e-73	97	69.33	*Penicillium brasilianum*	CEJ59049
Oxidoreductase	332	0	100	75	*Stachybotrys chartarum*	KFA54517
Hypothetical protein	717	0	95	52.47	*Colletotrichum simmondsii*	KXH52233

7) *Verticillium dahliae* contig NW_009276921, VNRPKS-I-1 cluster

Gene name	Size	E value	% Coverage	% identity	Top blast hit	Accession number of top blast hit
U3 small nucleolar ribonucleoprotein IMP3	183	7e-117	100	88.52	*Sodiomyces alkalinus*	XP_028469265
37S ribosomal protein	207	1e-95	96	69.85	*Sodiomyces alkalinus*	XP_028469266
Complex I intermediate associate protein	693	0	99	72.05	*Sodiomyces alkalinus*	XP_028469267
Short chain dehydrogenase	284	1e-157	100	79.23	*Colletotrichum fioriniae*	EXF81015
Hypothetical protein	286	6e-53	57	52.73	*Sodiomyces alkalinus*	XP_028469272
L 2,4 diaminobutyrate decarboxylase	479	0	96	58.67	*Colletotrichum sidae*	TEA12782
DUF 92 domain containing protein	390	4e-168	99	64.86	*Sodiomyces alkalinus*	XP_028469270
Hypothetical protein	223	4e-77	49	100	*Verticillium longisporum*	KAG7142964
Multicopper oxidase	586	0	91	65.99	*Colletotrichum tofieldiae*	KZL72261
NR-PKS-I	2189	0	100	76.45	*Colletotrichum karsti*	XP_038751444
Oxidoreductase/Hypothetical protein	220	1e-117	91	72.73	*Colletotrichum truncatum*	XP_036586400
Hypothetical protein	246	1e-53	51	70.63	*Colletotrichum higginsianum*	CCF47183
Transcription factor	578	6e-176	97	70.54	*Colletotrichum truncatum*	XP_036589502
Glycosyl transferase	187	2e-82	100	61.65	*Sodiomyces alkalinus*	XP_028469283
Dihydrodipicolinate synthetase family	331	9e-178	97	73.68	*Daldinia sp.*	OTB10674
Autophagy related protein 2	1786	0	98	67.90	*Sodiomyces alkalinus*	XP_028469284

8) *Verticillium dahliae* contig NW_009276930, VRPKS-I-8 cluster

Gene name	Size	E value	% Coverage	% identity	Top blast hit	Accession number of top blast hit
PKS	2574	0	95	77.88	*Stemphylium lycopersici*	RAR12984
Enoyl reductase	330	2e-164	99	64.15	*Fusarium mexicanum*	RAR12984
Hydrolase	288	0	100	85.12	*Stemphylium lycopersici*	KNG48003
Fungal ZN(2) Cys(6) cluster domain containing protein/Fujikurins biosynthesis transcription factor	366	1e-126	99	57.53	*Stemphylium lycopersici*	KNG48004
MFS gliotoxin efflux transporter	470	0	99	69.57	*Ascochyta lentis*	KAF9701447
Cytochrome monooxygenase	513	0	93	73.65	*Fusarium sp.*	KAF5026356
Hypothetical protein	237	8e-47	62	54.38	*Cadophora malorum*	KAG4414469
Hypothetical protein	93	2e-24	98	54.37	*Stylonectria norvegica*	KAF7562826
Hypothetical protein	244	1e-38	100	39.38	*Stachybotrys chartarum*	KEY69576
Dehydrogenase	823	1e-138	84	46.63	*Ustilaginoidea virens*	QUC21319
Hypothetical protein	1331	0	99	44.89	*Fusarium mundagurra*	KAF5705177

9) *Verticillium dahliae* contig NW_009276967, VRPKS-I-17 cluster

Gene name	Size	E value	% Coverage	% identity	Top blast hit	Accession number of top blast hit
Golgi to ER traffic protein	333	2e-180	100	78.44	Colletotrichum gloeosporioides	KAF3811196
putative 6-phosphofructo-2-kinase/fructose-2 like protein	572	0	93	88.60	Colletotrichum karsti	XP_038750648
Hydrolase	823	0	100	62.98	Colletotrichum scovillei	XP_035333467
54S ribosomal protein	352	0	99	73.14	Sodiomyces alkalinus	XP_028466988
Hsp 70 nucleotide exchange factor	209	3e-98	97	71.57	Sodiomyces alkalinus	XP_028466987
Actin	251	1e-175	94	99.16	Acinetobacter baumannii	WP_147504702
ABC transporter	1302	0	97	67.79	Colletotrichum chlorophyti	OLN85600
Esterase/Hypothetical protein	255	9e-151	100	78.52	Colletotrichum sojae	KAF6806434
PKS	2290	0	99	70.46	Colletotrichum chlorophyti	OLN86478
Serine palmitoyltransferase like protein/Hypothetical protein	114	3e-44	78	76.67	Sodiomyces alkalinus	XP_028466984
Elongator complex protein	824	0	99	66.79	Colletotrichum tofieldiae	KZL71342
Ubiquitin modifier	104	1e-56	99	79.61	Claviceps pusilla	KAG6012378
Zinc finger protein (transcription factor)	249	2e-94	89	60.26	Colletotrichum musicola	KAF6845115
Asparagine zinc finger protein	159	4e-07	71	33.63	Colletotrichum incanum	OHW91750
Mitochondrial FAD carrier protein	196	6e-88	94	71.36	Colletotrichum sidae	TEA17977

10) *Verticillium dahliae* contig NW_009276941, VRPKS-I-11 cluster

Gene name	Size	E value	% Coverage	% identity	Top blast hit	Accession number of top blast hit
Putative lyase	231	4e-140	100	82.25	*Cylindrodendrum hubeiense*	KAF7550322
Dibenzothiophene desulfurization enzyme C like protein	428	0	100	79.69	*Neonectria ditissima*	KPM44574
Acetate transporter	202	1e-101	99	77.50	*Aspergillus sclerotiicarbonarius*	PYI02021
FAD binding domain protein	506	0	99	68.25	*Sodiomyces alkalinus*	XP_028469411
Hydrolase	383	3e-168	99	58.40	*Sodiomyces alkalinus*	XP_028463045
Phenol-2 monooxygenase	729	0	99	68.12	*Sodiomyces alkalinus*	XP_028463046
Methyltransferase	514	0	100	69.42	*Sodiomyces alkalinus*	XP_028463047
Myb related protein B	207	4e-08	15	75.76	*Sodiomyces alkalinus*	XP_028463048
Serine 3 dehydrogenase	283	9e-155	98	73.93	*Akanthomyces lecanii*	OAA81050
Non-canonical non-ribosomal peptide synthetase ascB like protein	693	0	100	57.55	*Sodiomyces alkalinus*	XP_028463042
PKS-NRPS	2158	0	76	45.29	*Sodiomyces alkalinus*	XP_028463040
Pectate lyase	243	4e-141	100	80.74	*Monosporascus ibericus*	RYP08594
Hypothetical protein	403	2e-95	98	41.44	*Sodiomyces alkalinus*	XP_028465602
DNA damage binding protein	565	0	100	71.25	*Sodiomyces alkalinus*	XP_028465605
Sterol 3-beta glucosyltransferase	1460	0	99	69.78	*Colletotrichum aengim*	XP_037179391

Hypothetical protein	280	7e-136	95	72.39	*Sporothrix insectorum*	OAA62000

11) *Verticillium albo-atrum* contig NMXJ01000004, VRPKS-I-16 cluster

**Gene name**	**Size**	**E value**	**% Coverage**	**% identity**	**Top blast hit**	**Accession number of top blast hit**
Hypothetical protein	528	0	94	72.75	Fusarium decemcellulare	KAF4998379
Integral membrane protein	384	0	99	71.69	Neonectria ditissima	KPM38699
Amino transferase	380	0	97	78.92	Colletotrichum karsti	XP_038747040
Dihydrodipicolinate synthase family	310	5e-145	100	64.63	Pseudogymnoascus sp	KFZ23593
Hypothetical protein	149	1e-21	96	42.68	Diaporthe batatas	KAG8160464.
Hypothetical protein	131	1e-32	83	50.43	Fusarium gaditjirri	KAF4952502
Glycosyl hydrolase/glycosidase	664	0	99	77.98	Colletotrichum trifolii	TDZ54568
Hypothetical/BTB domain containing protein	289		100	56.12	Pyricularia pennisetigena	XP_029745318
Hypothetical protein	426	0	98	63.92	Stachybotrys chlorohalonata	KFA60422
PAP2 superfamily protein	384	2e-159	99	58.49	Colletotrichum truncatum	XP_036575451
Hypothetical protein	170	3e-06	87	31.06	Colletotrichum sidae	TEA18685
PKS	2718	0	91	46.58	Hyaloscypha variabilis	PMD43053
Integral membrane protein/glycosyltransferase	257	8e-71	93	46.74	Colletotrichum musicola	KAF6837090
Methyl transferase	447	0	99	66.59	Coccidioides posadasii str. Silveira	EFW14833
MFS super family	600	0	95	79.23	Fusarium decemcellulare	KAF5012339
Hypothetical protein	449	1e-171	94	57.38	Pyricularia oryzae	XP_003719045
Thiol specific monooxygenase	507	0	92	75.16	Diaporthe ampelina	KKY36389
lycogen debranching enzyme	660	0	99	68.95	Monosporascus ibericus	RYP03266

12) *Verticillium albo-atrum* contig NMXJ01000009, VRPKS-I-2 cluster

**Gene name**	**Size**	**E value**	**% Coverage**	**% identity**	**Top blast hit**	**Accession number of top blast hit**
Oxidoreductase	680	0	67	68.26	*Colletotrichum truncatum*	XP_036581492
Sterol uptake protein	254	8e-76	98	43.70	*Colletotrichum karsti*	XP_038743400
3-ketoacyl Co-A reductase	322	8e-123	98	55.62	*Tolypocladium ophioglossoides*	KND89420
Hypothetical protein	193	2e-121	100	86.01	*Colletotrichum fructicola*	XP_031875990
Efflux pump/MFS protein	588	0	99	85.52	*Colletotrichum fructicola*	KAF4886521
Hypothetical protein	167	2e-113	100	95.21	*Colletotrichum fructicola*	XP_031875989
Alpha-beta hydrolase	433	0	98	86.89	*Colletotrichum fructicola*	XP_031875995.
PKS-NRPS	3800	0	100	86.09	*Colletotrichum fructicola*	KAF4886526
Hypothetical protein	435	0	100	86.90	*Colletotrichum fructicola*	XP_031875984
Alcohol dehydrogenase	353	0	100	91.78	*Colletotrichum fructicola*	XP_031875992
Acetyltransferase	215	6e-70	100	52.21	*Sodiomyces alkalinus*	XP_028470801
Alcohol dehydrogenase	346	3e-178	98	69.59	*Fusarium oxysporum*	SCO92394
MFS (Maltose permease)	534	0	99	81.77	*Cylindrodendrum hubeiense*	KAF7556152

Hypothetical protein	476	0	98	76.02	*Neonectria ditissima*	KPM41868
Hypothetical protein	301	7e-118	99	55.81	*Fusarium ambrosium*	RSM03635
ABC multidrug transporter	1469	0	99	69.05	*Neonectria ditissima*	KPM39725
Fungal transcription factor	1800	0	36	47.71	*Blastomyces gilchristii*	XP_031581329

13) *Verticillium albo-atrum* contig NMXJ01000001, VRPKS-I-12 cluster

**Gene name**	**Size**	**E value**	**% Coverage**	**% identity**	**Top blast hit**	**Accession number of top blast hit**
Complex I intermediate associated protein	804	0	100	72.90	*Sodiomyces alkalinus*	XP_028469267
Dehydrogenase	283	6e-166	100	84.10	*Colletotrichum fioriniae*	EXF81015
Cytochrome P-450	476	0	97	73.93	*Sodiomyces alkalinus*	XP_028469280
Hydrolase	396	6e-100	92	45.12	*Colletotrichum salicis*	KXH61195
Asparatic protease	436	5e-180	95	56.49	*Colletotrichum orchidophilum*	XP_022470055
FAD binding monooxygenase	426	0	99	70.45	*Colletotrichum scovillei*	KAG7054835
Hypothetical protein/Protein kinase	887	0	99	62.77	*Sodiomyces alkalinus*	XP_028469275
PKS	3888	0	99	74.31	*Sodiomyces alkalinus*	XP_028469272
L 2,4 diaminobutyrate decarboxylase	515	0	93	66.53	*Colletotrichum graminicola*	XP_008093084
DUF 92 domain containing protein	387	2e-163	99	62.79	*Sodiomyces alkalinus*	XP_028469270
Hypothetical protein	320	3e-22	33	44.86	*Verticillium longisporum*	KAG7142964
Multicopper oxidase	561	0	96	67.77	*Colletotrichum tofieldiae*	KZL72261
NR-PKS-I	2188	0	99	79.22	*Colletotrichum gloeosporioides*	EQB55056
Oxidoreductase	325	0	99	85.80	*Colletotrichum truncatum*	XP_036586400
Transcription factor/Hypothetical protein	424	1e-156	100	56.78	*Colletotrichum nymphaeae*	KXH55671
Transcription factor Cmr1	834	0	99	70.82	*Colletotrichum truncatum*	XP_036589502

14) *Verticillium albo-atrum* contig NMXJ01000001, VRPKS-I-9 cluster

**Gene name**	**Size**	**E value**	**% Coverage**	**% identity**	**Top blast hit**	**Accession number of top blast hit**
Hypothetical protein	1129	0	95	59.65	*Sodiomyces alkalinus*	XP_028464300
Galactose transporter	532	0	90	76.53	*Sodiomyces alkalinus*	XP_028464187
Hypothetical protein	207	8e-19	62	45.04	*Colletotrichum sidae*	TEA17102
Phosphopantothenoylcysteine decarboxylase	610	0	99	79.17	*Sodiomyces alkalinus*	XP_028464284
Hypothetical protein	400	8e-31	66	39.23	*Geosmithia morbida*	XP_035320824
PKS-NRPS	3644	0	99	49.78	*Monosporascus sp*	RYP45085
ABC like protein	503	0	91	82.03	*Colletotrichum chlorophyti*	OLN84498
Galactosyl transferase	329	4e-153	84	71.97	*Sodiomyces alkalinus*	XP_028468709
40S ribosomal protein	256	0	100	96.88	*Sodiomyces alkalinus*	XP_028468707
GTP-cyclohydrolase	427	0	97	60.41	*Akanthomyces lecanii*	OAA77453

15) *Verticillium albo-atrum* contig NMXJ01000001, VRPKS-I-17 cluster

**Gene name**	**Size**	**E value**	**% Coverage**	**% identity**	**Top blast hit**	**Accession number of top blast hit**

Hypothetical protein	221	1e-41	58	51.52	*Fusarium albosuccineum*	KAF4462046
Isoflavone reductase	302	4e-92	99	48.003	*Colletotrichum higginsianum*	XP_018160455
Mitochondrial import receptor	352	0	100	83.62	*Sodiomyces alkalinus*	XP_028466992
6-phosphofructo2-kinase	570	0	93	86.36	*Colletotrichum karsti*	XP_038750648
Hydrolase/Hypothetical protein	827	0	100	63.56	*Colletotrichum orbiculare*	TDZ22411
54S ribosomal protein	356	0	98	76.42	*Sodiomyces alkalinus*	XP_028466988
HSP70 nucleotide exchange factor	209	6e-101	97	72.55	*Sodiomyces alkalinus*	XP_028466987
Actin	375	0	100	100	*Tolypocladium paradoxum*	POR36442
Peptidyl t-RNA hydrolase	152	8e-55	78	72.58	*Purpureocillium lilacinum*	XP_018176407
ABC transporter	1267	0	98	68.24	*Colletotrichum sojae*	KAF6806433
Esterase	255	6e-154	100	80.86	*Colletotrichum sojae*	KAF6806434
PKS	2277	0	99	71.66	*Colletotrichum truncatum*	XP_036585096
Hypothetical protein/Serine palmitoyltransferase domain containing protein	113	3e-49	100	71.68	*Sodiomyces alkalinus*	XP_028466984
Elongator protein	821	0	99	70.85	*Colletotrichum truncatum*	XP_036582858
Ubiquitine related modifier	104	1e-155	99	79.61	*Cordyceps militaris*	XP_006668991
Zinc finger protein	445	3e-128	91	48.72	*Colletotrichum incanum*	OHW91750
Mitochondrial FAD carrier protein	310	1e-167	99	73.68	*Colletotrichum chlorophyti*	OLN84903
Splicing factor	373	8e-178	85	81.50	*Colletotrichum camelliae*	KAG2106276

16) *Verticillium albo-atrum* contig NMXJ01000001, VRPKS-I-23 cluster

Gene name	Size	E value	% Coverage	% identity	Top blast hit	Accession number of top blast hit
Myosin class II heavy chain protein	2138	0	100	63.13	*Sodiomyces alkalinus*	XP_028465411
SRP54 domain containing protein	669	0	99	80.89	*Sodiomyces alkalinus*	XP_028465413
ER to Golgi transporter	323	0	99	85.98	*Colletotrichum truncatum*	XP_036588854
MPV17/PMP22 family protein	231	8e-102	98	63.16	*Colletotrichum musicola*	KAF6806760
Stress responsive A/B barrel domain containing protein	199	2e-56	56	72.57	*Sodiomyces alkalinus*	XP_028465416
HSP90 like protein	1392	0	99	67	*Sodiomyces alkalinus*	XP_028465417
Efflux pump/transporter	588	0	97	51.30	*Fusarium graminum*	KAF4989526
R-PKS-I	2651	0	89	51.24	*Sodiomyces alkalinus*	XP_028462492
Tryptophan dimethylallyltransferase domain containing protein	456	0	97	60.49	*Colletotrichum truncatum*	XP_036588933
Hypothetical protein	361	2e-113	100	49.48	*Sodiomyces alkalinus*	XP_028462491
Hypothetical protein	550	8e-143	95	47.70	*Fusarium sp.*	RSL49290
Hypothetical protein	250	3e-06	62	33.33	*Sodiomyces alkalinus*	XP_028462488
GATA zinc finger domain containing protein	119	1e-16	36	75	*Colletotrichum tanaceti*	TKW48536
Homoserine kinase	356	0	99	90.45	*Colletotrichum scovillei*	XP_035325878
pfkB family kinase	367	4e-64	98	38.34	*Sodiomyces alkalinus*	XP_028463289

Hypothetical protein	194	1e-53	95	55.73	*Sodiomyces alkalinus*	XP_028463291

17) *Verticillium albo-atrum* contig NMXJ01000003, VRPKS-I-25 cluster

Gene name	Size	E value	% Coverage	% identity	Top blast hit	Accession number of top blast hit
Hypothetical protein	1064	0	68	51.05	*Colletotrichum incanum*	KZL75670
Fungal specific transcription factor	774	0	93	63.44	*Colletotrichum scovillei*	XP_035329228
Protein OS-9 like protein	528	0	99	60	*Colletotrichum spinosum*	TDZ38446
Key lime pathogenicity protein/hypothetical protein	676	1e-179	98		*Colletotrichum fructicola*	XP_031885775
S-adenosyl-Lmethionine dependent methyltransferase	315	6e-170	100	76.19	*Sodiomyces alkalinus*	XP_028466391
Dihydroxy acid dehydratase	647	0	100	72.15	*Sodiomyces alkalinus*	XP_028466806
Zinc binding dehydrogenase/Hypothetical protein	294	8e-167	99		*Stachybotrys chartarum*	KEY68920
PKS	2103	0	88	73.91	*Stachybotrys chartarum*	KEY68922
Hydrolase/xylosidase domain containing protein	1089	0	52	85.44	*Colletotrichum truncatum*	XP_036575294
Monooxygenase	487	0	98	70.39	*Colletotrichum asianum*	KAF0322960
STB-5 protein	751	0	99	58.41	*Colletotrichum chlorophyti*	OLN81943
Sodium transport ATPase	1071	0	98	74.95	*Monosporascus sp.*	RYP34097
Bifunctional polynucleotide phosphatase/kinase	456	0	99	70.28	*Sodiomyces alkalinus*	XP_028468241
RNA polymerase II mediator complex subunit	269	2e-161	98	78.57	*Sodiomyces alkalinus*	XP_028468239
Hypothetical protein	426	1e-97	97	41.40	*Cylindrodendrum hubeiense*	KAF7557101
Hypothetical protein	568	0	100	57.22	*Colletotrichum fioriniae*	EXF84616

*No significant similarity found

18) *Verticillium albo-atrum* contig NMXJ01000003, VRPKS-I-24 cluster

Gene name	Size	E value	% Coverage	% identity	Top blast hit	Accession number of top blast hit
Ubiquitine carboxyl-terminal hydrolase	247	2e-146	99	83.33	*Sodiomyces alkalinus*	XP_028469647
RNA helicase	726	0	96	72.07	*Sodiomyces alkalinus*	XP_028469646
Synaptobrevin	237	9e-147	100	90.72	*Sodiomyces alkalinus*	XP_028469645
Hypothetical protein	193	1e-37	85	45.71	*Fusarium oxysporum f. sp. pisi*	EXA28620
Hypothetical protein	108	4e-11	69	46.25	*Trichoderma gamsii*	XP_024405574
Dehydrogenase	430	0	93	73.02	*Colletotrichum plurivorum*	KAF6833458
Hypothetical protein	241	5e-103	95	64.96	*Sodiomyces alkalinus*	XP_028469642
Sec1 family protein	738	0	99	67.52	*Sodiomyces alkalinus*	XP_028469641
Hypothetical protein	805	0	92	63.83	*Colletotrichum karsti*	XP_038751043
Arylsulfatase	591	0	93	68.09	*Fusarium albosuccineum*	KAF4467271
Cytochrome P-450	562	2e-161	99	42.14	*Hypoxylon sp.*	OTA56529

R-PKS-I	2453	0	99	63.30	*Cylindrodendrum hubeiense*	KAF7552999
Hypothetical protein	143	NSS*	0	0	NSS*	NSS*
Cutinase like protein	292	4e-141	90	78.87	*Cadophora sp.*	PVH70228
Major facilitator superfamily transporter	525	0	100	85.71	*Fusarium oxysporum f. sp.rapae*	KAG7404936
Hypothetical protein	1125	0	99	69.64	*Sodiomyces alkalinus*	XP_028469639
Mitosis protein dim1	143	2e-95	100	91.61	*Colletotrichum plurivorum*	KAF6833464
Cell morphogenesis protein	2575	0	99	79.85	*Colletotrichum trifolii*	TDZ47227

19) *Verticillium albo-atrum* contig NMXJ01000003, VRPKS-I-19 cluster

Gene name	Size	E value	% Coverage	% identity	Top blast hit	Accession number of top blast hit
Methyltransferase/Hypothetical protein	275	6e-105	99	54.38	*Fusarium gaditjirri*	KAF4945087
Hypothetical protein	160	5e-60	95	58.55	*Colletotrichum karsti*	XP_038750884
Hypothetical protein	77	2e-21	97	55.13	*Sodiomyces alkalinus*	XP_028465568
Hypothetical protein	300	2e-82	92	48.56	*Acremonium chrysogenum*	KFH42767
N-glycosidase like protein	213	2e-55	84	51.50	*Chaetomium globosum*	XP_001221113
Mitochondrial inner membrane translocase	541	0	100	75.87	*Sodiomyces alkalinus*	XP_028468965
37S ribosomal protein	250	2e-97	100	59.36	*Colletotrichum nymphaeae*	KXH60786
DeSL like protein sdu1	232	2e-134	99	80.09	*Colletotrichum chlorophyti*	OLN97068
Obg like ATPase	394	0	100	90.10	*Pyricularia grisea*	XP_030984371
Exopolygalacturonase	814	0	55	69.44	*Monosporascus sp.*	RYP36431
Hypothetical protein	519	1e-81	85	38.81	*Neonectria ditissima*	KPM41855
Thioredoxin domain containing protein	162	1e-84	98	76.73	*Hypoxylon sp.*	OTA67286
Major facilitator superfamily transporter	572	0	98	71.45	*Sodiomyces alkalinus*	XP_028468956
Hypothetical protein	301	1e-125	100	58.61	*Claviceps purpurea*	KAG6238209
PKS-I	2108	0	99	63.26	*Epichloe festucae*	QPH02303
Hypothetical protein	1279	0	99	77.60	*Monosporascus sp.*	RYP73509
L-theronate dehydrogenase	1135	0	98	74	*Colletotrichum siamense*	KAF4818215
Fungal specific transcription factor	687	0	100	64.74	*Colletotrichum graminicola*	XP_008095606
Major facilitator superfamily transporter	488	0	98	78.38	*Colletotrichum incanum*	OHW90295
Hydrolase	349	4e-122	100	51	*Pyrenochaeta sp.*	OAL42911
Cutinase	224	1e-113	90	79.80	*Stachybotrys chlorohalonata*	KFA64195
Vacuolar protein sorting associated protein	757	0	98	71.71	*Colletotrichum sidae*	

20) *Verticillium albo-atrum* contig NMXJ01000006, VRPKS-I-5 cluster

Gene name	Size	E value	% Coverage	% identity	Top blast hit	Accession number of top blast hit
Phosphatidylinositol N-acetylglucosaminyltransferase	222	2e-80	100	58.30	*Colletotrichum incanum*	KZL82123
Hypothetical protein	1615	0	98	71.71	*Sodiomyces alkalinus*	XP_028471323
Efflux pump/MFS protein	612	0	93	68.47	*Glarea lozoyensis*	XP_008088684
Hypothetical protein	181	1e-64	88	58.64	*Botryotinia calthae*	TEY65540

Hypothetical protein	490	2e-95	98	41.85	*Botryotinia squamosa*	KAF7857384
PKS-NRPS	4017	0	100	62.50	*Botrytis tulipae*	TGO20046
Hypothetical protein	298	2e-28	47	45.14	*Fusarium ambrosium*	RSM05402
Hypothetical protein	137	2e-77	100	83.21	*Purpureocillium lilacinum*	XP_018184317
Hypothetical protein	157	2e-50	100	62.58	*Colletotrichum graminicola*	XP_008094022
Isochorismatase/hydrolase	244	7e-131	99	77.78	*Colletotrichum truncatum*	XP_036585971
NSS*	NSS	NSS	NSS	NSS	NSS	NSS

*No significant similarity found

21) *Verticillium albo-atrum* contig NMXJ01000007, VRPKS-I-26 cluster

Gene name	Size	E value	% Coverage	% identity	Top blast hit	Accession number of top blast hit
Hypothetical protein	420	1e-88	84	43.70	*Colletotrichum tofieldiae*	KZL73082
Ankyrin repeat protein	810	8e-62	76	28.68	*Colletotrichum truncatum*	XP_036575058
NSS*	NSS	NSS	NSS	NSS	NSS	NSS
Thiol methyltransferase/hypothetical	78	1e-30	100	70.51	*Verticillium longisporum*	KAG7128937
Peptidase domain containing protein	1015	0	98	55.06	*Microbacterium sp.*	WP_144806847
Hypothetical protein	578	0	100	88.08	*Colletotrichum higginsianum*	XP_018157666.
Hypothetical protein	381	4e-96	85	61.20	*Exophiala spinifera*	XP_016240708
Pectate lyase F	236	5e-141	97	83.98	*Colletotrichum fructicola*	XP_031890093
Hypothetical protein	1004	0	100	71.14	*Colletotrichum chlorophyti*	OLN96860
Hypothetical protein	163	2e-60	100	53.99	*Colletotrichum simmondsii*	KXH28221
Hypothetical protein	148	1e-10	54	45	*Diaporthe batatas*	KAG8165617
PKS-I	2326	0	78	56.29	*Monosporascus sp.*	RYP50633
Short chain dehydrogenase	330	9e-174	93	72.73	*Stachybotrys chartarum*	KFA49246
Hypothetical protein	130	2e-58	100	71.54	*Fusarium longipes*	RGP77546
Leucine aminopeptidase	373	0	100	74.26	*Cylindrodendrum hubeiense*	KAF7555029
Hypothetical protein	497	3e-89	74	54.23	*Colletotrichum gloeosporioides*	KAF3801861
Hypothetical protein/Midasin	342	3e-18	29	63.46	*Colletotrichum siamense*	XP_036488857
Hypothetical protein	200	8e-94	99	59.32	*Sodiomyces alkalinus*	XP_028470403
Hypothetical protein	126	5e-22	53	70.15	*Hypoxylon sp.*	OTA94229

*No significant similarity found

22) *Verticillium albo-atrum* contig NMXJ01000002, VRPKS-I-3 cluster

Gene name	Size	E value	% Coverage	% identity	Top blast hit	Accession number of top blast hit
T-complex protein 1	547	0	100	91.59	*Colletotrichum chlorophyti*	OLN83711
HMG box protein	394	1e-105	98	54.36	*Colletotrichum orchidophilum*	XP_022468387
Sporulation protein rmd1	514	0	99	81.54	*Colletotrichum asianum*	KAF0325559
Dehydrogenase/hypothetical protein	135	1e-13	71	38.14	*Pyricularia sp.*	TLD33949
Saccharopine dehydrogenase	414	0	99	65.47	*Sodiomyces alkalinus*	XP_028468308
60S ribosomal protein	110	2e-54	78	97.67	*Colletotrichum sojae*	KAF6820981
Hypothetical protein	408	7e-49	79	34.65	*Magnaporthiopsis poae*	KLU90970
PKS-NRPS	4677	0	86	67.40	*Colletotrichum truncatum*	XP_036576988

Cytochrome P-450 oxidoreductase	496	0	100	82.46	*Colletotrichum truncatum*	XP_036576987
Cytochrome P-450	501	0	99	70.57	*Colletotrichum truncatum*	XP_036576986
Trans-enoyl reductase	355	0	100	77.87	*Colletotrichum truncatum*	XP_036576985
Diels alderase	425	0	100	71.76	*Colletotrichum siamense*	KAF4852455
Alpha-beta hydrolase	447	0	100	79.69	*Colletotrichum truncatum*	XP_036576983
Thioredoxin domain containing protein	245	9e-120	98	68.60	*Colletotrichum sublineola*	KDN69576
C-6 finger domain containing protein	619	1e-127	94	40.76	*Colletotrichum higginsianum*	XP_018161555
UPF0045 protein	115	1e-61	97		*Colletotrichum gloeosporioides*	KAF3804039
Hypothetical protein	437	0	100	67.73	*Colletotrichum trifolii*	TDZ68279
Serine threonine protein kinase	548	0	99	61.40	*Colletotrichum karsti*	XP_038743611
Hypothetical protein	268	2e-101	100	65.93	*Colletotrichum trifolii*	TDZ68275
Kelch repeat containing protein	711	0	68	62.42	*Colletotrichum chlorophyti*	OLN85773

23) *Verticillium albo-atrum* contig NW_NMXJ01000005, PKS-III

**Gene name**	**Size**	**E value**	**% Coverage**	**% identity**	**Top blast hit**	**Accession number of top blast hit**
Dioxygenase	395	0	100	74.43	*Colletotrichum sojae*	KAF6820147
Transporter	585	0	96	77.03	*Fusarium mundagurra*	KAF5696253
Hypothetical protein	372	1e-50	83	37.78	*Trichoderma guizhouense*	OPB42090
Carbonic anhydrase	271	4e-152	98	75.94	*Colletotrichum incanum*	KZL87566
Hypothetical protein	265	8e-10	37	33.98	*Ophiocordyceps sinensis*	EQL01270
Acetyltransferase	268	9e-152	93	79.68	*Colletotrichum chlorophyti*	OLN95961
Oxidoreductase	451	0	99	61.38	*Colletotrichum higginsianum*	XP_018159254
NSS*	495	NSS	NSS	NSS	NSS	NSS
Feruloyl esterase	295	7e-153	98	71.48	*Colletotrichum orbiculare*	TDZ25527
Fungal specific transcription factor	804	0	100	72.75	*Colletotrichum higginsianum*	TID06918
BFR2 protein	588	0	100	63.13	*Sodiomyces alkalinus*	XP_028470322
Hypothetical protein	136	2e-36	96	52.67	*Colletotrichum spinosum*	TDZ30131
PKS-III	454	0	100	73.79	*Colletotrichum simmondsii*	KXH46773
Fructose bisphosphate aldolase	362	0	100	88.67	*Sodiomyces alkalinus*	XP_028470318
Specific exonuclease	1306	0	85	77.41	*Sodiomyces alkalinus*	XP_028470311

*No significant similarity found

24) *Verticillium alfalfae* contig NW_003315037, VRPKS-I-22 cluster

**Gene name**	**Size**	**E value**	**% Coverage**	**% identity**	**Top blast hit**	**Accession number of top blast hit**
RNA export protein	358	0	100	82.12	Colletotrichum musicola	KAF6822509
Hypothetical protein	297	1e-164	100	80.81	Sodiomyces alkalinus	XP_028463293
Ethanonamine-phosphate cytidylyltransferase like protein	430	0	99	80.22	Colletotrichum spinosum	TDZ39069
Aminotransferase	394	0	100	65.81	Sodiomyces alkalinus	XP_028463295
Hypothetical protein	285	9e-08	69	28.29	Sodiomyces alkalinus	XP_028463296
Peroxiredoxin	412	2e-160	78	65.23	*Plectosphaerella cucumerina*	KAH7362586
Methonine aminopeptidase	448	0	99	75.95	*Colletotrichum sidae*	TEA17142

MSF-1	192	2e-120	98	85.42	Sodiomyces alkalinus	XP_028463299
Hypothetical protein	131		95	59.20	*Plectosphaerella cucumerina*	KAH7362585
Hypothetical protein	322	5e-25	95	29.13	Fusarium equiseti	CAG7565864
Lactamase	300	1e-137	100	62	Monosporascus sp.	RYP11973
Sulfate permease	822	0	95	52.89	Massariosphaeria phaeospora	KAF2866440
PKS-I	2554	0	95	70.42	Fusarium miscanthi	ALQ32877
Glucosyl transferase	553	0	98	59.45	Fusarium austroafricanum	KAF4443747
Esterase/Hydrolase	207	7e-99	100	64.22	Fusarium nygamai	PNP83713
Monooxygenase	389	0	94	72.36	Bimuria novae-zelandiae	KAF1970089
E-fflux pump	408	0	97	75.76	Colletotrichum graminicola	XP_008093595
Oxidoreductase	372	5e-133	90	54.84	Fusarium sarcochroum	KAF4960431
Hypothetical protein	253	2e-124	100	70.36	*Plectosphaerella cucumerina*	KAH7357817
Hypothetical protein	883	0	100	94.11	Verticillium nonalfalfae	XP_028494824
Hypothetical protein	90	1e-37	97	75	Pseudomassariella vexata	XP_040719305
Tubulin gamma chain protein	461	0	100	96	Sodiomyces alkalinus	XP_028463304

25) *Verticillium alfalfae* contig NW_003315037, VRPKS-I-23 cluster

Gene name	Size	E value	% Coverage	% identity	Top blast hit	Accession number of top blast hit
Myosin class II	2204	0	91	57.77	*Sodiomyces alkalinus*	XP_028465411
Signal recognition receptor	652	0	99	74.15	*Sodiomyces alkalinus*	XP_028465413
Transport protein	352	0	100	85.54	*Colletotrichum simmondsii*	KXH42740
PXMP 2/4 family protein	255	4e-142	100	57.25	*Colletotrichum musicola*	KAF6806760
Virulence sensor protein/Heat shock protein 90 (Hsp90)	1660	0	84	67.05	*Sodiomyces alkalinus*	XP_028465417
Drug resistance transporter	485	0	99	53.12	*Stylonectria norvegica*	KAF7555861
PKS	2402	0	100	49.71	*Sodiomyces alkalinus*	XP_028462492
Hypothetical protein	363	1e-98	99	48.44	*Sodiomyces alkalinus*	XP_028462491
Cocaine esterase	600	0	94	60.27	*Sodiomyces alkalinus*	XP_028462489
Hypothetical protein	242	2e-86	66	30.65	*Sodiomyces alkalinus*	XP_028462488
SnodProt1	138	1e-79	99	64.49	*Hirsutella minnesotensis*	KJZ76905
Hypothetical protein	146	1e-94	100	100	*Verticillium dahliae*	XP_009656175
Homoserine kinase	355	0	100	91.89	*Colletotrichum tofieldiae*	KZL71078
Hypothetical protein	351	1e-59	100	36.86	*Sodiomyces alkalinus*	XP_028463289

26) *Verticillium alfalfae* contig NW_003315034, VRPKS-I-9 and VRPKS-I-11 cluster

Gene name	Size	E value	% Coverage	% identity	Top blast hit	Accession number of top blast hit
Thioredoxin	410	7e-169	63	73.21	*Colletotrichum higginsianum*	XP_018163738
X-PRO DIPEPTIDYL-PEPTIDASE PROTEIN	300	1e-147	100	69.10	Fusarium oxysporum f. sp. melonis	EXK26648
Short chain dehydrogenase	228	9e-157	100	51.19	Plectosphaerella cucumerina	KAH7375436
Ankyrin repeat domain containing protein	317	3e-15	67	47.69	*Sordaria macrospora*	XP_003349884

Efflux pump transporter	483	0	99	53.55	Lasiodiplodia theobromae	XP_035374841
Short chain dehydrogenase	287	3e-96	99	56.14	Hirsutella minnesotensis	KJZ71235
Hypothetical protein	204	3e-52	97	38.33	Lasiodiplodia theobromae	XP_035374837
PKS-NRPS C	3529	0	99	39.48	Lasiodiplodia theobromae	KAF963027
Fungal specific transcription factor	819	5e-109	59	37.32	Ilyonectria destructans	KAH6995025
Hypothetical protein	435	2e-17	40	34.46	Plectosphaerella cucumerina	KAH7368326
PKS-NRPS D	3898	0	97	70.54	*Hirsutella rhossilie*	XP_044717051
Mg transporter/Hypothetical protein	1143	3e-119	56	34.42	Colletotrichum truncatum	XP_036585489
GTPase activator protein (Rho GAP)	238	0	72	65.47	*Colletotrichum incanum*	KZL81424

27) *Verticillium alfalfae* contig NW_003315038, VRPKS-I-17 cluster

Gene name	Size	E value	% Coverage	% identity	Top blast hit	Accession number of top blast hit
Golgi to ER traffic protein	333	0	100	78.81	*Colletotrichum gloeosporioides*	KAF3811196
putative mitochondrial import receptor	352	0	100	83.90	*Sodiomyces alkalinus*	XP_028466992
putative 6-phosphofructo-2-kinase/fructose-2 like protein	574	0	100	84.54	*Plectosphaerella cucumerina*	KAH7363100
Hydrolase	697	3e-144	99	71.11	*Plectosphaerella cucumerina*	KAH7363099
54S ribosomal protein	352	0	99	73.14	*Sodiomyces alkalinus*	XP_028466988
Hsp 70 nucleotide exchange factor	172	3e-125	98	72.38	*Sodiomyces alkalinus*	XP_028466987
Actin	375	0	100	100	*Tolypocladium paradoxum*	POR36442
ABC transporter	1326	0	99	72.84	*Colletotrichum chlorophyti*	OLN85600
Esterase/Hypothetical protein	255	3e-151	100	79.30	*Colletotrichum sojae*	KAF6806434
R-PKS-I	2391	0	99	70.58	*Colletotrichum chlorophyti*	OLN86478
Elongator complex protein	836	0	99	62.92	*Colletotrichum tofieldiae*	KZL71342
Ubiquitin modifier	104	1e-56	99	79.61	*Claviceps pusilla*	KAG6012378
Zinc finger protein (transcription factor)	439	2e-135	94	59.70	*Colletotrichum musicola*	KAF6845115
Mitochondrial FAD carrier protein	196	3e-148	98	68.63	*Colletotrichum sidae*	TEA17977

28) *Verticillium alfalfae* contig NW_003315038, VNRPKS-I-1 cluster

Gene name	Size	E value	% Coverage	% identity	Top blast hit	Accession number of top blast hit
Carrier Protein	225	3e-126	96	76.39	*Plectosphaerella cucumerina*	KAH7358165
U3 small nucleolar ribonucleoprotein IMP3	183	8e-117	100	88.52	*Sodiomyces alkalinus*	XP_028469265
37S ribosomal protein	207	5e-95	96	68.84	*Sodiomyces alkalinus*	XP_028469266
Complex I intermediate associate protein	710	0	99	74.16	*Sodiomyces alkalinus*	XP_028469267
Tetrahydroxynaphthalene reductase	284	1e-126	100	82.52	*Colletotrichum fioriniae*	EXF81015
L 2,4 diaminobutyrate decarboxylase	448	0	96	59.36	*Colletotrichum sidae*	TEA12782
Hypothetical protein	201	NA	NA	NA	NA	NA
Multicopper oxidase	549	0	90	66.34	*Colletotrichum tofieldiae*	KZL72261
NR-PKS-I	2088	0	100	77.51	*Colletotrichum karsti*	XP_038751444
Oxidoreductase/Hypothetical protein	301	1e-117	99	71.17	*Colletotrichum truncatum*	XP_036586400
Hypothetical protein	88	NA	NA	NA	NA	NA

Transcription factor CMR1	578	0	96	67.63	*Colletotrichum truncatum*	XP_036589502
Fungal specific transcription factor	254		95	57.83	*Colletotrichum incanum*	OHW97099
Glycosyl transferase	325	4e-175	100	74.54	*Plectosphaerella cucumerina*	KAH7358014
Dihydrodipicolinate synthetase family	143	4e-56	88	68.50	*Fusarium fujikuroi*	KLP22936

29) *Verticillium alfalfae* contig NW_003315038, VRPKS-I-1 cluster

**Gene name**	**Size**	**E value**	**% Coverage**	**% identity**	**Top blast hit**	**Accession number of top blast hit**
Protein SYM1	284	3e-125	83	74.49	*Colletotrichum musicola*	KAF6800315
Hypothetical protein	298	1e-91	94	59.45	*Plectosphaerella cucumerina*	KAH7369210
Hypothetical protein	381	1e-115	99	59.25	*Plectosphaerella cucumerina*	KAH7369210
Oxidoreductase	332	0	100	75	*Stachybotrys chartarum*	KFA54517
Hypothetical protein	154	3e-73	100	69.48	*Aspergillus udagawae*	GFF25928
Hypothetical protein	198	2e-60	92	55.19	*Diaporthe helianthi*	POS73388
R-PKS-NRPS	3750	0	99	52.92	*Massariosphaeria phaeosp*	KAF2874833
Hypothetical protein	330	5e-81	100	40.84	*Phialocephala subalpina*	CZR58350
Hydrolase/aminopeptidase	367	5e-95	94	49.43	*Tothia fuscella*	KAF2431630
Enoyl reductase	195	5e-48	91	55.56	*Aspergillus hiratsukae*	KAF7118427
Hypothetical protein	195	2e-142	96	40.59	*Lasiodiplodia theobromae*	KAB2573329
Transcription factor (Isoflavipucine cluster transcription factor)	266	6e-52	56	56.21	*Torrubiella hemipterig*	CEJ81614
Efflux pump/MFS-type efflux transporter	586	0	92	70.59	*Torrubiella hemipte*	CEJ81615
Hypothetical protein/hydrolase	219	1e-58	65	52.05	*Sodiomyces alkalinus*	XP_028470755
AP-3 complex subunit beta-2 like protein	304	4e-86	97	78.98	*Colletotrichum truncatum*	XP_036580440
RTA-1	333	2e-112	99	52.51	*Colletotrichum chlorophyti*	OLN87357

30) *Verticillium alfalfae* contig NW_003315032, VRPKS-I-19 cluster

**Gene name**	**Size**	**E value**	**% Coverage**	**% identity**	**Top blast hit**	**Accession number of top blast hit**
Extracellular serine rich protein	216	9e-150	86	70.90	*Colletotrichum higginsianum*	XP_018161331
Hypothetical protein	77	3e-21	97	55.13	*Sodiomyces alkalinus*	XP_028465568
Hypothetical protein	288	5e-65	95	46.72	*Acremonium chrysogenum*	KFH42767
NADH ubiquinone oxidoreductase	151	9e-36	79	57.50	*Thermothielavioides terrestris*	SPQ17832
Mitochondrial inner membrane translocase	544	0	100	75.61	*Sodiomyces alkalinu*	XP_028468965
Ribosomal protein	89	3e-31	85	71.50	*Colletotrichum fioriniae*	EXF75199.
Obg like ATPase	394	0	100	88.83	*Claviceps africana*	KAG5929667
Exopolygalacturonase/Thioredoxin domain	364	2e-178	97	63.90	*Monosporascus sp*	RYP54678
Thioredoxin	162	8e-79	98	71.25	*Colletotrichum orchidophilum*	XP_022480245
Ammonium transporter/drug resistance protein	576	0	98	69.80	*Sodiomyces alkalinus*	XP_028468956
PKS-I (mycocerosic acid synthase)	243	9e-82	98	50.35	*Claviceps lovelessii*	KAG6015049
R-PKS-I	1524	0	100	57.60	*Claviceps citrina*	KAG6041353
Esterase	237	2e-63	96	49.67	*Claviceps citrina*	KAG6041354
L-theronate dehydrogenase	920	0	98	56.95	*Colletotrichum orchidophilum*	KAF4986227
Fungal specific transcription factor	623	0	100	65.63	*Plectosphaerella cucumerina*	KAH7363480

MFS transporter	488	0	99	76.18	*Colletotrichum chlorophyti*	OLN87319
Chromosome monooxygenase/chromosome P450	520	3e-113	84	39.86	*Colletotrichum orchidophilum*	XP_022469251
Alpha/beta hydrolase	346	3e-117	98	53.80	*Zopfia rhizophila*	KAF2180147
Cutinase	224	9e-116	91	80.39	*Sodiomyces alkalinus*	XP_028468955
Vacuolar protein sorting associate protein 9	770	0	98	69.56	*Colletotrichum orbiculare*	TDZ20835
Hypothetical protein	477	2e-32	66	44.80	*Colletotrichum tofieldiae*	KZL68686
Acetylxylan esterase 2 like protein	566	3e-71	66	58.29	*Colletotrichum siamense*	KAF4811263

31) *Verticillium alfalfae* contig NW_003315025, VRPKS-I-26 cluster

Gene name	Size	E value	% Coverage	% identity	Top blast hit	Accession number of top blast hit
Short chain dehydrogenase	292	4e-124	99	59.45	*Colletotrichum siamense*	XP_036493403
Ankyrin repeat protein	1140	2e-108	62	34.87	*Plectosphaerella cucumerina*	KAH7362763
Hypothetical protein	1017	0	96	56.17	*Paraphoma chrysanthemicola*	KAH7091334
Hypothetical protein	577	0	100	86.70	*Colletotrichum higginsianum*	XP_018157666
Integral membrane protein	365	7e-168	99	69.56	*Colletotrichum incanum*	KZL85847
Dehydrogenase/oxidoreductase	582	0	96	75.62	*Colletotrichum sublineola*	KDN72067
Hypothetical protein	1241	0	69	71.30	*Paraphoma chrysanthemicola*	KAH7362743
PKS-I	852	0	94	65.31	*Monosporascus sp*	RYP50633
Tubulin tyrosine ligase protein	472	0	99	71.30	*Coniochaeta sp.*	KAB5518934
Short chain dehydrogenase	581	4e-164	50	69.77	*Dactylonectria estremocensis*	KAH7144232
LEA domain containing protein	518	0	97	43	*Colletotrichum orchidophilum*	XP_022467882

32) *Verticillium alfalfae* contig NW_003315023, VRPKS-I-23 cluster

Gene name	Size	E value	% Coverage	% identity	Top blast hit	Accession number of top blast hit
FAD binding domain/Acetate transporter	667	0	68	68.47	*Sodiomyces alkalinus*	XP_028469411
Hydrolase	381	1e-97	96	58.97	*Sodiomyces alkalinus*	XP_028463045
Phenol-2 monooxygenase	390	4e-114	99	70.56	*Sodiomyces alkalinus*	XP_028463046
Methyltransferase	430	0	100	60.29	*Sodiomyces alkalinus*	XP_028463047
Myb related protein B	421	3e-93	100	42.51	*Sodiomyces alkalinus*	XP_028463048
3-oxoacyl reductase	283	4e-155	98	74.29	*Akanthomyces lecanii*	OAA81050
Non-canonical non-ribosomal peptide synthetase ascB like protein	798	0	98	46.68	*Sodiomyces alkalinus*	XP_028463042
Phenolppthiocerol synthesis PKS (ppsA)	1911	0	94	56.33	*Tolypocladium ophioglossoides*	KND89106
Putative transporter	119	2e-38	85.54	68	*Colletotrichum tofieldiae*	KZL65137
MFS	343	0	96	88.22	*Colletotrichum chlorophyti*	OLN93090
Pectate lyase	243	2e-154	100	93	*Plectosphaerella cucumerina*	KAH7359289
Hypothetical protein	403	6e-100	99	45.82	*Plectosphaerella cucumerina*	KAH7349693
DNA damage binding protein	565	0	99	73.27	*Plectosphaerella cucumerina*	KAH7349692
Sterol 3-beta glucosyltransferase	1359	0	97	68.71	*Colletotrichum tropicale*	KAF4825980
Cytochrome monooxygenase	274	7e-93	97	52.43	*Colletotrichum higginsianum*	TIC94206
Hypothetical protein	260	7e-115	95	65.30	*Sporothrix insectorum*	OAA62000

33) *Verticillium alfalfae* contig NW_003315025, PKS-III

Gene name	Size	E value	% Coverage	% identity	Top blast hit	Accession number of top blast hit
Carbonic anhydrase	858	1e-141	31	71.54	*Colletotrichum simmondsii*	KXH49803
Acetyltransferase	92	3e-11	38	91.43	*Colletotrichum higginsianum*	XP_018159255
Oxidoreductase	451	0	98	60.54	*Colletotrichum higginsianum*	XP_018159254
Fungal specific transcription factor	813	0	100	70.16	*Colletotrichum incanum*	KZL80546
BFR2 protein	276	1e-123	90	63.41	*Colletotrichum plurivorum*	KAF6823613
PAB dependent poly A specific ribonuclease	1947	0	55	77.73	*Sodiomyces alkalinus*	XP_028470311
Mitochondrial 2 oxoglutarate carrier	335	1e-170	100	86.27	*Colletotrichum chlorophyti*	OLN95994
Hypothetical protein	646	3e-123	98	54.36	*Colletotrichum truncatum*	XP_036586171
Splicing factor spf30	311	2e-148	100	72.81	*Colletotrichum truncatum*	XP_036586172
Iron sulfur protein	137	4e-29	95	75.64	*Colletotrichum plurivorum*	KAF6823606
Cell division control protein	191	4e-95	98	70.37	*Sodiomyces alkalinus*	XP_028470317
NADH ubiquinone oxidoreductase	356	3e-23	43	60	*Plectosphaerella plurivora*	KAH6681192
Hypothetical protein	426	2e-24	61	30.20	*Fusarium longipes*	RGP77896

34) *Verticillium isaacii* contig_NMXN01000002, VRPKS-I-16 cluster

Gene name	Size	E value	% Coverage	% identity	Top blast hit	Accession number of top blast hit
short chain dehydrogenase	332	6.00E-120	98%	59.88%	*Colletotrichum higginsianum IMI 349063*	XP_018159646.1
adenylate kinase	268	2.00E-155	99%	81.34%	*Colletotrichum karsti*	XP_038745165.1
LETM1 domain-containing protein mdm28 like	542	0.00E+00	94%	75.73%	*Plectosphaerella plurivora*	KAH6687845.1
Sorting nexin-4	475	0	100%	80.17%	*Plectosphaerella plurivora*	KAH6687844.1
Kexin like protein	859	0	97%	70.12%	*Plectosphaerella plurivora*	KAH6687843.1
hypothetical protein B0T11DRAFT_7649	1666	0.00E+00	95%	58.45%	*Plectosphaerella cucumerina*	KAH7375268.1
putative RNA-binding proteinc	188	3.00E-62	55%	91.35%	*Colletotrichum liriopes*	GKT41776.1
hypothetical protein F5X68DRAFT_206337	1067	0.00E+00	100%	59.80%	*Plectosphaerella plurivora*	KAH6687840.1
glycosyl hydrolase	638	0.00E+00	94%	65.41%	*Colletotrichum truncatum*	XP_036587401.1
serine hydrolase FSH	239	1.00E-78	100%	58.33%	*Plectosphaerella plurivora*	KAH6673998.1
putative polyketide synthase	2575	0	99%	71.74%	*Plectosphaerella cucumerina*	KAH7369281.1
Alpha/Beta hydrolase protein	557	0.00E+00	98%	65.09%	*Plectosphaerella plurivora*	KAH6688446.1
hypothetical protein ACRE_054030	167	4.00E-64	100%	65.87%	*Acremonium chrysogenum ATCC 11550*	KFH43814.1
flavin depend monooxygenase that catalyses the oxidation of rubrofusarin to 9-hydroxyrubrofusarin	523	0	100%	77.71%	*Fusarium albosuccineum*	KAF4463211.1
xylosidase/arabinosidase	625	0	95%	89.98%	*Plectosphaerella plurivora*	KAH6670864.1

hypothetical protein DL764_000444	972	0	98%	77.80%	*Monosporascus ibericus*	RYP10822.1
hypothetical protein HYQ44_009310	169	3.00E-22	83%	57.82%	*Verticillium longisporum*	KAG7111115.1
Dolichyl-diphosphooligosaccharide--protein glycosyltransferase subunit SWP1	287	2.00E-92	99%	58.54%	*Colletotrichum sidae*	TEA10577.1
glutaminyl-peptide cyclotransferase	388	0	100%	72.63%	*Sodiomyces alkalinus F11*	XP_028462781.1
putative oxidoreductase YusZ	284	5.00E-131	99%	65.25%	*Colletotrichum spaethianum*	GJC88340.1
CDP-alcohol phosphatidyltransferase	427	0	92%	82.62%	*Sodiomyces alkalinus F11*	XP_028462783.1

35) *Verticillium isaacii* contig_ NMXN01000005, VRPKS-I-23 cluster

Gene name	Size	E value	% Coverage	% identity	Top blast hit	Accession number of top blast hit
hypothetical protein CH063_11708	26	3.20E-02	100%	73.33%	*Colletotrichum higginsianum*	CCF41433.1
Ribokinase-like protein	365	8.00E-65	98%	38.11%	*Sodiomyces alkalinus F11*	XP_028463289.1
homoserine kinase	356	0	100%	90.17%	*Plectosphaerella cucumerina*	KAH7362595.1
SnodProt1	138	1.00E-65	100%	72.46%	*Plectosphaerella cucumerina*	KAH7368131.1
hypothetical protein B0T11DRAFT_355575	247	4.00E-07	36%	45.45%	*Plectosphaerella cucumerina*	KAH7358954.1
hypothetical protein B0T11DRAFT_306916	423	3.00E-101	91%	62.18%	*Plectosphaerella cucumerina*	KAH7358952.1
uncharacterized protein CGCS363_v012205	365	2.00E-104	93%	51.50%	*Colletotrichum siamense*	XP_036491211.1
aromatic prenyltransferase	456	0.00E+00	100%	68.49%	*Plectosphaerella cucumerina*	KAH7358950.1
phenolpthiocerol synthesis polyketide synthase ppsA	2347	0	99%	51.79%	*Sodiomyces alkalinus F11*	XP_028462492.1
drug resistance transporter	588	0.00E+00	99%	70.31%	*Plectosphaerella plurivora*	KAH6697430.1
Hybrid signal transduction histidine kinase K like protein	1386	0	99%	66.50%	*Plectosphaerella cucumerina*	KAH7358170.1
hypothetical protein B0T11DRAFT_299362	220	5.00E-62	51%	82.30%	*Plectosphaerella cucumerina*	KAH7358281.1
hypothetical protein B0T11DRAFT_284115	230	1.00E-96	100%	75.65%	*Plectosphaerella cucumerina*	KAH7358280.1
uncharacterized protein CORC01_08067	323	3.00E-168	100%	86.69%	*Colletotrichum orchidophilum*	XP_022473766.1
SRP54-domain-containing protein	689	0.00E+00	100%	78.59%	*Sodiomyces alkalinus F11*	XP_028465413.1
myosin class II heavy chain	1497	0	97%	62.47%	*Sodiomyces alkalinus F11*	XP_028465411.1

36) *Verticillium isaacii* contig_ NMXN01000012, VRPKS-I-26 cluster

Gene name	Size	E value	% Coverage	% identity	Top blast hit	Accession number of top blast hit
hypothetical protein F5X68DRAFT_228683	72	4.00E-04	31%	86.96%	*Plectosphaerella plurivora*	KAH6692307.1
peptidase family C25-domain-containing protein	1017	0	99%	57.20%	*Paraphoma chrysanthemicola*	KAH7091334.1

hypothetical protein	578	0.00E+00	96%	88.33%	*Colletotrichum higginsianum IMI 349063*	XP_018157666.1
Satratoxin biosynthesis SC1 cluster protein 4	758	2.00E-137	69%	58.71%	*Exophiala spinifera*	XP_016240708.1
putative pectate lyase F	236	1.00E-140	97%	83.98%	*Colletotrichum gloeosporioides*	XP_045257419.1
hypothetical protein	1004	0.00E+00	100%	72.87%	*Plectosphaerella cucumerina*	KAH7362743.1
hypothetical protein B0T11DRAFT_318355	163	4.00E-55	98%	65.85%	*Plectosphaerella cucumerina*	KAH7362742.1
uncharacterized protein KVR01_004169	257	6.00E-06	22%	45.61%	*Diaporthe batatas*	XP_044646333.1
putative polyketide synthase	2349	0	99%	52.38%	*Whalleya microplaca*	KAI1073750.1
TTL domain-containing protein	473	0	100%	73.65%	*Coniochaeta sp. 2T2.1*	KAB5518934.1
retinol dehydrogenase	315	0.00E+00	99%	79.17%	*Plectosphaerella cucumerina*	KAH7369374.1
leucyl aminopeptidase	372	0.00E+00	100%	79.03%	*Dactylonectria estremocensis*	KAH7144232.1
heterokaryon incompatibility protein-domain-containing protein	742	0.00E+00	100%	75.61%	*Chaetomium sp. MPI-CAGE-AT-0009*	KAH6847694.1
FAD-dependent monooxygenase	453	0	99%	86.89%	*Stachybotrys elegans*	KAH7303249.1
glycosyl transferase	427	1.00E-137	77%	58.56%	*Trichoderma arundinaceum*	RFU78228.1
alpha-l-rhamnosidase	898	0	92%	69.63%	*Colletotrichum truncatum*	XP_036586343.1
hypothetical protein SODALDRAFT_319240	134	5.00E-25	95%	39.39%	*Sodiomyces alkalinus F11*	XP_028470409.1

37) *Verticillium isaacii* contig_ NMXN01000007, VRPKS-I-19 cluster

**Gene name**	**Size**	**E value**	**% Coverage**	**% identity**	**Top blast hit**	**Accession number of top blast hit**
alpha glucoside transporter	542	0	99%	75.41%	*Cylindrodendrum hubeiense*	KAF7546071.1
hypothetical protein SODALDRAFT_333514	77	2.00E-21	97%	55.13%	*Sodiomyces alkalinus F11*	XP_028465568.1
hypothetical protein ACRE_064810	279	4.00E-82	98%	52.31%	*Acremonium chrysogenum ATCC 11550*	KFH42767.1
hypothetical protein B0J18DRAFT_6692	109	2.00E-28	78%	63.22%	*Chaetomium sp. MPI-SDFR-AT-0129*	KAH6634037.1
Mitochondrial import inner membrane translocase subunit tim44 like protein	541	0	100%	75.69%	*Sodiomyces alkalinus F11*	XP_028468965.1
hypothetical protein CFIO01_06157	250	3.00E-91	100%	56.00%	*Colletotrichum fioriniae PJ7*	EXF75199.1
DeSI-like protein sdu1	239	2.00E-102	76%	82.07%	*Colletotrichum orbiculare MAFF 240422*	TDZ21862.1
Obg-like ATPase 1	394	0	100%	90.61%	*Pyricularia pennisetigena*	XP_029751038.1
exopolygalacturonase B like protein	469	0.00E+00	94%	72.26%	*Monosporascus sp. mg162*	RYP54678.1
hypothetical protein AK830_g4742	499	4.00E-75	82%	39.64%	*Neonectria ditissima*	KPM41855.1
thioredoxin-like protein	162	3.00E-85	98%	76.25%	*Hypoxylon sp. FL0890*	KAI0834638.1
MFS general substrate transporter	578	0	97%	70.86%	*Sodiomyces alkalinus F11*	XP_028468956.1

hypothetical protein J1614_005793	297	5.00E-110	89%	63.53%	*Leptosphaeria biglobosa*	KAH9873395.1
Type I Polyketide synthase	2177	0.00E+00	99%	77.43%	Leptosphaeria biglobosa	KAH9873394.1
L-threonate dehydrogenase like protein	1108	0.00E+00	99%	71.90%	*Colletotrichum scovillei*	XP_035328982.1
hypothetical protein F5X68DRAFT_50483	688	0.00E+00	95%	68.99%	*Plectosphaerella plurivora*	KAH6669659.1
major facilitator superfamily transporter	488	0	98%	79.42%	*Colletotrichum incanum*	OHW90295.1
cutinase-2	224	7.00E-132	92%	87.92%	*Plectosphaerella cucumerina*	KAH7368196.1
putative beta-glucosidase G	832	0	99%	68.77%	*Aspergillus sydowii CBS 593.65*	XP_040708017.1
vacuolar protein sorting-associated protein	483	0	96%	74.15%	*Plectosphaerella cucumerina*	KAH7368195.1

38) *Verticillium isaacii* contig_ NMXN01000010, VRPKS-I-18 cluster

**Gene name**	**Size**	**E value**	**% Coverage**	**% identity**	**Top blast hit**	**Accession number of top blast hit**
Sec7 domain-containing protein	1558	0	99%	64.12%	*Plectosphaerella plurivora*	KAH6686645.1
glycosyl hydrolase family 16 protein	425	1.00E-135	64%	71.17%	*Colletotrichum truncatum*	XP_036580125.1
secreted protein	211	2.00E-93	90%	76.96%	*Sodiomyces alkalinus F11*	XP_028465800.1
hypothetical protein ZTR_07120	526	0	98%	86.46%	*Talaromyces verruculosus*	KUL84026.1
general substrate transporter	518	0.00E+00	98%	68.95%	*Dactylonectria macrodidyma*	KAH7160676.1
calcineurin-like phosphoesterase	363	0.00E+00	100%	72.45%	*Plectosphaerella plurivora*	KAH6686639.1
serine hydrolase FSH	253	3.00E-134	99%	76.98%	*Fulvia fulva*	UJO18001.1
AMP-dependent synthetase/ligase	611	5.00E-161	99%	44.19%	*Hirsutella minnesotensis 3608*	KJZ72768.1
Reducing polyketide synthase PKS2	2256	0	99%	75.10%	*Fulvia fulva*	UJO18003.1
hypothetical protein BDW02DRAFT_369921	235	7.00E-55	100%	47.46%	*Decorospora gaudefroyi*	KAF1833738.1
carbohydrate-binding module family 50 protein	686	0.00E+00	99%	58.67%	*Plectosphaerella plurivora*	KAH6684917.1
putative chitinase	539	0	96%	80.73%	*Decorospora gaudefroyi*	KAF1833741.1
hypothetical protein P154DRAFT_517162	391	3.00E-119	100%	49.51%	*Amniculicola lignicola CBS 123094*	KAF2007437.1
SGNH hydrolase	711	0.00E+00	100%	74.02%	*Macroventuria anomochaeta*	XP_033567680.1
glycoside hydrolase superfamily	751	0	98%	67.43%	*Plectosphaerella plurivora*	KAH6658770.1
hypothetical protein F5X68DRAFT_145888	1025	0.00E+00	98%	55.58%	*Plectosphaerella plurivora*	KAH6658772.1
putative UDP-glucose-4-epimerase	254	2.00E-127	98%	71.83%	*Plectosphaerella cucumerina*	KAH7366775.1
MFS general substrate transporter	471	6.00E-155	97%	51.52%	*Oidiodendron maius Zn*	KIN03209.1

39) *Verticillium isaacii* contig_ NMXN01000005, VRPKS-I-17 cluster

Gene name	Size	E value	% Coverage	% identity	Top blast hit	Accession number of top blast hit
splicing factor 3b subunit 4	370	1.00E-153	85%	80.82%	Colletotrichum camelliae	KAH0444958.1
Mitochondrial FAD carrier protein FLX1	310	3.00E-157	99%	74.30%	Colletotrichum chlorophyti	OLN84903.1
Asparagine-rich zinc finger protein AZF1 like	439	3.00E-122	88%	50.25%	Colletotrichum fioriniae PJ7	EXF85239.1
Urm1-domain-containing protein	104	7.00E-56	96%	79.00%	Xylaria curta	KAI0546841.1
elongator protein 2 (WD domain-containing protein)	841	0	99%	67.50%	Colletotrichum truncatum	XP_036582858.1
Reducing polyketide synthase PKS2 like protein	113	9.00E-46	83%	75.53%	Plectosphaerella cucumerina	KAH7363092.1
Lovastatin diketide synthase LovF 16	2277	0	99%	71.71%	Colletotrichum chlorophyti	OLN86478.1
duf341 family	255	2.00E-147	100%	79.69%	Colletotrichum sojae	KAF6806434.1
ABC transporter	1284	0	97%	70.95%	Colletotrichum scovillei	KAG7055337.1
Actin	375	0.00E+00	100%	100.00%	Tolypocladium paradoxum	POR36442.1
hsp70-like protein	209	3.00E-96	97%	71.08%	Sodiomyces alkalinus F11	XP_028466987.1
54S ribosomal protein L7	356	0	98%	76.42%	Sodiomyces alkalinus F11	XP_028466988.1
ubiquitin C-terminal hydrolase	800	0	100%	63.79%	Plectosphaerella cucumerina	KAH7363099.1
6-phosphofructo-2-kinase	570	0	100%	84.78%	Plectosphaerella cucumerina	KAH7363100.1
mitochondrial import receptor subunit tom-40	352	0.00E+00	100%	83.33%	Sodiomyces alkalinus F11	XP_028466992.1
Isoflavone reductase	307	3.00E-69	97%	48.84%	Colletotrichum higginsianum IMI 349063	XP_018160455.1

40) *Verticillium isaacii* contig_ NMXN01000010, VRPKS-I-13 cluster

Gene name	Size	E value	% Coverage	% identity	Top blast hit	Accession number of top blast hit
major facilitator superfamily domain-containing protein	130	5.00E-28	99%	40.00%	*Xylariales sp. PMI_506*	KAH8666575.1
uncharacterized protein CHGG_08617	449	5.00E-170	92%	57.24%	*Chaetomium globosum CBS 148.51*	XP_001226544.1
thiol-specific monooxygenase	507	0.00E+00	99%	76.54%	*Plectosphaerella plurivora*	KAH6686480.1
glycogen debranching enzyme	665	0.00E+00	98%	76.97%	*Dactylonectria macrodidyma*	KAH7115576.1
major facilitator superfamily domain-containing protein	583	0.00E+00	100%	85.08%	*Plectosphaerella cucumerina*	KAH7368056.1
endoglucanase-1	233	2.00E-133	98%	78.60%	*Plectosphaerella cucumerina*	KAH7362705.1
hypothetical protein SODALDRAFT_4980	221	3.00E-60	81%	60.22%	*Sodiomyces alkalinus F11*	XP_028469806.1
epoxide hydrolase	409	0.00E+00	98%	62.69%	*Plectosphaerella plurivora*	KAH6662678.1
endo-1,4-beta-glucanase	296	3.00E-93	81%	60.00%	*Colletotrichum sojae*	KAF6788143.1
hypothetical protein BT63DRAFT_423135	236	4.00E-38	85%	73.63%	*Microthyrium microscopicum*	KAF2670841.1

polyketide synthase	3080	0.00E+00	99%	53.42%	*Fusarium avenaceum*	KIL85244.1
hypothetical protein FALBO_5956	314	0.00E+00	100%	85.67%	*Fusarium albosuccineum*	KAF4467173.1
Uracil permease like protein	527	0	98%	73.43%	*Colletotrichum camelliae*	KAH0420306.1
2OG-Fe oxygenase family protein	332	2.00E-158	99%	70.69%	*Colletotrichum incanum*	OHW94343.1
c6 transcription factor	585	0	99%	67.67%	*Plectosphaerella cucumerina*	KAH7362695.1
hypothetical protein B0T11DRAFT_334166	256	2.00E-63	88%	48.02%	*Plectosphaerella cucumerina*	KAH7374617.1

41) *Verticillium isaacii* contig_ NMXN01000006, VRPKS-I-20 cluster

Gene name	Size	E value	% Coverage	% identity	Top blast hit	Accession number of top blast hit
SUMO-activating enzyme subunit uba-2	456	0	94%	74.54%	*Sodiomyces alkalinus F11*	XP_028470156.1
gluconate 5-dehydrogenase	271	1.00E-105	99%	71.08%	*Sodiomyces alkalinus F11*	XP_028470157.1
feruloyl esterase b	315	8.00E-176	93%	79.52%	*Colletotrichum musicola*	KAF6811137.1
Dihydrosphingosine 1-phosphate phosphatase	554	0.00E+00	94%	73.57%	*Colletotrichum asianum*	KAF0325175.1
DNA damage-responsive transcriptional repressor RPH1	1425	0	77%	66.19%	*Colletotrichum fioriniae PJ7*	EXF81018.1
mannan endo-1,4-beta-mannosidase	401	0.00E+00	95%	82.81%	*Plectosphaerella plurivora*	KAH6695781.1
polyketide synthase	3430	0.00E+00	70%	47.07%	*Ophiocordyceps camponoti-floridani*	KAF4594413.1
Alpha-ketoglutarate-dependent 2,4-dichlorophenoxyacetate dioxygenase	343	0.00E+00	98%	70.71%	*Penicillium arizonense*	XP_022486531.1
serine hydrolase FSH	283	8.00E-91	100%	46.64%	*Cordyceps sp. RAO-2017*	PHH90555.1
Cytochrome P450 4V2 [	542	0	100%	65.83%	*Penicillium arizonense*	XP_022486670.1
Chloramphenicol acetyltransferase-like domain protein [Metarhizium album ARSEF 1941	527	0	96%	55.83%	*Akanthomyces lecanii RCEF 1005*	OAA79799.1
hypothetical protein FALBO_6676	100	2.00E-19	93%	49.46%	*Fusarium albosuccineum*	KAF4466462.1
glutamyl-tRNA amidotransferase subunit A	936	0	65%	70.26%	*Plectosphaerella cucumerina*	KAH7374727.1
hypothetical protein B0J13DRAFT_652468	254	4.00E-135	100%	74.41%	*Dactylonectria estremocensis*	KAH7118096.1
NAD-binding Rossmann fold oxidoreductase family protein	354	0.00E+00	97%	73.84%	*Talaromyces rugulosus*	XP_035348268.1
hypothetical protein EDB80DRAFT_562441	1105	0.00E+00	62%	52.92%	*Ilyonectria destructans*	KAH7018764.1

42) *Verticillium isaacii* contig_ NMXN01000012, PKS-III cluster

Gene name	Size	E value	% Coverage	% identity	Top blast hit	Accession number of top blast hit

allantoate permease	503	0	100%	84.68%	*Plectosphaerella cucumerina*	KAH7375308.1
alpha-ketoglutarate-dependent sulfonate dioxygenase	378	0	100%	84.13%	*Plectosphaerella plurivora*	KAH6681182.1
hypothetical protein B0T11DRAFT_143288	221	1.00E-85	88%	61.73%	*Plectosphaerella cucumerina*	KAH7347634.1
hypothetical protein CPLU01_05979	289	9.00E-54	64%	48.44%	*Colletotrichum plurivorum*	KAF6832745.1
carbonic anhydrase	271	4.00E-146	93%	76.77%	*Colletotrichum incanum*	KZL87566.1
Acetyltransferase	270	2.00E-149	87%	83.97%	*Colletotrichum higginsianum IMI 349063*	XP_018159255.1
oxidoreductase	452	0	99%	64.67%	*Plectosphaerella plurivora*	KAH6681205.1
Feruloyl esterase B	294	1.00E-150	91%	75.84%	*Colletotrichum orbiculare MAFF 240422*	TDZ25527.1
transcriptional activator protein acu-15	709	0	98%	73.83%	*Colletotrichum spaethianum*	GJC81216.1
TRAUB-domain-containing protein	594	4.00E-132	78%	74.50%	*Plectosphaerella cucumerina*	KAH7375290.1
hypothetical protein K456DRAFT_1765144	127	7.00E-23	86%	58.93%	*Colletotrichum gloeosporioides 23*	KAH9228351.1
putative chalcone and stilbene synthase domain-containing protein	444	0	100%	75.73%	*Colletotrichum sublineola*	KDN65911.1
fructose-bisphosphate aldolase	362	0	100%	90.88%	*Plectosphaerella cucumerina*	KAH7375300.1
PAB-dependent poly(A)-specific ribonuclease subunit PAN2	1082	0	98%	79.04%	*Plectosphaerella cucumerina*	KAH7375292.1
uncharacterized protein D7B24_007593	608	0	89%	68.93%	*Verticillium nonalfalfae*	XP_028494368.1
putative mitochondrial 2-oxoglutarate/malate carrier protein	335	0	100%	86.87%	*Colletotrichum chlorophyti*	OLN95994.1
hypothetical protein B0T11DRAFT_249550	637	2.00E-173	96%	60.57%	*Plectosphaerella cucumerina*	KAH7375296.1
Splicing factor spf30	304	3.00E-113	99%	68.83%	*Colletotrichum truncatum*	XP_036586172.1
Iron-sulfur protein IND1	296	7.00E-177	99%	79.59%	*Colletotrichum truncatum*	XP_036586173.1
centrin-3	189	2.00E-88	98%	68.09%	*Sodiomyces alkalinus F11*	XP_028470317.1
short chain dehydrogenase	145	4.00E-75	100%	83.45%	*Sodiomyces alkalinus F11*	XP_028470310.1

43) *Verticillium isaacii* contig_ NMXN01000003, VRPKS-I-14 cluster

**Gene name**	**Size**	**E value**	**% Coverage**	**% identity**	**Top blast hit**	**Accession number of top blast hit**
hypothetical protein F4819DRAFT_459179	1030	0	95%	41.14%	*Hypoxylon fuscum*	KAI1401228.1
Cytochrome P450 52A13	519	0	97%	67.67%	*Colletotrichum gloeosporioides*	XP_045269785.1
hypothetical protein CGLO_06291	624	6.00E-155	92%	41.51%	*Colletotrichum gloeosporioides Cg-14*	EQB53932.1
Acyl-CoA dehydrogenase/oxidase	596	0	92%	52.44%	*Cordyceps javanica*	TQV95615.1

fatty acid desaturase domain-containing protein	608	0	100%	63.74%	*Sarocladium implicatum*	KAH8177350.1
putative polyketide synthase	3079	0	100%	56.21%	*Truncatella angustata*	KAH8198320.1
putative major facilitator superfamily transporter	584	0	83%	62.30%	*Truncatella angustata*	XP_045957260.1
cytochrome P450	506	0	100%	79.05%	*Plectosphaerella cucumerina*	KAH7367666.1
hypothetical protein CEP52_003390	374	6.00E-159	97%	69.86%	*Fusarium sp. AF-4*	RSM10820.1
short-chain dehydrogenase/reductase	310	1.00E-109	94%	50.93%	*Aspergillus flavipes*	A0A7L8UXK7.1
hypothetical protein B0T11DRAFT_56928	204	7.00E-107	100%	71.98%	*Plectosphaerella cucumerina*	KAH7367670.1
Vanillyl-alcohol oxidase	562	0	99%	87.12%	*Plectosphaerella plurivora*	KAH6673908.1
Protein rds1 like protein	497	0	92%	79.26%	*Sodiomyces alkalinus F11*	XP_028471380.1
FK506-binding protein 2	191	2.00E-82	100%	70.68%	*Colletotrichum orbiculare MAFF 240422*	TDZ17824.1
hypothetical protein HYQ45_016732	88	5.00E-04	100%	71.43%	*Verticillium longisporum*	KAG7113540.1
hypothetical protein FALBO_210	144	2.00E-69	98%	73.24%	*Fusarium albosuccineum*	KAF4472888.1
hypothetical protein F5883DRAFT_436293	1088	0	100%	75.09%	*Diaporthaceae sp. PMI_573*	KAH8748597.1

44) *Verticillium isaacii* contig_ NMXN01000002, VRPKS-I-25 cluster

**Gene name**	**Size**	**E value**	**% Coverage**	**% identity**	**Top blast hit**	**Accession number of top blast hit**
RING-3 protein	1475	0	43%	69.46%	*Plectosphaerella plurivora*	KAH6680056.1
glucosidase II beta subunit-like protein-domain-containing protein	524	0	95%	63.23%	*Plectosphaerella plurivora*	KAH6680057.1
GNAT family n-acetyltransferase	187	4.00E-90	99%	64.17%	*Clonostachys solani*	CAH0054579.1
key lime pathogenicity protein	673	6.00E-146	86%	51.24%	*Colletotrichum sublineola*	KDN66949.1
hypothetical protein SODALDRAFT_165672	374	3.00E-44	90%	42.36%	*Sodiomyces alkalinus F11*	XP_028466392.1
S-adenosyl-L-methionine-dependent methyltransferase	313	6.00E-156	92%	81.10%	*Sodiomyces alkalinus F11*	XP_028466391.1
dihydroxy-acid dehydratase	651	0	99%	85.23%	*Plectosphaerella plurivora*	KAH6671001.1
NAD(P)-binding protein	363	0	100%	85.00%	*Stachybotrys chartarum IBT 7711*	KEY68920.1
short chain dehydrogenase	340	0	92%	85.99%	*Stachybotrys chartarum IBT 7711*	KEY68921.1
polyketide synthase	2356	0	99%	89.64%	*Stachybotrys chartarum IBT 40293*	KFA46917.1
beta-xylosidase (glycosyl hydrolase)	603	0	95%	85.07%	*Colletotrichum truncatum*	XP_036575294.1
hypothetical protein ColLi_06807	527	0	87%	59.10%	*Colletotrichum liriopes*	GKT46626.1
Thiol-specific monooxygenase	492	0	98%	67.56%	*Colletotrichum fructicola*	XP_031891081.1

Protein STB5-like protein 3	765	0	88%	61.73%	*Colletotrichum chlorophyti*	OLN81943.1
Sodium transport ATPase 5 like protein	1071	0	98%	74.30%	*Monosporascus sp. MG133*	RYP34097.1
bifunctional polynucleotide phosphatase/kinase	453	0	99%	67.69%	*Sodiomyces alkalinus F11*	XP_028468241.1
mediator complex, subunit Med18	269	2.00E-164	99%	80.07%	*Plectosphaerella cucumerina*	KAH7347312.1
C6 transcription factor	326	1.00E-116	90%	62.75%	*Plectosphaerella cucumerina*	KAH7366962.1

45) *Verticillium isaacii* contig_ NMXN01000008, VRPKS-I-2 cluster

Gene name	Size	E value	% Coverage	% identity	Top blast hit	Accession number of top blast hit
HpcH/HpaI aldolase	225	4.00E-149	100%	96.89%	*Plectosphaerella cucumerina*	KAH7362559.1
sugar transporter domain-containing protein	547	0	99%	87.36%	*Plectosphaerella cucumerina*	KAH7362558.1
major facilitator superfamily transporter	496	0	100%	85.92%	*Plectosphaerella cucumerina*	KAH7362562.1
hypothetical protein B0T11DRAFT_233715	493	0	93%	71.71%	*Plectosphaerella cucumerina*	KAH7374470.1
O-methyltransferase	235	1.00E-110	100%	69.36%	*Plectosphaerella plurivora*	KAH6687359.1
putative 1-aminocyclopropane-1-carboxylate synthase	416	0	97%	72.24%	*Plectosphaerella plurivora*	KAH6687356.1
major facilitator superfamily domain-containing protein	996	0	57%	82.11%	*Plectosphaerella plurivora*	KAH6687355.1
alcohol dehydrogenase-like protein	353	0	99%	90.06%	*Colletotrichum fructicola*	XP_031875992.1
Polyketide synthase-nonribosomal peptide synthetase	435	0	100%	87.82%	*Colletotrichum fructicola*	XP_031875984.1
polyketide synthetase	3917	0	100%	85.82%	*Colletotrichum fructicola*	KAF4886526.1
Alpha/beta hydrolase	433	0	99%	84.22%	*Colletotrichum fructicola*	XP_031875995.1
Major Facilitator Superfamily protein	584	0	99%	84.96%	*Colletotrichum fructicola*	KAF4886521.1
uncharacterized protein CGMCC3_g17405	193	5.00E-125	100%	87.56%	*Colletotrichum fructicola*	XP_031875990.1
D-isomer specific 2-hydroxyacid dehydrogenase	326	0	99%	78.59%	*Plectosphaerella plurivora*	KAH6689062.1
PPR1-transcription factor regulating pyrimidine pathway	466	2.00E-111	99%	55.52%	*Plectosphaerella plurivora*	KAH6689063.1
smr domain-containing protein	730	0	100%	84.17%	*Colletotrichum graminicola M1.001*	XP_008090428.1

46) *Verticillium isaacii* contig_ NMXN01000005, VRPKS-I-12 and VNRPKS-I-1 clusters

Gene name	Size	E value	% Coverage	% identity	Top blast hit	Accession number of top blast hit
hypothetical protein FPOAC1_009761	238	3.00E-82	89%	59.72%	*Fusarium poae*	XP_044706852.1
cytochrome P450	476	0	97%	74.57%	*Sodiomyces alkalinus F11*	XP_028469280.1

beta-ketoacyl synthase	366	8.00E-89	99%	46.51%	*Thelonectria olida*	KAH6899712.1
eukaryotic aspartyl protease	432	0	96%	56.73%	*Colletotrichum simmondsii*	KXH41725.1
FAD/NAD(P)-binding domain-containing protein	419	0	93%	72.11%	*Sodiomyces alkalinus F11*	XP_028469276.1
hypothetical protein SODALDRAFT_331204	890	0	86%	70.91%	*Sodiomyces alkalinus F11*	XP_028469275.1
polyketide synthase	3948	0	97%	75.30%	*Sodiomyces alkalinus F11*	XP_028469272.1
L-2,4-diaminobutyrate decarboxylase	514	0	88%	64.41%	*Colletotrichum trifolii*	TDZ67276.1
duf92 domain protein	388	5.00E-130	99%	62.37%	*Sodiomyces alkalinus F11*	XP_028469270.1
uncharacterized protein CTRU02_08118	316	0.016	61%	24.77%	*Colletotrichum truncatum*	XP_036582041.1
multicopper oxidase	561	0	98%	67.88%	*Plectosphaerella cucumerina*	KAH7358155.1
Conidial pigment polyketide synthase	2188	0	100%	78.78%	*Colletotrichum gloeosporioides Cg-14*	EQB55056.1
oxidoreductase	325	0	99%	84.88%	*Colletotrichum truncatum*	XP_036586400.1
transcription factor	424	2.00E-148	100%	56.88%	*Colletotrichum incanum*	KZL86702.1
transcription factor Cmr1	894	0	100%	72.43%	*Colletotrichum musicola*	KAF6845250.1

47) *Verticillium isaacii* contig_ NMXN01000008, VRPKS-I-6 cluster

Gene name	Size	E value	% Coverage	% identity	Top blast hit	Accession number of top blast hit
cytochrome P450	462	0	99%	65.81%	*Aspergillus avenaceus*	KAE8150739.1
putative alcohol dehydrogenase	370	0	100%	79.73%	*Xylariaceae sp. FL0016*	KAI1344559.1
cytochrome P450	529	0	93%	67.87%	*Aspergillus alliaceus*	XP_031905229.1
solid-state culture-specific protein-like protein	466	0	99%	63.42%	*Didymosphaeria enalia*	KAF2268085.1
ABC transporter	1166	0	90%	58.76%	*Clohesyomyces aquaticus*	ORY13587.1
solid-state culture-specific ATP-grasp domain protein	470	0	100%	65.32%	*Didymosphaeria enalia*	KAF2268082.1
putative hybrid NRPS/PKS enzyme	3987	0	99%	57.65%	*Xylariomycetidae sp. FL2044*	KAH9905503.1
serine hydrolase-domain-containing protein	284	1.00E-131	100%	63.03%	*Xylariaceae sp. FL0016*	KAI1344560.1
Dihydrolipoyllysine-residue acetyltransferase component of acetoin cleaving system	215	2.00E-79	94%	50.78%	*Colletotrichum siamense*	XP_036493445.1
thioredoxin	332	4.00E-172	100%	72.89%	*Plectosphaerella plurivora*	KAH6691560.1
putative polyol transporter 2	599	0	99%	82.58%	*Ilyonectria sp. MPI-CAGE-AT-0026*	KAH6967656.1
Transaldolase	365	0	100%	87.16%	*Colletotrichum siamense*	KAF4850344.1
hypothetical protein B0T10DRAFT_76705	250	1.00E-48	73%	51.49%	*Thelonectria olida*	KAH6887013.1
hypothetical protein C8035_v000460	325	1.00E-129	97%	63.66%	*Colletotrichum spinosum*	TDZ12713.1

hypothetical protein CH35J_006353	643	2.00E-127	80%	46.34%	*Colletotrichum higginsianum*	TIC99959.1
MFS sugar transporter	489	0	97%	74.11%	*Colletotrichum sojae*	KAF6797496.1
protein PXR1	366	2.00E-75	50%	82.64%	*Plectosphaerella cucumerina*	KAH7368324.1
hypothetical protein HYQ45_010318	159	1.00E-45	78%	64.00%	*Verticillium longisporum*	KAG7131019.1

48) *Verticillium isaacii* contig_ NMXN01000006, VRPKS-I-5 cluster

Gene name	Size	E value	% Coverage	% identity	Top blast hit	Accession number of top blast hit
hypothetical protein jhhlp_008516	710	0	99%	73.50%	*Lomentospora prolificans*	PKS05149.1
Isochorismatase-like protein	244	6.00E-126	90%	76.13%	*Plectosphaerella plurivora*	KAH6676014.1
hypothetical protein B0T11DRAFT_333834	160	4.00E-32	63%	64.71%	*Plectosphaerella cucumerina*	KAH7347765.1
DUF636 domain-containing protein	137	9.00E-68	100%	83.94%	*Purpureocillium lilacinum*	XP_018184317.1
putative PKS-NRPS protein	3934	0	99%	65.20%	*Biscogniauxia sp. FL1348*	KAI0597589.1
hypothetical protein B0I37DRAFT_378309	507	4.00E-63	62%	57.67%	*Chaetomium sp. MPI-CAGE-AT-0009*	KAH6844803.1
hypothetical protein BGAL_0184g00240	226	4.00E-71	84%	57.81%	*Botrytis galanthina*	THV49682.1
major facilitator superfamily-domain-containing protein	612	0	89%	72.68%	*Biscogniauxia sp. FL1348*	KAI0597572.1
glutathione S-transferase domain-containing protein	224	1.00E-124	99%	78.92%	*fungal sp. No.14919*	GAW23379.1
hypothetical protein SODALDRAFT_41197	1568	0	98%	67.49%	*Sodiomyces alkalinus F11*	XP_028471323.1
GPI-GlcNAc transferase complex	222	2.00E-38	97%	63.13%	*Plectosphaerella cucumerina*	KAH7347756.1
pseudouridine synthase	602	0	97%	52.59%	*Sodiomyces alkalinus F11*	XP_028471325.1
GTP-binding protein RHO2	187	3.00E-108	100%	79.68%	*Colletotrichum tanaceti*	TKW53379.1
DNA-directed RNA polymerase III subunit RPC9	119	2.00E-38	75%	67.78%	*Sodiomyces alkalinus F11*	XP_028471326.1

49) *Verticillium isaacii* contig_ NMXN01000009, VRPKS-I-9 cluster

Gene name	Size	E value	% Coverage	% identity	Top blast hit	Accession number of top blast hit
hypothetical protein S40285_06408	391	9.00E-110	92%	55.12%	*Stachybotrys chlorohalonata IBT 40285*	KFA62410.1
FAD dependent oxidoreductase	440	0	97%	77.52%	*Plectosphaerella cucumerina*	KAH7368673.1
major facilitator superfamily domain-containing protein	538	0	99%	83.27%	*Plectosphaerella cucumerina*	KAH7368674.1
uncharacterized protein B0I36DRAFT_355828	178	1.00E-10	37%	78.79%	*Microdochium trichocladiopsis*	XP_046005612.1
acetamidase	558	0	99%	77.74%	*Plectosphaerella plurivora*	KAH6684967.1
polyketide synthase 3	4064	0	99%	72.13%	*Hirsutella rhossiliensis*	XP_044717051.1

hypothetical protein CEP52_015090	549	8.00E-48	44%	41.06%	*Fusarium sp. AF-4*	RSL88831.1
lipase	560	0	96%	68.96%	*Plectosphaerella cucumerina*	KAH7376670.1
polysaccharide deacetylase	518	5.00E-156	69%	78.33%	*Plectosphaerella cucumerina*	KAH7374645.1
hypothetical protein B0T11DRAFT_318679	477	1.00E-108	45%	72.81%	*Plectosphaerella cucumerina*	KAH7363183.1
hypothetical protein ISF_06027	85	8.00E-22	75%	57.81%	*Cordyceps fumosorosea ARSEF 2679*	XP_018703129.1
tetratricopeptide repeat protein 1	285	2.00E-108	89%	79.22%	*Colletotrichum karsti*	XP_038750970.1
HAD-like protein	270	3.00E-135	99%	71.32%	*Stachybotrys chartarum IBT 40288*	KFA78825.1
C6 zinc finger domain-containing protein	701	0	100%	66.53%	*Colletotrichum incanum*	OHX01157.1

50) *Verticillium klebahnii* contig_ NMXL01000003, VRPKS-I-16 cluster

**Gene name**	**Size**	**E value**	**% Coverage**	**% identity**	**Top blast hit**	**Accession number of top blast hit**
choline/ethanolaminephosphotransferase	418	0.00E+00	94%	82.74%	*Sodiomyces alkalinus F11*	XP_028462783.1
estradiol 17-beta-dehydrogenase (short-chain dehydrogenase)	284	2.00E-126	99%	63.83%	*Colletotrichum tofieldiae*	KZL75089.1
glutaminyl-peptide cyclotransferase	388	0.00E+00	100%	72.63%	*Sodiomyces alkalinus F11*	XP_028462781.1
Dolichyl-diphosphooligosaccharide--protein glycosyltransferase subunit SWP1	287	3.00E-92	99%	58.19%	*Colletotrichum sidae*	TEA10577.1
hypothetical protein HYQ44_009310	169	3.00E-24	87%	57.24%	*Verticillium longisporum*	KAG7111115.1
putative copper-transporting ATPase	1069	0.00E+00	100%	80.50%	*Monosporascus ibericus*	RYP10822.1
xylosidase/arabinosidase	599	0.00E+00	100%	90.48%	*Plectosphaerella plurivora*	KAH6670864.1
flavin depend monooxygenase that catalyses the oxidation of rubrofusarin to 9-hydroxyrubrofusarin	524	0.00E+00	100%	78.86%	*Fusarium albosuccineum*	KAF4463211.1
hypothetical protein ACRE_054030	167	7.00E-64	100%	65.87%	*Acremonium chrysogenum ATCC 11550*	KFH43814.1
Alpha/Beta hydrolase protein	557	0	94%	66.10%	*Plectosphaerella plurivora*	KAH6688446.1
putative polyketide synthase	2603	0	99%	72.37%	*Plectosphaerella cucumerina*	KAH7369281.1
serine hydrolase FSH	239	1.00E-78	100%	58.33%	*Plectosphaerella plurivora*	KAH6673998.1
glycosyl hydrolase	656	0	94%	63.12%	*Colletotrichum gloeosporioides*	XP_045266644.1
Spindle pole body protein ppc89	1066	0	100%	59.44%	*Plectosphaerella cucumerina*	KAH7375266.1
putative RNA-binding proteinc	188	3.00E-62	55%	91.35%	*Colletotrichum liriopes*	GKT41776.1
hypothetical protein B0T11DRAFT_7649	1667	0.00E+00	96%	57.78%	*Plectosphaerella cucumerina*	KAH7375268.1
subtilase	859	0.00E+00	97%	71.07%	*Plectosphaerella plurivora*	KAH6687843.1
Sorting nexin-4	475	0	100%	80.17%	*Plectosphaerella plurivora*	KAH6687844.1
mitochondrial distribution and morphology protein	542	0	94%	75.73%	*Plectosphaerella plurivora*	KAH6687845.1

adenylate kinase	268	4.00E-155	99%	80.97%	*Colletotrichum karsti*	XP_038745165.1

51) *Verticillium klebahnii* contig_ NMXL01000003, VRPKS-I-17 cluster

Gene name	Size	E value	% Coverage	% identity	Top blast hit	Accession number of top blast hit
mRNA degradation protein pet127, mitochondrial	234	3.00E-41	53%	59.20%	*Sodiomyces alkalinus F11*	XP_028466993.1
splicing factor 3b subunit 4	372	8.00E-157	85%	80.50%	*Colletotrichum camelliae*	KAH0444958.1
Mitochondrial FAD carrier protein FLX1	310	7.00E-158	99%	74.61%	*Colletotrichum chlorophyti*	OLN84903.1
Asparagine-rich zinc finger protein AZF1 like	439	1.00E-122	88%	50.25%	*Colletotrichum fioriniae PJ7*	EXF85239.1
ubiquitin-like modifier 1	104	1.00E-56	96%	80.00%	*Xylaria curta*	KAI0546841.1
elongator protein 2 (WD domain-containing protein)	840	0	100%	67.18%	*Colletotrichum tofieldiae*	KZL71342.1
hypothetical protein B0T11DRAFT_282059	113	9.00E-46	83%	75.53%	*Plectosphaerella cucumerina*	KAH7363092.1
Lovastatin diketide synthase LovF 16	2277	0	99%	71.80%	*Colletotrichum chlorophyti*	OLN86478.1
Esterase FUS5 like protein	255	2.00E-147	100%	79.69%	*Colletotrichum sojae*	KAF6806434.1
ABC transporter	1284	0	97%	70.71%	*Colletotrichum scovillei*	KAG7055337.1
RF-1 domain-containing protein	148	3.00E-41	70%	88.57%	*Colletotrichum siamense*	XP_036497959.1
Actin	375	0	100%	100.00%	*Tolypocladium paradoxum*	POR36442.1
hsp70-like protein	209	3.00E-96	97%	71.08%	*Sodiomyces alkalinus F11*	XP_028466987.1
54S ribosomal protein L7	356	0	98%	76.42%	*Sodiomyces alkalinus F11*	XP_028466988.1
ubiquitin C-terminal hydrolase	800	0	100%	64.03%	*Plectosphaerella cucumerina*	KAH7363099.1
6-phosphofructo-2-kinase	558	0	100%	82.87%	*Plectosphaerella cucumerina*	KAH7363100.1
Mitochondrial import receptor subunit tom-40	352	0	100%	83.33%	*Sodiomyces alkalinus F11*	XP_028466992.1
Isoflavone reductase	307	1.00E-63	98%	50.99%	*Colletotrichum higginsianum IMI 349063*	XP_018160455.1

52) *Verticillium klebahnii* contig_ NMXL01000003, VRPKS-I-23 cluster

Gene name	Size	E value	% Coverage	% identity	Top blast hit	Accession number of top blast hit
Ribokinase-like protein	366	6.00E-55	98%	36.80%	*Sodiomyces alkalinus F11*	XP_028463289.1
homoserine kinase	356	0	100%	90.17%	*Plectosphaerella cucumerina*	KAH7362595.1
SnodProt1	138	5.00E-66	100%	72.46%	*Plectosphaerella cucumerina*	KAH7368131.1
hypothetical protein B0T11DRAFT_355575	247	7.00E-07	35%	45.36%	*Plectosphaerella cucumerina*	KAH7358954.1
hypothetical protein F5X68DRAFT_4497	851	0	97%	62.98%	*Plectosphaerella plurivora*	KAH6697434.1

uncharacterized protein CGCS363_v012205	365	6.00E-105	93%	51.77%	*Colletotrichum siamense*	XP_036491211.1
aromatic prenyltransferase	456	0	100%	68.93%	*Plectosphaerella cucumerina*	KAH7358950.1
phenolpthiocerol synthesis polyketide synthase ppsA	2382	0	99%	46.01%	*Plectosphaerella plurivora*	KAH6697431.1
drug resistance transporter	588	0	99%	69.80%	*Plectosphaerella plurivora*	KAH6697430.1
hsp90-like protein	1386	0	99%	66.35%	*Plectosphaerella cucumerina*	KAH7358170.1
hypothetical protein B0T11DRAFT_299362	190	2.00E-62	59%	82.30%	*Plectosphaerella cucumerina*	KAH7358281.1
hypothetical protein B0T11DRAFT_284115	230	5.00E-98	100%	76.09%	*Plectosphaerella cucumerina*	KAH7358280.1
uncharacterized protein CORC01_08067	323	3.00E-168	100%	86.69%	*Colletotrichum orchidophilum*	XP_022473766.1
SRP54-domain-containing protein	689	0	100%	78.88%	*Sodiomyces alkalinus F11*	XP_028465413.1
hypothetical protein SODALDRAFT_201001	1689	0	99%	61.07%	*Sodiomyces alkalinus F11*	XP_028465411.1

53) *Verticillium klebahnii* contig_ NMXL01000003, VNRPKS-I-2 cluster

**Gene name**	**Size**	**E value**	**% Coverage**	**% identity**	**Top blast hit**	**Accession number of top blast hit**
major facilitator superfamily protein	529	0	96%	77.80%	*Sarocladium implicatum*	KAH8171005.1
TAP-like protein-domain-containing protein	539	0	100%	76.30%	*Plectosphaerella cucumerina*	KAH7367786.1
general substrate transporter	547	0	94%	80.35%	*Xylariomycetidae sp. FL2044*	KAH9886191.1
endoglucanase	253	3.00E-130	88%	92.86%	*Plectosphaerella cucumerina*	KAH7361706.1
cysteine synthase	316	0	99%	80.25%	*Fusarium decemcellulare*	KAF5004266.1
O-methyltransferase MdmC	184	3.00E-97	94%	82.76%	*Colletotrichum chlorophyti*	OLN96991.1
hypothetical protein B0T11DRAFT_330726	208	2.00E-49	85%	48.59%	*Plectosphaerella cucumerina*	KAH7358996.1
Bacteriodes thetaiotaomicron symbiotic chitinase	154	7.00E-51	98%	60.26%	*Scedosporium apiospermum*	XP_016643895.1
Bacteriodes thetaiotaomicron symbiotic chitinase	515	0	97%	52.05%	*Scedosporium apiospermum*	XP_016643895.1
PKS16 protein	2431	0	76%	80.96%	*Monosporascus ibericus*	RYP01440.1
putative monooxygenase	413	0	94%	83.17%	*Monosporascus ibericus*	RYP01441.1
hypothetical protein CDV31_016912	239	1.00E-64	66%	66.88%	*Fusarium ambrosium*	RSL82673.1

54) *Verticillium klebahnii* contig_ NMXL01000017, VRPKS-I-25 cluster

**Gene name**	**Size**	**E value**	**% Coverage**	**% identity**	**Top blast hit**	**Accession number of top blast hit**
RING-3 protein	1173	0	54%	69.46%	*Plectosphaerella plurivora*	KAH6680056.1
glucosidase II beta subunit-like protein-domain-containing protein	524	0	95%	63.62%	*Plectosphaerella plurivora*	KAH6680057.1

unnamed protein product	187	3.00E-90	99%	64.17%	*Clonostachys solani*	CAH0054579.1
hypothetical protein CFIO01_00827	673	3.00E-145	93%	49.08%	*Colletotrichum fioriniae PJ7*	EXF83685.1
hypothetical protein SODALDRAFT_165672	374	7.00E-52	74%	45.21%	*Sodiomyces alkalinus F11*	XP_028466392.1
S-adenosyl-L-methionine-dependent methyltransferase	313	1.00E-156	92%	81.44%	*Sodiomyces alkalinus F11*	XP_028466391.1
dihydroxy-acid dehydratase	616	0	99%	81.73%	*Plectosphaerella plurivora*	KAH6671001.1
polyketide synthase	2356	0	99%	89.34%	*Stachybotrys chartarum IBT 40293*	KFA46917.1
beta-xylosidase	563	0	94%	86.49%	*Colletotrichum tofieldiae*	GKT66924.1
hypothetical protein ColLi_06807	527	5.00E-158	85%	58.64%	*Colletotrichum liriopes*	GKT46626.1
uncharacterized protein CGMCC3_g2624	492	0	98%	67.36%	*Colletotrichum fructicola*	XP_031891081.1
Protein STB5-like protein 3	769	0	88%	61.73%	*Colletotrichum chlorophyti*	OLN81943.1
Sodium transport ATPase 5 like protein	1071	0	98%	74.11%	*Monosporascus sp. MG133*	RYP34097.1
bifunctional polynucleotide phosphatase/kinase	454	0	99%	67.32%	*Sodiomyces alkalinus F11*	XP_028468241.1
mediator complex, subunit Med18	269	5.00E-165	99%	80.07%	*Plectosphaerella cucumerina*	KAH7347312.1
hypothetical protein B0T11DRAFT_49340	364	7.00E-117	92%	51.00%	*Plectosphaerella cucumerina*	KAH7366962.1

55) *Verticillium klebahnii* contig_ NMXL01000002, VRPKS-I-13 cluster

Gene name	Size	E value	% Coverage	% identity	Top blast hit	Accession number of top blast hit
major facilitator superfamily domain-containing protein	203	2.00E-40	77%	46.34%	*Xylariales sp. PMI_506*	KAH8666575.1
hypothetical protein MCOR01_008058	449	1.00E-172	96%	56.04%	*Pyricularia oryzae*	KAH8841391.1
thiol-specific monooxygenase	396	0	100%	75.00%	*Plectosphaerella plurivora*	KAH6686480.1
glycogen debranching enzyme	665	0	98%	76.97%	*Dactylonectria macrodidyma*	KAH7115576.1
uncharacterized protein VDAG_02217	236	1.00E-71	100%	44.44%	*Verticillium dahliae VdLs.17*	XP_009656541.1
major facilitator superfamily domain-containing protein	583	0	100%	85.08%	*Plectosphaerella cucumerina*	KAH7368056.1
endoglucanase-1	233	2.00E-133	98%	78.60%	*Plectosphaerella cucumerina*	KAH7362705.1
hypothetical protein SODALDRAFT_4980	234	2.00E-49	66%	62.58%	*Sodiomyces alkalinus F11*	XP_028469806.1
epoxide hydrolase	409	0	98%	62.44%	*Plectosphaerella plurivora*	KAH6662678.1
Endoglucanase-4 like protein	296	7.00E-95	81%	56.56%	*Colletotrichum karsti*	XP_038740775.1
hypothetical protein BT63DRAFT_423135	233	2.00E-32	75%	74.14%	*Microthyrium microscopicum*	KAF2670841.1
polyketide synthase	3128	0	99%	53.50%	*Fusarium avenaceum*	KIL85244.1
hypothetical protein FALBO_5956	314	0	100%	85.67%	*Fusarium albosuccineum*	KAF4467173.1

hypothetical protein TRIATDRAFT_297088	194	2.00E-44	100%	45.88%	*Trichoderma atroviride IMI 206040*	XP_013948886.1
hypothetical protein CcaCcLH18_14085	488	0	97%	81.72%	*Colletotrichum camelliae*	KAH0420306.1
2OG-Fe oxygenase family protein	332	5.00E-159	99%	70.69%	*Colletotrichum incanum*	OHW94343.1

56) *Verticillium klebahnii* contig_ NMXL01000002, VRPKS-I-18 cluster

Gene name	Size	E value	% Coverage	% identity	Top blast hit	Accession number of top blast hit
PH and SEC7 domain-containing protein	1558	0	99%	56.35%	*Fusarium oxysporum f. sp. cubense*	TVY79447.1
glycosyl hydrolase family 16 protein	425	1.00E-135	64%	71.17%	*Colletotrichum truncatum*	XP_036580125.1
secreted protein	211	2.00E-93	90%	76.96%	*Sodiomyces alkalinus F11*	XP_028465800.1
Putative Sulfatase	526	0	98%	85.69%	*Talaromyces verruculosus*	KUL84026.1
general substrate transporter	518	0	98%	68.75%	*Dactylonectria macrodidyma*	KAH7160676.1
calcineurin-like phosphoesterase	363	0	100%	71.63%	*Plectosphaerella plurivora*	KAH6686639.1
serine hydrolase FSH	270	1.00E-124	99%	69.89%	*Fulvia fulva*	UJO18001.1
Reducing polyketide synthase PKS2	2561	0	77%	70.63%	*Fulvia fulva*	UJO18003.1
hypothetical protein BDW02DRAFT_369921	235	4.00E-49	100%	47.46%	*Decorospora gaudefroyi*	KAF1833738.1
carbohydrate-binding module family 50 protein	686	0	99%	58.67%	*Plectosphaerella plurivora*	KAH6684917.1
glycoside hydrolase superfamily	539	0	96%	80.50%	*Decorospora gaudefroyi*	KAF1833741.1
hypothetical protein P154DRAFT_517162	391	8.00E-122	100%	50.24%	*Amniculicola lignicola CBS 123094*	KAF2007437.1
SGNH hydrolase	711	0	100%	74.16%	*Macroventuria anomochaeta*	XP_033567680.1
glycoside hydrolase superfamily	734	0	98%	66.80%	*Plectosphaerella plurivora*	KAH6658770.1
hypothetical protein F5X68DRAFT_145888	1025	0	98%	55.68%	*Plectosphaerella plurivora*	KAH6658772.1
hypothetical protein B0T11DRAFT_325103	254	3.00E-123	98%	70.24%	*Plectosphaerella cucumerina*	KAH7366775.1
MFS general substrate transporter	320	5.00E-114	97%	56.91%	*Oidiodendron maius Zn*	KIN03209.1

57) *Verticillium klebahnii* contig_ NMXL01000006, VRPKS-I-19 cluster

Gene name	Size	E value	% Coverage	% identity	Top blast hit	Accession number of top blast hit
vacuolar protein sorting-associated protein	756	0	98%	74.28%	*Plectosphaerella cucumerina*	KAH7368195.1
putative beta-glucosidase G	832	0	99%	68.40%	*Aspergillus sydowii CBS 593.65*	XP_040708017.1
cutinase-2	224	5.00E-131	92%	87.44%	*Plectosphaerella cucumerina*	KAH7368196.1

major facilitator superfamily transporter [	1118	0	96%	67.96%	*Colletotrichum karsti*	XP_038748329.1
L-threonate dehydrogenase like protein	1108	0	99%	72.10%	*Colletotrichum graminicola M1.001*	XP_008095607.1
Type I Polyketide synthase	2177	0	99%	77.25%	*Leptosphaeria biglobosa*	KAH9873394.1
hypothetical protein J1614_005793	229	7.00E-96	97%	66.96%	*Leptosphaeria biglobosa*	KAH9873395.1
MFS general substrate transporter	575	0	98%	67.21%	*Sodiomyces alkalinus F11*	XP_028468956.1
thioredoxin-like protein	162	3.00E-85	98%	76.25%	*Hypoxylon sp. FL0890*	KAI0834638.1
hypothetical protein AK830_g4742	499	5.00E-87	91%	38.32%	*Neonectria ditissima*	KPM41855.1
exopolygalacturonase	461	0	93%	70.47%	*Monosporascus sp. mg162*	RYP54678.1
Obg-like ATPase 1	394	0	100%	90.36%	*Pyricularia grisea*	XP_030984371.1
DeSI-like protein sdu1	235	3.00E-114	99%	75.97%	*Colletotrichum chlorophyti*	OLN97068.1
putative 37S ribosomal protein S18 like	250	3.00E-91	100%	56.00%	*Colletotrichum fioriniae PJ7*	EXF75199.1
Mitochondrial import inner membrane translocase subunit tim44	541	0	100%	75.69%	*Sodiomyces alkalinus F11*	XP_028468965.1
N-glycosidase like protein	190	4.00E-56	83%	57.86%	*Plectosphaerella cucumerina*	KAH7347667.1
hypothetical protein ACRE_064810	287	3.00E-81	95%	51.26%	*Acremonium chrysogenum ATCC 11550*	KFH42767.1
hypothetical protein SODALDRAFT_333514	77	2.00E-21	97%	55.13%	*Sodiomyces alkalinus F11*	XP_028465568.1
alpha-glucosides permease MPH2/3	542	0	99%	75.41%	*Cylindrodendrum hubeiense*	KAF7546071.1

58) *Verticillium klebahnii* contig_ NMXL01000001, VRPKS-I-20 cluster

Gene name	Size	E value	% Coverage	% identity	Top blast hit	Accession number of top blast hit
Ubiquitin-activating enzyme E1-like	610	0	96%	77.68%	*Sodiomyces alkalinus F11*	XP_028470156.1
3-oxoacyl-[acyl-carrier-protein] reductase	271	2.00E-105	99%	71.33%	*Sodiomyces alkalinus F11*	XP_028470157.1
feruloyl esterase b	315	1.00E-175	93%	79.18%	*Colletotrichum musicola*	KAF6811137.1
Dihydrosphingosine 1-phosphate phosphatase	554	0	94%	73.57%	*Colletotrichum asianum*	KAF0325175.1
DNA damage-responsive transcriptional repressor RPH1	1748	0	62%	65.68%	*Colletotrichum fioriniae PJ7*	EXF81018.1
mannan endo-1,4-beta-mannosidase	401	0	95%	82.81%	*Plectosphaerella plurivora*	KAH6695781.1
cytochrome P450	1076	0	100%	75.19%	*Colletotrichum fioriniae PJ7*	EXF83899.1
polyketide synthase	2450	0	99%	67.20%	*Penicillium arizonense*	XP_022486633.1
hypothetical protein PENARI_c014G04062	651	1.00E-180	50%	72.21%	*Penicillium arizonense*	XP_022486531.1
Cytochrome P450 4V2	541	0	94%	65.59%	*Penicillium arizonense*	XP_022486670.1
BCL5p	527	2.00E-171	96%	53.35%	*Akanthomyces lecanii RCEF 1005*	OAA79799.1
hypothetical protein FALBO_6676	100	3.00E-20	93%	51.61%	*Fusarium albosuccineum*	KAF4466462.1
NMRAL1 protein	317	0	100%	80.13%	*Plectosphaerella plurivora*	KAH6661474.1

hypothetical protein B0J13DRAFT_486524	254	4.00E-135	100%	74.41%	*Dactylonectria estremocensis*	KAH7119443.1
NAD-binding Rossmann fold oxidoreductase family protein	346	0	99%	73.55%	*Talaromyces rugulosus*	XP_035348268.1
hypothetical protein EDB80DRAFT_562441	1169	0	59%	52.66%	*Ilyonectria destructans*	KAH7018764.1

59) *Verticillium klebahnii* contig_ NMXL01000010, PKS-III cluster

Gene name	Size	E value	% Coverage	% identity	Top blast hit	Accession number of top blast hit
NADH-ubiquinone oxidoreductase 30.4 kDa subunit	412	1.00E-51	81%	50.76%	*Plectosphaerella plurivora*	KAH6681192.1
short chain dehydrogenase	301	3.00E-167	96%	77.32%	*Sodiomyces alkalinus F11*	XP_028470310.1
centrin-3	189	2.00E-88	98%	68.09%	*Sodiomyces alkalinus F11*	XP_028470317.1
Iron-sulfur protein IND1	296	7.00E-177	99%	79.59%	*Colletotrichum truncatum*	XP_036586173.1
Splicing factor spf30	304	1.00E-109	99%	69.16%	*Colletotrichum truncatum*	XP_036586172.1
hypothetical protein B0T11DRAFT_249550	636	2.00E-173	96%	60.67%	*Plectosphaerella cucumerina*	KAH7375296.1
putative mitochondrial 2-oxoglutarate/malate carrier protein	335	0	100%	86.57%	*Colletotrichum chlorophyti*	OLN95994.1
hypothetical protein BN1708_004965	609	0	89%	70.00%	*Verticillium longisporum*	CRK29490.1
PAB-dependent poly(A)-specific ribonuclease subunit PAN2	1082	0	98%	79.13%	*Plectosphaerella cucumerina*	KAH7375292.1
fructose-bisphosphate aldolase	362	0	100%	90.88%	*Plectosphaerella cucumerina*	KAH7375300.1
putative chalcone and stilbene synthase domain-containing protein	444	0	100%	75.28%	*Colletotrichum sublineola*	KDN65911.1
hypothetical protein K456DRAFT_1765144	127	3.00E-23	86%	58.93%	*Colletotrichum gloeosporioides 23*	KAH9228351.1
TRAUB domain-containing protein	596	3.00E-131	78%	74.17%	*Plectosphaerella cucumerina*	KAH7375290.1
transcriptional activator protein acu-15	709	0	98%	73.83%	*Colletotrichum spaethianum*	GJC81216.1
Feruloyl esterase B	294	3.00E-151	92%	75.37%	*Colletotrichum orbiculare MAFF 240422*	TDZ25527.1
oxidoreductase	452	0	99%	65.11%	*Plectosphaerella plurivora*	KAH6681205.1
Acetyltransferase	270	1.00E-149	88%	83.61%	*Colletotrichum higginsianum IMI 349063*	XP_018159255.1
carbonic anhydrase	271	2.00E-146	93%	76.77%	*Colletotrichum incanum*	KZL87566.1
Six-hairpin glycosidase-like protein	892	0	91%	69.76%	*Plectosphaerella cucumerina*	KAH7353461.1
putative MFS hexose transporter	540	0	99%	83.64%	*Plectosphaerella cucumerina*	KAH7353460.1

60) *Verticillium klebahnii* contig_ NMXL01000010, VRPKS-I-26 cluster

Gene name	Size	E value	% Coverage	% identity	Top blast hit	Accession number of top blast hit
hypothetical protein SODALDRAFT_319240	134	4.00E-25	95%	39.39%	*Sodiomyces alkalinus F11*	XP_028470409.1
alpha-l-rhamnosidase	1219	0	71%	68.12%	*Colletotrichum truncatum*	XP_036586343.1
FAD-dependent monooxygenase	453	0	100%	85.65%	*Stachybotrys elegans*	KAH7303249.1
heterokaryon incompatibility protein-domain-containing protein	741	0	100%	75.40%	*Chaetomium sp. MPI-CAGE-AT-0009*	KAH6847694.1
retinol dehydrogenase	312	0	100%	78.53%	*Plectosphaerella plurivora*	KAH6675343.1
TTL domain-containing protein	473	0	100%	73.24%	*Coniochaeta sp. 2T2.1*	KAB5518934.1
RecName: Full=Highly reducing polyketide synthase apmlA; Short=HRPKS apmlA; AltName: Full=Phaeospelide A biosynthesis cluster protein apmlA	2349	0	99%	49.55%	*Apiospora sphaerosperma*	P0CU84.1
uncharacterized protein KVR01_004169	257	7.00E-06	22%	45.61%	*Diaporthe batatas*	XP_044646333.1
hypothetical protein B0T11DRAFT_318355	163	4.00E-55	98%	65.85%	*Plectosphaerella cucumerina*	KAH7362742.1
Retinoic acid induced 16-like protein-domain-containing protein	1004	0	100%	72.63%	*Plectosphaerella cucumerina*	KAH7362743.1
putative pectate lyase F	236	6.00E-141	97%	84.42%	*Colletotrichum fructicola*	XP_031890093.1
hypothetical protein PFICI_10960	1230	0	83%	51.88%	*Pestalotiopsis fici W106-1*	XP_007837732.1
hypothetical protein	578	0	96%	88.51%	*Colletotrichum higginsianum IMI 349063*	XP_018157666.1
peptidase family C25-domain-containing protein	1019	0	99%	56.89%	*Paraphoma chrysanthemicola*	KAH7091334.1

61) *Verticillium klebahnii* contig_ NMXL01000019, VRPKS-I-14 cluster

Gene name	Size	E value	% Coverage	% identity	Top blast hit	Accession number of top blast hit
hypothetical protein F5883DRAFT_436293	1518	0	96%	79.66%	*Diaporthaceae sp. PMI_573*	KAH8748597.1
hypothetical protein FALBO_210	144	5.00E-63	85%	77.24%	*Fusarium albosuccineum*	KAF4472888.1
hypothetical protein HYQ45_016732	88	2.00E-04	100%	71.43%	*Verticillium longisporum*	KAG7113540.1
FK506-binding protein	212	1.00E-82	100%	67.14%	*Plectosphaerella cucumerina*	KAH7367672.1
Protein rds1 like protein	465	0	98%	79.26%	*Sodiomyces alkalinus F11*	XP_028471380.1
Vanillyl-alcohol oxidase	562	0	99%	86.58%	*Plectosphaerella plurivora*	KAH6673908.1
hypothetical protein B0T11DRAFT_56928	204	2.00E-98	100%	71.50%	*Plectosphaerella cucumerina*	KAH7367670.1
NAD(P)-binding protein	266	2.00E-85	82%	58.45%	*Cenococcum geophilum 1.58*	OCK87093.1
hypothetical protein CEP52_003390	374	5.00E-158	97%	69.86%	*Fusarium sp. AF-4*	RSM10820.1
cytochrome P450	506	0	100%	79.05%	*Plectosphaerella cucumerina*	KAH7367666.1
major facilitator superfamily domain-containing protein	595	0	82%	61.62%	*Truncatella angustata*	XP_045957260.1

putative polyketide synthase	3116	0	100%	57.03%	*Truncatella angustata*	KAH8198320.1
fatty acid desaturase domain-containing protein	608	0	100%	63.58%	*Sarocladium implicatum*	KAH8177350.1
Acyl-CoA dehydrogenase/oxidase	130	2.00E-37	88%	56.52%	*Fusarium sp. DS 682*	KAF9769306.1
Acyl-CoA dehydrogenase/oxidase	442	3.00E-128	91%	51.98%	*Cordyceps javanica*	TQV95615.1
hypothetical protein F5X99DRAFT_371098	612	6.00E-131	94%	41.67%	*Biscogniauxia marginata*	KAI1504825.1
cytochrome P450 CYP4/CYP19/CYP26 subfamilies protein	519	0	95%	77.98%	*Colletotrichum karsti*	XP_038744559.1
hypothetical protein F4819DRAFT_459179	866	3.00E-105	91%	33.26%	*Hypoxylon fuscum*	KAI1401228.1

62) *Verticillium klebahnii* contig_ NMXL01000013, VRPKS-I-21 cluster

Gene name	Size	E value	% Coverage	% identity	Top blast hit	Accession number of top blast hit
hypothetical protein ColTof4_01230	279	2.00E-85	97%	58.97%	*Colletotrichum tofieldiae*	GKT68807.1
reverse transcriptase	771	0	99%	87.13%	*Purpureocillium lilacinum*	XP_018173011.1
reverse transcriptase	436	0	89%	82.35%	*Purpureocillium lilacinum*	XP_018173011.1
hypothetical protein ACRE_090730	1474	2.00E-112	49%	38.69%	*Acremonium chrysogenum ATCC 11550*	KFH40265.1
polyketide synthase	2351	0	92%	69.20%	*Colletotrichum plurivorum*	KAF6826616.1
predicted protein	105	8.00E-19	67%	58.90%	*Verticillium alfalfae VaMs.102*	XP_002999652.1
Zinc-type alcohol dehydrogenase-like protein C16A3.02c	110	4.00E-18	99%	40.17%	*Verticillium dahliae VDG2*	KAF3351609.1
hypothetical protein B0J13DRAFT_566569	295	2.00E-38	40%	50.83%	*Dactylonectria estremocensis*	KAH7123403.1
hypothetical protein GQ607_002381	167	4.00E-19	95%	36.93%	*Colletotrichum asianum*	KAF0330502.1
Poly(ADP-ribose) polymerase, catalytic domain protein	391	9.00E-09	23%	36.96%	*Pseudogymnoascus sp. VKM F-3808*	KFX93482.1

63) *Verticillium klebahnii* contig_ NMXL01000003, VRPKS-I-12 and VNRPKS-I-1 clusters

Gene name	Size	E value	% Coverage	% identity	Top blast hit	Accession number of top blast hit
hypothetical protein FPOAC1_009761	238	2.00E-81	89%	59.26%	*Fusarium* poae	XP_044706852.1
cytochrome P450	477	0	95%	75.55%	*Sodiomyces alkalinus F11*	XP_028469280.1
beta-ketoacyl synthase	366	9.00E-89	99%	46.51%	*Thelonectria olida*	KAH6899712.1
eukaryotic aspartyl protease	454	3.00E-175	96%	53.65%	*Colletotrichum simmondsii*	KXH41725.1
putative monooxygenase	419	0	93%	72.11%	*Sodiomyces alkalinus F11*	XP_028469276.1
protein kinase subdomain-containing protein	890	0	86%	70.91%	*Sodiomyces alkalinus F11*	XP_028469275.1
polyketide synthase	3943	0	97%	75.14%	*Sodiomyces alkalinus F11*	XP_028469272.1
pyridoxal-dependent decarboxylase	514	0	88%	67.32%	*Plectosphaerella cucumerina*	KAH7358159.1

duf92 domain protein	391	8.00E-132	99%	62.63%	*Sodiomyces alkalinus F11*	XP_028469270.1
uncharacterized protein CTRU02_08118	316	0.005	50%	26.47%	*Colletotrichum truncatum*	XP_036582041.1
laccase-like multicopper oxidase	561	0	98%	67.88%	*Plectosphaerella cucumerina*	KAH7358155.1
Conidial pigment polyketide synthase PfmaE like protein	2188	0	100%	79.23%	*Colletotrichum karsti*	XP_038751444.1
oxidoreductase	325	0	99%	84.57%	*Colletotrichum truncatum*	XP_036586400.1
transcription factor	424	3.00E-148	100%	56.88%	*Colletotrichum incanum*	KZL86702.1
transcription factor Cmr1	926	0	98%	73.07%	*Colletotrichum musicola*	KAF6845250.1
glycosyl transferase family protein	325	9.00E-164	100%	77.91%	*Plectosphaerella cucumerina*	KAH7358014.1
dihydrodipicolinate synthase	331	0	98%	81.90%	*Plectosphaerella cucumerina*	KAH7358015.1

64) *Verticillium klebahnii* contig_ NMXL01000011, VRPKS-I-9 cluster

Gene name	Size	E value	% Coverage	% identity	Top blast hit	Accession number of top blast hit
FAD dependent oxidoreductase	334	0	95%	77.36%	*Plectosphaerella cucumerina*	KAH7368673.1
major facilitator superfamily domain-containing protein	517	0	86%	85.08%	*Plectosphaerella cucumerina*	KAH7368674.1
uncharacterized protein B0I36DRAFT_355828	178	8.00E-11	37%	78.79%	*Microdochium trichocladiopsis*	XP_046005612.1
putative amidase	558	0	99%	78.82%	*Plectosphaerella plurivora*	KAH6684967.1
lovastatin nonaketide synthase	4064	0	99%	72.49%	*Hirsutella rhossiliensis*	XP_044717051.1
hypothetical protein B0T11DRAFT_345058	549	5.00E-38	61%	32.69%	*Plectosphaerella cucumerina*	KAH7375153.1
lipase	560	0	96%	69.33%	*Plectosphaerella cucumerina*	KAH7376670.1
polysaccharide deacetylase	518	3.00E-156	69%	78.33%	*Plectosphaerella cucumerina*	KAH7374645.1
hypothetical protein B0T11DRAFT_318679	275	8.00E-135	98%	67.52%	*Plectosphaerella cucumerina*	KAH7363183.1
predicted protein	275	3.00E-07	38%	36.45%	*Verticillium alfalfae VaMs.102*	XP_003001333.1
hypothetical protein ISF_06027	85	8.00E-22	75%	57.81%	*Cordyceps fumosorosea ARSEF 2679*	XP_018703129.1
tetratricopeptide repeat protein 1	285	6.00E-109	89%	79.22%	*Colletotrichum karsti*	XP_038750970.1
HAD-like protein	270	4.00E-141	99%	71.17%	*Thozetella sp. PMI_491*	KAH8895695.1
C6 zinc finger domain-containing protein	518	0	99%	74.63%	*Colletotrichum karsti*	XP_038750968.1

65) *Verticillium klebahnii* contig_ NMXL01000020, VRPKS-I-2 cluster

Gene name	Size	E value	% Coverage	% identity	Top blast hit	Accession number of top blast hit

Fusaric acid cluster transcription factor FUB10	425	1.00E-158	100%	72.64%	*Colletotrichum fructicola*	KAF4886529.1
Alcohol dehydrogenase superfamily, zinc-type	353	0	100%	90.65%	*Colletotrichum fructicola*	XP_031875992.1
putative polyketide synthase	3935	0	99%	86.73%	*Colletotrichum fructicola*	KAF4886526.1
Alpha/beta hydrolase	433	0	99%	85.61%	*Colletotrichum fructicola*	XP_031875995.1
MFS general substrate transporter-like protein	580	0	100%	85.69%	*Colletotrichum fructicola*	KAF4886521.1
uncharacterized protein CGMCC3_g17405	193	8.00E-123	100%	86.53%	*Colletotrichum fructicola*	XP_031875990.1
D-isomer specific 2-hydroxyacid dehydrogenase	326	0	99%	78.29%	*Plectosphaerella plurivora*	KAH6689062.1
putative transcriptional regulatory protein	466	4.00E-111	99%	50.24%	*Plectosphaerella plurivora*	KAH6689063.1
SMR domain-containing protein	730	0	100%	84.04%	*Colletotrichum graminicola M1.001*	XP_008090428.1

66) *Verticillium klebahnii* contig_ NMXL01000008, VRPKS-I-5 cluster

Gene name	Size	E value	% Coverage	% identity	Top blast hit	Accession number of top blast hit
pseudouridine synthase	599	0	97%	52.15%	*Sodiomyces alkalinus F11*	XP_028471325.1
GPI-GlcNAc transferase complex	222	1.00E-45	99%	61.06%	*Colletotrichum graminicola M1.001*	XP_008094050.1
hypothetical protein SODALDRAFT_41197	1664	0	98%	73.48%	*Sodiomyces alkalinus F11*	XP_028471323.1
glutathione S-transferase	224	1.00E-123	99%	78.03%	*fungal sp. No.14919*	GAW23379.1
MFS general substrate transporter	433	7.00E-130	96%	66.11%	*Glarea lozoyensis ATCC 20868*	XP_008088685.1
major facilitator superfamily-domain-containing protein	612	0	88%	73.43%	*Biscogniauxia mediterranea*	KAI1639146.1
hypothetical protein BGAL_0184g00240	256	4.00E-75	86%	53.81%	*Botrytis galanthina*	THV49682.1
hypothetical protein B0I37DRAFT_378309	506	4.00E-71	62%	57.67%	*Chaetomium sp. MPI-CAGE-AT-0009*	KAH6844803.1
lovastatin nonaketide synthase protein	3912	0	99%	64.69%	*Biscogniauxia mediterranea*	KAI1485991.1
DUF636 domain-containing protein	137	9.00E-69	100%	85.40%	*Purpureocillium lilacinum*	XP_018184317.1
hypothetical protein B0T11DRAFT_333834	160	7.00E-32	63%	64.71%	*Plectosphaerella cucumerina*	KAH7347765.1
Isochorismatase-like protein	244	6.00E-126	90%	76.13%	*Plectosphaerella plurivora*	KAH6676014.1
hypothetical protein jhhlp_008516	905	0	89%	74.57%	*Lomentospora prolificans*	PKS05149.1
putative transporter	111	4.00E-52	100%	79.82%	*Tolypocladium ophioglossoides CBS 100239*	KND87833.1

67) *Verticillium klebahnii* contig_ NMXL01000002, VRPKS-I-6 cluster

Gene name	Size	E value	% Coverage	% identity	Top blast hit	Accession number of top blast hit
COX17 protein	71	4.00E-21	70%	82.00%	*Diaporthaceae sp. PMI_573*	KAH8788412.1
hypothetical protein HYQ45_010318	159	6.00E-46	78%	64.80%	*Verticillium longisporum*	KAG7131019.1
protein PXR1	364	4.00E-72	48%	83.33%	*Plectosphaerella cucumerina*	KAH7368324.1
MFS sugar transporter	477	0	97%	67.34%	*Colletotrichum sojae*	KAF6797496.1
hypothetical protein CSPAE12_03025	640	2.00E-129	80%	48.56%	*Colletotrichum incanum*	OHW98243.1
hypothetical protein C8035_v000460	325	2.00E-130	97%	63.47%	*Colletotrichum spinosum*	TDZ12713.1
hypothetical protein B0T10DRAFT_76705	277	3.00E-51	77%	47.26%	*Thelonectria olida*	KAH6887013.1
Transaldolase	365	0	100%	86.61%	*Colletotrichum siamense*	KAF4850344.1
membrane transporter D1	599	0	99%	82.24%	*Ilyonectria destructans*	KAH7006833.1
thioredoxin	328	4.00E-173	100%	74.09%	*Plectosphaerella cucumerina*	KAH7368325.1
alpha beta hydrolase protein	271	1.00E-120	94%	64.20%	*Colletotrichum truncatum*	XP_036575502.1
serine hydrolase-domain-containing protein	284	7.00E-132	100%	63.38%	*Xylariaceae sp. FL0016*	KAI1344560.1
Polyketide synthase-nonribosomal peptide synthetase	4028	0	99%	66.66%	*Aspergillus burnettii*	KAF5861957.1
hypothetical protein CC78DRAFT_576631	470	0	100%	65.74%	*Didymosphaeria enalia*	KAF2268082.1
P-loop containing nucleoside triphosphate hydrolase protein	1120	0	98%	56.49%	*Clohesyomyces aquaticus*	ORY13587.1
solid-state culture-specific protein-like protein	466	0	99%	63.42%	*Didymosphaeria enalia*	KAF2268085.1
cytochrome P450	458	0	94%	67.28%	*Aspergillus alliaceus*	XP_031905229.1
putative alcohol dehydrogenase	370	0	100%	79.73%	*Xylariaceae sp. FL0016*	KAI1344559.1
cytochrome P450	494	0	95%	64.23%	*Aspergillus avenaceus*	KAE8150739.1

68) *Verticillium longisporum* contig_ CVQH01005557, VRPKS-I-22 cluster

Gene name	Size	E value	% Coverage	% identity	Top blast hit	Accession number of top blast hit
phospholipase D1	1095	0	100%	76.61%	*Plectosphaerella cucumerina*	KAH7357821.1
tubulin gamma chain	461	0	100%	96.32%	*Plectosphaerella cucumerina*	KAH7357822.1
uncharacterized protein BCR38DRAFT_425087	93	5.00E-37	94%	73.86%	*Pseudomassariella vexata*	XP_040719305.1
hypothetical protein SODALDRAFT_68083	884	2.00E-08	14%	36.57%	*Sodiomyces alkalinus F11*	XP_028463302.1
P-loop containing nucleoside triphosphate hydrolase protein	250	3.00E-106	100%	67.20%	*Plectosphaerella cucumerina*	KAH7357817.1

hypothetical protein FNYG_02927	345	5.00E-125	97%	56.01%	*Fusarium nygamai*	PNP83710.1
major facilitator superfamily transporter	169	7.00E-77	100%	75.74%	*Cytospora leucostoma*	ROW13285.1
hypothetical protein BU23DRAFT_475183	419	0	87%	72.19%	*Bimuria novae-zelandiae CBS 107.79*	KAF1970089.1
DUF341 domain-containing protein	207	2.00E-98	100%	63.76%	*Fusarium napiforme*	KAF5540875.1
UDP-glucosyl transferase family protein	553	0	98%	60.88%	*Fusarium sp. NRRL 66182*	KAF5020447.1
polyketide synthase	2632	0	99%	70.23%	*Fusarium mexicanum*	KAF5539484.1
sulfate transporter family-domain-containing protein	805	0	99%	53.77%	*Bisporella sp. PMI_857*	KAH8586595.1
hypothetical protein DL769_010058	281	1.00E-119	100%	57.00%	*Monosporascus sp. CRB-8-3*	RYP55738.1
hypothetical protein B0T11DRAFT_99011	247	2.00E-10	33%	39.02%	*Plectosphaerella cucumerina*	KAH7358030.1
hypothetical protein CNYM01_04222	153	2.00E-67	99%	78.29%	*Colletotrichum nymphaeae SA-01*	KXH64953.1
hypothetical protein F5X68DRAFT_276198	130	8.00E-43	94%	64.23%	*Plectosphaerella plurivora*	KAH6686951.1
MSF1 protein	192	1.00E-115	98%	85.94%	*Sodiomyces alkalinus F11*	XP_028463299.1
amine oxidase	734	0	90%	79.29%	*Colletotrichum incanum*	KZL83967.1
cytochrome c oxidase assembly protein CtaG/Cox11	238	3.00E-133	87%	86.06%	*Colletotrichum scovillei*	XP_035329109.1
60S ribosomal protein L26-1	136	1.00E-67	100%	85.40%	*Plectosphaerella cucumerina*	KAH7347842.1

69) *Verticillium longisporum* contig_ CVQH01020307, VRPKS-I-23 cluster

Gene name	Size	E value	% Coverage	% identity	Top blast hit	Accession number of top blast hit
tubulin gamma chain	461	0	100%	96.32%	*Plectosphaerella cucumerina*	KAH7357822.1
uncharacterized protein BCR38DRAFT_425087	90	5.00E-37	97%	73.86%	*Pseudomassariella vexata*	XP_040719305.1
hypothetical protein SODALDRAFT_68083	883	4.00E-09	13%	37.01%	*Sodiomyces alkalinus F11*	XP_028463302.1
P-loop containing nucleoside triphosphate hydrolase protein	251	2.00E-106	100%	67.73%	*Plectosphaerella cucumerina*	KAH7357817.1
hypothetical protein FSARC_10491	332	7.00E-127	97%	54.91%	*Fusarium sarcochroum*	KAF4960431.1
major facilitator superfamily transporter	594	0	89%	78.11%	*Colletotrichum graminicola M1.001*	XP_008093595.1
FAD dependent	324	5.00E-123	86%	57.27%	*Fusarium sp. NRRL 25303*	KAF5635100.1
DUF341 domain-containing protein	207	1.00E-98	100%	64.22%	*Fusarium napiforme*	KAF5540875.1
hypothetical protein F66182_7526	552	0	98%	61.13%	*Fusarium sp. NRRL 66182*	KAF5020447.1
polyketide synthase	2635	0	99%	70.43%	*Fusarium mexicanum*	KAF5539484.1

sulfate transporter family-domain-containing protein	767	0	99%	53.27%	*Bisporella sp. PMI_857*	KAH8586595.1
hypothetical protein FPSE5266_02886	339	1.00E-18	90%	24.26%	*Fusarium pseudograminearum*	KAF0638536.1
hypothetical protein CNYM01_04222	115	6.00E-53	95%	74.55%	*Colletotrichum nymphaeae SA-01*	KXH64953.1
hypothetical protein F5X68DRAFT_276198	130	2.00E-46	98%	64.84%	*Plectosphaerella plurivora*	KAH6686951.1
MSF1 protein	192	5.00E-115	91%	88.07%	*Sodiomyces alkalinus F11*	XP_028463299.1
methionine aminopeptidase 2-like protein	441	0	100%	84.98%	*Plectosphaerella plurivora*	KAH6686947.1
Peroxiredoxin-like 2C	395	5.00E-142	73%	63.87%	*Plectosphaerella plurivora*	KAH6686952.1
hypothetical protein IFR05_013588	289	1.00E-04	59%	24.18%	*Cadophora sp. M221*	KAG4430930.1
branched-chain-amino-acid aminotransferase	476	0	88%	76.18%	*Plectosphaerella plurivora*	KAH6686954.1
branched-chain-amino-acid aminotransferase	89	2.00E-15	84%	60.76%	*Plectosphaerella plurivora*	KAH6686954.1
ethanolamine-phosphate cytidylyltransferase	447	0	93%	85.44%	*Plectosphaerella cucumerina*	KAH7362590.1
hypothetical protein B0T11DRAFT_85792	433	7.00E-173	86%	77.04%	*Plectosphaerella cucumerina*	KAH7362591.1
WD domain-containing protein	358	0	100%	82.68%	*Colletotrichum simmondsii*	KXH43027.1
hypothetical protein FHL15_001926	159	2.00E-19	94%	42.48%	*Xylaria flabelliformis*	TRX97132.1
Ribokinase-like protein	369	1.00E-41	99%	36.24%	*Sodiomyces alkalinus F11*	XP_028463289.1
homoserine kinase	244	3.00E-91	96%	95.53%	*Plectosphaerella cucumerina*	KAH7362595.1
uncharacterized protein VDAG_01851	155	9.00E-74	100%	92.26%	*Verticillium dahliae VdLs.17*	XP_009656175.1
SnodProt1	138	4.00E-64	100%	68.12%	*Plectosphaerella cucumerina*	KAH7368131.1
hypothetical protein B0T11DRAFT_355575	258	8.00E-13	84%	35.56%	*Plectosphaerella cucumerina*	KAH7358954.1
hypothetical protein F5X68DRAFT_4497	600	5.00E-151	97%	61.12%	*Plectosphaerella plurivora*	KAH6697434.1
hypothetical protein SODALDRAFT_82945	363	4.00E-89	100%	46.84%	*Sodiomyces alkalinus F11*	XP_028462491.1
phenolpthiocerol synthesis polyketide synthase ppsA	2305	0	98%	47.31%	*Sodiomyces alkalinus F11*	XP_028462492.1
drug resistance transporter	485	0	98%	69.52%	*Plectosphaerella cucumerina*	KAH7358948.1
hypothetical protein BN1723_	578	7.00E-90	56%	51.06%	*Plectosphaerella cucumerina*	KAH7358170.1
2-oxoacid dehydrogenase acyltransferase	523	0	100%	66.29%	*Plectosphaerella cucumerina*	KAH7375969.1
ATP10 protein	343	4.00E-128	99%	58.52%	*Plectosphaerella cucumerina*	KAH7375968.1
vesicle transport V-SNARE protein	232	2.00E-129	93%	80.73%	*Plectosphaerella plurivora*	KAH6691283.1

70) *Verticillium longisporum* contig_ CVQH01023638, VRPKS-I-17 cluster

Gene name	Size	E value	% Coverage	% identity	Top blast hit	Accession number of top blast hit
Lovastatin diketide synthase LovF 16	2158	0	97%	69.51%	*Colletotrichum chlorophyti*	OLN86478.1
uncharacterized protein SMAC_07940	114	2.00E-61	97%	83.04%	*Sordaria macrospora k-hell*	XP_003350624.1
ABC transporter	1601	0	83%	66.96%	*Colletotrichum nymphaeae SA-01*	KXH49589.1
Actin	375	0	100%	99.73%	*Tolypocladium paradoxum*	POR36442.1
hsp70-like protein	209	3.00E-94	98%	67.77%	*Plectosphaerella plurivora*	KAH6686283.1
54S ribosomal protein L7	352	0	99%	73.14%	*Sodiomyces alkalinus F11*	XP_028466988.1
ubiquitin C-terminal hydrolase	1066	0	87%	66.80%	*Plectosphaerella cucumerina*	KAH7363099.1
6-phosphofructo-2-kinase	341	0	100%	86.46%	*Plectosphaerella plurivora*	KAH6686287.1
Mitochondrial import receptor subunit tom-40	146	1.00E-84	99%	86.30%	*Colletotrichum higginsianum IMI 349063*	XP_018160456.1

71) *Verticillium longisporum* contig_ CVQH01020607, VRPKS-I-23 cluster

Gene name	Size	E value	% Coverage	% identity	Top blast hit	Accession number of top blast hit
virulence sensor protein bvgS	234	2.00E-112	100%	72.77%	*Plectosphaerella cucumerina*	KAH7358170.1
drug resistance transporter	524	0	97%	69.40%	*Plectosphaerella cucumerina*	KAH7358948.1
phenolpthiocerol synthesis polyketide synthase ppsA	2291	0	99%	49.04%	*Sodiomyces alkalinus F11*	XP_028462492.1
hypothetical protein SODALDRAFT_82945	363	4.00E-88	100%	46.84%	*Sodiomyces alkalinus F11*	XP_028462491.1
hypothetical protein F5X68DRAFT_4497	719	0	93%	63.05%	*Plectosphaerella plurivora*	KAH6697434.1
hypothetical protein F5X68DRAFT_257601	242	3.00E-12	63%	45.11%	*Plectosphaerella plurivora*	KAH6697435.1
SnodProt1	138	2.00E-65	100%	70.29%	*Plectosphaerella cucumerina*	KAH7368131.1
homoserine kinase	501	0	88%	94.22%	*Plectosphaerella cucumerina*	KAH7362595.1
hypothetical protein SODALDRAFT_67746	367	9.00E-48	99%	36.10%	*Sodiomyces alkalinus F11*	XP_028463289.1

72) *Verticillium longisporum* contig_ CVQH01022527, VRPKS-I-26 cluster

Gene name	Size	E value	% Coverage	% identity	Top blast hit	Accession number of top blast hit
hypothetical protein AK830_g141	353	7.00E-161	84%	82.55%	*Neonectria ditissima*	KPM46366.1
short chain dehydrogenase reductase	292	2.00E-103	99%	55.33%	*Colletotrichum truncatum*	XP_036579832.1
regulator-ty1 transposition protein	166	2.00E-27	89%	40.94%	*Colletotrichum incanum*	OHW92586.1
ankyrin repeat-containing domain protein	778	8.00E-122	96%	35.94%	*Plectosphaerella plurivora*	KAH6670890.1

hypothetical protein F5X68DRAFT_228683	77	3.00E-17	62%	77.55%	*Plectosphaerella plurivora*	KAH6692307.1
peptidase family C25-domain-containing protein	1017	0	100%	54.67%	*Paraphoma chrysanthemicola*	KAH7091334.1
hypothetical protein CMUS01_12901	577	0	93%	87.15%	*Colletotrichum musicola*	KAF6813152.1
hypothetical protein F5X68DRAFT_158933	365	0	98%	79.18%	*Plectosphaerella plurivora*	KAH6670884.1
choline dehydrogenase	616	0	97%	80.94%	*Plectosphaerella cucumerina*	KAH7368263.1
hypothetical protein CSUB01_04894	991	0	100%	71.51%	*Plectosphaerella cucumerina*	KAH7362743.1
hypothetical protein B0T11DRAFT_318355	162	3.00E-57	98%	66.46%	*Plectosphaerella cucumerina*	KAH7362742.1
hypothetical protein F5B20DRAFT_586947	1201	0	88%	63.21%	*Whalleya microplaca*	KAI1073750.1
hypothetical protein F5B20DRAFT_586947	342	9.00E-111	98%	55.39%	*Whalleya microplaca*	KAI1073750.1
TTL domain-containing protein	470	0	100%	69.98%	*Coniochaeta sp. 2T2.1*	KAB5518934.1
retinol dehydrogenase	300	1.00E-156	100%	73.08%	*Plectosphaerella plurivora*	KAH6675343.1

73) *Verticillium longisporum* contig_ CVQH01000225, VRPKS-I-17 cluster

Gene name	Size	E value	% Coverage	% identity	Top blast hit	Accession number of top blast hit
Asparagine-rich zinc finger protein AZF1	441	5.00E-115	93%	49.53%	*Colletotrichum trifolii*	TDZ74112.1
Asparagine-rich zinc finger protein AZF1	444	4.00E-113	93%	49.19%	*Colletotrichum trifolii*	TDZ74112.1
Ubiquitin-related modifier 1	79	1.00E-29	93%	70.27%	*Colletotrichum chlorophyti*	OLN84992.1
elongator protein 2 (WD domain-containing protein)	330	1.00E-110	87%	61.72%	*Colletotrichum tofieldiae*	KZL71342.1
Ubiquitin- modifier 1	104	9.00E-55	99%	78.64%	*Claviceps pusilla*	KAG6012378.1
elongator protein 2 (WD domain-containing protein)	824	0	99%	66.67%	*Colletotrichum tofieldiae*	KZL71342.1
Elongator complex protein 2	739	5.00E-162	47%	72.24%	*Colletotrichum chlorophyti*	OLN84991.1
Elongator complex protein 2 like	143	2.00E-37	98%	61.54%	*Plectosphaerella plurivora*	KAH6686279.1
hypothetical protein SODALDRAFT_332602	114	7.00E-44	78%	76.67%	*Sodiomyces alkalinus F11*	XP_028466984.1
Lovastatin diketide synthase LovF 16	2268	0	99%	69.14%	*Colletotrichum chlorophyti*	OLN86478.1
hypothetical protein	255	6.00E-150	100%	78.12%	*Colletotrichum sojae*	KAF6806434.1
ABC transporter	1319	0	98%	68.73%	*Colletotrichum nymphaeae SA-01*	KXH49589.1
peptidyl-tRNA hydrolase domain-containing protein	194	3.00E-44	71%	69.57%	*Pyricularia oryzae 70-15*	XP_003719872.1

Actin	375	0	100%	99.47%	*Tolypocladium paradoxum*	POR36442.1
hsp70-like protein	209	6.00E-94	98%	67.77%	*Plectosphaerella plurivora*	KAH6686283.1
54S ribosomal protein L7	352	0	99%	73.14%	*Sodiomyces alkalinus F11*	XP_028466988.1
ubiquitin C-terminal hydrolase	624	0	100%	62.88%	*Plectosphaerella cucumerina*	KAH7363099.1
6-phosphofructo-2-kinase	572	0	100%	84.83%	*Plectosphaerella cucumerina*	KAH7363100.1
duf410 domain-containing protein	194	1.00E-65	69%	81.48%	*Colletotrichum sojae*	KAF6819727.1

74) *Verticillium longisporum* contig_ CVQH01002447, VRPKS-I-19 cluster

Gene name	Size	E value	% Coverage	% identity	Top blast hit	Accession number of top blast hit
Type I Iterative PKS	1896	0	100%	77.60%	*Leptosphaeria biglobosa*	KAH9873394.1
hypothetical protein J1614_005793	289	2.00E-117	90%	64.12%	*Leptosphaeria biglobosa*	KAH9873395.1
L-threonate dehydrogenase like protei	1132	0	99%	72.76%	*Colletotrichum scovillei*	XP_035328982.1
hypothetical protein F5X68DRAFT_50483	621	0	100%	67.62%	*Plectosphaerella plurivora*	KAH6669659.1
MFS transporter prlL like protein	488	0	99%	75.61%	*Colletotrichum chlorophyti*	OLN87319.1
hypothetical protein HYQ44_014562	188	8.00E-83	92%	83.91%	*Verticillium longisporum*	KAG7105862.1
cytochrome P450	540	2.00E-122	96%	37.52%	*Ilyonectria sp. MPI-CAGE-AT-0026*	KAH6972830.1
alpha/beta-hydrolase	350	2.00E-113	97%	54.39%	*Zopfia rhizophila CBS 207.26*	KAF2180147.1
cutinase-2	224	6.00E-132	92%	89.37%	*Plectosphaerella cucumerina*	KAH7368196.1
flavonol synthase-like protein	357	0	100%	71.76%	*Colletotrichum graminicola M1.001*	XP_008089570.1
hypothetical protein	76	2.00E-37	100%	86.84%	*Plectosphaerella plurivora*	KAH6687904.1
NADH2 dehydrogenase 14K chain	90	1.00E-36	76%	85.51%	*Plectosphaerella cucumerina*	KAH7369386.1
translation initiation factor eIF-2B subunit delta	471	0	68%	75.93%	*Plectosphaerella cucumerina*	KAH7369385.1

75) *Verticillium longisporum* contig_ CVQH01000001, VRPKS-I-19 cluster

Gene name	Size	E value	% Coverage	% identity	Top blast hit	Accession number of top blast hit
Acetylxylan esterase 2	283	6.00E-90	83%	58.92%	*Colletotrichum siamense*	KAF4811263.1
hypothetical protein VdG1_08811	633	2.00E-14	25%	100.00%	*Verticillium dahliae VDG1*	KAF3352645.1
vacuolar protein sorting-associated protein	770	0.00E+00	98%	71.80%	*Plectosphaerella plurivora*	KAH6662429.1
cutinase-2	224	3.00E-132	92%	89.37%	*Plectosphaerella cucumerina*	KAH7368196.1
alpha/beta-hydrolase	249	6.00E-73	97%	52.67%	*Zopfia rhizophila CBS 207.26*	KAF2180147.1
hypothetical protein HYQ44_014562	197	7.00E-140	100%	100.00%	*Verticillium longisporum*	KAG7105862.1
putative transporter C1683.12-like protein 11	488	0	99%	76.39%	*Colletotrichum chlorophyti*	OLN87319.1

Fungal specific transcription factor, putative	639	0	100%	66.10%	*Plectosphaerella plurivora*	KAH6669659.1
L-threonate dehydrogenase like protein	1134	0	99%	72.55%	*Colletotrichum graminicola M1.001*	XP_008095607.1
hypothetical protein J1614_005793	289	1.00E-116	90%	64.12%	*Leptosphaeria biglobosa*	KAH9873395.1
Type I Iterative PKS	2291	0.00E+00	99%	78.27%	*Leptosphaeria biglobosa*	KAH9873394.1
drug resistance protein	576	0.00E+00	97%	71.48%	*Plectosphaerella cucumerina*	KAH7347641.1
thioredoxin-like protein	162	1.00E-79	98%	71.88%	*Hypoxylon sp. FL0890*	KAI0834638.1
exopolygalacturonase	470	0	98%	69.44%	*Monosporascus sp. mg162*	RYP54678.1
Obg-like ATPase 1	464	0.00E+00	90%	84.73%	*Colletotrichum gloeosporioides*	XP_045260146.1
Type I Iterative PKS	71	9.00E-17	97%	85.51%	*Leptosphaeria biglobosa*	KAH9873394.1
drug resistance protein	570	0.00E+00	95%	69.82%	*Plectosphaerella cucumerina*	KAH7347641.1
exopolygalacturonase	617	0.00E+00	74%	68.98%	*Monosporascus sp. mg162*	RYP54678.1
Obg-like ATPase	394	0.00E+00	100%	88.58%	*Claviceps africana*	KAG5929667.1
DeSI-like protein sdu1	259	9.00E-127	97%	74.21%	*Colletotrichum chlorophyti*	OLN97068.1
putative 37S ribosomal protein S18, mitochondrial	248	2.00E-91	100%	57.26%	*Colletotrichum chlorophyti*	OLN97067.1
TIM44 subunit of mitochondria import inner membrane translocase	298	7.00E-54	75%	67.69%	*Sodiomyces alkalinus F11*	XP_028468965.1

76) *Verticillium longisporum* contig_ CVQH01024750, VRPKS-I-26 cluster

Gene name	Size	E value	% Coverage	% identity	Top blast hit	Accession number of top blast hit
hypothetical protein SODALDRAFT_326753	68	6.00E-18	80%	70.91%	*Sodiomyces alkalinus F11*	XP_028470403.1
hypothetical protein GQ607_002185	319	1.00E-09	30%	65.00%	*Colletotrichum asianum*	KAF0330781.1
peptidase family M28	373	0	91%	81.34%	*Dactylonectria estremocensis*	KAH7144232.1
retinol dehydrogenase	312	1.00E-169	100%	76.60%	*Plectosphaerella plurivora*	KAH6675343.1
TTL domain-containing protein	470	0	100%	69.98%	*Coniochaeta sp. 2T2.1*	KAB5518934.1
putative polyketide synthase	1260	0.00%	99%	63.19%	*Whalleya microplaca*	KAI1073750.1
hypothetical protein B0T11DRAFT_318355	156	5.00E-40	98%	60.98%	*Plectosphaerella cucumerina*	KAH7362742.1
Retinoic acid induced 16-like protein-domain-containing protein	1268	0	78%	71.54%	*Plectosphaerella cucumerina*	KAH7362743.1
GMC oxidoreductase family protein Mala s like	587	0	92%	84.53%	*Plectosphaerella cucumerina*	KAH7368263.1
hypothetical protein F5X68DRAFT_158933	365	0	98%	81.37%	*Plectosphaerella plurivora*	KAH6670884.1
hypothetical protein CMUS01_12901	577	0	92%	87.19%	*Colletotrichum musicola*	KAF6813152.1

peptidase family C25-domain-containing protein	989	0	100%	54.44%	*Paraphoma chrysanthemicola*	KAH7091334.1
hypothetical protein F5X68DRAFT_228683	77	3.00E-17	62%	77.55%	*Plectosphaerella plurivora*	KAH6692307.1
ankyrin repeat-containing domain protein	778	3.00E-121	96%	35.84%	*Plectosphaerella plurivora*	KAH6670890.1

77) *Verticillium longisporum* contig_ CVQH01024750, VNRPKS-I-1 cluster

Gene name	Size	E value	% Coverage	% identity	Top blast hit	Accession number of top blast hit
oxidoreductase	326	0	99%	83.69%	*Colletotrichum truncatum*	XP_036586400.1
Conidial pigment polyketide synthase PfmaE like protein	2164	0	100%	75.58%	*Colletotrichum karsti*	XP_038751444.1
laccase-like multicopper oxidase	545	0	96%	64.60%	*Plectosphaerella cucumerina*	KAH7358155.1
hypothetical protein BN1723_013595	165	1.00E-107	90%	100.00%	*Verticillium longisporum*	CRK25564.1
hypothetical protein SODALDRAFT_331196	364	2.00E-137	99%	59.34%	*Sodiomyces alkalinus F11*	XP_028469270.1
L-2,4-diaminobutyrate decarboxylase	479	5.00E-173	96%	60.76%	*Plectosphaerella cucumerina*	KAH7358159.1
putative kinetochore protein	91	8.00E-24	100%	63.74%	*Phaeoacremonium minimum UCRPA7*	XP_007911737.1
versicolorin reductase	284	1.00E-158	100%	81.82%	*Plectosphaerella cucumerina*	KAH7358161.1
complex I intermediate-associated protein	483	0	99%	73.81%	*Sodiomyces alkalinus F11*	XP_028469267.1

78) *Verticillium longisporum* contig_ CVQH01006668, VNRPKS-I-1 cluster

Gene name	Size	E value	% Coverage	% identity	Top blast hit	Accession number of top blast hit
hypothetical protein SODALDRAFT_82466	612	4.00E-77	86%	36.02%	*Sodiomyces alkalinus F11*	XP_028462476.1
Elongation factor 1-alpha like protein	359	0	95%	93.90%	*Diaporthaceae sp. PMI_573*	KAH8762000.1
versicolorin reductase	284	6.00E-162	100%	82.52%	*Plectosphaerella cucumerina*	KAH7358161.1
polyketide synthase	246	2.00E-54	67%	53.94%	*Sodiomyces alkalinus F11*	XP_028469272.1
pyridoxal-dependent decarboxylase	474	1.00E-163	98%	58.94%	*Plectosphaerella plurivora*	KAH6697039.1
hypothetical protein SODALDRAFT_331196	390	3.00E-153	99%	64.86%	*Sodiomyces alkalinus F11*	XP_028469270.1
hypothetical protein BN1708_002822	223	4.00E-149	90%	99.01%	*Verticillium longisporum*	CRK15632.1
iron transport multicopper oxidase fetC	565	0	92%	64.94%	*Colletotrichum liriopes*	GKT45119.1
Conidial pigment polyketide synthase PfmaE like protein	2189	0	100%	76.49%	*Colletotrichum karsti*	XP_038751444.1

oxidoreductase	326	0	99%	83.38%	*Colletotrichum truncatum*	XP_036586400.1
transcription factor	286	3.00E-53	44%	71.43%	*Colletotrichum higginsianum*	CCF47183.1
hypothetical protein DL767_005224	729	0	80%	56.09%	*Monosporascus sp. MG133*	RYP32460.1
glycosyl transferase family protein	307	9.00E-151	100%	69.94%	*Sodiomyces alkalinus* F11	XP_028469283.1

79) *Verticillium longisporum* contig_ CVQH01006891, VRPKS-I-11 cluster

Gene name	Size	E value	% Coverage	% identity	Top blast hit	Accession number of top blast hit
mg2+ transporter	1009	0	82%	38.82%	*Colletotrichum asianum*	KAF0332070.1
metallo-beta-lactamase family protein	227	1.00E-88	88%	61.23%	*Plectosphaerella cucumerina*	KAH7359326.1
Aldo/keto reductase	323	4.00E-149	97%	65.08%	*Ilyonectria destructans*	KAH6999345.1
AMP-binding enzyme domain-containing protein	1180	0	99%	77.09%	*Hirsutella rhossiliensis*	XP_044717051.1
polyamine transporter 1	587	0	98%	74.18%	*Plectosphaerella plurivora*	KAH6687403.1
lactose permease	525	0	95%	81.98%	*Fusarium decemcellulare*	KAF4989538.1
polyketide synthase	2672	0	80%	44.24%	*Acremonium chrysogenum ATCC 11550*	KFH45364.1
putative polyketide synthase	402	0	99%	70.64%	*Acremonium chrysogenum ATCC 11550*	KFH45364.1
uncharacterized protein BCR38DRAFT_489105	206	1.00E-61	87%	52.72%	*Pseudomassariella vexata*	XP_040711558.1
short-chain dehydrogenase	288	2.00E-99	100%	64.93%	*Plectosphaerella cucumerina*	KAH7347401.1
putative efflux pump antibiotic resistance protein	1071	0	48%	60.42%	*Hypoxylon sp. FL0890*	KAI0837754.1
kinase domain-containing protein	394	0	92%	78.96%	*Epichloe festucae Fl1*	QPH06552.1
hypothetical protein BN1708_011581	68	3.00E-26	80%	100.00%	*Verticillium longisporum*	CRK15839.1
ankyrin repeat-containing domain protein	334	9.00E-61	66%	49.33%	*Plectosphaerella cucumerina*	KAH7375435.1
short-chain dehydrogenase	250	2.00E-111	100%	63.49%	*Plectosphaerella plurivora*	KAH6692198.1

80) *Verticillium longisporum* contig_ CVQH01025950, VRPKS-I-8 cluster

Gene name	Size	E value	% Coverage	% identity	Top blast hit	Accession number of top blast hit
polyketide synthase	2506	0	98%	69.55%	*Fusarium* sp. NRRL 66182	KAF5026352.1
alcohol dehydrogenase like domain-containing protein	362	0	97%	79.60%	*Stemphylium lycopersici*	KNG48002.1
Enoyl reductase	324	0	99	64.15	*Fusarium mexicanum*	RAR12984
Hydrolase	256	0	100	85.12	*Stemphylium lycopersici*	KNG48003
Fungal ZN(2) Cys(6) cluster domain containing protein/Fujikurins biosynthesis transcription factor	359	2e-158	99	57.53	*Stemphylium lycopersici*	KNG48004
MFS gliotoxin efflux transporter	473	0	99	69.57	*Ascochyta lentis*	KAF9701447

Cytochrome monooxygenase	515	0	93	73.65	*Fusarium sp.*	KAF5026356
Hypothetical protein	229	3e-123	62	54.38	*Cadophora malorum*	KAG4414469
Hypothetical protein	96	2e-65	98	54.37	*Stylonectria norvegica*	KAF7562826
Hypothetical protein	240	2e-149	100	39.38	*Stachybotrys chartarum*	KEY69576
Dehydrogenase	828	1e-183	84	46.63	*Ustilaginoidea virens*	QUC21319
Hypothetical protein	1314	0	99	44.89	*Fusarium mundagurra*	KAF5705177

81) *Verticillium longisporum* contig_CVQH01027305, VRPKS-I-1 cluster

Gene name	Size	E value	% Coverage	% identity	Top blast hit	Accession number of top blast hit
dihydroorotate dehydrogenase	399	0	100%	75.31%	*Sodiomyces alkalinus F11*	XP_028470759.1
Protein SYM1	267	4.00E-121	82%	77.68%	*Colletotrichum viniferum*	KAF4911848.1
hypothetical protein F5X68DRAFT_40851	716	3.00E-175	95%	58.38%	*Plectosphaerella plurivora*	KAH6673933.1
WW domain-containing oxidoreductase	332	1.00E-147	100%	76.51%	*Colletotrichum sidae*	TEA18735.1
hypothetical protein IFM61606_09584	154	2.00E-64	100%	70.13%	*Aspergillus udagawae*	GFF25928.1
integral membrane protein	310	3.00E-127	96%	68.56%	*Plectosphaerella plurivora*	KAH6672729.1
polyketide synthase	4107	0	99%	42.69%	*Halenospora varia*	KAH6673321.1
hypothetical protein V490_02118	467	5.00E-115	99%	44.23%	*Pseudogymnoascus sp. VKM F-3557*	KFX98761.1
hypothetical protein CDV55_104319	361	9.00E-99	94%	49.42%	*Aspergillus turcosus*	RHZ60657.1
hypothetical protein DBV05_g8002	195	2.00E-42	96%	38.62%	*Lasiodiplodia theobromae*	KAB2573329.1
hypothetical protein BX600DRAFT_462019	413	1.00E-79	61%	52.55%	*Xylariales sp. PMI_506*	KAH8666574.1
major facilitator superfamily domain-containing protein	585	0	90%	67.49%	*Xylariales sp. PMI_506*	KAH8666575.1
hypothetical protein Forpe1208_v005775	168	8.00E-40	96%	53.09%	*Fusarium oxysporum f. sp. rapae*	KAG7415270.1

82) *Verticillium longisporum* contig_CVQH01027305, VRPKS-I-23 cluster

Gene name	Size	E value	% Coverage	% identity	Top blast hit	Accession number of top blast hit
hypothetical protein SPI_04859	280	6.00E-134	95%	71.27%	*Sporothrix insectorum RCEF 264*	OAA62000.1
benzoate 4-monooxygenase cytochrome P450	268	3.00E-91	97%	52.43%	*Colletotrichum higginsianum*	TIC94206.1
Sterol 3-beta-glucosyltransferase	1458	0	94%	70.56%	*Colletotrichum siamense*	XP_036496831.1
WD repeat-containing protein	565	0	99%	72.74%	*Plectosphaerella cucumerina*	KAH7349692.1
hypothetical protein B0T11DRAFT_289698	337	2.00E-63	94%	44.78%	*Plectosphaerella cucumerina*	KAH7349693.1

pectate lyase	243	2.00E-154	100%	92.18%	*Plectosphaerella cucumerina*	KAH7359289.1
putative transporter	533	0	93%	81.53%	*Colletotrichum chlorophyti*	OLN93090.1
phenolpthiocerol synthesis polyketide synthase ppsA	1092	0	99%	47.21%	*Sodiomyces alkalinus F11*	XP_028463040.1
hypothetical protein	772	0	98%	57.50%	*Sodiomyces alkalinus F11*	XP_028463042.1
serine 3-dehydrogenase	283	1.00E-155	98%	74.64%	*Akanthomyces lecanii RCEF 1005*	OAA81050.1
clock controlled protein	413	1.00E-150	97%	65.27%	*Colletotrichum tofieldiae*	GKT84128.1
Clock-controlled protein 8	530	4.00E-85	59%	66.80%	*Colletotrichum tanaceti*	TKW48619.1
hypothetical protein SODALDRAFT_329702	432	8.00E-31	80%	64.19%	*Sodiomyces alkalinus F11*	XP_028462398.1
hypothetical protein SODALDRAFT_329702	354	6.00E-54	92%	52.25%	*Sodiomyces alkalinus F11*	XP_028462398.1
D-isomer specific 2-hydroxyacid dehydrogenase	387	2.00E-171	88%	73.41%	*Plectosphaerella cucumerina*	KAH7368637.1

83) *Verticillium longisporum* contig_CVQH01004113, PKS-III cluster

Gene name	Size	E value	% Coverage	% identity	Top blast hit	Accession number of top blast hit
NADH-ubiquinone oxidoreductase 30.4 kDa subunit	440	1.00E-99	95%	51.95%	*Plectosphaerella plurivora*	KAH6681192.1
short chain dehydrogenase	301	1.00E-150	99%	76.17%	*Sodiomyces alkalinus F11*	XP_028470310.1
centrin-3	190	4.00E-96	98%	69.68%	*Sodiomyces alkalinus F11*	XP_028470317.1
Iron-sulfur protein IND1	299	3.00E-158	98%	78.91%	*Colletotrichum sidae*	TEA13372.1
Splicing factor spf30	310	3.00E-113	100%	71.61%	*Colletotrichum truncatum*	XP_036586172.1
hypothetical protein B0T11DRAFT_249550	384	4.00E-84	98%	53.98%	*Plectosphaerella cucumerina*	KAH7375296.1
putative mitochondrial 2-oxoglutarate/malate carrier protein	335	1.00E-170	100%	86.27%	*Colletotrichum chlorophyti*	OLN95994.1
PAB-dependent poly(A)-specific ribonuclease subunit PAN2	340	0	100%	82.65%	*Sodiomyces alkalinus F11*	XP_028470311.1
chalcone and stilbene synthase domain-containing protein	479	0	75%	77.07%	*Plectosphaerella cucumerina*	KAH7375301.1
TRAUB-domain-containing protein	445	2.00E-129	66%	72.00%	*Sodiomyces alkalinus F11*	XP_028470322.1
putative stilbene synthase 2	432	0	100%	72.85%	*Colletotrichum chlorophyti*	OLN95986.1
TRAUB-domain-containing protein	596	0	100%	63.48%	*Sodiomyces alkalinus F11*	XP_028470322.1
fungal specific transcription factor domain-containing protein	609	0	99%	72.43%	*Colletotrichum incanum*	KZL80546.1

Feruloyl esterase B	294	1.00E-145	91%	72.49%	*Colletotrichum orbiculare MAFF 240422*	TDZ25527.1
oxidoreductase	451	0	99%	64.37%	*Plectosphaerella plurivora*	KAH6681205.1
Acetyltransferase	244	4.00E-112	81%	68.80%	*Colletotrichum higginsianum IMI 349063*	XP_018159255.1
carbonic anhydrase	271	3.00E-142	98%	70.79%	*Colletotrichum simmondsii*	KXH49803.1
Glutathione S-transferase	275	6.00E-128	100%	67.63%	*Lasiodiplodia theobromae*	KAF9634029.1

84) *Verticillium nonalfalfae* contig_NW_021167278, VRPKS-I-22 cluster

Gene name	Size	E value	% Coverage	% identity	Top blast hit	Accession number of top blast hit
RNA polymerase II transcription elongation factor	361	6.00E-109	100%	64.75%	Colletotrichum tofieldiae	KZL76908.1
COQ7-domain-containing protein	244	3.00E-121	76%	90.86%	Sodiomyces alkalinus F11	XP_028463306.1
phospholipase D1	1819	0	99%	72.51%	Sodiomyces alkalinus F11	XP_028463305.1
tubulin/FtsZ family protein	461	0	100%	96.10%	Sodiomyces alkalinus F11	XP_028463304.1
uncharacterized protein BCR38DRAFT_425087	90	1.00E-37	97%	75.00%	Pseudomassariella vexata	XP_040719305.1
hypothetical protein SODALDRAFT_68083	883	4.00E-11	13%	36.30%	Sodiomyces alkalinus F11	XP_028463302.1
P-loop containing nucleoside triphosphate hydrolase protein	251	6.00E-117	100%	67.73%	Plectosphaerella cucumerina	KAH7357817.1
NAD-binding Rossmann fold oxidoreductase family protein	332	2.00E-124	99%	53.31%	*Fusarium* sarcochroum	KAF4960431.1
major facilitator superfamily transporter	588	0	89%	76.42%	Colletotrichum graminicola M1.001	XP_008093595.1
FAD-dependent monooxygenase kojA like protein	389	0	93%	71.98%	Bimuria novae-zelandiae CBS 107.79	KAF1970089.1
DUF341 domain-containing protein	218	2.00E-109	100%	68.81%	*Fusarium* nygamai	PNP83713.1
UDP-glucuronosyltransferase 2B13 like protein	553	0	98%	61.61%	*Fusarium* sp. NRRL 66182	KAF5020447.1
polyketide synthase	2635	0	99%	70.41%	*Fusarium* mexicanum	KAF5539484.1
sulfate transporter family-domain-containing protein	1066	0	75%	54.28%	Bisporella sp. PMI_857	KAH8586595.1
hypothetical protein FLONG3_8802	341	2.00E-21	84%	26.17%	*Fusarium* longipes	RGP66606.1
hypothetical protein CNYM01_04222	286	4.00E-64	51%	79.45%	Colletotrichum nymphaeae SA-01	KXH64953.1
MSF1 protein	192	5.00E-115	91%	88.07%	Sodiomyces alkalinus F11	XP_028463299.1
methionine aminopeptidase 2-like protein	441	0	100%	84.75%	Plectosphaerella plurivora	KAH6686947.1

Peroxiredoxin-like 2C	412	8.00E-163	82%	64.81%	Plectosphaerella plurivora	KAH6686952.1
uncharacterized protein D7B24_007046	265	3.00E-170	100%	100.00%	Verticillium nonalfalfae	XP_028494840.1

85) *Verticillium nonalfalfae* contig_ NW_021167382, VRPKS-I-17 cluster

Gene name	Size	E value	% Coverage	% identity	Top blast hit	Accession number of top blast hit
hypothetical protein G7Z17_g6650	164	4.00E-34	78%	63.85%	*Cylindrodendrum hubeiense*	KAF7549055.1
Golgi to ER traffic protein 4 like	334	3.00E-168	100%	79.10%	*Colletotrichum gloeosporioides Cg-14*	EQB49343.1
mitochondrial import receptor subunit tom-40	352	0	100%	83.90%	*Sodiomyces alkalinus F11*	XP_028466992.1
6-phosphofructo-2-kinase	574	0	100%	84.54%	*Plectosphaerella cucumerina*	KAH7363100.1
ubiquitin C-terminal hydrolase	823	0	100%	65.64%	*Plectosphaerella cucumerina*	KAH7363099.1
54S ribosomal protein L7	352	0	99%	73.14%	*Sodiomyces alkalinus F11*	XP_028466988.1
hsp70-like protein	209	1.00E-94	98%	68.25%	*Plectosphaerella plurivora*	KAH6686283.1
Actin	375	0	100%	100.00%	*Tolypocladium paradoxum*	POR36442.1
ABC transporter	1573	0	83%	68.64%	*Colletotrichum nymphaeae SA-01*	KXH49589.1
uncharacterized protein CORC01_10575	255	2.00E-150	100%	78.91%	*Colletotrichum orchidophilum*	XP_022471281.1
Reducing polyketide synthase PKS2	2419	0	93%	70.29%	*Colletotrichum chlorophyti*	OLN86478.1
elongator protein 2 (WD domain-containing protein)	828	0	99%	65.79%	*Colletotrichum truncatum*	XP_036582858.1
Ubiquitin- modifier 1	104	2.00E-56	99%	79.61%	*Claviceps pusilla*	KAG6012378.1
Asparagine-rich zinc finger protein AZF1	438	2.00E-116	89%	50.98%	*Colletotrichum trifolii*	TDZ74112.1

86) *Verticillium nonalfalfae* contig_ NW_021167282, VRPKS-I-19 cluster

Gene name	Size	E value	% Coverage	% identity	Top blast hit	Accession number of top blast hit
siderochrome-iron transporter MirB	422	0	100%	69.43%	*Ilyonectria sp. MPI-CAGE-AT-0026*	KAH6972343.1
Extracellular serine-rich protein	229	1.00E-60	81%	70.90%	*Colletotrichum higginsianum IMI 349063*	XP_018161331.1
hypothetical protein SODALDRAFT_333514	77	2.00E-21	97%	55.13%	*Sodiomyces alkalinus F11*	XP_028465568.1
hypothetical protein ACRE_064810	288	5.00E-78	95%	47.45%	*Acremonium chrysogenum ATCC 11550*	KFH42767.1
N-glycosidase like protein	192	2.00E-51	83%	61.49%	*Plectosphaerella cucumerina*	KAH7347667.1
TIM44 subunit of mitochondria import inner membrane translocase	543	0	87%	78.83%	*Sodiomyces alkalinus F11*	XP_028468965.1
hypothetical protein CFIO01_06157	248	5.00E-95	100%	57.66%	*Colletotrichum fioriniae PJ7*	EXF75199.1

DeSI-like protein sdu1	257	9.00E-129	97%	74.80%	*Colletotrichum chlorophyti*	OLN97068.1
Obg-like ATPase	394	0	100%	88.58%	*Claviceps africana*	KAG5929667.1
exopolygalacturonase B like protein	470	0	98%	69.16%	*Monosporascus sp. MG133*	RYP36431.1
thioredoxin	162	3.00E-78	98%	70.62%	*Colletotrichum orchidophilum*	XP_022480245.1
drug resistance protein	576	0	97%	71.08%	*Plectosphaerella cucumerina*	KAH7347641.1
hypothetical protein J1614_005792	2176	0	99%	78.94%	*Leptosphaeria biglobosa*	KAH9873394.1
hypothetical protein J1614_005793	289	2.00E-117	90%	64.50%	*Leptosphaeria biglobosa*	KAH9873395.1
Ketose-bisphosphate aldolase class-ii family protein	1096	0	99%	70.90%	*Colletotrichum higginsianum IMI 349063*	XP_018154864.1
hypothetical protein F5X68DRAFT_50483	626	0	100%	67.72%	*Plectosphaerella plurivora*	KAH6669659.1
putative transporter C1683.12-like protein 11	488	0	99%	76.59%	*Colletotrichum chlorophyti*	OLN87319.1

87) *Verticillium nonalfalfae* contig_ NW_021167434, VRPKS-I-23 cluster

Gene name	Size	E value	% Coverage	% identity	Top blast hit	Accession number of top blast hit
virulence sensor protein bvgS	1397	0	98%	65.63%	*Plectosphaerella cucumerina*	KAH7358170.1
drug resistance transporter	454	0	98%	68.53%	*Plectosphaerella cucumerina*	KAH7358948.1
phenolpthiocerol synthesis polyketide synthase ppsA	2379	0	98%	50.04%	*Sodiomyces alkalinus F11*	XP_028462492.1
hypothetical protein SODALDRAFT_82945	363	1.00E-90	100%	47.11%	*Sodiomyces alkalinus F11*	XP_028462491.1
hypothetical protein F5X68DRAFT_4497	721	0	93%	64.37%	*Plectosphaerella plurivora*	KAH6697434.1
hypothetical protein F5X68DRAFT_257601	258	3.00E-11	88%	36.61%	*Plectosphaerella plurivora*	KAH6697435.1

88) *Verticillium nonalfalfae* contig_ NW_021167247, VRPKS-I-26 cluster

Gene name	Size	E value	% Coverage	% identity	Top blast hit	Accession number of top blast hit
Short chain dehydrogenase citE	292	5.00E-111	99%	57.73%	*Colletotrichum siamense*	KAF4822533.1
ankyrin repeat-containing domain protein	1141	7.00E-110	60%	35.52%	*Plectosphaerella plurivora*	KAH6670890.1
C25 family cysteine peptidase	1017	0	99%	55.14%	*Paraphoma chrysanthemicola*	KAH7091334.1
hypothetical protein CMUS01_12901	577	0	92%	87.57%	*Colletotrichum musicola*	KAF6813152.1
hypothetical protein F5X68DRAFT_158933	365	0	98%	81.64%	*Plectosphaerella plurivora*	KAH6670884.1
choline dehydrogenase	616	0	95%	82.57%	*Plectosphaerella cucumerina*	KAH7368263.1
Retinoic acid induced 16-like protein-domain-containing protein	1277	0	77%	72.01%	*Plectosphaerella cucumerina*	KAH7362743.1
hypothetical protein B0T11DRAFT_318355	162	5.00E-48	98%	65.85%	*Plectosphaerella cucumerina*	KAH7362742.1

putative polyketide synthase	1466	0	97%	54.85%	*Whalleya microplaca*	KAI1073750.1
TTL domain-containing protein	472	0	99%	69.15%	*Coniochaeta sp. 2T2.1*	KAB5518934.1
peptidase family M28	609	2.00E-176	52%	76.09%	*Dactylonectria estremocensis*	KAH7144232.1
hypothetical protein CGCVW01_v010845	317	4.00E-10	30%	64.00%	*Colletotrichum viniferum*	KAF4913593.1
hypothetical protein SODALDRAFT_326753	200	1.00E-87	94%	57.52%	*Sodiomyces alkalinus F11*	XP_028470403.1
hypothetical protein CI238_07639	126	0.004	26%	100.00%	*Colletotrichum incanum*	KZL63877.1
ubiquitin carboxyl-terminal hydrolase	397	6.00E-127	84%	64.41%	*Plectosphaerella cucumerina*	KAH7369286.1

89) *Verticillium nonalfalfae* contig_ NW_021167324, VNRPKS-I-1 cluster

Gene name	Size	E value	% Coverage	% identity	Top blast hit	Accession number of top blast hit
oxidoreductase	326	0.00E+00	99%	84.62%	*Colletotrichum truncatum*	XP_036586400.1
Conidial yellow pigment biosynthesis polyketide synthase	2192	0.00E+00	100%	75.72%	*Colletotrichum gloeosporioides*	XP_045268234.1
iron transport multicopper oxidase fetC	549	0	94%	65.80%	*Colletotrichum liriopes*	GKT45119.1
uncharacterized protein D7B24_009601	201	9.00E-137	99%	94.47%	*Verticillium nonalfalfae*	XP_028492744.1
duf92 domain protein	390	1.00E-145	99%	64.86%	*Sodiomyces alkalinus F11*	XP_028469270.1
pyridoxal-dependent decarboxylase	533	0	98%	59.77%	*Plectosphaerella plurivora*	KAH6697039.1
putative kinetochore protein	91	8.00E-24	100%	63.74%	*Phaeoacremonium minimum UCRPA7*	XP_007911737.1
complex I intermediate-associated protein	710	0.00E+00	99%	74.30%	*Sodiomyces alkalinus F11*	XP_028469267.1
ribosomal protein S18	162	9.00E-73	95%	69.48%	*Sodiomyces alkalinus F11*	XP_028469266.1

90) *Verticillium nonalfalfae* contig_ NW_021167257, VNRPKS-I-3 cluster

Gene name	Size	E value	% Coverage	% identity	Top blast hit	Accession number of top blast hit
dibenzothiophene desulfurization enzyme C	428	0	100%	81.02%	*Ilyonectria destructans*	KAH7000608.1
Acetate transporter protein	204	2.00E-110	97%	86.36%	*Ilyonectria destructans*	KAH7000609.1
FAD binding domain-containing protein	511	0	95%	69.75%	*Sodiomyces alkalinus F11*	XP_028469411.1
alpha/beta-hydrolase	381	2.00E-160	99%	58.96%	*Sodiomyces alkalinus F11*	XP_028463045.1
FAD/NAD(P)-binding domain-containing protein	759	0	99%	68.64%	*Sodiomyces alkalinus F11*	XP_028463046.1
S-adenosyl-L-methionine-dependent methyltransferase	486	0	100%	64.42%	*Sodiomyces alkalinus F11*	XP_028463047.1
hypothetical protein SODALDRAFT_329430	411	2.00E-100	100%	45.08%	*Sodiomyces alkalinus F11*	XP_028463048.1

serine 3-dehydrogenase	283	1.00E-154	98%	73.93%	*Akanthomyces lecanii RCEF 1005*	OAA81050.1
acetyl-CoA synthetase-like protein	772	0	98%	56.58%	*Sodiomyces alkalinus F11*	XP_028463042.1
phenolpthiocerol synthesis polyketide synthase ppsA	423	4.00E-131	87%	55.50%	*Sodiomyces alkalinus F11*	XP_028463040.1
putative transporter C460.03-like protein 2	515	0.00E+00	91%	86.53%	*Colletotrichum chlorophyti*	OLN93090.1
pectate lyase	243	7.00E-154	100%	92.59%	*Plectosphaerella cucumerina*	KAH7359289.1
hypothetical protein B0T11DRAFT_289698	403	9.00E-99	99%	45.58%	*Plectosphaerella cucumerina*	KAH7349693.1
WD repeat-containing protein	565	0	99%	72.57%	*Plectosphaerella cucumerina*	KAH7349692.1
Sterol 3-beta-glucosyltransferase	1453	0.00E+00	94%	69.89%	*Colletotrichum siamense*	KAF4841498.1
Tryprostatin B 6-hydroxylase	276	6.00E-93	97%	52.99%	*Colletotrichum higginsianum*	TIC94206.1
NYN domain-containing protein	200	2.00E-91	94%	73.54%	*Hypomontagnella submonticulosa*	KAI2617645.1

91) *Verticillium nonalfalfae* contig_ NW_021167286, PKS-III cluster

**Gene name**	**Size**	**E value**	**% Coverage**	**% identity**	**Top blast hit**	**Accession number of top blast hit**
putative ATP-dependent helicase	1116	0	51%	75.48%	*Colletotrichum chlorophyti*	OLN95968.1
uncharacterized protein GLRG_10587	489	1.00E-71	67%	43.13%	*Colletotrichum graminicola M1.001*	XP_008099463.1
NADH-ubiquinone oxidoreductase 30.4 kDa subunit	496	3.00E-52	83%	50.00%	*Plectosphaerella cucumerina*	KAH7375303.1
short chain dehydrogenase	312	1.00E-138	99%	72.82%	*Sodiomyces alkalinus F11*	XP_028470310.1
centrin-3	191	7.00E-93	98%	69.31%	*Sodiomyces alkalinus F11*	XP_028470317.1
Iron-sulfur protein IND1	296	3.00E-177	99%	79.59%	*Colletotrichum sidae*	TEA13372.1
Splicing factor spf30	309	8.00E-118	100%	71.20%	*Colletotrichum truncatum*	XP_036586172.1
hypothetical protein B0T11DRAFT_249550	646	1.00E-149	96%	58.02%	*Plectosphaerella cucumerina*	KAH7375296.1
putative mitochondrial 2-oxoglutarate/malate carrier protein	335	1.00E-170	100%	86.27%	*Colletotrichum chlorophyti*	OLN95994.1
PAB-dependent poly(A)-specific ribonuclease subunit PAN2	1965	0	55%	77.55%	*Sodiomyces alkalinus F11*	XP_028470311.1
putative stilbene synthase 2	432	0	100%	72.91%	*Colletotrichum chlorophyti*	OLN95986.1
TRAUB-domain-containing protein	594	2.00E-145	85%	72.05%	*Sodiomyces alkalinus F11*	XP_028470322.1
fungal specific transcription factor domain-containing protein	608	0	99%	72.39%	*Colletotrichum incanum*	KZL80546.1
Feruloyl esterase B	294	3.00E-145	91%	72.12%	*Colletotrichum orbiculare MAFF 240422*	TDZ25527.1

oxidoreductase	451	0	99%	64.37%	*Plectosphaerella plurivora*	KAH6681205.1
Acetyltransferase	278	8.00E-145	84%	82.55%	*Colletotrichum higginsianum IMI 349063*	XP_018159255.1
carbonic anhydrase	271	6.00E-145	98%	71.91%	*Colletotrichum simmondsii*	KXH49803.1

92) *Verticillium nonalfalfae* contig_ NW_021167275, VRPKS-I-9 and VRPKS-I-11 clusters

**Gene name**	**Size**	**E value**	**% Coverage**	**% identity**	**Top blast hit**	**Accession number of top blast hit**
X-Pro dipeptidyl-peptidase protein	300	1.00E-138	100%	68.44%	*Diaporthaceae sp. PMI_573*	KAH8742789.1
uncharacterized protein D7B24_006854	466	0	100%	100.00%	*Verticillium nonalfalfae*	XP_028494997.1
aflatoxin biosynthesis ketoreductase nor-1	250	6.00E-111	100%	63.49%	*Plectosphaerella plurivora*	KAH6692198.1
ankyrin repeat-containing domain protein	338	3.00E-57	47%	59.39%	*Plectosphaerella cucumerina*	KAH7375435.1
hypothetical protein C2857_005093	362	0	98%	80.79%	*Epichloe festucae Fl1*	QPH06552.1
putative efflux pump antibiotic resistance protein	550	0	94%	65.77%	*Hypoxylon sp. FL0890*	KAI0837754.1
short-chain dehydrogenase	287	1.00E-107	99%	63.51%	*Plectosphaerella cucumerina*	KAH7347401.1
uncharacterized protein BCR38DRAFT_489105	204	9.00E-61	87%	52.49%	*Pseudomassariella vexata*	XP_040711558.1
putative polyketide synthase	3858	0	100%	47.99%	*Acremonium chrysogenum ATCC 11550*	KFH45364.1
fungal-specific transcription factor domain-containing protein	750	0	82%	61.79%	*Plectosphaerella cucumerina*	KAH7347400.1
hypothetical protein VSDG_07360	365	9.00E-42	84%	34.60%	*Valsa sordida*	ROV93079.1
hypothetical protein B0T11DRAFT_326518	524	3.00E-36	54%	33.45%	*Plectosphaerella cucumerina*	KAH7368326.1
putative Hybrid PKS-NRPS biosynthetic cluster	3856	0	99%	70.68%	*Hirsutella rhossiliensis*	XP_044717051.1
HpcH/HpaI aldolase	284	3.00E-150	98%	79.72%	*Colletotrichum higginsianum IMI 349063*	XP_018154477.1
major facilitator superfamily domain-containing protein	495	0	97%	79.25%	*Plectosphaerella cucumerina*	KAH7361596.1
D-isomer specific 2-hydroxyacid dehydrogenase	304	4.00E-162	98%	76.33%	*Ilyonectria sp. MPI-CAGE-AT-0026*	KAH6989082.1
hypothetical protein BKA56DRAFT_641098	426	4.00E-119	97%	45.33%	*Ilyonectria sp. MPI-CAGE-AT-0026*	KAH6989083.1

93) *Verticillium nubilum* contig_ NMXI01000001, VRPKS-I-17 cluster

**Gene name**	**Size**	**E value**	**% Coverage**	**% identity**	**Top blast hit**	**Accession number of top blast hit**
hypothetical protein B0T11DRAFT_91086	133	7.00E-39	72%	69.70%	*Plectosphaerella cucumerina*	KAH7363103.1

duf410 domain-containing protein	331	4.00E-166	91%	82.24%	*Colletotrichum sojae*	KAF6819727.1
isoflavone reductase	290	2.00E-81	97%	44.55%	*Colletotrichum graminicola M1.*	XP_008091926.1
mitochondrial import receptor subunit tom-40	352	0	100%	83.05%	*Sodiomyces alkalinus F11*	XP_028466992.1
6-phosphofructo-2-kinase/fructose-2,6-bisphosphatase	528	0	100%	86.11%	*Colletotrichum plurivorum*	KAF6841603.1
ubiquitin C-terminal hydrolase	823	0	100%	66.63%	*Plectosphaerella cucumerina*	KAH7363099.1
54S ribosomal protein L7	352	0	99%	73.22%	*Sodiomyces alkalinus F11*	XP_028466988.1
nucleotide exchange factor Fes1	209	1.00E-94	99%	70.67%	*Sodiomyces alkalinus F11*	XP_028466987.1
Actin	375	0	100%	100.00%	*Tolypocladium paradoxum*	POR36442.1
ABC transporter	1485	0	87%	69.67%	*Colletotrichum incanum*	OHW91062.1
duf341 family	255	5.00E-150	100%	77.73%	*Colletotrichum sojae*	KAF6806434.1
Lovastatin diketide synthase LovF 16	2296	0	99%	70.16%	*Colletotrichum chlorophyti*	OLN86478.1
hypothetical protein SODALDRAFT_332602	113	8.00E-44	79%	76.67%	*Sodiomyces alkalinus F11*	XP_028466984.1
elongator protein 2 (WD domain-containing protein)	826	0	99%	66.27%	*Colletotrichum truncatum*	XP_036582858.1
Ubiquitin- modifier 1	89	1.00E-49	100%	83.15%	*Claviceps pusilla*	KAG6012378.1
hypothetical protein CGLO_15588	438	8.00E-120	88%	49.26%	*Colletotrichum gloeosporioides 14*	EQB45519.1
Mitochondrial FAD carrier protein FLX1	312	1.00E-147	99%	71.83%	*Colletotrichum higginsianum*	TIC98890.1

94) *Verticillium nubilum* contig_ NMXI01000076, VRPKS-I-10 cluster

Gene name	Size	E value	% Coverage	% identity	Top blast hit	Accession number of top blast hit
Proline oxidase	468	0	88%	62.26%	*Penicillium expansum*	XP_016597406.1
alpha/beta hydrolase fold family protein	601	0	84%	66.27%	*Colletotrichum tofieldiae*	KZL64421.1
hypothetical protein EDB80DRAFT_89890	418	0	81%	88.27%	*Ilyonectria destructans*	KAH7001356.1
shikimate/quinate 5-dehydrogenase	366	8.00E-130	99%	59.79%	*Colletotrichum higginsianum 349063*	XP_018164247.1
Proline utilization trans-activator like protein	689	0	95%	61.47%	*Plectosphaerella cucumerina*	KAH7375570.1
beta-ketoacyl synthase domain-containing pro	1703	0	99%	54.33%	*Coniochaeta sp. PMI_546*	KAH8906691.1
hypothetical protein F5X68DRAFT_47703	219	3.00E-51	88%	42.73%	*Plectosphaerella plurivora*	KAH6693919.1
hypothetical protein B0T11DRAFT_86728	594	0	99%	69.13%	*Plectosphaerella cucumerina*	KAH7362695.1
2OG-Fe oxygenase family protein	332	8.00E-157	99%	69.79%	*Colletotrichum incanum*	OHW94343.1
Uracil permease like protein	566	0	97%	76.90%	*Colletotrichum camelliae*	KAH0420306.1
d-isomer specific 2-hydroxyacid dehydrogen	314	0	100%	85.03%	*Fusarium albosuccineum*	KAF4467173.1
hypothetical protein BT63DRAFT_423135	323	4.00E-60	78%	69.46%	*Microthyrium microscopicum*	KAF2670841.1
Endoglucanase-4	295	9.00E-107	87%	57.85%	*Colletotrichum sidae*	TEA13904.1
epoxide hydrolase	405	3.00E-174	99%	61.19%	*Plectosphaerella plurivora*	KAH6662678.1

95) *Verticillium nubilum* contig_ NMXI01000091, VRPKS-I-15 cluster

Gene name	Size	E value	% Coverage	% identity	Top blast hit	Accession number of top blast hit
hypothetical protein M426DRAFT_316265	382	4.00E-121	92%	49.72%	*Hypoxylon sp. CI-4A*	OTB08973.1
hypothetical protein FDECE_625	488	0	98%	78.84%	*Fusarium decemcellulare*	KAF5013378.1

hypothetical protein CH63R_01711	269	4.00E-60	82%	48.46%	*Colletotrichum higginsianum 349063*	XP_018165048.1
dihydroxy-acid dehydratase	622	0	99%	82.88%	*Plectosphaerella cucumerina*	KAH7367439.1
putative salicylate hydroxylase	445	0	92%	78.35%	*Dactylonectria macrodidyma*	KAH7133940.1
Type I Iterative Polyketide synthase (PKS)	2578	0	87%	68.74%	*Ilyonectria destructans*	KAH7019735.1
short chain dehydrogenase	254	2.00E-122	99%	66.01%	*Ilyonectria destructans*	KAH7019726.1
serine hydrolase FSH	264	7.00E-92	98%	54.98%	*Dactylonectria macrodidyma*	KAH7133931.1
putative short-chain dehydrogenase/reductase	283	1.00E-154	98%	74.19%	*Ilyonectria destructans*	KAH7019737.1
beta-lactamase/transpeptidase-like protein	594	0	95%	77.76%	*Dactylonectria macrodidyma*	KAH7133934.1
trichothecene 3-o-acetyltransferase	454	0	97%	71.24%	*Dactylonectria macrodidyma*	KAH7133935.1
polyketide synthase	2690	0	99%	68.09%	*Dactylonectria macrodidyma*	KAH7133936.1
inorganic phosphate transport PHO88	165	1.00E-43	100%	42.86%	*Scedosporium apiospermum*	XP_016645996.1
putative O-methyltransferase	257	8.00E-151	100%	86.77%	*Dactylonectria macrodidyma*	KAH7133929.1
putative ariadne-like RING finger protein R81	697	0	97%	56.61%	*Colletotrichum siamense*	KAF4823164.1

96) *Verticillium nubilum* contig_ NMXI01000024, VRPKS-I-19 cluster

Gene name	Size	E value	% Coverage	% identity	Top blast hit	Accession number of top blast hit
uncharacterized protein B0J16DRAFT_30603	213	3.00E-85	99%	62.26%	*Fusarium flagelliforme*	XP_045983921.1
uncharacterized protein B0J16DRAFT_41549	302	1.00E-170	99%	75.08%	*Fusarium flagelliforme*	XP_045983920.1
uncharacterized protein B0J16DRAFT_36324	1353	0	98%	77.39%	*Fusarium flagelliforme*	XP_045983919.1
uncharacterized protein B0J16DRAFT_34344	1203	0	100%	76.84%	*Fusarium flagelliforme*	XP_045983918.1
hypothetical protein HYQ44_014562	136	0.028	75%	34.92%	*Verticillium longisporum*	KAG7105862.1
phthalate transporter	488	0	98%	75.57%	*Emericellopsis atlantica*	XP_046114251.1
hypothetical protein F5X68DRAFT_50483	670	0	100%	64.61%	*Plectosphaerella plurivora*	KAH6669659.1
Ketose-bisphosphate aldolase class-ii family protein	1134	0	98%	73.91%	*Colletotrichum higginsianum 349063*	XP_018154864.1
Type I Iterative PKS	2174	0	100%	85.90%	*Leptosphaeria biglobosa*	KAH9873394.1
major facilitator superfamily transporte	577	0	98%	67.65%	*Plectosphaerella plurivora*	KAH6679835.1
thioredoxin-like protein	162	3.00E-75	91%	73.65%	*Hypoxylon sp. FL0890*	KAI0834638.1
exopolygalacturonase B like protein	487	0	98%	68.39%	*Monosporascus sp. mg162*	RYP54678.1
Obg-like ATPase	394	0	100%	90.10%	*Pyricularia grisea*	XP_030984371.1
DeSI-like protein sdu1	219	6.00E-106	99%	75.12%	*Colletotrichum chlorophyti*	OLN97068.1
putative 37S ribosomal protein S18, mitochondrial	250	6.00E-93	100%	56.40%	*Colletotrichum nymphaeae SA-0*	KXH60786.1
Mitochondrial import inner membrane translo subunit tim44	540	0	100%	74.59%	*Sodiomyces alkalinus F11*	XP_028468965.1

97) *Verticillium nubilum* contig_ NMXI01000024, VRPKS-I-21 cluster

Gene name	Size	E value	% Coverage	% identity	Top blast hit	Accession number of top blast hit
uncharacterized protein B0J15DRAFT_50616	685	0	100%	67.92%	*Fusarium solani*	XP_046123856.1
ricin B lectin	175	4.00E-109	100%	84.57%	*Colletotrichum simmondsii*	KXH31006.1
Alpha/Beta hydrolase protein	230	4.00E-90	97%	66.67%	*Fusarium sp. MPI-SDFR-AT-00*	KAH7180213.1

general substrate transporter	230	3.00E-80	90%	62.88%	*Dactylonectria macrodidyma*	KAH7161520.1
kinesin family protein	811	0	99%	77.04%	*Plectosphaerella cucumerina*	KAH7367003.1
Lovastatin diketide synthase LovF 2	2453	0	100%	70.18%	*Colletotrichum chlorophyti*	OLN91972.1

98) *Verticillium nubilum* contig_ NMXI01000023, VRPKS-I-26 cluster

Gene name	Size	E value	% Coverage	% identity	Top blast hit	Accession number of top blast hit
Short chain dehydrogenase citE like protein	287	3.00E-110	99%	60.07%	*Colletotrichum camelliae*	KAH0428843.1
hypothetical protein ColSpa_11746	421	1.00E-83	85%	42.11%	*Colletotrichum spaethianum*	GJC88908.1
ankyrin repeat-containing domain protein	782	6.00E-124	88%	36.75%	*Plectosphaerella plurivora*	KAH6670890.1
hypothetical protein F5X68DRAFT_228683	77	2.00E-16	62%	73.47%	*Plectosphaerella plurivora*	KAH6692307.1
peptidase family C25-domain-containing prot	1017	0.00E+00	100%	54.44%	*Paraphoma chrysanthemicola*	KAH7091334.1
hypothetical protein CMUS01_12901	577	0	93%	86.78%	*Colletotrichum musicola*	KAF6813152.1
putative pectate lyase F	236	5.00E-141	97%	84.85%	*Colletotrichum fructicola*	XP_031890093.1
hypothetical protein VDGE_07268	990	0	94%	76.15%	*Plectosphaerella cucumerina*	KAH7362743.1
hypothetical protein F5X68DRAFT_264904	162	4.00E-44	84%	72.26%	*Plectosphaerella plurivora*	KAH6670875.1
uncharacterized protein KVR01_004169	130	9.00E-10	56%	43.24%	*Diaporthe batatas*	XP_044646333.1
hypothetical protein DL768_003915	2345	0	99%	51.02%	Whalleya microplaca	KAI1073750.1
TTL domain-containing protein	472	0	100%	74.69%	*Coniochaeta sp. 2T2.1*	KAB5518934.1
retinol dehydrogenase	343	2.00E-164	89%	72.40%	*Plectosphaerella cucumerina*	KAH7369374.1
peptidase family M28	373	0	91%	81.82%	*Dactylonectria estremocensis*	KAH7144232.1
hypothetical protein B0T11DRAFT_271851	339	1.00E-111	85%	58.16%	*Plectosphaerella cucumerina*	KAH7376217.1
hypothetical protein FPSE_04937	125	2.00E-50	100%	74.62%	*Fusarium pseudogramine CS3096*	XP_009256330.1
uncharacterized protein CGCS363_v014577	329	2.00E-11	29%	66.00%	*Colletotrichum siamense*	XP_036488857.1
hypothetical protein SODALDRAFT_32675:	200	2.00E-90	99%	56.78%	*Sodiomyces alkalinus F11*	XP_028470403.1

99) *Verticillium nubilum* contig_ NMXI01000025, VRPKS-I-23 cluster

Gene name	Size	E value	% Coverage	% identity	Top blast hit	Accession number of top blast hit
putative amid-like mitochondrial protein	196	2.00E-90	99%	72.31%	*Phaeoacremonium min UCRPA7*	XP_007913672.1
hypothetical protein SODALDRAFT_20092!	308	5.00E-23	34%	71.03%	*Sodiomyces alkalinus F11*	XP_028465407.1
hypothetical protein SODALDRAFT_33471:	229	1.00E-47	59%	65.22%	*Sodiomyces alkalinus F11*	XP_028465408.1
SRP54-domain-containing protein	668	0	100%	80.00%	*Sodiomyces alkalinus F11*	XP_028465413.1
hypothetical protein CSAL01_08458	323	9.00E-169	99%	85.94%	*Colletotrichum salicis*	KXH65676.1
PXMP2/4 family protein 3 like	229	1.00E-114	99%	76.21%	*Plectosphaerella cucumerina*	KAH7358280.1
hypothetical protein B0T11DRAFT_299362	113	7.00E-62	100%	79.65%	*Plectosphaerella cucumerina*	KAH7358281.1
virulence sensor protein bvgS	1391	0	99%	65.71%	*Plectosphaerella cucumerina*	KAH7358170.1
drug resistance transporter	588	0	99%	69.51%	*Plectosphaerella plurivora*	KAH6697430.1
phenolpthiocerol synthesis polyketide synthas ppsA	2376	0	99%	50.96%	*Sodiomyces alkalinus F11*	XP_028462492.1
hypothetical protein	544	2.00E-129	61%	65.14%	*Sodiomyces alkalinus F11*	XP_028462485.1

100) *Verticillium nubilum* contig_ NMXI01000041, VPKS-III cluster

Gene name	Size	E value	% Coverage	% identity	Top blast hit	Accession number of top blast hit
short chain dehydrogenase	300	1.00E-165	97%	77.40%	*Sodiomyces alkalinus F11*	XP_028470310.1
centrin-3	190	2.00E-95	98%	69.68%	*Sodiomyces alkalinus F11*	XP_028470317.1
Iron-sulfur protein IND1	296	2.00E-161	99%	81.29%	*Colletotrichum sidae*	TEA13372.1
Splicing factor spf30	304	4.00E-107	99%	72.19%	*Colletotrichum truncatum*	XP_036586172.1
hypothetical protein B0T11DRAFT_249550	636	3.00E-149	96%	60.57%	*Plectosphaerella cucumerina*	KAH7375296.1
putative mitochondrial 2-oxoglutarate/malate carrier protein	335	5.00E-172	100%	87.46%	*Colletotrichum gloeosporioides*	XP_045260884.1
exonuclease	1601	0	65%	76.44%	*Sodiomyces alkalinus F11*	XP_028470311.1
fructose-bisphosphate aldolase	362	0	100%	89.78%	*Plectosphaerella cucumerina*	KAH7375300.1
chalcone and stilbene synthase domain-contai protein	439	0	100%	75.45%	*Plectosphaerella cucumerina*	KAH7375301.1
apoptosis-antagonizing transcription factor	539	2.00E-166	100%	59.70%	*Plectosphaerella cucumerina*	KAH7375290.1
fungal specific transcription factor domain-containing protein	578	0	100%	72.90%	*Colletotrichum incanum*	KZL80546.1
Feruloyl esterase B	137	3.00E-32	91%	50.00%	*Colletotrichum trifolii*	TDZ44944.1
oxidoreductase	451	0	99%	64.59%	*Plectosphaerella plurivora*	KAH6681205.1
Acetyltransferase	273	1.00E-145	86%	82.70%	*Colletotrichum higginsianum 349063*	XP_018159255.1
carbonic anhydrase	271	2.00E-142	93%	74.41%	*Colletotrichum incanum*	KZL87566.1

101) *Verticillium nubilum* contig_ NMXI01000015, VRPKS-I-4 cluster

Gene name	Size	E value	% Coverage	% identity	Top blast hit	Accession number of top blast hit
hypothetical protein F5X68DRAFT_175070	212	9.00E-136	97%	89.37%	*Plectosphaerella plurivora*	KAH6672776.1
quinone oxidoreductase	335	1.00E-130	100%	69.35%	*Plectosphaerella cucumerina*	KAH7353556.1
hypothetical protein F5X68DRAFT_264742	1823	0	99%	67.93%	*Plectosphaerella plurivora*	KAH6672778.1
biotrophy-associated secreted protein 2	168	9.00E-33	98%	57.71%	*Colletotrichum truncatum*	XP_036577822.1
uncharacterized protein KVR01_013856	359	0	100%	70.75%	*Diaporthe batatas*	XP_044637156.1
MFS general substrate transporter	599	0	98%	67.68%	*Diaporthe batatas*	XP_044637161.1
hypothetical protein F4775DRAFT_593279	3987	0	99%	58.01%	*Biscogniauxia sp. FL1348*	KAI0597589.1
bacterial alpha-L-rhamnosidase-domain-containing protein	869	4.00E-36	19%	66.67%	*Plectosphaerella cucumerina*	KAH7347261.1
secreted protein	972	0	100%	69.20%	*Plectosphaerella cucumerina*	KAH7353831.1
Clp1 protein	469	0	93%	69.25%	*Sodiomyces alkalinus F11*	XP_028469991.1
Plasma membrane proteolipid 3 like protein	151	2.00E-63	100%	67.09%	*Plectosphaerella cucumerina*	KAH7353829.1
Argonaute-binding protein 1	473	9.00E-127	78%	48.94%	*Sodiomyces alkalinus F11*	XP_028469989.1
inositol polyphosphate multikinase	394	6.00E-172	96%	65.10%	*Colletotrichum tofieldiae*	GKT61693.1
Pre-mRNA-splicing factor ATP-dependent R helicase prp22	1188	0	99%	85.65%	*Colletotrichum siamense*	KAF4842091.1
HAD-superfamily hydrolase	371	4.00E-154	98%	62.47%	*Whalleya microplaca*	KAI1081230.1

102) *Verticillium tricorpus* contig_ JPET01000002, VRPKS-I-17 cluster

Gene name	Size	E value	% Coverage	% identity	Top blast hit	Accession number of top blast hit
Pet127-domain-containing protein	133	7.00E-37	89%	57.14%	*Sodiomyces alkalinus F11*	XP_028466993.1
splicing factor 3b subunit 4	372	2.00E-150	85%	79.87%	*Colletotrichum camelliae*	KAH0444958.1
Mitochondrial FAD carrier protein FLX1	310	1.00E-168	99%	73.99%	*Colletotrichum chlorophyti*	OLN84903.1
Asparagine-rich zinc finger protein	447	3.00E-121	94%	50.00%	*Colletotrichum gloeosporioides 23*	KAH9241535.1
Urm1-domain-containing protein	104	2.00E-56	96%	80.00%	*Xylaria curta*	KAI0546841.1
elongator protein 2	840	0.00%	99%	67.10%	*Colletotrichum truncatum*	XP_036582858.1
Lovastatin diketide synthase LovF 16	2468	0	92%	71.98%	*Colletotrichum chlorophyti*	OLN86478.1
hypothetical protein	255	8.00E-148	100%	80.08%	*Colletotrichum sojae*	KAF6806434.1
ABC transporter	1482	0	84%	71.01%	*Colletotrichum scovillei*	KAG7055337.1
Actin	375	0	100%	100.00%	*Tolypocladium paradoxum*	POR36442.1
54S ribosomal protein L7	356	0	98%	75.57%	*Sodiomyces alkalinus F11*	XP_028466988.1
nucleotide exchange factor Fes1	209	1.00E-95	97%	71.08%	*Sodiomyces alkalinus F11*	XP_028466987.1
ubiquitin C-terminal hydrolase	800	0	100%	63.55%	*Plectosphaerella cucumerina*	KAH7363099.1
6-phosphofructo-2-kinase	570	0.00%	100%	84.78%	*Plectosphaerella cucumerina*	KAH7363100.1
mitochondrial import receptor subunit tom-40	352	0	100%	83.33%	*Sodiomyces alkalinus F11*	XP_028466992.1
isoflavone reductase	204	3.00E-18	92%	54.46%	*Colletotrichum sojae*	KAF6819729.1

103) *Verticillium tricorpus* contig_ JPET01000002, VRPKS-I-14 cluster

Gene name	Size	E value	% Coverage	% identity	Top blast hit	Accession number of top blast hit
hypothetical protein E8E14_009652	900	5.00E-142	84%	38.36%	*Neopestalotiopsis sp. 37M*	KAF3020162.1
cytochrome P450 CYP4/CYP19/CYP26 subfamilies protein	519	0.00%	95%	77.98%	*Colletotrichum karsti*	XP_038744559.1
hypothetical protein FPCIR_5538	607	1.00E-127	93%	41.19%	*Fusarium pseudocircinatum*	KAF5592855.1
Acyl-CoA dehydrogenase/oxidase	505	8.00E-150	91%	52.93%	*Cordyceps javanica*	TQV95615.1
fatty acid desaturase domain-containing protein	577	0.00%	100%	60.33%	*Sarocladium implicatum*	KAH8177350.1
putative polyketide synthase	3161	0	100%	57.90%	*Truncatella angustata*	XP_045957259.1
major facilitator superfamily domain-containing protein	586	0	84%	61.68%	*Truncatella angustata*	XP_045957260.1
cytochrome P450	509	0	100%	79.45%	*Plectosphaerella cucumerina*	KAH7367666.1
hypothetical protein CEP53_002056	374	9.00E-149	93%	71.63%	*Fusarium sp. AF-6*	RSL69861.1

hypothetical protein B0T11DRAFT_56928	204	1.00E-100	100%	72.46%	*Plectosphaerella cucumerina*	KAH7367670.1
hypothetical protein B0T11DRAFT_350760	560	0	99%	86.94%	*Plectosphaerella cucumerina*	KAH7369187.1
Protein rds1 like protein	465	0	98%	80.57%	*Sodiomyces alkalinus F11*	XP_028471380.1
FK506-binding protein 2	194	8.00E-82	100%	69.59%	*Colletotrichum orbiculare MAFF 240422*	TDZ17824.1
hypothetical protein HYQ45_016732	88	2.00E-04	100%	72.53%	*Verticillium longisporum*	KAG7113540.1
hypothetical protein FALBO_210	142	2.00E-70	100%	73.24%	*Fusarium albosuccineum*	KAF4472888.1
hypothetical protein F5883DRAFT_436293	936	0	99%	71.46%	*Diaporthaceae sp. PMI_573*	KAH8748597.1

104) *Verticillium tricorpus* contig_ JPET01000001, VRPKS-I-16 cluster

Gene name	Size	E value	% Coverage	% identity	Top blast hit	Accession number of top blast hit
hypothetical protein B0T11DRAFT_7516	205	5.00E-49	64%	69.92%	*Plectosphaerella cucumerina*	KAH7375254.1
choline/ethanolaminephosphotransferase	427	0	92%	82.37%	*Sodiomyces alkalinus F11*	XP_028462783.1
estradiol 17-beta-dehydrogenase	282	3.00E-120	100%	65.60%	*Colletotrichum tofieldiae*	KZL75089.1
glutaminyl-peptide cyclotransferase	388	0	100%	72.89%	*Sodiomyces alkalinus F11*	XP_028462781.1
Dolichyl-diphosphooligosaccharide--protein glycosyltransferase subunit SWP1	287	2.00E-91	99%	58.19%	*Colletotrichum sidae*	TEA10577.1
hypothetical protein HYQ44_009310	162	1.00E-25	77%	63.08%	*Verticillium longisporum*	KAG7111115.1
xylosidase/arabinosidase	599	0	100%	90.82%	*Plectosphaerella plurivora*	KAH6670864.1
flavin depend monooxygenase that catalyses the oxidation of rubrofusarin to 9-hydroxyrubrofusarin	523	0	100%	78.29%	*Fusarium albosuccineum*	KAF4463211.1
hypothetical protein CEP51_004340	167	3.00E-62	100%	62.28%	*Fusarium floridanum*	RSL83703.1
Alpha/Beta hydrolase protein	557	0	93%	66.60%	*Plectosphaerella plurivora*	KAH6688446.1
putative polyketide synthase	2599	0	99%	72.03%	*Plectosphaerella cucumerina*	KAH7369281.1
serine hydrolase FSH	239	7.00E-81	100%	56.33%	*Plectosphaerella plurivora*	KAH6673998.1
uncharacterized protein GCG54_00000013	638	0	94%	64.82%	*Colletotrichum gloeosporioides*	XP_045266644.1
hypothetical protein B0T11DRAFT_249503	1066	0.00%	100%	58.96%	*Plectosphaerella cucumerina*	KAH7375266.1
RNA recognition domain-containing protein	155	5.00E-62	83%	77.69%	*Colletotrichum sojae*	KAF6816001.1
hypothetical protein F5X68DRAFT_6871	1669	0	97%	59.17%	*Plectosphaerella plurivora*	KAH6687842.1
subtilase	859	0	97%	69.29%	*Plectosphaerella plurivora*	KAH6687843.1
Sorting nexin-4	475	0	100%	79.88%	*Plectosphaerella plurivora*	KAH6687844.1
mitochondrial distribution and morphology protein	542	0	94%	76.37%	*Plectosphaerella plurivora*	KAH6687845.1
adenylate kinase	268	8.00E-155	99%	80.97%	*Colletotrichum karsti*	XP_038745165.1

105) *Verticillium tricorpus* contig_ JPET01000001, VRPKS-I-25 cluster

Gene name	Size	E value	% Coverage	% identity	Top blast hit	Accession number of top blast hit
hypothetical protein B0T11DRAFT_325269	436	1.00E-116	100%	51.56%	*Plectosphaerella cucumerina*	KAH7366966.1
hypothetical protein B0T11DRAFT_49340	356	9.00E-114	91%	60.07%	*Plectosphaerella cucumerina*	KAH7366962.1
mediator complex, subunit Med18	269	2.00E-164	99%	80.07%	*Plectosphaerella cucumerina*	KAH7347312.1
bifunctional polynucleotide phosphatase/kinase	455	0	99%	67.17%	*Sodiomyces alkalinus F11*	XP_028468241.1
calcium-transporting ATPase	1071	0	98%	74.39%	*Monosporascus sp. MG133*	RYP34097.1
Protein STB5-like protein 3	771	0	88%	61.36%	*Colletotrichum chlorophyti*	OLN81943.1
Thiol-specific monooxygenase like protein [	492	0	100%	67.14%	*Colletotrichum fructicola*	XP_031891081.1
hypothetical protein	530	8.00E-170	87%	57.91%	*Colletotrichum incanum*	KZL63649.1
Non-reducing end alpha-L-arabinofuranosidase BoGH43A	603	0	95%	84.90%	*Colletotrichum siamense*	XP_036494585.1
polyketide synthase	2358	0	99%	89.27%	*Stachybotrys chartarum IBT 40293*	KFA46917.1
short chain dehydrogenase	304	0	98%	91.33%	*Stachybotrys chartarum IBT 7711*	KEY68921.1
dihydroxy-acid dehydratase	616	0	99%	82.04%	*Plectosphaerella plurivora*	KAH6671001.1
S-adenosyl-L-methionine-dependent methyltransferase	313	1.00E-155	92%	81.10%	*Sodiomyces alkalinus F11*	XP_028466391.1
hypothetical protein SODALDRAFT_165672	372	2.00E-37	88%	40.44%	*Sodiomyces alkalinus F11*	XP_028466392.1
hypothetical protein CFIO01_00827	673	8.00E-149	92%	49.61%	*Colletotrichum fioriniae PJ7*	EXF83685.1
unnamed protein product	187	2.00E-90	99%	64.17%	*Clonostachys solani*	CAH0054579.1
glucosidase II beta subunit-like protein-domain-containing protein	524	0	95%	63.42%	*Plectosphaerella plurivora*	KAH6680057.1
hypothetical protein	749	0	92%	64.30%	*Plectosphaerella plurivora*	KAH6680056.1

106) *Verticillium tricorpus* contig_ JPET01000001, VRPKS-I-19 cluster

Gene name	Size	E value	% Coverage	% identity	Top blast hit	Accession number of top blast hit
Alpha-glucosides permease MPH3	542	0	99%	74.68%	*Cylindrodendrum hubeiense*	KAF7546071.1
hypothetical protein SODALDRAFT_333514	77	2.00E-22	97%	57.69%	*Sodiomyces alkalinus F11*	XP_028465568.1
hypothetical protein ACRE_064810	279	2.00E-76	98%	49.47%	*Acremonium chrysogenum ATCC 11550*	KFH42767.1
N-glycosidase like protein	198	3.00E-56	80%	58.12%	*Plectosphaerella cucumerina*	KAH7347667.1

TIM44 subunit of mitochondria import inner membrane translocase	541	0	100%	76.10%	*Sodiomyces alkalinus F11*	XP_028468965.1
mitochondrial ribosomal protein subunit s18	250	2.00E-90	100%	56.00%	*Colletotrichum fioriniae PJ7*	EXF75199.1
DeSI-like protein sdu1	236	1.00E-113	99%	75.21%	*Colletotrichum chlorophyti*	OLN97068.1
Obg-like ATPase 1	394	0	100%	90.36%	*Pyricularia grisea*	XP_030984371.1
exopolygalacturonase	461	0	98%	69.44%	*Monosporascus sp. mg162*	RYP54678.1
hypothetical protein F5882DRAFT_368496	392	2.00E-51	85%	31.40%	*Hyaloscypha sp. PMI_1271*	KAH8752665.1
hypothetical protein AK830_g4742	500	3.00E-68	83%	38.67%	*Neonectria ditissima*	KPM41855.1
thioredoxin-like protein	219	5.00E-76	67%	76.19%	*Hypoxylon sp. FL0890*	KAI0834638.1
MFS general substrate transporter	574	0	89%	75.43%	*Sodiomyces alkalinus F11*	XP_028468956.1
hypothetical protein J1614_005793	317	4.00E-107	95%	57.76%	*Leptosphaeria biglobosa*	KAH9873395.1
Type I Polyketide synthase	2176	0	99%	78.03%	*Leptosphaeria biglobosa*	KAH9873394.1
Ketose-bisphosphate aldolase class-ii family protein	1133	0	99%	73.21%	*Colletotrichum higginsianum IMI 349063*	XP_018154864.1
uncharacterized protein CkaCkLH20_03768	1135	0	100%	67.57%	*Colletotrichum karsti*	XP_038748329.1
Cytochrome monooxygenase like protein	98	1.00E-08	70%	43.66%	*Fusarium austroafricanum*	KAF4441573.1
cutinase-2	224	8.00E-132	92%	88.41%	*Plectosphaerella cucumerina*	KAH7368196.1
probable beta-glucosidase precursor	832	0	99%	67.55%	*Aspergillus sydowii CBS 593.65*	XP_040708017.1
uncharacterized protein FRV6_15384	281	8.00E-65	97%	45.13%	*Fusarium oxysporum*	SCO91256.1
vacuolar protein sorting-associated protein	435	6.00E-163	98%	72.98%	*Plectosphaerella cucumerina*	KAH7368195.1

107) *Verticillium tricorpus* contig_ JPET01000003, VRPKS-I-13 cluster

Gene name	Size	E value	% Coverage	% identity	Top blast hit	Accession number of top blast hit
hypothetical protein F5X68DRAFT_47703	256	5.00E-64	89%	48.25%	*Plectosphaerella plurivora*	KAH6693919.1
hypothetical protein B0T11DRAFT_86728	557	0	99%	63.55%	*Plectosphaerella cucumerina*	KAH7362695.1
2OG-Fe oxygenase superfamily protein	332	1.00E-159	98%	72.40%	*Colletotrichum nymphaeae SA-01*	KXH44953.1
Uracil permease	525	0	94%	81.89%	*Colletotrichum camelliae*	KAH0420306.1
hypothetical protein FALBO_5956	314	0	100%	86.31%	*Fusarium albosuccineum*	KAF4467173.1
polyketide synthase	3209	0	99%	55.19%	*Fusarium avenaceum*	KIL85244.1
hypothetical protein HBH70_059960	236	5.00E-33	86%	69.95%	*Parastagonospora nodorum*	KAH5143667.1
endo-1,4-beta-glucanase	297	6.00E-97	81%	60.82%	*Colletotrichum sojae*	KAF6788143.1
epoxide hydrolase	409	0	98%	62.94%	*Plectosphaerella plurivora*	KAH6662678.1
hypothetical protein SODALDRAFT_4980	221	3.00E-60	81%	60.22%	*Sodiomyces alkalinus F11*	XP_028469806.1

108) *Verticillium tricorpus* contig_ JPET01000004, VRPKS-I-26 cluster

Gene name	Size	E value	% Coverage	% identity	Top blast hit	Accession number of top blast hit
hypothetical protein SODALDRAFT_319240	134	4.00E-25	95%	39.39%	*Sodiomyces alkalinus F11*	XP_028470409.1
alpha-l-rhamnosidase	898	0	97%	68.71%	*Colletotrichum truncatum*	XP_036586343.1
hypothetical protein	427	2.00E-137	77%	58.86%	*Trichoderma arundinaceum*	RFU78228.1
FAD-dependent monooxygenase	452	0	96%	84.70%	*Stachybotrys elegans*	KAH7303249.1
heterokaryon incompatibility protein-domain-containing protein	742	0	100%	75.88%	*Chaetomium sp. MPI-CAGE-AT-0009*	KAH6847694.1
peptidase family M28	347	0	91%	77.71%	*Dactylonectria estremocensis*	KAH7144232.1
retinol dehydrogenase	312	0	100%	78.21%	*Plectosphaerella cucumerina*	KAH7369374.1
TTL domain-containing protein	473	0	100%	73.03%	*Coniochaeta sp. 2T2.1*	KAB5518934.1
Whalleya microplaca	2350	52.66%	0	2459	*2375*	
uncharacterized protein KVR01_004169	224	2.00E-05	25%	43.86%	*Diaporthe batatas*	XP_044646333.1
hypothetical protein B0T11DRAFT_318355	163	4.00E-57	98%	67.68%	*Plectosphaerella cucumerina*	KAH7362742.1
hypothetical protein	1004	0	100%	72.57%	*Plectosphaerella cucumerina*	KAH7362743.1

109) *Verticillium tricorpus* contig_ JPET01000006, VRPKS-I-20 cluster

Gene name	Size	E value	% Coverage	% identity	Top blast hit	Accession number of top blast hit
SUMO-activating enzyme subunit uba-2	353	4.00E-153	93%	73.03%	*Sodiomyces alkalinus F11*	XP_028470156.1
hypothetical protein	309	7.00E-98	99%	62.66%	*Plectosphaerella cucumerina*	KAH7347695.1
feruloyl esterase b	315	1.00E-176	93%	79.52%	*Colletotrichum musicola*	KAF6811137.1
long-chain base protein	554	0	100%	70.97%	*Plectosphaerella cucumerina*	KAH7347694.1
uncharacterized protein	1723	0	64%	65.16%	*Colletotrichum sublineola*	KDN67348.1
mannan endo-1,4-beta-mannosidase	401	0	95%	82.55%	*Plectosphaerella plurivora*	KAH6695781.1
bifunctional p-450-like protein	1076	0	100%	75.37%	*Colletotrichum truncatum*	XP_036579478.1
polyketide synthase	2475	0	99%	67.08%	*Penicillium arizonense*	XP_022486633.1
Alpha-ketoglutarate-dependent 2,4-dichlorophenoxyacetate dioxygenase	327	2.00E-174	98%	68.28%	*Penicillium arizonense*	XP_022486531.1
Cytochrome P450 4V2	763	0	67%	65.78%	*Penicillium arizonense*	XP_022486670.1
unnamed protein product	525	0	96%	55.47%	*Akanthomyces lecanii RCEF 1005*	OAA79799.1
hypothetical protein FALBO_6676	100	5.00E-18	98%	43.88%	*Fusarium albosuccineum*	KAF4466462.1
hypothetical protein	906	0	63%	70.05%	*Plectosphaerella cucumerina*	KAH7374727.1
hypothetical protein B0J13DRAFT_486524	254	2.00E-134	100%	74.02%	*Dactylonectria estremocensis*	KAH7119443.1
uncharacterized protein TRUGW13939_09250	346	0	99%	74.13%	*Talaromyces rugulosus*	XP_035348268.1

hypothetical protein EDB80DRAFT_562441	1180	0	58%	51.94%	*Ilyonectria destructans*	KAH7018764.1

110) *Verticillium tricorpus* contig_ JPET01000004, PKS-III cluster

Gene name	Size	E value	% Coverage	% identity	Top blast hit	Accession number of top blast hit
NADH-ubiquinone oxidoreductase 30.4 kDa subunit	336	1.00E-49	75%	50.19%	*Plectosphaerella cucumerina*	KAH7375303.1
short chain dehydrogenase	301	3.00E-165	96%	76.63%	*Sodiomyces alkalinus F11*	XP_028470310.1
hypothetical protein	189	2.00E-88	98%	68.09%	*Sodiomyces alkalinus F11*	XP_028470317.1
hypothetical protein	290	4.00E-166	94%	79.56%	*Ilyonectria destructans*	KAH7019330.1
Splicing factor spf30	303	4.00E-114	99%	69.38%	*Colletotrichum truncatum*	XP_036586172.1
hypothetical protein B0T11DRAFT_249550	599	1.00E-160	96%	59.33%	*Plectosphaerella cucumerina*	KAH7375296.1
putative mitochondrial 2-oxoglutarate/malate carrier protein	335	0	100%	86.87%	*Colletotrichum chlorophyti*	OLN95994.1
uncharacterized protein D7B24_007593	549	0	98%	67.68%	*Verticillium nonalfalfae*	XP_028494368.1
PAB-dependent poly -specific ribonuclease subunit PAN2	1062	0	98%	78.66%	*Plectosphaerella cucumerina*	KAH7375292.1
fructose-bisphosphate aldolase	362	0	100%	94.48%	*Verticillium alfalfae VaMs.102*	XP_003000775.1
putative chalcone and stilbene synthase domain-containing protein	444	0	100%	73.65%	*Colletotrichum sublineola*	KDN65911.1
hypothetical protein K456DRAFT_1765144	127	3.00E-19	86%	58.93%	*Colletotrichum gloeosporioides 23*	KAH9228351.1
TRAUB-domain-containing protein	594	5.00E-121	83%	71.43%	*Sodiomyces alkalinus F11*	XP_028470322.1
Transcriptional activator protein acu-15	806	0	100%	72.75%	*Colletotrichum higginsianum*	TID06918.1
Feruloyl esterase B	294	2.00E-153	94%	74.55%	*Colletotrichum orbiculare MAFF 240422*	TDZ25527.1
oxidoreductase	451	0	99%	65.11%	*Plectosphaerella plurivora*	KAH6681205.1
Acetyltransferase	269	4.00E-148	88%	83.54%	*Colletotrichum higginsianum IMI 349063*	XP_018159255.1
carbonic anhydrase	271	1.00E-145	93%	76.38%	*Colletotrichum incanum*	KZL87566.1
hypothetical protein	289	2.00E-54	65%	52.91%	*Colletotrichum incanum*	OHW90569.1
hypothetical protein B0T11DRAFT_143288	221	2.00E-82	88%	59.69%	*Plectosphaerella cucumerina*	KAH7347634.1
alpha-ketoglutarate-dependent sulfonate dioxygenase	378	0	100%	83.60%	*Plectosphaerella plurivora*	KAH6681182.1

111) *Verticillium tricorpus* contig_ JPET01000002, VRPKS-I-23 cluster

Gene name	Size	E value	% Coverage	% identity	Top blast hit	Accession number of top blast hit
hypothetical protein SODALDRAFT_67746	364	1.00E-64	99%	38.07%	*Sodiomyces alkalinus F11*	XP_028463289.1
homoserine kinase	359	2.00E-176	100%	81.62%	*Plectosphaerella cucumerina*	KAH7362595.1
SnodProt1	138	8.00E-64	84%	76.72%	*Plectosphaerella cucumerina*	KAH7368131.1
uncharacterized protein VDAG_01853	129	3.00E-07	52%	79.49%	*Verticillium dahliae VdLs.17*	XP_009656177.1
hypothetical protein SODALDRAFT_82915	851	0	96%	62.06%	*Sodiomyces alkalinus F11*	XP_028462489.1
uncharacterized protein CGCS363_v012205	365	5.00E-104	93%	51.23%	*Colletotrichum siamense*	XP_036491211.1
aromatic prenyltransferase	195	1.00E-68	70%	71.53%	*Plectosphaerella cucumerina*	KAH7358950.1
aromatic prenyltransferase	238	3.00E-89	94%	64.00%	*Plectosphaerella cucumerina*	KAH7358950.1
Reducing polyketide synthase	2346	0	99%	51.56%	*Sodiomyces alkalinus F11*	XP_028462492.1
hypothetical protein	558	0	96%	52.71%	*Stylonectria norvegica*	KAF7555861.1
virulence sensor protein bvgS	1386	0	99%	66.88%	*Plectosphaerella cucumerina*	KAH7358170.1
hypothetical protein B0T11DRAFT_299362	191	1.00E-62	59%	82.30%	*Plectosphaerella cucumerina*	KAH7358281.1
hypothetical protein B0T11DRAFT_284115	230	1.00E-97	100%	76.09%	*Plectosphaerella cucumerina*	KAH7358280.1
uncharacterized protein CORC01_08067	323	3.00E-168	100%	86.69%	*Colletotrichum orchidophilum*	XP_022473766.1
SRP54-domain-containing protein	668	0.00E+00	100%	81.04%	*Sodiomyces alkalinus F11*	XP_028465413.1
hypothetical protein SODALDRAFT_201001	1494	0	97%	61.28%	*Sodiomyces alkalinus F11*	XP_028465411.1

112) *Verticillium tricorpus* contig_ JPET01000002, VRPKS-I-12 cluster

Gene name	Size	E value	% Coverage	% identity	Top blast hit	Accession number of top blast hit
hypothetical protein FPOAC1_009761	238	4.00E-82	89%	58.60%	*Fusarium poae*	XP_044706852.1
cytochrome P450	474	0	97%	74.36%	*Sodiomyces alkalinus F11*	XP_028469280.1
Beta-ketoacyl synthase	366	1.00E-92	99%	45.26%	*Tolypocladium paradoxum*	POR38434.1
hypothetical protein CSAL01_09615	432	7.00E-180	96%	56.25%	*Colletotrichum salicis*	KXH61197.1
FAD/NAD -binding domain-containing protein	426	0	93%	74.12%	*Sodiomyces alkalinus F11*	XP_028469276.1
hypothetical protein SODALDRAFT_331204	890	0	88%	70.13%	*Sodiomyces alkalinus F11*	XP_028469275.1
polyketide synthase	3982	0	96%	75.43%	*Sodiomyces alkalinus F11*	XP_028469272.1
L-2,4-diaminobutyrate decarboxylase	515	0	87%	65.56%	*Colletotrichum trifolii*	TDZ67276.1
hypothetical protein	388	4.00E-127	99%	58.99%	*Colletotrichum fructicola*	XP_031880272.1

hypothetical protein BN1723_013595	316	2.00E-72	78%	48.28%	*Verticillium longisporum*	CRK25564.1
laccase-like multicopper oxidase	561	0	98%	66.79%	*Plectosphaerella cucumerina*	KAH7358155.1
Conidial yellow pigment biosynthesis polyketide synthase	2188	0	100%	79.19%	*Colletotrichum karsti*	XP_038751444.1
hypothetical protein	325	0	99%	85.80%	*Colletotrichum truncatum*	XP_036586400.1
hypothetical protein	425	1.00E-145	100%	56.02%	*Colletotrichum plurivorum*	KAF6839547.1
transcription factor Cmr1	925	0	98%	72.32%	*Colletotrichum musicola*	KAF6845250.1
glycosyl transferase family protein	325	9.00E-164	100%	77.91%	*Plectosphaerella cucumerina*	KAH7358014.1
aldolase	245	8.00E-124	99%	75.31%	*Xylariaceae sp. FL0662B*	KAI0012622.1

113) *Verticillium tricorpus* contig_ JPET01000008, VRPKS-I-9 cluster

Gene name	Size	E value	% Coverage	% identity	Top blast hit	Accession number of top blast hit
Phosphomevalonate kinase	417	0	100%	65.32%	*Colletotrichum shisoi*	TQN71383.1
ATP-dependent RNA helicase DBP7	768	0	100%	73.43%	*Plectosphaerella cucumerina*	KAH7376931.1
major facilitator superfamily transporter	532	0	90%	78.38%	*Plectosphaerella plurivora*	KAH6695233.1
hypothetical protein F5Y05DRAFT_369401	505	8.00E-148	86%	54.36%	*Hypoxylon sp. FL0543*	KAI1142003.1
metalloprotease	460	2.00E-133	45%	86.06%	*Colletotrichum incanum*	OHW96414.1
uncharacterized protein B0I36DRAFT_355828	145	5.00E-08	42%	75.81%	*Microdochium trichocladiopsis*	XP_046005612.1
polyketide synthase 3	3971	0	99%	71.29%	*Hirsutella rhossiliensis*	XP_044717051.1
para-nitrobenzyl esterase	560	0	96%	69.14%	*Plectosphaerella cucumerina*	KAH7376670.1
polysaccharide deacetylase	526	6.00E-168	68%	79.17%	*Plectosphaerella cucumerina*	KAH7374645.1
hypothetical protein B0T11DRAFT_318679	475	1.00E-102	43%	72.12%	*Plectosphaerella cucumerina*	KAH7363183.1
hypothetical protein ISF_06027	85	5.00E-21	75%	56.25%	*Cordyceps fumosorosea ARSEF 2679*	XP_018703129.1
tetratricopeptide repeat protein 1	285	3.00E-111	89%	80.39%	*Colletotrichum karsti*	XP_038750970.1
hypothetical protein	270	2.00E-141	99%	71.17%	*Thozetella sp. PMI_491*	KAH8895695.1
C6 zinc finger domain-containing protein	705	0	100%	64.78%	*Colletotrichum karsti*	XP_038750968.1

114) *Verticillium tricorpus* contig_ JPET01000003, VRPKS-I-2 cluster

Gene name	Size	E value	% Coverage	% identity	Top blast hit	Accession number of top blast hit

uncharacterized protein GCG54_00004628	533	0	97%	60.66%	*Colletotrichum gloeosporioides*	XP_045262617.1
snare associated Golgi protein-domain-containing protein	419	2.00E-136	78%	73.41%	*Plectosphaerella plurivora*	KAH6691624.1
smr domain-containing protein	730	0	100%	84.04%	*Colletotrichum graminicola M1.001*	XP_008090428.1
hypothetical protein B0T11DRAFT_85615	177	5.00E-33	98%	44.83%	*Plectosphaerella cucumerina*	KAH7362564.1
D-isomer specific 2-hydroxyacid dehydrogenase	432	0	71%	78.46%	*Plectosphaerella plurivora*	KAH6689062.1
Major Facilitator Superfamily protein	577	0	100%	83.14%	*Colletotrichum fructicola*	KAF4886521.1
uncharacterized protein CGMCC3_g17406	167	4.00E-110	100%	92.22%	*Colletotrichum fructicola*	XP_031875989.1
Alpha/beta hydrolase	433	0	99%	84.26%	*Colletotrichum fructicola*	XP_031875995.1
polyketide synthase	4429	0	90%	85.90%	*Colletotrichum fructicola*	KAF4886526.1
uncharacterized protein CGMCC3_g17410	358	0	98%	89.80%	*Colletotrichum fructicola*	XP_031875992.1
hypothetical protein	508	0	100%	72.83%	*Colletotrichum fructicola*	KAF4886529.1
major facilitator superfamily domain-containing protein	712	0	78%	81.48%	*Plectosphaerella plurivora*	KAH6687355.1
O-methyltransferase	233	2.00E-111	100%	70.21%	*Plectosphaerella plurivora*	KAH6687359.1
kinase-like domain-containing protein	119	8.00E-64	100%	71.43%	*Tolypocladium ophioglossoides CBS 100239*	KND87680.1
hypothetical protein	882	0	53%	85.41%	*Plectosphaerella cucumerina*	KAH7362562.1

115) *Verticillium tricorpus* contig_ JPET01000003, VRPKS-I-6 cluster

Gene name	Size	E value	% Coverage	% identity	Top blast hit	Accession number of top blast hit
COX17 protein	70	2.00E-22	71%	84.00%	*Diaporthaceae sp. PMI_573*	KAH8788412.1
hypothetical protein HYQ45_010318	159	3.00E-51	87%	61.87%	*Verticillium longisporum*	KAG7131019.1
protein PXR1	365	2.00E-74	50%	81.94%	*Plectosphaerella cucumerina*	KAH7368324.1
hypothetical protein CSOJ01_13003	489	0	94%	76.29%	*Colletotrichum sojae*	KAF6797496.1
hypothetical protein CSPAE12_03025	633	2.00E-134	80%	48.46%	*Colletotrichum incanum*	OHW98243.1
hypothetical protein C8035_v000460	324	4.00E-132	97%	64.17%	*Colletotrichum spinosum*	TDZ12713.1
hypothetical protein EDB80DRAFT_717894	251	1.00E-50	89%	44.36%	*Ilyonectria destructans*	KAH7006831.1
Transaldolase	365	0	100%	87.12%	*Ilyonectria destructans*	KAH7006832.1
membrane transporter D1	599	0	99%	82.58%	*Ilyonectria destructans*	KAH7006833.1
thioredoxin	329	1.00E-171	100%	72.42%	*Plectosphaerella plurivora*	KAH6691560.1
uncharacterized protein BDZ99DRAFT_567991	184	4.00E-20	90%	31.95%	*Mytilinidion resinicola*	XP_033581321.1

serine hydrolase-domain-containing protein	284	3.00E-130	100%	63.38%	*Aspergillus avenaceus*	KAE8150729.1
Polyketide synthase-nonribosomal peptide synthetase	4028	0	99%	66.86%	*Aspergillus avenaceus*	KAE8150730.1
hypothetical protein CC78DRAFT_576631	470	0	100%	64.04%	*Didymosphaeria enalia*	KAF2268082.1
P-loop containing nucleoside triphosphate hydrolase protein	1108	0	98%	58.55%	*Clohesyomyces aquaticus*	ORY13587.1
hypothetical protein	466	0	99%	62.99%	*Didymosphaeria enalia*	KAF2268085.1
cytochrome P450	458	0	94%	66.59%	*Aspergillus alliaceus*	XP_031905229.1
putative alcohol dehydrogenase	370	0	100%	77.34%	*Aspergillus avenaceus*	KAE8150737.1

116) *Verticillium tricorpus* contig_ JPET01000007, VRPKS-I-5 cluster

**Gene name**	**Size**	**E value**	**% Coverage**	**% identity**	**Top blast hit**	**Accession number of top blast hit**
pseudouridine synthase [Sodiomyces alkalinus F11]	105	7.00E-15	87%	44.79%	*Sodiomyces alkalinus F11*	XP_028471325.1
GPI-GlcNAc transferase complex [Colletotrichum graminicola M1.001]	222	3.00E-54	99%	60.62%	*Colletotrichum graminicola M1.001*	XP_008094050.1
hypothetical protein SODALDRAFT_41197 [Sodiomyces alkalinus F11]	1644	0	96%	73.19%	*Sodiomyces alkalinus F11*	XP_028471323.1
glutathione S-transferase	244	7.00E-126	99%	78.92%	*fungal sp. No.14919*	GAW23379.1
major facilitator superfamily-domain-containing protein [Biscogniauxia mediterranea]	612	0.00E+00	88%	73.20%	*Biscogniauxia mediterranea*	KAI1639146.1
uncharacterized protein	191	2.00E-59	87%	53.25%	*Cucurbitaria berberidis CBS 394.84*	XP_040789151.1
hypothetical protein B0I37DRAFT_378309 [Chaetomium sp. MPI-CAGE-AT-0009]	505	2.00E-63	66%	56.88%	*Chaetomium sp. MPI-CAGE-AT-0009*	KAH6844803.1
putative lovastatin nonaketide synthase protein	4131	0.00E+00	96%	65.60%	*Biscogniauxia sp. FL1348*	KAI0597589.1
hypothetical protein B0T11DRAFT_333834 [Plectosphaerella cucumerina]	160	9.00E-32	63%	64.71%	*Plectosphaerella cucumerina*	KAH7347765.1
Isochorismatase-like protein [Plectosphaerella plurivora]	244	1.00E-128	90%	77.93%	*Plectosphaerella plurivora*	KAH6676014.1
vacuolar protein-sorting-associated protein [Plectosphaerella cucumerina]	228	4.00E-112	100%	86.40%	*Plectosphaerella cucumerina*	KAH7347768.1
Carboxypeptidase 2 like protein	546	0	99%	77.29%	*Stachybotrys chartarum IBT 40288*	KFA75754.1
zinc-containing alcohol	98	8.00E-54	100%	90.82%	*Chaetomium sp. MPI-CAGE-AT-0009*	KAH6848194.1

117) *Verticillium tricorpus* contig_ JPET01000005, VRPKS-I-17 cluster

Gene name	Size	E value	% Coverage	% identity	Top blast hit	Accession number of top blast hit
hypothetical protein CGLO_11330	334	1.00E-174	90%	81.19%	*Colletotrichum gloeosporioides Cg-14*	EQB49343.1
isoflavone reductase	307	5.00E-72	98%	48.03%	*Colletotrichum musicola*	KAF6837214.1
mitochondrial import receptor subunit tom-40	352	0	100%	83.33%	*Sodiomyces alkalinus F11*	XP_028466992.1
6-phosphofructo-2-kinase	570	0	93%	88.60%	*Colletotrichum karsti*	XP_038750648.1
ubiquitin C-terminal hydrolase	819	0	100%	65.23%	*Plectosphaerella cucumerina*	KAH7363099.1
54S ribosomal protein L7	356	0	98%	76.42%	*Sodiomyces alkalinus F11*	XP_028466988.1
nucleotide exchange factor Fes1	209	2.00E-96	97%	71.57%	*Sodiomyces alkalinus F11*	XP_028466987.1
Actin	375	0	100%	100.00%	*Tolypocladium paradoxum*	POR36442.1
ABC transporter	1321	0	98%	68.58%	*Pseudomassariella vexata*	XP_040710981.1
duf341 family	255	1.00E-148	100%	80.86%	*Colletotrichum sojae*	KAF6806434.1
Lovastatin diketide synthase LovF 16	2277	0	99%	72.06%	*Colletotrichum chlorophyti*	OLN86478.1
hypothetical protein B0T11DRAFT_282059	113	9.00E-46	83%	75.53%	*Plectosphaerella cucumerina*	KAH7363092.1
elongator protein 2	837	0	100%	67.42%	*Colletotrichum tofieldiae*	KZL71342.1
ubiquitin-related modifier 1	104	4.00E-56	99%	80.58%	*Cordyceps militaris CM01*	XP_006668991.1
Asparagine-rich zinc finger protein	441	6.00E-125	93%	50.12%	*Colletotrichum fioriniae PJ7*	EXF85239.1
Mitochondrial FAD carrier protein FLX1	310	4.00E-158	99%	74.61%	*Colletotrichum chlorophyti*	OLN84903.1
splicing factor 3b subunit 4	337	3.00E-153	94%	80.50%	*Colletotrichum camelliae*	KAH0444958.1

118) *Verticillium zaregamsianum* contig_NMXM01000005, VRPKS-I-23 cluster

Gene name	Size	E value	% Coverage	% identity	Top blast hit	Accession number of top blast hit
hypothetical protein SODALDRAFT_201001	2144	0	99%	61.19%	*Sodiomyces alkalinus F11*	XP_028465411.1
SRP54-domain-containing protein	689	0	100%	78.30%	*Sodiomyces alkalinus F11*	XP_028465413.1
uncharacterized protein CORC01_08067	323	3.00E-168	100%	86.69%	*Colletotrichum orchidophilum*	XP_022473766.1
hypothetical protein B0T11DRAFT_284115	230	1.00E-97	100%	76.09%	*Plectosphaerella cucumerina*	KAH7358280.1
hypothetical protein B0T11DRAFT_299362	190	2.00E-62	59%	82.30%	*Plectosphaerella cucumerina*	KAH7358281.1
virulence sensor protein bvgS	1386	0	99%	66.38%	*Plectosphaerella cucumerina*	KAH7358170.1
drug resistance transporter	588	0	99%	69.45%	*Plectosphaerella plurivora*	KAH6697430.1

phenolpthiocerol synthesis polyketide synthase ppsA	2325	0	99%	51.93%	*Sodiomyces alkalinus F11*	XP_028462492.1
aromatic prenyltransferase	456	0	100%	68.49%	*Plectosphaerella cucumerina*	KAH7358950.1
uncharacterized protein CGCS363_v012205	365	2.00E-104	93%	51.77%	*Colletotrichum siamense*	XP_036491211.1
hypothetical protein B0T11DRAFT_306916	372	2.00E-90	94%	62.89%	*Plectosphaerella cucumerina*	KAH7358952.1
hypothetical protein B0T11DRAFT_355575	250	2.00E-06	36%	46.94%	*Plectosphaerella cucumerina*	KAH7358954.1
SnodProt1	138	6.00E-67	100%	71.01%	*Plectosphaerella cucumerina*	KAH7368131.1
GATA zinc finger domain-containing protein 7	144	1.00E-16	31%	71.74%	*Colletotrichum tanaceti*	TKW48536.1
homoserine kinase	356	0	100%	90.17%	*Plectosphaerella cucumerina*	KAH7362595.1
hypothetical protein SODALDRAFT_67746	365	2.00E-61	98%	37.20%	*Sodiomyces alkalinus F11*	XP_028463289.1
hypothetical protein B0T11DRAFT_85809	195	7.00E-49	98%	59.28%	*Plectosphaerella cucumerina*	KAH7362593.1

119) *Verticillium zaregamsianum* contig_NMXM01000004, VRPKS-I-16 cluster

Gene name	Size	E value	% Coverage	% identity	Top blast hit	Accession number of top blast hit
hypothetical protein CEP52_001073	165	2.00E-30	98%	37.29%	*Fusarium sp. AF-4*	RSM14903.1
choline/ethanolaminephosphotransferase	427	0	92%	82.62%	*Sodiomyces alkalinus F11*	XP_028462783.1
Oxidoreductase BOA17	284	5.00E-118	99%	64.54%	*Colletotrichum siamense*	XP_036491827.1
glutaminyl-peptide cyclotransferase	388	0	100%	73.15%	*Sodiomyces alkalinus F11*	XP_028462781.1
Dolichyl-diphosphooligosaccharide--protein glycosyltransferase subunit SWP1	287	1.00E-91	99%	58.54%	*Colletotrichum sidae*	TEA10577.1
uncharacterized protein D7B24_005734	168	2.00E-26	87%	61.07%	*Verticillium nonalfalfae*	XP_028495810.1
hypothetical protein ACRE_054030	167	5.00E-62	99%	66.27%	*Acremonium chrysogenum ATCC 11550*	KFH43814.1
flavin depend monooxygenase that catalyses the oxidation of rubrofusarin to 9-hydroxyrubrofusarin	523	0	100%	78.48%	*Fusarium albosuccineum*	KAF4463211.1
xylosidase/arabinosidase	599	0	100%	90.65%	*Plectosphaerella plurivora*	KAH6670864.1
Alpha/Beta hydrolase protein	557	0	94%	65.91%	*Plectosphaerella plurivora*	KAH6688446.1
putative polyketide synthase	2602	0	99%	72.07%	*Plectosphaerella cucumerina*	KAH7369281.1
serine hydrolase FSH	239	3.00E-82	100%	57.14%	*Plectosphaerella plurivora*	KAH6673998.1
glycosyl hydrolase	638	0	98%	63.89%	*Colletotrichum truncatum*	XP_036587401.1
hypothetical protein B0T11DRAFT_249503	1065	0	100%	59.62%	*Plectosphaerella cucumerina*	KAH7375266.1
RNA recognition domain-containing protein	187	1.00E-64	72%	78.52%	*Colletotrichum higginsianum IMI 349063*	XP_018158756.1
hypothetical protein SODALDRAFT_157430	945	0	96%	70.59%	*Sodiomyces alkalinus F11*	XP_028467151.1

hypothetical protein B0T11DRAFT_7649	691	1.00E-73	94%	40.34%	*Plectosphaerella cucumerina*	KAH7375268.1
subtilase	859	0	97%	68.69%	*Plectosphaerella plurivora*	KAH6687843.1
Sorting nexin-4 like protein	475	0	100%	80.00%	*Sodiomyces alkalinus F11*	XP_028467149.1
mitochondrial distribution and morphology protein	542	0	94%	76.37%	*Plectosphaerella plurivora*	KAH6687845.1
adenylate kinase	268	6.00E-155	99%	80.60%	*Colletotrichum karsti*	XP_038745165.1
short chain dehydrogenase	313	7.00E-103	97%	55.02%	*Colletotrichum higginsianum IMI 349063*	XP_018159646.1

120) *Verticillium zaregamsianum* contig_NMXM01000004, VRPKS-I-25 cluster

Gene name	Size	E value	% Coverage	% identity	Top blast hit	Accession number of top blast hit
hypothetical protein B0T11DRAFT_325269	567	1.00E-162	97%	56.36%	*Plectosphaerella cucumerina*	KAH7366966.1
hypothetical protein B0T11DRAFT_49340	354	2.00E-114	92%	61.07%	*Plectosphaerella cucumerina*	KAH7366962.1
mediator complex, subunit Med18	269	2.00E-165	100%	80.51%	*Plectosphaerella cucumerina*	KAH7347312.1
hypothetical protein	451	0	99%	68.20%	*Sodiomyces alkalinus F11*	XP_028468241.1
hypothetical protein DL767_004432	1071	0	98%	74.11%	*Monosporascus sp. MG133*	RYP34097.1
Protein STB5-like protein 3	759	0	89%	62.25%	*Colletotrichum chlorophyti*	OLN81943.1
uncharacterized protein CGMCC3_g2624	492	0	98%	68.18%	*Colletotrichum fructicola*	XP_031891081.1
hypothetical protein	528	2.00E-161	87%	59.19%	*Colletotrichum incanum*	KZL63649.1
hypothetical protein CONLIGDRAFT_699567	318	9.00E-62	81%	44.83%	*Coniochaeta ligniaria NRRL 30616*	OIW32684.1
Non-reducing end alpha-L-arabinofuranosidase BoGH43A	603	0	95%	84.38%	*Colletotrichum siamense*	XP_036494585.1
hypothetical protein S40293_03578	2356	0	99%	91.30%	*Stachybotrys chartarum IBT 40293*	KFA46917.1
hypothetical protein S7711_03850	324	0	98%	86.25%	*Stachybotrys chartarum IBT 7711*	KEY68921.1
hypothetical protein S7711_03849	363	0	100%	85.79%	*Stachybotrys chartarum IBT 7711*	KEY68920.1
dihydroxy-acid dehydratase	627	0	99%	80.52%	*Plectosphaerella plurivora*	KAH6671001.1
S-adenosyl-L-methionine-dependent methyltransferase	313	4.00E-157	92%	81.79%	*Sodiomyces alkalinus F11*	XP_028466391.1
hypothetical protein SODALDRAFT_165672	372	1.00E-44	73%	45.98%	*Sodiomyces alkalinus F11*	XP_028466392.1
hypothetical protein CFIO01_00827	673	3.00E-152	91%	49.07%	*Colletotrichum fioriniae PJ7*	EXF83685.1
hypothetical protein FDECE_5916	187	3.00E-89	99%	66.31%	*Fusarium decemcellulare*	KAF5007792.1

glucosidase II beta subunit-like protein-domain-containing protein	524	0	87%	65.97%	*Plectosphaerella plurivora*	KAH6680057.1
hypothetical protein	1565	0	39%	69.53%	*Plectosphaerella plurivora*	KAH6680056.1

121) *Verticillium zaregamsianum* contig_NMXM01000003, PKS-III cluster

Gene name	Size	E value	% Coverage	% identity	Top blast hit	Accession number of top blast hit
hypothetical protein	289	1.00E-54	78%	45.11%	*Colletotrichum incanum*	OHW90569.1
carbonic anhydrase	271	7.00E-148	94%	76.95%	*Colletotrichum incanum*	KZL87566.1
Acetyltransferase	269	2.00E-150	88%	84.39%	*Colletotrichum higginsianum IMI 349063*	XP_018159255.1
oxidoreductase	451	0	99%	64.67%	*Plectosphaerella plurivora*	KAH6681205.1
Feruloyl esterase B	294	8.00E-153	94%	74.46%	*Colletotrichum orbiculare MAFF 240422*	TDZ25527.1
Transcriptional activator protein acu-15	804	0	100%	72.38%	*Colletotrichum higginsianum*	TID06918.1
TRAUB-domain-containing protein	587	5.00E-139	79%	74.43%	*Sodiomyces alkalinus F11*	XP_028470322.1
hypothetical protein K456DRAFT_1765144	127	2.00E-23	86%	59.82%	*Colletotrichum gloeosporioides 23*	KAH9228351.1
putative chalcone and stilbene synthase domain-containing protein	444	0	100%	75.06%	*Colletotrichum sublineola*	KDN65911.1
fructose-bisphosphate aldolase	362	0	100%	90.06%	*Plectosphaerella cucumerina*	KAH7375300.1
PAB-dependent poly-specific ribonuclease subunit PAN2	1082	0	98%	79.31%	*Plectosphaerella cucumerina*	KAH7375292.1
uncharacterized protein D7B24_007593	548	0	98%	67.68%	*Verticillium nonalfalfae*	XP_028494368.1
putative mitochondrial 2-oxoglutarate/malate carrier protein	335	0	100%	86.87%	*Colletotrichum chlorophyti*	OLN95994.1
hypothetical protein B0T11DRAFT_249550	636	3.00E-179	96%	62.56%	*Plectosphaerella cucumerina*	KAH7375296.1
Splicing factor spf30	303	1.00E-114	99%	69.71%	*Colletotrichum truncatum*	XP_036586172.1
Iron-sulfur protein IND1	296	7.00E-177	99%	79.59%	*Colletotrichum truncatum*	XP_036586173.1
centrin-3	189	8.00E-89	98%	66.31%	*Plectosphaerella plurivora*	KAH6681196.1
short chain dehydrogenase	301	1.00E-167	95%	79.02%	*Sodiomyces alkalinus F11*	XP_028470310.1
NADH-ubiquinone oxidoreductase 30.4 kDa subunit	499	3.00E-52	81%	49.62%	*Plectosphaerella plurivora*	KAH6681192.1
hypothetical protein Cob_v001966	579	1.00E-84	78%	39.66%	*Colletotrichum orbiculare MAFF 240422*	TDZ25446.1

122) *Verticillium zaregamsianum* contig_NMXM01000001, VRPKS-I-18 cluster

Gene name	Size	E value	% Coverage	% identity	Top blast hit	Accession number of top blast hit
MFS general substrate transporter	502	1.00E-163	91%	52.07%	*Oidiodendron maius Zn*	KIN03209.1
hypothetical protein B0T11DRAFT_325103	254	3.00E-116	98%	71.43%	*Plectosphaerella cucumerina*	KAH7366775.1
SGNH hydrolase	2316	0	43%	56.07%	*Plectosphaerella plurivora*	KAH6658772.1
uncharacterized protein BU25DRAFT_464600	391	3.00E-121	100%	51.60%	*Macroventuria anomochaeta*	XP_033567678.1
	535	0	97%	80.73%	*Decorospora gaudefroyi*	KAF1833741.1
carbohydrate-binding module family 50 protein	686	0	99%	59.10%	*Plectosphaerella plurivora*	KAH6684917.1
hypothetical protein B0T11DRAFT_337949	411	7.00E-93	90%	46.48%	*Plectosphaerella cucumerina*	KAH7368878.1
Reducing polyketide synthase PKS2	2857	0	79%	77.41%	*Fulvia fulva*	UJO18003.1
hypothetical protein CLAFUR5_06163	253	2.00E-133	98%	78.31%	*Fulvia fulva*	UJO18001.1
	453	0	90%	67.64%	*Plectosphaerella plurivora*	KAH6686639.1
general substrate transporter	518	0	99%	68.81%	*Dactylonectria macrodidyma*	KAH7160676.1
hypothetical protein ZTR_07120	526	0	98%	85.69%	*Talaromyces verruculosus*	KUL84026.1
secreted protein	211	4.00E-93	90%	76.44%	*Sodiomyces alkalinus F11*	XP_028465800.1
glycosyl hydrolase family 16 protein	425	1.00E-134	64%	70.80%	*Colletotrichum truncatum*	XP_036580125.1
Sec7 domain-containing protein	1558	0	97%	64.21%	*Plectosphaerella plurivora*	KAH6686645.1
hypothetical protein CFIO01_08750	437	3.00E-154	78%	65.51%	*Colletotrichum fioriniae PJ7*	EXF74680.1

123) *Verticillium zaregamsianum* contig_NMXM01000001, VRPKS-I-6 cluster

Gene name	Size	E value	% Coverage	% identity	Top blast hit	Accession number of top blast hit
COX17 protein	70	8.00E-23	71%	86.00%	*Diaporthaceae sp. PMI_573*	KAH8788412.1
hypothetical protein HYQ45_010318	159	4.00E-44	78%	62.40%	*Verticillium longisporum*	KAG7131019.1
protein PXR1	361	3.00E-72	49%	83.33%	*Plectosphaerella cucumerina*	KAH7368324.1
MFS sugar transporter	489	0	95%	75.05%	*Colletotrichum truncatum*	XP_036588620.1
hypothetical protein CSPAE12_03025	637	8.00E-133	80%	48.27%	*Colletotrichum incanum*	OHW98243.1
hypothetical protein C8035_v000460	326	6.00E-131	96%	64.26%	*Colletotrichum spinosum*	TDZ12713.1
hypothetical protein B0T10DRAFT_76705	206	3.00E-49	88%	48.78%	*Thelonectria olida*	KAH6887013.1
Transaldolase	365	0	100%	87.16%	*Colletotrichum siamense*	KAF4850344.1
membrane transporter D1	599	0	99%	82.58%	*Ilyonectria destructans*	KAH7006833.1
thioredoxin	328	4.00E-173	100%	73.78%	*Plectosphaerella cucumerina*	KAH7368325.1

hypothetical protein BDV96DRAFT_558457	184	5.00E-25	94%	34.43%	*Lophiotrema nucula*	KAF2106919.1
Dihydrolipoyllysine-residue acetyltransferase component of acetoin cleaving system	271	2.00E-119	97%	62.74%	*Colletotrichum siamense*	XP_036493445.1
serine hydrolase-domain-containing protein	284	5.00E-132	100%	63.73%	*Aspergillus avenaceus*	KAE8150729.1
hypothetical protein BDV25DRAFT_139560	4028	0	99%	67.20%	*Aspergillus avenaceus*	KAE8150730.1
hypothetical protein CC78DRAFT_576631	470	0	100%	64.47%	*Didymosphaeria enalia*	KAF2268082.1
P-loop containing nucleoside triphosphate hydrolase protein	1072	0	95%	58.36%	*Clohesyomyces aquaticus*	ORY13587.1
hypothetical protein	466	0	99%	62.77%	*Didymosphaeria enalia*	KAF2268085.1
cytochrome P450	531	0	97%	66.54%	*Aspergillus avenaceus*	KAE8150736.1
putative alcohol dehydrogenase	368	0	100%	77.08%	*Aspergillus avenaceus*	KAE8150737.1
cytochrome P450	477	0	96%	65.60%	*Aspergillus avenaceus*	KAE8150739.1

124) *Verticillium zaregamsianum* contig_NMXM01000002, VRPKS-I-7 cluster

Gene name	Size	E value	% Coverage	% identity	Top blast hit	Accession number of top blast hit
hypothetical protein B0I35DRAFT_444981	498	9.00E-05	36%	25.60%	*Stachybotrys elegans*	KAH7304539.1
Alpha/Beta hydrolase protein	496	3.00E-139	93%	50.94%	*Xylariaceae sp. FL0594*	KAI0391246.1
serine hydrolase domain-containing protein	255	3.00E-110	96%	67.48%	*Sarocladium implicatum*	KAH8169124.1
hypothetical protein LIA77_11249	622	5.00E-91	72%	49.67%	*Sarocladium implicatum*	KAH8169123.1
polyketide synthase dehydratase domain-containing protein	2690	0	96%	78.19%	*Sarocladium implicatum*	KAH8169122.1
major facilitator superfamily protein	491	0	97%	77.50%	*Sarocladium implicatum*	KAH8169121.1
aldose 1-epimerase domain-containing protein	427	0	98%	75.00%	*Sarocladium implicatum*	KAH8169120.1
aldose 1-epimerase domain-containing protein	452	0	92%	78.80%	*Sarocladium implicatum*	KAH8169120.1
cytochrome p450 domain-containing protein	539	0	100%	72.17%	*Sarocladium implicatum*	KAH8169119.1
hypothetical protein AK830_g5169	819	0	98%	57.03%	*Neonectria ditissima*	KPM41386.1
hypothetical protein AK830_g5170	734	0	98%	69.86%	*Neonectria ditissima*	KPM41355.1
hypothetical protein FGSG_03330	462	0	98%	65.65%	*Fusarium graminearum PH-1*	XP_011322406.1

125) *Verticillium zaregamsianum* contig_NMXM01000003, VRPKS-I-26 cluster

Gene name	Size	E value	% Coverage	% identity	Top blast hit	Accession number of top blast hit
hypothetical protein F5X68DRAFT_228683	78	6.00E-15	100%	62.82%	*Plectosphaerella plurivora*	KAH6692307.1

peptidase family C25-domain-containing protein	1019	0	99%	57.48%	*Paraphoma chrysanthemicola*	KAH7091334.1
hypothetical protein	578	0	96%	88.33%	*Colletotrichum higginsianum IMI 349063*	XP_018157666.1
Satratoxin biosynthesis SC1 cluster protein 4	375	5.00E-174	100%	68.11%	*Plectosphaerella cucumerina*	KAH7362744.1
uncharacterized protein KVR01_002788	522	4.00E-139	95%	43.90%	*Diaporthe batatas*	XP_044647803.1
putative pectate lyase F	236	3.00E-140	97%	83.98%	*Colletotrichum fructicola*	XP_031890093.1
Retinoic acid induced 16-like protein-domain-containing protein	1004	0	100%	73.21%	*Plectosphaerella cucumerina*	KAH7362743.1
hypothetical protein B0T11DRAFT_318355	163	1.00E-54	98%	65.85%	*Plectosphaerella cucumerina*	KAH7362742.1
uncharacterized protein KVR01_004169	223	9.00E-06	25%	45.61%	*Diaporthe batatas*	XP_044646333.1
hypothetical protein F5B20DRAFT_586947	2349	0	99%	52.43%	*Whalleya microplaca*	KAI1073750.1
TTL domain-containing protein	473	0	100%	73.44%	*Coniochaeta sp. 2T2.1*	KAB5518934.1
retinol dehydrogenase	312	0	100%	78.21%	*Plectosphaerella plurivora*	KAH6675343.1
peptidase family M28	372	0	100%	78.76%	*Dactylonectria estremocensis*	KAH7144232.1
uncharacterized protein PODANS_6_2250	216	7.00E-30	80%	41.71%	*Podospora anserina S mat+*	XP_001910274.1
heterokaryon incompatibility protein-domain-containing protein	742	0	100%	77.63%	*Chaetomium sp. MPI-CAGE-AT-0009*	KAH6847694.1
FAD-dependent monooxygenase	453	0	100%	84.55%	*Stachybotrys elegans*	KAH7303249.1
glycosyl transferase	427	3.00E-135	77%	58.26%	*Trichoderma arundinaceum*	RFU78228.1
alpha-l-rhamnosidase	898	0	97%	68.11%	*Colletotrichum truncatum*	XP_036586343.1

126) *Verticillium zaregamsianum* contig_NMXM01000003, VNRPKS-I-2 cluster

Gene name	Size	E value	% Coverage	% identity	Top blast hit	Accession number of top blast hit
putative monooxygenase	501	0	74%	87.67%	*Monosporascus ibericus*	RYP01441.1
PKS16 protein	2126	0	100%	84.68%	*Monosporascus ibericus*	RYP01440.1
putative MFS multidrug transporter	514	0	97%	85.52%	*Monosporascus ibericus*	RYP01439.1
hypothetical protein CEP54_016375	209	2.00E-23	66%	38.93%	*Fusarium sp. AF-8*	RSL39030.1
NmrA-like family protein	292	6.00E-105	98%	55.48%	*Ilyonectria sp. MPI-CAGE-AT-0026*	KAH6955623.1
hypothetical protein DL768_001135	453	0	99%	79.65%	*Monosporascus sp. mg162*	RYP53985.1
amidohydrolase 3	674	0	94%	78.02%	*Colletotrichum karsti*	XP_038751725.1
major facilitator superfamily domain-containing protein	466	0	99%	73.77%	*Fusarium zealandicum*	KAF4979810.1
alpha/beta fold hydrolase	131	1.00E-26	97%	35.15%	*Zasmidium cellare ATCC 36951*	XP_033665360.1
Alpha/Beta hydrolase protein	299	0	98%	79.73%	*Fusarium sarcochroum*	KAF4949579.1

cellobiose dehydrogenase	538	0	95%	76.12%	*Plectosphaerella plurivora*	KAH6685971.1
general substrate transporter	521	0	100%	82.73%	*Plectosphaerella plurivora*	KAH6695665.1
hypothetical protein FSARC_5046	565	0	98%	58.82%	*Fusarium sarcochroum*	KAF4967388.1
general substrate transporter	534	0	94%	79.49%	*Corynespora cassiicola Philippines*	PSN67207.1
hypothetical protein BS50DRAFT_553126	285	5.00E-159	100%	76.57%	*Corynespora cassiicola Philippines*	PSN67208.1

127) *Verticillium zaregamsianum* contig_NMXM01000009, VRPKS-I-14 cluster

Gene name	Size	E value	% Coverage	% identity	Top blast hit	Accession number of top blast hit
lipolytic G-D-S-L family	1445	0	97%	49.08%	*Cylindrodendrum hubeiense*	KAF7556154.1
hypothetical protein FALBO_210	142	6.00E-62	86%	75.61%	*Fusarium albosuccineum*	KAF4472888.1
hypothetical protein HYQ45_016732	88	0.002	100%	70.33%	*Verticillium longisporum*	KAG7113540.1
FK506-binding protein	191	9.00E-76	100%	69.27%	*Plectosphaerella cucumerina*	KAH7367672.1
hypothetical protein SODALDRAFT_267625	465	0	98%	79.69%	*Sodiomyces alkalinus F11*	XP_028471380.1
Vanillyl-alcohol oxidase	562	0	99%	86.40%	*Plectosphaerella plurivora*	KAH6673908.1
hypothetical protein B0T11DRAFT_56928	204	1.00E-99	100%	72.46%	*Plectosphaerella cucumerina*	KAH7367670.1
short-chain dehydrogenase/reductase	339	2.00E-118	94%	57.05%	*Cenococcum geophilum 1.58*	OCK87093.1
hypothetical protein CEP53_002056	374	1.00E-149	97%	69.86%	*Fusarium sp. AF-6*	RSL69861.1
cytochrome P450	506	0	100%	79.25%	*Plectosphaerella cucumerina*	KAH7367666.1
major facilitator superfamily domain-containing protein	584	0	81%	63.52%	*Truncatella angustata*	XP_045957260.1
putative polyketide synthase	2898	0	100%	56.98%	*Truncatella angustata*	XP_045957259.1
fatty acid desaturase domain-containing protein	610	0	100%	64.34%	*Sarocladium implicatum*	KAH8177350.1
Acyl-CoA dehydrogenase/oxidase	603	0	93%	50.09%	*Ophiocordyceps polyrhachis-furcata BCC 54312*	RCI16331.1
hypothetical protein CGLO_06291	489	1.00E-124	98%	39.75%	*Colletotrichum gloeosporioides Cg-14*	EQB53932.1

128) *Verticillium zaregamsianum* contig_NMXM01000006, VRPKS-I-21 cluster

Gene name	Size	E value	% Coverage	% identity	Top blast hit	Accession number of top blast hit
NAD-binding protein	305	3.00E-111	87%	64.54%	*Sodiomyces alkalinus F11*	XP_028469385.1
hypothetical protein SODALDRAFT_331329	213	4.00E-36	53%	57.52%	*Sodiomyces alkalinus F11*	XP_028469386.1
uncharacterized protein CkaCkLH20_05965	592	3.00E-92	89%	37.17%	*Colletotrichum karsti*	XP_038746018.1
translation initiation factor SUI1	119	5.00E-59	100%	84.87%	*Sodiomyces alkalinus F11*	XP_028469384.1

nucleotide exchange factor SIL1	422	3.00E-131	92%	60.46%	*Plectosphaerella cucumerina*	KAH7359363.1
nadh-ubiquinone oxidoreductase kda subunit	169	1.00E-77	100%	67.84%	*Colletotrichum incanum*	KZL88236.1
hypothetical protein LI328DRAFT_141063	434	4.00E-06	43%	28.28%	*Trichoderma asperelloides*	KAH8128511.1
40s ribosomal protein s15	153	6.00E-88	84%	100.00%	*Colletotrichum truncatum*	XP_036583553.1
cytochrome p450 domain-containing protein	507	0	100%	84.28%	*Sarocladium implicatum*	KAH8171308.1
Lovastatin diketide synthase LovF 2	2331	0	100%	74.50%	*Colletotrichum chlorophyti*	OLN91972.1
hypothetical protein SODALDRAFT_331321	352	1.00E-36	94%	36.02%	*Sodiomyces alkalinus F11*	XP_028469379.1
Monothiol glutaredoxin-7	266	1.00E-83	98%	63.60%	*Colletotrichum chlorophyti*	OLN81123.1
hypothetical protein BN1723_009283	137	0.012	87%	38.46%	*Verticillium longisporum*	CRK11148.1
glutathione-dependent formaldehyde-activating	207	1.00E-44	99%	48.56%	*Plectosphaerella plurivora*	KAH6682279.1
hypothetical protein G7046_g1361	1253	0	97%	48.50%	*Stylonectria norvegica*	KAF7562768.1
Arsenical-resistance protein Acr3	384	0	97%	80.48%	*Colletotrichum viniferum*	KAF4919918.1
uncharacterized protein GCG54_00009924	566	0	99%	58.97%	*Colletotrichum gloeosporioides*	XP_045271398.1
cystathionine gamma-lyase	421	0	96%	91.89%	*Colletotrichum tofieldiae*	KZL65728.1
hypothetical protein SODALDRAFT_377226	466	0	99%	69.65%	*Sodiomyces alkalinus F11*	XP_028469373.1
hypothetical protein K445DRAFT_313536	319	5.00E-140	92%	67.91%	*Daldinia sp. EC12*	OTB19762.1

129) *Verticillium zaregamsianum* contig_NMXM01000013, VRPKS-I-19 cluster

Gene name	Size	E value	% Coverage	% identity	Top blast hit	Accession number of top blast hit
vacuolar protein sorting-associated protein	161	7.00E-13	63%	50.48%	*Plectosphaerella cucumerina*	KAH7368195.1
putative beta-glucosidase G	832	0	99%	68.89%	*Aspergillus sydowii CBS 593.65*	XP_040708017.1
cutinase-2	224	1.00E-131	92%	87.44%	*Plectosphaerella cucumerina*	KAH7368196.1
cytochrome P450	544	2.00E-119	91%	40.84%	*Ilyonectria destructans*	KAH7009626.1
uncharacterized protein CkaCkLH20_03768	1128	0	99%	67.87%	*Colletotrichum karsti*	XP_038748329.1
uncharacterized protein GLRG_06876	1117	0	98%	72.47%	*Colletotrichum graminicola M1.001*	XP_008095607.1
Type I Polyketide synthase	2141	0	99%	75.93%	*Leptosphaeria biglobosa*	KAH9873394.1
hypothetical protein E4U21_006806	297	2.00E-108	99%	57.38%	*Claviceps maximensis*	KAG6006661.1
MFS general substrate transporter	574	0	98%	70.00%	*Sodiomyces alkalinus F11*	XP_028468956.1
thioredoxin-like protein	162	8.00E-86	98%	77.36%	*Hypoxylon sp. FL0890*	KAI0834638.1

hypothetical protein AK830_g4742	498	7.00E-78	83%	40.90%	*Neonectria ditissima*	KPM41855.1
hypothetical protein F5882DRAFT_368496	392	7.00E-53	92%	31.28%	*Hyaloscypha sp. PMI_1271*	KAH8752665.1
exopolygalacturonase	469	0	94%	71.59%	*Monosporascus sp. mg162*	RYP54678.1
uncharacterized protein PgNI_04980	394	0	100%	89.85%	*Pyricularia grisea*	XP_030984371.1
DeSI-like protein sdu1	235	1.00E-114	99%	75.97%	*Colletotrichum orbiculare MAFF 240422*	TDZ21862.1
hypothetical protein CFIO01_06157	250	2.00E-91	100%	56.40%	*Colletotrichum fioriniae PJ7*	EXF75199.1
TIM44 subunit of mitochondria import inner membrane translocase	541	0	100%	75.92%	*Sodiomyces alkalinus F11*	XP_028468965.1
hypothetical protein B0J18DRAFT_6692	111	6.00E-27	76%	61.63%	*Chaetomium sp. MPI-SDFR-AT-0129*	KAH6634037.1
hypothetical protein ACRE_064810	288	3.00E-85	95%	49.46%	*Acremonium chrysogenum ATCC 11550*	KFH42767.1
hypothetical protein SODALDRAFT_333514	77	3.00E-21	97%	55.13%	*Sodiomyces alkalinus F11*	XP_028465568.1
Alpha-glucosides permease MPH3 like protein	542	0	99%	75.23%	*Cylindrodendrum hubeiense*	KAF7546071.1

130) *Verticillium zaregamsianum* contig_NMXM01000005, VRPKS-I-12 and VNPKS-I-1 clusters

Gene name	Size	E value	% Coverage	% identity	Top blast hit	Accession number of top blast hit
transcription factor Cmr1	925	0	99%	72.20%	*Colletotrichum musicola*	KAF6845250.1
hypothetical protein	424	1.00E-145	100%	56.41%	*Colletotrichum incanum*	KZL86702.1
uncharacterized protein	325	0	99%	85.19%	*Colletotrichum truncatum*	XP_036586400.1
Conidial pigment polyketide synthase	2188	0	100%	79.05%	*Colletotrichum camelliae*	KAH0426510.1
laccase-like multicopper oxidase	561	0	98%	67.33%	*Plectosphaerella cucumerina*	KAH7358155.1
uncharacterized protein CTRU02_08118	316	0.002	62%	25.65%	*Colletotrichum truncatum*	XP_036582041.1
hypothetical protein SODALDRAFT_331196	388	2.00E-129	99%	63.92%	*Sodiomyces alkalinus F11*	XP_028469270.1
L-2,4-diaminobutyrate decarboxylase	514	0	87%	65.11%	*Colletotrichum trifolii*	TDZ67276.1
polyketide synthase	3891	0	99%	75.46%	*Sodiomyces alkalinus F11*	XP_028469272.1
hypothetical protein SODALDRAFT_331204	888	0	86%	70.04%	*Sodiomyces alkalinus F11*	XP_028469275.1
FAD/NAD-binding domain-containing protein	428	0	93%	74.00%	*Sodiomyces alkalinus F11*	XP_028469276.1
eukaryotic aspartyl protease	432	0	96%	56.97%	*Colletotrichum simmondsii*	KXH41725.1
beta-ketoacyl synthase	366	2.00E-90	100%	47.06%	*Thelonectria olida*	KAH6899712.1
cytochrome P450	484	0	94%	75.11%	*Sodiomyces alkalinus F11*	XP_028469280.1
hypothetical protein FPOAC1_009761	238	2.00E-82	89%	59.53%	*Fusarium poae*	XP_044706852.1

131) *Verticillium zaregamsianum* contig_NMXM01000008, VRPKS-I-5 cluster

Gene name	Size	E value	% Coverage	% identity	Top blast hit	Accession number of top blast hit

hypothetical protein jhhlp_008516	813	0	99%	75.61%	*Lomentospora prolificans*	PKS05149.1
Isochorismatase-like protein	244	2.00E-127	90%	77.48%	*Plectosphaerella plurivora*	KAH6676014.1
hypothetical protein B0T11DRAFT_333834	160	9.00E-32	63%	64.71%	*Plectosphaerella cucumerina*	KAH7347765.1
DUF636 domain-containing protein	137	1.00E-67	100%	81.75%	*Metarhizium acridum*	KAG8415691.1
hypothetical protein F4775DRAFT_593279	4017	0	99%	65.91%	*Biscogniauxia sp. FL1348*	KAI0597589.1
hypothetical protein B0I37DRAFT_378309	506	3.00E-64	62%	58.22%	*Chaetomium sp. MPI-CAGE-AT-0009*	KAH6844803.1
hypothetical protein BGAL_0184g00240	199	6.00E-60	89%	53.33%	*Botrytis galanthina*	THV49682.1
major facilitator superfamily-domain-containing protein	612	0	89%	72.68%	*Biscogniauxia sp. FL1348*	KAI0597572.1
MFS general substrate transporter	428	1.00E-137	92%	67.25%	*Glarea lozoyensis ATCC 20868*	XP_008088685.1
glutathione S-transferase domain-containing protein	224	1.00E-122	99%	76.68%	*fungal sp. No.14919*	GAW23379.1
hypothetical protein SODALDRAFT_41197	1629	0	98%	71.49%	*Sodiomyces alkalinus F11*	XP_028471323.1
GPI-GlcNAc transferase complex	222	3.00E-46	97%	62.67%	*Plectosphaerella cucumerina*	KAH7347756.1
pseudouridine synthase	604	0	96%	52.15%	*Sodiomyces alkalinus F11*	XP_028471325.1

132) *Verticillium zaregamsianum* contig_NMXM01000001, VRPKS-I-1 cluster

Gene name	Size	E value	% Coverage	% identity	Top blast hit	Accession number of top blast hit
glycogen debranching enzyme	447	0	100%	79.06%	*Dactylonectria macrodidyma*	KAH7115576.1
thiol-specific monooxygenase	507	0	99%	76.94%	*Plectosphaerella plurivora*	KAH6686480.1
major facilitator superfamily domain-containing protein	579	0	91%	69.43%	*Xylariales sp. PMI_506*	KAH8666575.1
hypothetical protein BX600DRAFT_462019	343	2.00E-80	66%	54.55%	*Xylariales sp. PMI_506*	KAH8666574.1
hypothetical protein	194	2.00E-44	96%	37.23%	*Botryosphaeria dothidea*	KAF4312163.1
hypothetical protein CDV55_104319	360	9.00E-107	98%	48.02%	*Aspergillus turcosus*	RHZ60657.1
Alpha/Beta hydrolase protein	468	5.00E-117	99%	45.44%	*Aspergillus leporis*	KAB8069129.1
hypothetical protein VF21_05751	334	6.00E-06	33%	32.48%	*Pseudogymnoascus sp. 05NY08*	OBT75937.1
hypothetical protein G6514_000112	315	3.00E-66	96%	39.35%	*Epicoccum nigrum*	KAG9206825.1
uncharacterized protein PAC_08242	439	1.00E-112	94%	42.21%	*Phialocephala subalpina*	CZR58350.1
hypothetical protein	4122	0	99%	44.80%	*Didymosphaeria enalia*	KAF2268646.1
ABC transporter FUM19 like protein	1410	0	99%	77.02%	*Plectosphaerella cucumerina*	KAH7359175.1
hypothetical protein FDECE_14782	473	9.00E-07	34%	31.71%	*Fusarium decemcellulare*	KAF4989247.1

hypothetical protein S40285_07973	380	4.00E-148	96%	62.93%	*Stachybotrys chlorohalonata IBT 40285*	KFA69565.1
S-adenosyl-L-methionine-dependent methyltransferase	420	1.00E-130	95%	49.01%	*Emericellopsis atlantica*	XP_046116985.1
hypothetical protein FDECE_1566	598	0	95%	80.07%	*Fusarium decemcellulare*	KAF5012339.1
conserved hypothetical protein	447	0	99%	69.73%	*Uncinocarpus reesii 1704*	XP_002583601.1

133) *Verticillium zaregamsianum* contig_NMXM01000001, VRPKS-I-6 cluster

Gene name	Size	E value	% Coverage	% identity	Top blast hit	Accession number of top blast hit
hypothetical protein B0T11DRAFT_270545	719	0	99%	62.10%	*Plectosphaerella cucumerina*	KAH7375570.1
hypothetical protein F5X68DRAFT_47703	256	1.00E-63	89%	47.37%	*Plectosphaerella plurivora*	KAH6693919.1
hypothetical protein B0T11DRAFT_86728	585	0	99%	67.50%	*Plectosphaerella cucumerina*	KAH7362695.1
2OG-Fe oxygenase family protein	332	8.00E-158	99%	70.39%	*Colletotrichum incanum*	OHW94343.1
Uracil permease like protein	507	0	97%	82.76%	*Colletotrichum camelliae*	KAH0420306.1
uncharacterized protein HER10_EVM0008554	108	4.00E-16	76%	73.49%	*Colletotrichum scovillei*	XP_035337453.1
hypothetical protein jhhlp_008882	194	8.00E-54	96%	49.21%	*Lomentospora prolificans*	PKS05504.1
hypothetical protein FALBO_5956	314	0	100%	85.99%	*Fusarium albosuccineum*	KAF4467173.1
polyketide synthase	3176	0	99%	55.53%	*Fusarium avenaceum*	KIL85244.1
hypothetical protein BT63DRAFT_423135	196	2.00E-22	82%	69.57%	*Microthyrium microscopicum*	KAF2670841.1
endo-1,4-beta-glucanase	297	2.00E-94	81%	59.18%	*Colletotrichum sojae*	KAF6788143.1
epoxide hydrolase	409	0	98%	62.94%	*Plectosphaerella plurivora*	KAH6662678.1
hypothetical protein SODALDRAFT_4980	186	4.00E-45	78%	60.96%	*Sodiomyces alkalinus F11*	XP_028469806.1
endoglucanase-1	233	6.00E-134	98%	78.60%	*Plectosphaerella cucumerina*	KAH7362705.1
major facilitator superfamily domain-containing protein	583	0	100%	85.25%	*Plectosphaerella cucumerina*	KAH7368056.1
glycogen debranching enzyme	447	0	100%	79.06%	*Dactylonectria macrodidyma*	KAH7115576.1

SUPPLEMENTARY FILE S2
(Sayari et al - Verticillium Polyketide biosynthesis gene clusters)

The tables below show the top 10 tblastn hits NCBI's non-redundant nucleotide database for each of the *Verticillium* PKS genes examined in this study. The blast scores (E-value, percent sequence identity and coverage) and the Accession number of the top hits are indicated.

Verticillium dahliae contig NW_009276940, VRPKS-I-22	Species of BLAST hit	% coverage	% identity	E-value	Accession number
R-PKS-I	*Fusarium miscanthi*	99	70.93	0	ALQ32877
R-PKS-I	*Fusarium denticulatum*	100	70.66	0	KAF5687147
R-PKS-I	*Fusarium mexicanum*	99	70.83	0	KAF5539484
R-PKS-I	*Fusarium acutatum*	99	71.09	0	KAF4428951
R-PKS-I	*Fusarium sp*	99	71	0	KAF5020446
R-PKS-I	*Fusarium subglutinans*	99	70.79	0	XP_036534733
R-PKS-I	*Fusarium pseudoanthophilum*	100	70.86	0	KAF5573817
R-PKS-I	*Fusarium sarcochroum*	100	70.64	0	KAF4944666
R-PKS-I	*Fusarium sacchari*	99	70.94	0	ALQ32937
R-PKS-I	*Fusarium aywerte*	100	70.25	0	ALQ32768

2)

Verticillium dahliae contig NW_009276940, VRPKS-I-23	Species of BLAST hit	% coverage	% identity	E-value	Accession number
R-PKS-I	*Sodiomyces alkalinus*	99	49.71	0	XP_028462492
R-PKS-I	*Colletotrichum gloeosporio*	97	43.70	0	KAF3797175
R-PKS-I	*Colletotrichum karsti*	97	43.50	0	XP_038741012
R-PKS-I	*Colletotrichum chlorophyti*	98	43.08	0	OLN85715
R-PKS-I	*Colletotrichum aenigma*	97	43.02	0	XP_037174401
R-PKS-I	*Colletotrichum fructicola*	97	43.45	0	XP_031892623
R-PKS-I	*Colletotrichum fructicola*	97	43.45	0	KAF5488909
R-PKS-I	*Colletotrichum incanum*	99	43.42	0	OHW97630
R-PKS-I	*Colletotrichum viniferum*	97	43.19	0	KAF4918834
R-PKS-I	*Colletotrichum tropicale*	97	43.31	0	KAF4828937

Verticillium dahliae contig NW_009276935, VRPKS-I-26	Species of BLAST hit	% coverage	% identity	E-value	Accession number
R-PKS-I	*Monosporascus sp*	98	62.71	0	RYP50633
R-PKS-I	*Arthrinium phaeospermum*	99	60.26	0	P0CU84
R-PKS-I	*Verticillium longisporum*	80	87.35	0	CRK37551
R-PKS-I	*Verticillium longisporum*	77	91.73	0	CRK33507

R-PKS-I	*Didymosphaeria enalia*	98	52.79	0	KAF2259783
R-PKS-I	*Lachnellula willkommii*	98	53.11	0	TVY89575
R-PKS-I	*Lophiotrema nucula*	98	52.27	0	KAF2119266
R-PKS-I	*Lachnellula arida*	98	52.92	0	TVY19100
R-PKS-I	*Rutstroemia sp.*	98	53.70	0	PQE16274
R-PKS-I	*Aspergillus puulaa*	98	52.25	0	XP_041560187

Verticillium dahliae contig NW_009276935, PKS-III	Species of BLAST hit	% coverage	% identity	E-value	Accession number
PKS-III	*Colletotrichum chlorophyti*	100	72.01	0	OLN95986
PKS-III	*Colletotrichum tanaceti*	100	72.69	0	TKW57803
PKS-III	*Colletotrichum tofieldiae*	100	72.69	0	KZL74511
PKS-III	*Colletotrichum sublineola*	100	71.62	0	KDN65911
PKS-III	*Colletotrichum incanum*	100	72.46	0	KZL80557
PKS-III	*Colletotrichum graminicola*	100	71.56	0	XP_008099452
PKS-III	*Colletotrichum incanum*	100	72.23	0	OHW96035
PKS-III	*Colletotrichum simmondsii*	100	71.40	0	KXH46773
PKS-III	*Colletotrichum camelliae*	99	72.69	0	KAG2100071
PKS-III	*Colletotrichum karsti*	100	72.81	0	XP_038745402

Verticillium dahliae contig NW_009276938, VRPKS-I-19	Species of BLAST hit	% coverage	% identity	E-value	Accession number
R-PKS-I	*Claviceps purpurea*	99	61.92	0	KAG6168136
R-PKS-I	*Claviceps purpurea*	99	61.83	0	KAG6264852
R-PKS-I	*Claviceps purpurea*	99	61.83	0	KAG6172346
R-PKS-I	*Claviceps purpurea*	99	61.78	0	KAG6313294
R-PKS-I	*Claviceps purpurea*	99	61.78	0	KAG6257628
R-PKS-I	*Claviceps purpurea*	99	61.87	0	KAG6178588.

R-PKS-I	*Claviceps purpurea*	99	61.83	0	KAG6319079
R-PKS-I	*Claviceps purpurea*	99	61.83	0	KAG6144460
R-PKS-I	*Claviceps purpurea*	99	61.74	0	KAG6278039
R-PKS-I	*Claviceps purpurea*	99	61.74	0	KAG6303654

Verticillium dahliae contig NW_009276923, VRPKS-I-1	Species of BLAST hit	% coverage	% identity	E-value	Accession number
PKS-NRPS	*Massariosphaeria phaeospora*	99	44.38	0	KAF2874833
PKS-NRPS	*Phialocephala subalpina*	99	43.26	0	CZR58354
PKS-NRPS	*Coccidioides posadasii str. Silveira*	99	43.55	0	EFW16137
PKS-NRPS	*Coccidioides immitis*	99	43.44	0	TPX24000
PKS-NRPS	*Coccidioides immitis*	99	43.37	0	XP_001242733
PKS-NRPS	*Coccidioides posadasii*	99	43.40	0	XP_003069897
PKS-NRPS	*Pseudogymnoascus sp.*	99	43.07	0	KFZ23096
PKS-NRPS	*Saccharata proteae*	99	43.26	0	KAF2084690
PKS-NRPS	*Pseudogymnoascus sp*	99	43.05	0	KFZ13457
PKS-NRPS	*Pseudogymnoascus sp.*	99	42.92	0	KFX98753

Verticillium dahliae contig NW_009276921, VNRPKS-I-1	Species of BLAST hit	% coverage	% identity	E-value	Accession number
NR-PKS-I	*Colletotrichum karsti*	100	76.45	0	XP_038751444
NR-PKS-I	*Colletotrichum higginsianum*	100	75.74	0	TIC92505
NR-PKS-I	*Colletotrichum higginsianum*	100	75.74	0	XP_018155915
NR-PKS-I	*Colletotrichum higginsianum*	100	75.70	0	CCF45141
NR-PKS-I	*Colletotrichum gloeosporioides*	100	76.19	0	EQB55056
NR-PKS-I	*Colletotrichum gloeosporioides*	100	76.14	0	KAF3809075
NR-PKS-I	*Colletotrichum tropicale*	100	76.23	0	KAF4827098

NR-PKS-I	*Colletotrichum asianum*	100	76.19	0	KAF0322599
NR-PKS-I	*Colletotrichum fructicola*	100	76.14	0	KAF4937522
NR-PKS-I	*Colletotrichum siamense*	100	76.09	0	KAF4812766

Verticillium dahliae contig NW_009276930, VRPKS-I-8	Species of BLAST hit	% coverage	% identity	E-value	Accession number
R-PKS-I	*Stemphylium lycopersici*	95	77.88	0	RAR12984
R-PKS-I	*Stemphylium lycopersici*	95	78.17	0	RAR08647
R-PKS-I	*Stemphylium lycopersici*	95	78.17	0	KNG48001
R-PKS-I	*Fusarium babinda*	95	76.25	0	ALQ32784
R-PKS-I	*Fusarium mundagurra*	94	78.24	0	KAF5711130
R-PKS-I	*Fusarium sp.*	93	79.39	0	ALQ32968
R-PKS-I	*Fusarium tjaetaba*	94	78.78	0	XP_037202727
R-PKS-I	*Fusarium napiforme*	93	78.78	0	KAF5554444
R-PKS-I	*Fusarium verticillioides*	94	78.57	0	XP_018757761
R-PKS-I	*Fusarium verticillioides*	94	78.57	0	RBQ85559

Verticillium dahliae contig NW_009276967, VRPKS-I-17	Species of BLAST hit	% coverage	% identity	E-value	Accession number
R-PKS-I	*Colletotrichum chlorophyti*	99	70.46	0	OLN86478
R-PKS-I	*Colletotrichum truncatum*	99	69.83	0	XP_036585096
R-PKS-I	*Colletotrichum orbiculare*	99	69.69	0	TDZ25780
R-PKS-I	*Colletotrichum trifolii*	99	69.63	0	TDZ28230
R-PKS-I	*Colletotrichum sidae*	99	69.60	0	TEA18683
R-PKS-I	*Colletotrichum spinosum*	99	69.56	0	TDZ31026
R-PKS-I	*Colletotrichum musicola*	99	69.63	0	KAF6830768
R-PKS-I	*Colletotrichum fioriniae*	99	69.87	0	EXF85385
R-PKS-I	*Colletotrichum salicis*	99	69.75	0	KXH66496

R-PKS-I	*Colletotrichum scovillei*	99	69.75	0	XP_035338972

Verticillium dahliae contig NW_009276941, VNRPKS-I-3	Species of BLAST hit	% coverage	% identity	E-value	Accession number
NR-PKS-NRPS	*Sodiomyces alkalinus*	76	45.29	0	XP_028463040
NR-PKS-NRPS	*Colletotrichum chlorophyti*	21	86.95	0	OLN93090
NR-PKS-NRPS	*Tolypocladium ophioglossoides*	76	59.97	0	KND89106
NR-PKS-NRPS	*Colletotrichum tofieldiae*	21	87.79	0	KZL65137
NR-PKS-NRPS	*Colletotrichum incanum*	21	87.79	0	KZL68616
NR-PKS-NRPS	*Colletotrichum sublineol*	21	88.01	0	KDN66279
NR-PKS-NRPS	*Colletotrichum simmonds*	21	86.72	0	KXH41766
NR-PKS-NRPS	*Colletotrichum orchidophilum*	21	86.45	0	XP_022475083
NR-PKS-NRPS	*Colletotrichum musicola*	21	86.55	0	KAF6802064
NR-PKS-NRPS	*Colletotrichum sojae*	22	85.22	0	KAF6811701

Verticillium dahliae contig NW_009276917, VRPKS-I-11	Species of BLAST hit	% coverage	% identity	E-value	Accession number
R-PKS-I	*Colletotrichum incanum*	72	65.47	0	KZL81424
R-PKS-I	*Hirsutella minnesotensis*	95	61.28	6e-150	KJZ73816
R-PKS-I	*Ophiocordyceps sinensis*	31	68.91	2e-140	EQK98692
R-PKS-I	*Ophiocordyceps sinensis*	34	66.23	1e-136	KAF4511963
R-PKS-I	*Aspergillus novofumigatus*	95	52.79	1e-121	XP_024677790
R-PKS-I	*Pseudomassariella vexata*	94	52.43	6e-115	XP_040720636
R-PKS-I	*Metarhizium majus*	94	54.57	2e-113	KID93355
R-PKS-I	*Monilinia fructicola*	95	51.84	9e-112	KAG4026558
R-PKS-I	*Monilinia fructicola*	95	51.84	1e-111	KAA8566095
R-PKS-I	*Pochonia chlamydosporia*	95	53.40	1e-111	KAG8400261

Verticillium albo-atrum contig NMXJ01000004, VRPKS-I-22	Species of BLAST hit	% coverage	% identity	E-value	Accession number
R-PKS-I	*Hyaloscypha variabilis*	91	46.58	0	PMD43053
R-PKS-I	*Aulographum hederae*	92	46.88	0	KAF1988946
R-PKS-I	*Cadophora sp. DSE1049*	91	46.34	0	PVH88801
R-PKS-I	*Hyaloscypha bicolor*	91	44.78	0	XP_024734639
R-PKS-I	*Scytalidium lignicola*	91	39.87	0	RFU24364
R-PKS-I	*Cadophora sp.*	91	45.77	0	KAG4444512
R-PKS-I	*Cadophora malorum*	91	45.56	0	KAG4423991
R-PKS-I	*Tothia fuscella*	91	45.01	0	KAF2419578
R-PKS-I	*Fusarium oxysporum*	91	38.30	0	RKK77696
R-PKS-I	*Hyaloscypha hepaticicola*	91	45.77	0	PMD16306

Verticillium albo-atrum contig NMXJ01000009, VRPKS-I-1	Species of BLAST hit	% coverage	% identity	E-value	Accession number
PKS-NRPS	*Colletotrichum fructicola*	100	86.09	0	KAF4886526
PKS-NRPS	*Colletotrichum fructicola*	100	86.04	0	XP_031875985
PKS-NRPS	*Metarhizium majus*	99	63.96	0	KID93652
PKS-NRPS	*Metarhizium guizhouense*	99	63.91	0	KID82736
PKS-NRPS	*Metarhizium robertsii*	99	63.80	0	XP_007826303
PKS-NRPS	*Metarhizium robertsii*	99	63.80	0	EXU95862
PKS-NRPS	*Xylaria grammica*	98	61.25	0	RWA11947
PKS-NRPS	*Thermothelomyces thermophilus*	97	59.79	0	XP_003662442
PKS-NRPS	*Talaromyces atroroseus*	99	58.90	0	XP_020120442
PKS-NRPS	*Coccidioides immitis*	99	56.84	0	XP_001242733

Verticillium albo-atrum contig NMXJ01000001, VRPKS-I-12	Species of BLAST hit	% coverage	% identity	E-value	Accession number
PKS-NRPS	*Sodiomyces alkalinus*	97	74.31	0	XP_028469272
PKS-NRPS	*Colletotrichum nymphaeae*	97	72.10	0	KXH30697
PKS-NRPS	*Colletotrichum salicis*	97	72.20	0	KXH33485
PKS-NRPS	*Colletotrichum scovillei*	97	72.05	0	XP_035338903
PKS-NRPS	*Colletotrichum fioriniae*	97	71.92	0	EXF80071
PKS-NRPS	*Colletotrichum simmondsii*	97	72.10	0	KXH41720
PKS-NRPS	*Colletotrichum orchidophilum*	97	71.76	0	XP_022470047
PKS-NRPS	*Pochonia chlamydosporia*	97	66.01	0	XP_018147207
PKS-NRPS	*Cylindrodendrum hubeiense*	97	65.60	0	KAF7549858
PKS-NRPS	*Neonectria ditissima*	97	65.91	0	KPM42068

Verticillium albo-atrum contig NMXJ01000001, VNRPKS-I-1	Species of BLAST hit	% coverage	% identity	E-value	Accession number
NR-PKS-I	*Colletotrichum fructicola*	99	79.18	0	KAF4937522
NR-PKS-I	*Colletotrichum asianum*	99	79.18	0	KAF0322599
NR-PKS-I	*Colletotrichum viniferum*	99	79.09	0	KAF4919737
NR-PKS-I	*Colletotrichum gloeosporioides*	99	79.18	0	KAF3809075
NR-PKS-I	*Colletotrichum karsti*	99	79.27	0	XP_038751444
NR-PKS-I	*Colletotrichum siamense*	99	79.13	0	KAF4840476
NR-PKS-I	*Colletotrichum aenigma*	99	79.13	0	XP_037173402
NR-PKS-I	*Colletotrichum fructicola*	99	79.13	0	XP_031880204
NR-PKS-I	*Colletotrichum fructicola*	99	79.13	0	KAF4912855
NR-PKS-I	*Colletotrichum siamense*	99	79.13	0	XP_036489117

Verticillium albo-atrum contig NMXJ01000001, VRPKS-I-9	Species of BLAST hit	% coverage	% identity	E-value	Accession number
PKS-NRPS	*Monosporascus sp*	99	49.78	0	RYP45085
PKS-NRPS	*Hirsutella minnesotensis*	98	68.09	0	KJZ73816
PKS-NRPS	*Pseudomassariella vexata*	86	47.30	0	XP_040720636
PKS-NRPS	*Metarhizium guizhouense*	90	44.54	0	KID82218
PKS-NRPS	*Metarhizium majus*	90	44.75	0	KID93355
PKS-NRPS	*Metarhizium anisopliae*	90	44.41	0	KJK77205
PKS-NRPS	*Metarhizium anisopliae*	89	44.40	0	KFG80765
PKS-NRPS	*Metarhizium robertsii*	89	44.01	0	XP_007817815
PKS-NRPS	*Metarhizium brunneum*	90	44.03	0	XP_014539400
PKS-NRPS	*Pseudogymnoascus sp*	99	40.17	0	OBT39531

Verticillium albo-atrum contig NMXJ01000001, VRPKS-I-17	Species of BLAST hit	% coverage	% identity	E-value	Accession number
R-PKS-I	*Colletotrichum truncatum*	99	71.66	0	XP_036585096
R-PKS-I	*Colletotrichum chlorophyti*	99	72.28	0	OLN86478
R-PKS-I	*Colletotrichum trifolii*	99	71.63	0	TDZ28230
R-PKS-I	*Colletotrichum orbiculare*	99	71.67	0	TDZ25780
R-PKS-I	*Colletotrichum sidae*	99	71.63	0	TEA18683
R-PKS-I	*Colletotrichum spinosum*	99	71.58	0	TDZ31026
R-PKS-I	*Colletotrichum sojae*	99	71.43	0	KAF6806435
R-PKS-I	*Colletotrichum nymphaeae*	99	71.58	0	KXH49587
R-PKS-I	*Colletotrichum musicola*	99	71.52	0	KAF6830768
R-PKS-I	*Colletotrichum fioriniae*	99	71.47	0	EXF85385

Verticillium albo-atrum contig NMXJ01000001, VRPKS-I-23	Species of BLAST hit	% coverage	% identity	E-value	Accession number

R-PKS-I	*Sodiomyces alkalinus*	89	51.24	0	XP_028462492
R-PKS-I	*Colletotrichum incanum*	87	44.61	0	OHW97630
R-PKS-I	*Colletotrichum gloeosporioides*	86	44.53	0	KAF3797175
R-PKS-I	*Colletotrichum aenigma*	86	44.87	0	XP_037174401
R-PKS-I	*Colletotrichum siamense*	86	44.72	0	XP_036490386
R-PKS-I	*Colletotrichum tropicale*	86	44.68	0	KAF4828937
R-PKS-I	*Colletotrichum asianum*	86	44.57	0	KAF0329849
R-PKS-I	*Colletotrichum fructicola*	86	44.57	0	XP_031892623
R-PKS-I	*Colletotrichum tanaceti*	87	44.96	0	TKW51389
R-PKS-I	*Colletotrichum fructicola*	86	44.57	0	KAF5488909

Verticillium albo-atrum contig NMXJ01000003, VRPKS-I-25	Species of BLAST hit	% coverage	% identity	E-value	Accession number
R-PKS-I	*Stachybotrys chartarum*	88	73.91	0	KEY68922
R-PKS-I	*Stachybotrys chartarum*	99	73.91	0	KFA46917
R-PKS-I	*Physcia stellaris*	98	41.19	0	KAG7001446
R-PKS-I	*Stachybotrys chartarum*	97	71.92	0	KFA72420
R-PKS-I	*Monosporascus sp.*	98	55.77	0	RYP80513
R-PKS-I	*Monosporascus ibericus*	99	55.20	0	RYP07817
R-PKS-I	*Scytalidium lignicola*	98	55.68	0	RFU27489
R-PKS-I	*Chlorociboria aeruginascens*	98	54.57	0	TAQ85507
R-PKS-I	*Monosporascus cannonballus*	97	55.07	0	RYO84498
R-PKS-I	*Monosporascus sp.*	97	55.07	0	RYP15912

Verticillium albo-atrum contig NMXJ01000003, VRPKS-I-24	Species of BLAST hit	% coverage	% identity	E-value	Accession number
R-PKS-I	*Cylindrodendrum habeiensis*	99	63.30	0	KAF7552999
R-PKS-I	*Hyaloscypha variabilis*	99	62.39	0	PMD46834

R-PKS-I	*Viridothelium virens*	100	55.16	0	KAF2232763
R-PKS-I	*Zopfia rhizophila*	100	55.78	0	KAF2195930
R-PKS-I	*Pseudogymnoascus sp.*	99	54.53	0	KFY28507
R-PKS-I	*Trichoderma arundinaceum*	100	53.08	0	RFU79720
R-PKS-I	*Zopfia rhizophila*	100	54.25	0	KAF2195889
R-PKS-I	*Ascochyta lentis*	99	53.19	0	KAF9696958
R-PKS-I	*Letharia columbiana*	99	51.32	0	XP_037161903
R-PKS-I	*Cladonia uncialis subsp. uncialis*	99	51.33	0	ANM86582

Verticillium albo-atrum contig NMXJ01000003, VRPKS-I-19	Species of BLAST hit	% coverage	% identity	E-value	Accession number
R-PKS-I	*Epichloe festucae*	99	63.26	0	QPH02303
R-PKS-I	*Claviceps pusilla*	99	62.97	0	KAG6001262
R-PKS-I	*Claviceps purpurea*	99	63.01	0	KAG6209081
R-PKS-I	*Claviceps purpurea*	99	63.06	0	KAG6144460
R-PKS-I	*Metarhizium rileyi*	99	62.73	0	OAA35905
R-PKS-I	*Claviceps purpurea*	99	62.86	0	KAG6152537
R-PKS-I	*Claviceps purpurea*	99	62.91	0	KAG6172346
R-PKS-I	*Claviceps purpurea*	99	62.96	0	KAG6178588
R-PKS-I	*Claviceps purpurea*	99	62.96	0	KAG6168136
R-PKS-I	*Claviceps purpurea*	99	63.01	0	KAG6319079

Verticillium albo-atrum contig NMXJ01000006, VRPKS-I-5	Species of BLAST hit	% coverage	% identity	E-value	Accession number
PKS-NRPS	*Botrytis tulipae*	100	62.50	0	TGO20046
PKS-NRPS	*Botryotinia calthae*	100	62.25	0	TEY65541
PKS-NRPS	*Botrytis galanthina*	99	62.19	0	THV46179
PKS-NRPS	*Chlorociboria aeruginascens*	99	49.32	0	TAQ90374

PKS-NRPS	*Rutstroemia sp.*	100	44.64	0	PQE13487
PKS-NRPS	*Rutstroemia sp.*	100	44.49	0	PQE27755
PKS-NRPS	*Monosporascus sp.*	99	43.46	0	RYP90391
PKS-NRPS	*Penicillium italicum*	99	42.91	0	KGO65245
PKS-NRPS	*Polytolypa hystricis*	99	43.16	0	PGH21386
PKS-NRPS	*Chalara longipes*	99	43.37	0	KAE9373525

Verticillium albo-atrum contig NMXJ01000007, VRPKS-I-26	Species of BLAST hit	% coverage	% identity	E-value	Accession number
R-PKS-I	*Monosporascus sp. mg162*	78	56.29	0	RYP50633
R-PKS-I	*Arthrinium phaeospermum*	75	56.29	0	P0CU84
R-PKS-I	*Lasiodiplodia theobromae*	74	40.54	0	XP_035363749
R-PKS-I	*Colletotrichum gloeosporioides*	19	70.92	0	KAF3800899
R-PKS-I	*Colletotrichum camelliae*	19	70.38	0	KAG2086927
R-PKS-I	*Colletotrichum gloeosporioides*	19	70.92	0	EQB58169
R-PKS-I	*Colletotrichum karst*	19	70.85	0	XP_038744071
R-PKS-I	*Coniochaeta sp.*	19	73.20	0	KAB5518934
R-PKS-I	*Colletotrichum aenigma*	19	70.71	0	XP_037177975
R-PKS-I	*Colletotrichum plurivorum*	19	70.55	0	KAF6834567

Verticillium albo-atrum contig NMXJ01000002, VRPKS-I-3	Species of BLAST hit	% coverage	% identity	E-value	Accession number
PKS-NRPS	*Colletotrichum truncatum*	86	67.40	0	XP_036576988
PKS-NRPS	*Colletotrichum higginsianum*	86	63.17	0	AFI23580
PKS-NRPS	*Colletotrichum higginsianum*	86	62.87	0	XP_018161565
PKS-NRPS	*Colletotrichum tofie*	86	62.52	0	KZL75527
PKS-NRPS	*Colletotrichum spinosum*	86	61.78	0	TDZ28122

PKS-NRPS	*Colletotrichum orbiculare*	86	61.61	0	TDZ24970
PKS-NRPS	*Colletotrichum sidae*	86	61.66	0	TEA13957
PKS-NRPS	*Colletotrichum camelliae*	86	61.94	0	KAG2095187
PKS-NRPS	*Colletotrichum trifolii*	86	61.28	0	TDZ60804
PKS-NRPS	*Colletotrichum viniferum*	86	61.96	0	KAF4926162

Verticillium albo-atrum contig NMXJ01000005, PKS-III	Species of BLAST hit	% coverage	% identity	E-value	Accession number
PKS-III	*Colletotrichum simmondsii*	100	73.79	0	KXH46773
PKS-III	*Colletotrichum tofieldiae*	100	74.67	0	KZL74511
PKS-III	*Colletotrichum chlorophyti*	100	73.79	0	OLN95986
PKS-III	*Colletotrichum scovillei*	100	73.57	0	XP_035333635
PKS-III	*Colletotrichum incanum*	100	74.45	0	KZL80557
PKS-III	*Colletotrichum sublineola*	100	74.01	0	KDN65911
PKS-III	*Colletotrichum graminicola*	100	74.89	0	XP_008099452
PKS-III	*Colletotrichum incanum*	100	74.23	0	OHW96035
PKS-III	*Colletotrichum higginsianum*	100	74.01	0	XP_018159249
PKS-III	*Colletotrichum tanaceti*	100	74.01	0	TKW57803

Verticillium alfalfae contig NW_003315037, VRPKS-I-16	Species of BLAST hit	% coverage	% identity	E-value	Accession number
R-PKS-I	*Fusarium miscanthi*	100	70.92	0	ALQ32877
R-PKS-I	*Fusarium denticulatum*	100	71.25	0	KAF5687147
R-PKS-I	*Fusarium mexicanum*	97	70.97	0	KAF5539484
R-PKS-I	*Fusarium acutatum*	98	72.09	0	KAF4428951
R-PKS-I	*Fusarium sp*	99	73.41	0	KAF5020446
R-PKS-I	*Fusarium subglutinans*	100	71.68	0	XP_036534733
R-PKS-I	*Fusarium pseudoanthophilum*	99	70.89	0	KAF5573817
R-PKS-I	*Fusarium sarcochroum*	100	71.52	0	KAF4944666
R-PKS-I	*Fusarium sacchari*	100	71.63	0	ALQ32937
R-PKS-I	*Fusarium aywerte*	100	69.78	0	ALQ32768

Verticillium alfalfae contig NW_003315037, VRPKS-I-17	Species of BLAST hit	% coverage	% identity	E-value	Accession number
R-PKS-I	*Sodiomyces alkalinus*	98	50.06	0	XP_028462492
R-PKS-I	*Colletotrichum gloeosporio*	96	44.56	0	KAF3797175
R-PKS-I	*Colletotrichum karsti*	98	43.45	0	XP_038741012
R-PKS-I	*Colletotrichum chlorophyti*	98	44.56	0	OLN85715
R-PKS-I	*Colletotrichum aenigma*	96	44	0	XP_037174401
R-PKS-I	*Colletotrichum fructicola*	99	42.98	0	XP_031892623
R-PKS-I	*Colletotrichum fructicola*	98	44.32	0	KAF5488909
R-PKS-I	*Colletotrichum incanum*	99	41.85	0	OHW97630
R-PKS-I	*Colletotrichum viniferum*	96	45.85	0	KAF4918834
R-PKS-I	*Colletotrichum tropicale*	95	44.09	0	KAF4828937

Verticillium alfalfae contig NW_003315034, VRPKS-I-9	Species of BLAST hit	% coverage	% identity	E-value	Accession number
PKS-NRPS	*Acremonium chrysogenum*	99	44.13	0	KFH45364
PKS-NRPS	*Pseudomassariella vexata*	98	41.40	0	XP_040711562
PKS-NRPS	*Lasiodiplodia theobromae*	99	39.48	0	KAF9630276
PKS-NRPS	*Lasiodiplodia theobromae*	99	39.45	0	KAB2573439
PKS-NRPS	*Lasiodiplodia theobromae*	99	39.23	0	XP_035374836
PKS-NRPS	*Aplosporella prunicola*	99	39.41	0	XP_033401020
PKS-NRPS	*Elaphomyces granulatus*	99	38.46	0	OXV07567
PKS-NRPS	*Talaromyces stipitatus*	99	37.01	0	XP_002478535
PKS-NRPS	*Aspergillus carbonarius*	99	37.77	0	OOF95055
PKS-NRPS	*Aspergillus sclerotioniger*	99	37.99	0	XP_025471949

Verticillium alfalfae contig NW_003315034, VRPKS-I-5	Species of BLAST hit	% coverage	% identity	E-value	Accession number
PKS-NRPS	*Massariosphaeria phaeospora*	99	44.38	0	KAF2874833
PKS-NRPS	*Phialocephala subalpina*	99	43.26	0	CZR58354
PKS-NRPS	*Coccidioides posadasii str. Silveira*	99	43.55	0	EFW16137
PKS-NRPS	*Coccidioides immitis*	99	43.44	0	TPX24000

PKS-NRPS	*Coccidioides immitis*	99	43.37	0	XP_001242733
PKS-NRPS	*Coccidioides posadasii*	99	43.40	0	XP_003069897
PKS-NRPS	*Pseudogymnoascus sp.*	99	43.07	0	KFZ23096
PKS-NRPS	*Saccharata proteae*	99	43.26	0	KAF2084690
PKS-NRPS	*Pseudogymnoascus sp*	99	43.05	0	KFZ13457
PKS-NRPS	*Pseudogymnoascus sp.*	99	42.92	0	KFX98753

Verticillium alfalfae contig NW_003315038, VRPKS-I-19	Species of BLAST hit	% coverage	% identity	E-value	Accession number
R-PKS-I	*Colletotrichum chlorophyti*	99	71.25	0	OLN86478
R-PKS-I	*Colletotrichum truncatum*	99	70	0	XP_036585096
R-PKS-I	*Colletotrichum orbiculare*	99	69.58	0	TDZ25780
R-PKS-I	*Colletotrichum trifolii*	99	70.69	0	TDZ28230
R-PKS-I	*Colletotrichum sidae*	99	68.98	0	TEA18683
R-PKS-I	*Colletotrichum spinosum*	99	69	0	TDZ31026
R-PKS-I	*Colletotrichum musicola*	99	69.69	0	KAF6830768
R-PKS-I	*Colletotrichum fioriniae*	99	70.35	0	EXF85385
R-PKS-I	*Colletotrichum salicis*	99	70.55	0	KXH66496
R-PKS-I	*Colletotrichum scovillei*	99	68.39	0	XP_035338972

Verticillium alfalfae contig NW_003315038, VNRPKS-I-1	Species of BLAST hit	% coverage	% identity	E-value	Accession number
NR-PKS-I	*Colletotrichum karsti*	99	79.25	0	XP_038751444
NR-PKS-I	*Colletotrichum higginsianum*	99	79.26	0	TIC92505
NR-PKS-I	*Colletotrichum higginsianum*	99	79.18	0	XP_018155915
NR-PKS-I	*Colletotrichum higginsianum*	99	79.18	0	CCF45141
NR-PKS-I	*Colletotrichum gloeosporioides*	99	79.39	0	EQB55056

NR-PKS-I	*Colletotrichum gloeosporioides*	99	79.37	0	KAF3809075
NR-PKS-I	*Colletotrichum tropicale*	99	79.88	0	KAF4827098
NR-PKS-I	*Colletotrichum asianum*	99	79.12	0	KAF0322599
NR-PKS-I	*Colletotrichum fructicola*	99	79.19	0	KAF4937522
NR-PKS-I	*Colletotrichum siamense*	99	76.27	0	KAF4812766

Verticillium alfalfae contig NW_003315038, VRPKS-I-1	Species of BLAST hit	% coverage	% identity	E-value	Accession number
PKS-NRPS	*Massariosphaeria phaeospora*	99	52.92	0	KAF2874833
PKS-NRPS	*Phialocephala subalpina*	99	52.91	0	CZR58354
PKS-NRPS	*Coccidioides posadasii str. Silveira*	99	51.19	0	EFW16137
PKS-NRPS	*Coccidioides immitis*	99	51.92	0	TPX24000
PKS-NRPS	*Coccidioides immitis*	99	55.77	0	XP_001242733
PKS-NRPS	*Coccidioides posadasii*	99	55.20	0	XP_003069897
PKS-NRPS	*Pseudogymnoascus sp.*	99	55.68	0	KFZ23096
PKS-NRPS	*Saccharata proteae*	99	54.57	0	KAF2084690
PKS-NRPS	*Pseudogymnoascus sp*	99	55.07	0	KFZ13457
PKS-NRPS	*Pseudogymnoascus sp.*	99	55.07	0	KFX98753

) *Verticillium alfalfae* contig NW_003315032, VRPKS-I-9	Species of BLAST hit	% coverage	% identity	E-value	Accession number
R-PKS-I	*Claviceps citina*	100	67.40	0	KAG6041353
R-PKS-I	*Claviceps purpurea*	100	63.17	0	KAG6264852
R-PKS-I	*Claviceps purpurea*	100	62.87	0	KAG6172346
R-PKS-I	*Claviceps purpurea*	100	62.52	0	KAG6313294
R-PKS-I	*Claviceps purpurea*	100	61.78	0	KAG6257628
R-PKS-I	*Claviceps purpurea*	100	61.61	0	KAG6178588.

R-PKS-I	*Claviceps purpurea*	100	61.66	0	KAG6319079
R-PKS-I	*Claviceps purpurea*	100	61.94	0	KAG6144460
R-PKS-I	*Claviceps purpurea*	100	61.28	0	KAG6278039
R-PKS-I	*Claviceps purpurea*	100	61.96	0	KAG6303654

34) *Verticillium alfalfae* contig NW_003315025, VRPKS-I-26

Verticillium alfalfae contig NW_003315025, VRPKS-I-26	**Species of BLAST hit**	**% coverage**	**% identity**	**E-value**	**Accession number**
R-PKS-I	*Monosporascus sp*	95	65.31	0	RYP50633
R-PKS-I	*Arthrinium phaeospermum*	94	62.55	0	P0CU84
R-PKS-I	*Verticillium longisporum*	94	62.28	0	CRK37551
R-PKS-I	*Verticillium longisporum*	94	61.71	0	CRK33507
R-PKS-I	*Didymosphaeria enalia*	94	61.33	0	KAF2259783
R-PKS-I	*Lachnellula willkommii*	94	61.24	0	TVY89575
R-PKS-I	*Lophiotrema nucula*	94	60.47	0	KAF2119266
R-PKS-I	*Lachnellula arida*	94	60.21	0	TVY19100
R-PKS-I	*Rutstroemia sp.*	94	59.88	0	PQE16274
R-PKS-I	*Aspergillus puulaa*	94	58.57	0	XP_041560187

Verticillium alfalfae contig NW_003315023, NR-PKS-I-B	**Species of BLAST hit**	**% coverage**	**% identity**	**E-value**	**Accession number**
NR-PKS-NRPS	*Tolypocladium ophioglossoides*	94	56.33	0	KND89106
NR-PKS-NRPS	*Sodiomyces alkalinus*	94	55.21	0	XP_028463040
NR-PKS-NRPS	*Colletotrichum chlorophyti*	92	55.20	0	OLN93090
NR-PKS-NRPS	*Colletotrichum tofieldiae*	91	56.21	0	KZL65137
NR-PKS-NRPS	*Colletotrichum incanum*	86	56.89	0	KZL68616
NR-PKS-NRPS	*Colletotrichum sublineol*	86	56.74	0	KDN66279
NR-PKS-NRPS	*Colletotrichum simmonds*	85	56.34	0	KXH41766

NR-PKS-NRPS	*Colletotrichum orchidophilum*	83	55.34	0	XP_022475083
NR-PKS-NRPS	*Colletotrichum musicola*	82	56.21	0	KAF6802064
NR-PKS-NRPS	*Colletotrichum sojae*	82	55.51	0	KAF6811701

Verticillium alfalfae contig NW_003315025, PKS-III	**Species of BLAST hit**	**% coverage**	**% identity**	**E-value**	**Accession number**
PKS-III	*Colletotrichum chlorophyti*	100	72.57	0	OLN95986
PKS-III	*Colletotrichum tanaceti*	100	72.56	0	TKW57803
PKS-III	*Colletotrichum tofieldiae*	100	72.54	0	KZL74511
PKS-III	*Colletotrichum sublineola*	100	71.89	0	KDN65911
PKS-III	*Colletotrichum incanum*	100	72.12	0	KZL80557
PKS-III	*Colletotrichum graminicola*	100	71.06	0	XP_008099452
PKS-III	*Colletotrichum incanum*	100	72.14	0	OHW96035
PKS-III	*Colletotrichum simmondsii*	100	71.48	0	KXH46773
PKS-III	*Colletotrichum camelliae*	100	71.35	0	KAG2100071
PKS-III	*Colletotrichum karsti*	100	71.29	0	XP_038745402

*V. isaacii*_VRPKS-I-16_contig_NMXN01000002					
Gene name	Species of blast hit	E value	% Coverage	% identity	Accession number
putative polyketide synthase	*Plectosphaerella cucumerina*	0.00E+00	99%	71.74%	KAH7369281.1
putative polyketide synthase	*Plectosphaerella plurivora*	0	99%	71.18%	KAH6673997.1
hypothetical protein K402DRAFT_461387	*Aulographum hederae CBS 113979*	0.00E+00	99%	49.49%	KAF1988946.1
hypothetical protein L207DRAFT_422368	*Hyaloscypha variabilis F*	0	99%	48.19%	PMD43053.1
hypothetical protein DL98DRAFT_477463	*Cadophora sp. DSE1049*	0	99%	47.60%	PVH88801.1
uncharacterized protein K444DRAFT_592521	*Hyaloscypha bicolor E*	0.00E+00	99%	47.60%	XP_024734639.1
hypothetical protein IFR05_000103	*Cadophora sp. M221*	0.00E+00	99%	46.99%	KAG4444512.1
putative polyketide synthase	*Tothia fuscella*	0.00E+00	99%	47.12%	KAF2419578.1
hypothetical protein NA56DRAFT_673334	*Hyaloscypha hepaticicola*	0.00E+00	99%	45.16%	PMD16306.1
putative polyketide synthase	*Leptodontidium sp. MPI-SDFR-AT-0119*	0	98%	43.93%	KAH6715845.1

V. isaacii _ VRPKS-I-23_contig_NMXN01000005					
Gene name	*Species of blast hit*	E value	% Coverage	% identity	Accession number
phenolpthiocerol synthesis polyketide synthase ppsA	*Sodiomyces alkalinus F11*	0	99%	51.79%	XP_028462492.1
polyketide synthase	*Verticillium nonalfalfae*	0	95%	65.66%	XP_028490525.1
phenolpthiocerol synthesis polyketide synthase ppsA	*Plectosphaerella plurivora*	0.00E+00	99%	45.55%	KAH6697431.1
phenolpthiocerol synthesis polyketide synthase ppsA	*Plectosphaerella cucumerina*	0.00E+00	99%	45.01%	KAH7358949.1
beta-ketoacyl synthase domain-containing protein	*Colletotrichum incanum*	0	99%	45.17%	OHW97630.1
polyketide synthase (beta-ketoacyl synthase domain-containing protein)	*Colletotrichum tofieldiae*	0	98%	45.40%	KZL67886.1
Lovastatin diketide synthase LovF 13	*Colletotrichum chlorophyti*	0	99%	44.36%	OLN85715.1
beta-ketoacyl synthase domain-containing protein	*Colletotrichum graminicola M1.001*	0	98%	45.60%	XP_008090783.1
beta-ketoacyl synthase domain-containing protein	*Colletotrichum karsti*	0.00E+00	98%	44.69%	XP_038741012.1
Reducing polyketide synthase FUB1	*Colletotrichum gloeosporioides*	0.00E+00	98%	45.28%	XP_045256339.1

*V. isaacii*_ VRPKS-I-26_contig_NMXN01000012					
Gene name	Species of blast hit	E value	% Coverage	% identity	Accession number
hypothetical protein F5B20DRAFT_586947	Whalleya microplaca	0.00E+00	99%	52.38%	KAI1073750.1
hypothetical protein DL768_003915	Monosporascus sp. mg162	0	99%	51.54%	RYP50633.1
Ketol-acid reductoisomerase	Verticillium dahliae VDG2	0.00E+00	86%	68.15%	KAF3349326.1
RecName: Full=Highly reducing polyketide synthase apmlA; Short=HRPKS apmlA; AltName: Full=Phaeospelide A biosynthesis cluster protein apmlA	Apiospora sphaerosperma	0	99%	50.43%	P0CU84.1
hypothetical protein BN1708_007417	Verticillium longisporum	0.00E+00	87%	57.83%	CRK37551.1
hypothetical protein BN1708_001184	Verticillium longisporum	0	85%	57.57%	CRK33507.1
D-amino-acid oxidase	Verticillium dahliae VDG1	0.00E+00	81%	70.15%	KAF3356735.1
reducing type I polyketide synthase	Xylariomycetidae sp. FL2044	0	99%	43.07%	KAH9902283.1
hypothetical protein ACN42_g5244	Penicillium freii	0	98%	43.13%	KUM61888.1
reducing type I polyketide synthase	Annulohypoxylon maeteangense	0	98%	42.91%	KAI0883761.1

*V. isaacii*_ VRPKS-I-19_contig_NMXN01000007					

Gene name	Species of blast hit	E value	% Coverage	% identity	Accession number
hypothetical protein J1614_005792	*Leptosphaeria biglobosa*	0	99%	77.43%	KAH9873394.1
Type I Polyketide synthase	*Verticillium nonalfalfae*	0	100%	76.45%	XP_028494609.1
Reducing polyketide synthase PKS2 like protein	*Verticillium longisporum*	0	100%	76.49%	KAG7143635.1
hypothetical protein VD0004_g7884	*Verticillium dahliae*	0	100%	76.35%	PNH38983.1
fatty acid synthase S-acetyltransferase	*Verticillium dahliae*	0	100%	76.45%	KAH6703869.1
fatty acid synthase S-acetyltransferase	*Verticillium dahliae VdLs.17*	0	100%	75.80%	XP_009655626.1
hypothetical protein BN1708_003179	*Verticillium longisporum*	0	100%	76.01%	CRK18834.1
Inositol 2-dehydrogenase 2	*Verticillium dahliae VDG2*	0	96%	76.17%	KAF3346614.1
hypothetical protein BN1708_009954	*Verticillium longisporum*	0	88%	75.80%	CRK10893.1
hypothetical protein VDGD_03466	*Verticillium dahliae*	0	87%	75.97%	RBQ87991.1

*V. isaacii*_ VRPKS-I-18_contig_NMXN01000010					
Gene name	Species of blast hit	E value	% Coverage	% identity	Accession number
Reducing polyketide synthase PKS2	*Fulvia fulva*	0	99%	75.10%	UJO18003.1
Polyketide synthase	*Tolypocladium paradoxum*	0	99%	67.33%	POR36838.1
Polyketide synthase	*Tolypocladium capitatum*	0	99%	66.42%	PNY26863.1
polyketide synthase	*Thelonectria olida*	0	99%	64.31%	KAH6887502.1
hypothetical protein FDECE_8493	*Fusarium decemcellulare*	0	99%	63.44%	KAF5005076.1
fatty acid synthase S-acetyltransferase	*Trichoderma harzianum*	0	99%	63.98%	KKO98752.1
Lovastatin diketide synthase mokB	*Trichoderma lentiforme*	0	99%	63.76%	KAF3065508.1
hypothetical protein THARTR1_06994	*Trichoderma harzianum*	0	99%	63.43%	PNP52390.1
hypothetical protein TgHK011_004790	*Trichoderma gracile*	0	99%	62.72%	KAH0497490.1
Type I Iterative PKS	*Trichoderma virens FT-333*	0	99%	62.69%	UKZ74406.1

*V. isaacii*_ VRPKS-I-17_contig_NMXN01000005					
Gene name	Species of blast hit	E value	% Coverage	% identity	Accession number
Reducing polyketide synthase PKS2 like protein	*Plectosphaerella cucumerina*	9.00E-46	83%	75.53%	KAH7363092.1
Reducing polyketide synthase PKS2 like protein	*Sodiomyces alkalinus F11*	3.00E-45	79%	78.89%	XP_028466984.1
Reducing polyketide synthase PKS2 like protein	*Plectosphaerella plurivora*	9.00E-45	83%	75.53%	KAH6686280.1
Reducing polyketide synthase PKS2 like protein	*Colletotrichum truncatum*	2.00E-38	77%	70.65%	XP_036582859.1
Reducing polyketide synthase PKS2 like protein	*Colletotrichum fructicola*	3.00E-37	80%	66.32%	XP_031886881.1

Reducing polyketide synthase PKS2 like protein	*Colletotrichum karsti*	8.00E-37	76%	71.11%	XP_038750642.1
Reducing polyketide synthase PKS2 like protein	*Fusarium oxysporum f. sp. lycopersici 4287*	5.00E-36	82%	62.89%	XP_018234365.1
Reducing polyketide synthase PKS2 like protein	*Fusarium subglutinans*	8.00E-36	82%	62.89%	XP_036539494.1
Reducing polyketide synthase PKS2 like protein	*Colletotrichum tofieldiae*	2.00E-35	82%	66.34%	KZL71328.1
Reducing polyketide synthase PKS2 like protein	*Colletotrichum graminicola M1.001*	2.00E-35	82%	66.34%	XP_008091934.1

V._isaacii_PKS-I-F2_contig_NMXN01000005					
Gene name	Species of blast hit	E value	% Coverage	% identity	Accession number
Lovastatin diketide synthase LovF 16	*Colletotrichum chlorophyti*	0.00E+00	99%	71.71%	OLN86478.1
polyketide synthase	*Colletotrichum truncatum*	0.00E+00	99%	71.24%	XP_036585096.1
Reducing polyketide synthase PKS2	*Colletotrichum trifolii*	0	99%	71.28%	TDZ28230.1
fatty acid synthase S-acetyltransferase	*Verticillium alfalfae VaMs.102*	0	99%	82.69%	XP_003008481.1
Reducing polyketide synthase PKS2	*Colletotrichum orbiculare MAFF 240422*	0	99%	71.24%	TDZ25780.1
Reducing polyketide synthase PKS2	*Colletotrichum sidae*	0.00E+00	99%	71.19%	TEA18683.1
Reducing polyketide synthase PKS2	*Colletotrichum spinosum*	0	99%	71.15%	TDZ31026.1
polyketide synthase	*Colletotrichum sojae*	0	99%	71.09%	KAF6806435.1
polyketide synthase	*Colletotrichum musicola*	0	99%	71.30%	KAF6830768.1
beta-ketoacyl synthase domain-containing protein	*Colletotrichum orchidophilum*	0	99%	71.24%	XP_022471279.

*V. isaacii_* VRPKS-I-13_contig_NMXN01000010					
Gene name	Species of blast hit	E value	% Coverage	% identity	Accession number
polyketide synthase	*Fusarium avenaceum*	0	99%	53.42%	KIL85244.1
hypothetical protein BKA59DRAFT_387557	*Fusarium tricinctum*	0	99%	52.99%	KAH7261591.1
hypothetical protein DER45DRAFT_513901	*Fusarium avenaceum*	0.00E+00	99%	53.14%	KAH6968136.1
polyketide synthase	*Fusarium beomiforme*	0.00E+00	99%	52.70%	KAF4341752.1
putative polyketide synthase	*Fusarium aywerte*	0	99%	52.88%	ALQ32765.1
polyketide synthase	*Fusarium sporotrichioides*	0	99%	52.75%	RGP68838.1
unnamed protein product	*Fusarium graminearum*	0.00E+00	99%	52.56%	VTO93732.1
unnamed protein product	*Fusarium graminearum*	0	99%	52.46%	CAG2011682.1
unnamed protein product	*Fusarium graminearum*	0.00E+00	99%	52.49%	CZS84488.1
unnamed protein product	*Fusarium graminearum*	0.00E+00	99%	52.56%	CAF3490384.1

*V. isaacii_* VRPKS-I-20_contig_NMXN01000006					
Gene name	Species of blast hit	E value	% Coverage	% identity	Accession number
polyketide synthase	*Ophiocordyceps camponoti-floridani*	0	70%	47.07%	KAF4594413.1
hypothetical protein PENARI_c014G02915	*Penicillium arizonense*	0	69%	65.85%	XP_022486633.1
Bifunctional cytochrome reductase like protein	*Verticillium longisporum*	0.00E+00	30%	92.67%	KAG7141503.1
hypothetical protein BN1708_000330	*Verticillium longisporum*	0	30%	92.67%	CRJ80656.1
bifunctional P-450:NADPH-P450 reductase	*Verticillium alfalfae VaMs.102*	0.00E+00	30%	91.08%	XP_003004414.1
uncharacterized protein D7B24_002818	*Verticillium nonalfalfae*	0	30%	91.08%	XP_028490996.1
Bifunctional cytochrome reductase like protein	*Verticillium longisporum*	0	30%	91.80%	KAG7140967.1
bifunctional P-450:NADPH-P450 reductase	*Verticillium dahliae VdLs.17*	0	30%	91.71%	XP_009654377.1
Bifunctional cytochrome reductase like protein	*Verticillium longisporum*	0	30%	92.00%	KAG7108725.1
hypothetical protein VDGE_07177	*Verticillium dahliae*	0	30%	91.71%	RXG50553.1

*V. isaacii*_PKS-III_contig_NMXN01000012					
Gene name	Species of blast hit	E value	% Coverage	% identity	Accession number
putative chalcone and stilbene synthase domain-containing protein	*Colletotrichum sublineola*	0	100%	75.73%	KDN65911.1
chalcone and stilbene synthase domain-containing protein	*Colletotrichum tofieldiae*	0	100%	76.13%	GKT65818.1
chalcone and stilbene synthase domain-containing protein	*Plectosphaerella cucumerina*	0.00E+00	100%	77.35%	KAH7375301.1
chalcone synthase	*Colletotrichum spaethianum*	0	100%	75.90%	GJC81220.1
chalcone and stilbene synthase domain-containing protein	*Colletotrichum tofieldiae*	0.00E+00	100%	75.90%	KZL74511.1
chalcone and stilbene synthase domain-containing protein	*Colletotrichum incanum*	0	100%	75.90%	KZL80557.1
chalcone and stilbene synthase domain-containing protein	*Colletotrichum truncatum*	0	100%	75.73%	XP_036586176.1
chalcone synthase	*Colletotrichum liriopes*	0.00E+00	100%	75.45%	GKT49639.1
chalcone and stilbene synthase domain-containing protein	*Colletotrichum graminicola M1.001*	0	100%	75.28%	XP_008099452.1
putative chalcone and stilbene synthase domain-containing protein	*Colletotrichum incanum*	0	100%	75.68%	OHW96035.1

*V._isaacii_* VRPKS-I-14_contig_NMXN01000003					

Gene name	Species of blast hit	E value	% Coverage	% identity	Accession number
hypothetical protein TruAng_007522	*Truncatella angustata*	0	100%	56.21%	KAH8198320.1
putative polyketide synthase	*Truncatella angustata*	0	99%	56.75%	XP_045957259.1
hypothetical protein AJ80_00462	*Polytolypa hystricis UAMH7299*	0	100%	54.41%	PGH27912.1
Nonribosomal peptide synthetase-like protein	*Acremonium chrysogenum ATCC 11550*	0	99%	53.29%	KFH40504.1
hypothetical protein PDE_03926	*Penicillium oxalicum 114-2*	0	100%	46.86%	EPS28980.1
hypothetical protein EYB26_003290	*Talaromyces marneffei*	0.00E+00	99%	44.93%	QGA15630.1
hypothetical protein EYB25_008228	*Talaromyces marneffei*	0	99%	44.90%	KAE8549704.1
polyketide synthase, putative	*Talaromyces marneffei ATCC 18224*	0	99%	44.90%	XP_002151741.1
Highly reducing polyketide synthase AFT9-1	*Colletotrichum orbiculare MAFF 240422*	0	99%	44.65%	TDZ19628.1
Highly reducing polyketide synthase AFT9-1	*Colletotrichum siamense*	0.00E+00	99%	44.51%	KAF4876708.1

*V. isaacii*_ VRPKS-I-25_contig_NMXN01000002					
Gene name	Species of blast hit	E value	% Coverage	% identity	Accession number
hypothetical protein S40293_03578	*Stachybotrys chartarum IBT 40293*	0	99%	89.64%	KFA46917.1
hypothetical protein S40288_08039	*Stachybotrys chartarum IBT 40288*	0	99%	87.69%	KFA72420.1
hypothetical protein S7711_03851	*Stachybotrys chartarum IBT 7711*	0.00E+00	86%	89.19%	KEY68922.1
hypothetical protein DL764_002272	*Monosporascus ibericus*	0.00E+00	99%	56.76%	RYP07817.1
hypothetical protein DL770_006186	*Monosporascus sp. CRB-9-2*	0	99%	57.05%	RYP80513.1
hypothetical protein B7494_g6164	*Chlorociboria aeruginascens*	0.00E+00	99%	56.54%	TAQ85507.1
type I iterative PKS	*Aspergillus luchuensis*	0.00E+00	99%	55.80%	XP_041547082.1
polyketide synthase	*Aspergillus costaricaensis CBS 115574*	0.00E+00	99%	55.58%	XP_025542753.1
polyketide synthase	*Aspergillus niger*	0	99%	55.38%	GAQ46573.1
polyketide synthase	*Aspergillus neoniger CBS 115656*	0	99%	55.58%	XP_025484624.1

*V. isaacii*_ VRPKS-I-2_contig_NMXN01000008					
Gene name	Species of blast hit	E value	% Coverage	% identity	Accession number
Fusaridione A synthetase fsdS	*Colletotrichum fructicola*	0	100%	85.82%	KAF4886526.1
Fusaridione A synthetase	*Colletotrichum fructicola*	0.00E+00	100%	85.79%	XP_031875985.1
hypothetical protein ANO14919_082590	*fungal sp. No.14919*	0.00E+00	99%	56.00%	GAW18777.1
putative polyketide synthase	*Xylaria grammica*	0	99%	55.52%	KAI0412784.1
hypothetical protein EKO27_g3160	*Xylaria grammica*	0	99%	55.48%	RWA11947.1

hypothetical protein UA08_04451	*Talaromyces atroroseus*	0	99%	53.75%	XP_020120442.1
ChaA	*Chaetomium olivaceum*	0	99%	52.99%	QJX57338.1
hypothetical protein LOZ25_002353	*Ophidiomyces ophidiicola*	0	99%	48.62%	KAI2161307.1
hypothetical protein LOZ59_004326	*Ophidiomyces ophidiicola*	0.00E+00	99%	48.62%	KAI1956291.1
hypothetical protein LOZ61_000210	*Ophidiomyces ophidiicola*	0	99%	48.60%	KAI1918094.1

*V. isaacii*_ VRPKS-I-12_contig_NMXN01000005					
Gene name	Species of blast hit	E value	% Coverage	% identity	Accession number
polyketide synthase	*Sodiomyces alkalinus F11*	0.00E+00	97%	75.30%	XP_028469272.1
beta-ketoacyl synthase domain-containing protein	*Colletotrichum salicis*	0	97%	72.61%	KXH33485.1
beta-ketoacyl synthase domain-containing protein	*Colletotrichum fioriniae PJ7*	0	97%	72.57%	EXF80071.1
beta-ketoacyl synthase domain-containing protein	*Colletotrichum scovillei*	0	97%	72.44%	XP_035338903.1
beta-ketoacyl synthase domain-containing protein	*Colletotrichum nymphaeae SA-01*	0	97%	72.48%	KXH30697.1
beta-ketoacyl synthase domain-containing protein	*Colletotrichum simmondsii*	0	97%	72.39%	KXH41720.1
beta-ketoacyl synthase domain-containing protein	*Colletotrichum orchidophilum*	0	97%	72.34%	XP_022470047.1
hypothetical protein G7Z17_g6123	*Cylindrodendrum hubeiense*	0	97%	65.73%	KAF7549858.1
polyketide synthase	*Pochonia chlamydosporia 170*	0	97%	65.84%	XP_018147207.1
polyketide synthase	*Thelonectria olida*	0	97%	66.00%	KAH6899708.1

*V. isaacii*_ VNRPKS-I-1_contig_NMXN01000005					
hypothetical protein CGLO_05047	*Colletotrichum gloeosporioides Cg-14*	0	100%	78.78%	EQB55056.1
hypothetical protein GQ607_010262	*Colletotrichum asianum*	0	100%	78.69%	KAF0322599.1
hypothetical protein K456DRAFT_1724748	*Colletotrichum gloeosporioides 23*	0	100%	78.73%	KAH9233890.1
Conidial yellow pigment biosynthesis polyketide synthase	*Colletotrichum fructicola*	0	100%	78.73%	KAF4937522.1
Conidial yellow pigment biosynthesis polyketide synthase	*Colletotrichum viniferum*	0	100%	78.69%	KAF4919737.1
Conidial yellow pigment biosynthesis polyketide synthase	*Colletotrichum gloeosporioides*	0	100%	78.73%	XP_045268234.1
Conidial yellow pigment biosynthesis polyketide synthase	*Colletotrichum siamense*	0	100%	78.69%	KAF4840476.1
Conidial yellow pigment biosynthesis polyketide synthase	*Colletotrichum fructicola*	0	100%	78.69%	XP_031880204.1
hypothetical protein CcaCcLH18_10320	*Colletotrichum camelliae*	0	100%	78.78%	KAH0426510.1
uncharacterized protein CkaCkLH20_00019	*Colletotrichum karsti*	0	100%	78.82%	XP_038751444.1

*V. isaacii_* VRPKS-I-6_contig_NMXN01000008					
Gene name	Species of blast hit	E value	% Coverage	% identity	Accession number
putative hybrid pks-nrps	*Xylariomycetidae sp. FL2044*	0	99%	57.65%	KAH9905503.1
putative hybrid pks-nrps	*Aspergillus burnettii*	0	98%	68.18%	KAF5861957.1
putative hybrid pks-nrps	*Aspergillus avenaceus*	0	98%	68.51%	KAE8150730.1
putative hybrid pks-nrps	*Aspergillus alliaceus*	0	98%	67.99%	KAE8392342.1
putative hybrid pks-nrps	*Aspergillus alliaceus*	0	98%	67.88%	XP_031905223.1
putative hybrid pks-nrps	*Xylariaceae sp. FL0016*	0	98%	66.98%	KAI1344561.1
putative hybrid pks-nrps	*Didymosphaeria enalia*	0	90%	66.27%	KAF2268091.1
putative hybrid pks-nrps	*Clohesyomyces aquaticus*	0	90%	65.87%	ORY13581.1
putative hybrid pks-nrps	*Biscogniauxia sp. FL1348*	0	97%	66.25%	KAI0596210.1
putative hybrid pks-nrps	*Biscogniauxia marginata*	0	97%	66.43%	KAI1495559.1

*V. isaacii_* VRPKS-I-5_contig_NMXN01000006					
Gene name	Species of blast hit	E value	% Coverage	% identity	Accession number
hypothetical protein F4775DRAFT_593279	*Biscogniauxia sp. FL1348*	0	99%	65.20%	KAI0597589.1
hypothetical protein F5X96DRAFT_674085	*Biscogniauxia mediterranea*	0	99%	64.90%	KAI1485991.1
hypothetical protein F4809DRAFT_640890	*Biscogniauxia mediterranea*	0	99%	65.07%	KAI1637132.1
lovastatin nonaketide synthase protein	*Rutstroemia sp. NJR-2017a BBW*	0	100%	43.99%	PQE06091.1
amino acid adenylation domain-containing protein	*Hymenoscyphus varicosporioides*	0	99%	41.55%	KAH8646967.1
hypothetical protein V502_02428	*Pseudogymnoascus sp. VKM F-4520 (FW-2644)*	0	99%	41.76%	KFZ23096.1
hypothetical protein V501_03696	*Pseudogymnoascus sp. VKM F-4519 (FW-2642)*	0	99%	41.60%	KFZ13457.1
uncharacterized protein BP5553_05145	*Venustampulla echinocandica*	0	99%	40.02%	XP_031870368.1
Fusaridione A synthetase fsdS	*Colletotrichum fructicola*	0	99%	40.28%	KAF4886526.1
Fusaridione A synthetase	*Colletotrichum fructicola*	0	99%	40.26%	XP_031875985.1

*V. isaacii_* VRPKS-I-9_contig_NMXN01000009					
Gene name	Species of blast hit	E value	% Coverage	% identity	Accession number
AMP-binding enzyme domain-containing protein	*Hirsutella rhossiliensis*	0	99%	72.13%	XP_044717051.1
lovastatin nonaketide synthase	*Verticillium alfalfae VaMs.102*	0	100%	69.79%	XP_003005392.1

hypothetical protein HIM_06709	*Hirsutella minnesotensis 3608*	0	99%	65.59%	KJZ73816.1
lovastatin nonaketide synthase	*Dactylonectria macrodidyma*	0	99%	57.88%	KAH7176829.1
lovastatin nonaketide synthase	*Aspergillus novofumigatus IBT 16806*	0	100%	54.52%	XP_024677790.1
lovastatin nonaketide synthase	*Hypoxylon trugodes*	0	99%	52.48%	KAI1387791.1
hypothetical protein DL768_008516	*Monosporascus sp. mg162*	0	99%	51.27%	RYP45085.1
hypothetical protein JX265_007707	*Wardomyces moseri*	0	99%	50.61%	KAI1866406.1
hypothetical protein JN550_003285	*Wardomyces moseri*	0	99%	50.51%	KAI1873032.1

*V. klebahnii_* VRPKS-I-16_contig_NMXL01000003					
Gene name	Species of blast hit	E value	% Coverage	% identity	Accession number
putative polyketide synthase [Plectosphaerella cucumerina]	*Plectosphaerella cucumerina*	0.00E+00	99%	72.37%	KAH7369281.1
putative polyketide synthase [Plectosphaerella plurivora]	*Plectosphaerella plurivora*	0	99%	71.57%	KAH6673997.1
hypothetical protein K402DRAFT_461387 [Aulographum hederae CBS 113979]	*Aulographum hederae CBS 113979*	0.00E+00	99%	49.98%	KAF1988946.1
hypothetical protein L207DRAFT_422368 [Hyaloscypha variabilis F]	*Hyaloscypha variabilis F*	0	99%	48.54%	PMD43053.1
hypothetical protein DL98DRAFT_477463 [Cadophora sp. DSE1049]	*Cadophora sp. DSE1049*	0	99%	48.17%	PVH88801.1
uncharacterized protein K444DRAFT_592521 [Hyaloscypha bicolor E]	*Hyaloscypha bicolor E*	0.00E+00	99%	48.10%	XP_024734639.1
hypothetical protein IFR05_000103 [Cadophora sp. M221]	*Cadophora sp. M221*	0.00E+00	99%	47.56%	KAG4444512.1
putative polyketide synthase [Tothia fuscella]	*Tothia fuscella*	0.00E+00	99%	47.56%	KAF2419578.1
hypothetical protein NA56DRAFT_673334 [Hyaloscypha hepaticicola]	*Hyaloscypha hepaticicola*	0.00E+00	99%	45.95%	PMD16306.1
hypothetical protein CNMCM7691_001823 [Aspergillus felis]	*Aspergillus felis*	0	99%	46.49%	KAF7182343.1

*V. klebahnii_* VRPKS-I-17_contig_NMXL01000003					
Gene name	Species of blast hit	E value	% Coverage	% identity	Accession number
Lovastatin diketide synthase LovF 16 [Colletotrichum chlorophyti]	*Colletotrichum chlorophyti*	0	99%	71.80%	OLN86478.1
Reducing polyketide synthase PKS2 [Colletotrichum trifolii]	*Colletotrichum trifolii*	0	99%	71.45%	TDZ28230.1
polyketide synthase [Colletotrichum truncatum]	*Colletotrichum truncatum*	0	99%	71.24%	XP_036585096.1
Reducing polyketide synthase PKS2 [Colletotrichum orbiculare MAFF 240422]	*Colletotrichum orbiculare MAFF 240422*	0	99%	71.41%	TDZ25780.1
Reducing polyketide synthase PKS2 [Colletotrichum sidae]	*Colletotrichum sidae*	0	99%	71.37%	TEA18683.1
Reducing polyketide synthase PKS2 [Colletotrichum spinosum]	*Colletotrichum spinosum*	0	99%	71.32%	TDZ31026.1
polyketide synthase [Colletotrichum sojae]	*Colletotrichum sojae*	0	99%	71.31%	KAF6806435.1

fatty acid synthase S-acetyltransferase [Verticillium alfalfae VaMs.102]	*Verticillium alfalfae VaMs.102*	0	99%	82.59%	XP_003008481.1
polyketide synthase [Colletotrichum musicola]	*Colletotrichum musicola*	0	99%	71.52%	KAF6830768.1
beta-ketoacyl synthase domain-containing protein [Colletotrichum orchidophilum]	*Colletotrichum orchidophilum*	0	99%	71.29%	XP_022471279.1

*V. klebahnii_* VRPKS-I-23_contig_NMXL01000003					
Gene name	Species of blast hit	E value	% Coverage	% identity	Accession number
phenolpthiocerol synthesis polyketide synthase ppsA [Sodiomyces alkalinus F11]	*Sodiomyces alkalinus F11*	0	99%	51.87%	XP_028462492.1
polyketide synthase [Verticillium nonalfalfae]	*Verticillium nonalfalfae*	0	96%	65.04%	XP_028490525.1
phenolpthiocerol synthesis polyketide synthase ppsA [Plectosphaerella plurivora]	*Plectosphaerella plurivora*	0	99%	46.01%	KAH6697431.1
phenolpthiocerol synthesis polyketide synthase ppsA [Plectosphaerella cucumerina]	*Plectosphaerella cucumerina*	0	99%	45.32%	KAH7358949.1
beta-ketoacyl synthase domain-containing protein [Colletotrichum incanum]	*Colletotrichum incanum*	0	99%	45.07%	OHW97630.1
polyketide synthase (beta-ketoacyl synthase domain-containing protein) [Colletotrichum tofieldiae]	*Colletotrichum tofieldiae*	0	97%	45.47%	KZL67886.1
Lovastatin diketide synthase LovF 13 [Colletotrichum chlorophyti]	*Colletotrichum chlorophyti*	0	98%	44.69%	OLN85715.1
beta-ketoacyl synthase domain-containing protein [Colletotrichum karsti]	*Colletotrichum karsti*	0	97%	44.88%	XP_038741012.1
beta-ketoacyl synthase domain-containing protein [Colletotrichum incanum]	*Colletotrichum incanum*	0	97%	45.09%	KZL83622.1
Reducing polyketide synthase FUB1 [Colletotrichum gloeosporioides]	*Colletotrichum gloeosporioides*	0	97%	45.40%	XP_045256339.1

*V. klebahnii_* VNRPKS-I-2_contig_NMXL01000003					
Gene name	Species of blast hit	E value	% Coverage	% identity	Accession number
hypothetical protein DL764_006210 [Monosporascus ibericus]	*Monosporascus ibericus*	0	76%	80.96%	RYP01440.1
uncharacterized protein BGZ61DRAFT_593032 [Ilyonectria robusta]	*Ilyonectria robusta*	0	76%	76.15%	XP_046097594.1
hypothetical protein BKA56DRAFT_558919 [Ilyonectria sp. MPI-CAGE-AT-0026]	*Ilyonectria sp. MPI-CAGE-AT-0026*	0	76%	75.78%	KAH6970569.1
PKS16 protein [Ilyonectria destructans]	*Ilyonectria destructans*	0	76%	74.92%	KAH7000898.1
PKS16 protein [Dactylonectria estremocensis]	*Dactylonectria estremocensis*	0	76%	74.96%	KAH7155810.1
PKS16 protein [Dactylonectria macrodidyma]	*Dactylonectria macrodidyma*	0	76%	74.64%	KAH7120608.1

uncharacterized protein INS49_005730 [Diaporthe citri]	*Diaporthe citri*	0	76%	59.21%	XP_043027652.1
PKS16 protein [Corynespora cassiicola Philippines]	*Corynespora cassiicola Philippines*	0	76%	58.60%	PSN68069.1
PKS16 protein [Rhexocercosporidium sp. MPI-PUGE-AT-0058]	*Rhexocercosporidium sp. MPI-PUGE-AT-0058*	0	76%	53.44%	KAH7357101.1
polyketide synthase [Leptodontidium sp. 2 PMI_412]	Leptodontidium sp. 2 PMI_412	0	76%	53.71%	KAH9225058.1

*V. klebahnii*_ VRPKS-I-25_contig_NMXL01000017					
Gene name	Species of blast hit	E value	% Coverage	% identity	Accession number
hypothetical protein S40293_03578 [Stachybotrys chartarum IBT 40293]	*Stachybotrys chartarum IBT 40293*	0	99%	89.34%	KFA46917.1
hypothetical protein S40288_08039 [Stachybotrys chartarum IBT 40288]	*Stachybotrys chartarum IBT 40288*	0	99%	87.39%	KFA72420.1
hypothetical protein S7711_03851 [Stachybotrys chartarum IBT 7711]	*Stachybotrys chartarum IBT 7711*	0	86%	88.90%	KEY68922.1
hypothetical protein DL764_002272 [Monosporascus ibericus]	*Monosporascus ibericus*	0	99%	56.76%	RYP07817.1
hypothetical protein DL770_006186 [Monosporascus sp. CRB-9-2]	*Monosporascus sp. CRB-9-2*	0	99%	57.05%	RYP80513.1
hypothetical protein B7494_g6164 [Chlorociboria aeruginascens]	*Chlorociboria aeruginascens*	0	99%	56.23%	TAQ85507.1
type I iterative PKS [Aspergillus luchuensis]	*Aspergillus luchuensis*	0	99%	55.84%	XP_041547082.1
polyketide synthase [Aspergillus niger]	*Aspergillus niger*	0	99%	55.71%	GAQ46573.1
polyketide synthase [Aspergillus costaricaensis CBS 115574]	*Aspergillus costaricaensis CBS 115574*	0	99%	55.41%	XP_025542753.1
polyketide synthase [Aspergillus neoniger CBS 115656]	*Aspergillus neoniger CBS 115656*	0	99%	55.50%	XP_025484624.1

*V. klebahnii*_ VRPKS-I-13_contig_NMXL01000002					
Gene name	Species of blast hit	E value	% Coverage	% identity	Accession number
polyketide synthase [Fusarium avenaceum]	*Fusarium avenaceum*	0	99%	53.50%	KIL85244.1
hypothetical protein BKA59DRAFT_387557 [Fusarium tricinctum]	*Fusarium tricinctum*	0	99%	53.17%	KAH7261591.1
hypothetical protein DER45DRAFT_513901 [Fusarium avenaceum]	*Fusarium avenaceum*	0	99%	53.17%	KAH6968136.1
polyketide synthase [Fusarium beomiforme]	*Fusarium beomiforme*	0	99%	52.95%	KAF4341752.1
putative polyketide synthase [Fusarium aywerte]	*Fusarium aywerte*	0	99%	52.99%	ALQ32765.1
unnamed protein product [Fusarium graminearum]	*Fusarium graminearum*	0	99%	52.81%	VTO93732.1
unnamed protein product [Fusarium graminearum]	*Fusarium graminearum*	0	99%	52.72%	CAF3461387.1

unnamed protein product [Fusarium graminearum]	*Fusarium graminearum*	0	99%	52.75%	CZS84488.1
unnamed protein product [Fusarium graminearum]	*Fusarium graminearum*	0	99%	52.72%	CAG2002135.1
hypothetical protein FGRMN_6861 [Fusarium graminum]	*Fusarium graminum*	0	99%	52.74%	KAF4992917.1

*V. klebahnii*_ VRPKS-I-19_contig_NMXL01000006					
Gene name	Species of blast hit	E value	% Coverage	% identity	Accession number
hypothetical protein J1614_005792 [Leptosphaeria biglobosa]	*Leptosphaeria biglobosa*	0	99%	77.25%	KAH9873394.1
Type I Polyketide synthase [Verticillium nonalfalfae]	*Verticillium nonalfalfae*	0	100%	76.49%	XP_028494609.1
Reducing polyketide synthase PKS2 like protein [Verticillium longisporum]	*Verticillium longisporum*	0	100%	76.54%	KAG7143635.1
hypothetical protein VD0004_g7884 [Verticillium dahliae]	*Verticillium dahliae*	0	100%	76.40%	PNH38983.1
fatty acid synthase S-acetyltransferase [Verticillium dahliae]	*Verticillium dahliae*	0	100%	76.49%	KAH6703869.1
fatty acid synthase S-acetyltransferase [Verticillium dahliae VdLs.17]	*Verticillium dahliae VdLs.17*	0	100%	75.85%	XP_009655626.1
hypothetical protein BN1708_003179 [Verticillium longisporum]	*Verticillium longisporum*	0	100%	75.91%	CRK18834.1
Inositol 2-dehydrogenase 2 [Verticillium dahliae VDG2]	*Verticillium dahliae VDG2*	0	96%	76.07%	KAF3346614.1
hypothetical protein BN1708_009954 [Verticillium longisporum]	*Verticillium longisporum*	0	88%	75.70%	CRK10893.1
hypothetical protein VDGD_03466 [Verticillium dahliae]	*Verticillium dahliae*	0	87%	75.87%	RBQ87991.1

*V. klebahnii*_ VRPKS-I-20_contig_NMXL01000001					
Gene name	Species of blast hit	E value	% Coverage	% identity	Accession number
hypothetical protein PENARI_c014G02915 [Penicillium arizonense]	*Penicillium arizonense*	0	99%	67.20%	XP_022486633.1
hypothetical protein G6O67_006196 [Ophiocordyceps sinensis]	*Ophiocordyceps sinensis*	0	99%	51.01%	KAF4506076.1
Lovastatin nonaketide synthase-like protein [Acremonium chrysogenum ATCC 11550]	*Acremonium chrysogenum ATCC 11550*	0	99%	50.30%	KFH43961.1
uncharacterized protein BGW36DRAFT_428643 [Talaromyces proteolyticus]	*Talaromyces proteolyticus*	0	99%	50.31%	XP_046071584.1
type I iterative polyketide synthase [Purpureocillium lilacinum]	*Purpureocillium lilacinum*	0	99%	49.54%	GJN77380.1
putative polyketide synthase protein [Purpureocillium lilacinum]	*Purpureocillium lilacinum*	0	99%	49.48%	PWI74782.1
type I Iterative Polyketide synthase (PKS) [Purpureocillium lilacinum]	*Purpureocillium lilacinum*	0	99%	49.42%	GJN68941.1
uncharacterized protein BDV37DRAFT_285272 [Aspergillus pseudonomiae]	*Aspergillus pseudonomiae*	0	99%	49.35%	XP_031939187.1
hypothetical protein BDV32DRAFT_145394 [Aspergillus pseudonomiae]	*Aspergillus pseudonomiae*	0	99%	49.35%	KAB8264716.1
uncharacterized protein ANOM_000018 [Aspergillus nomiae NRRL 13137]	*Aspergillus nomiae NRRL 13137*	0	99%	48.95%	XP_015412436.1

*V. klebahnii*_PKS-III_contig_NMXL01000010					
Gene name	Species of blast hit	E value	% Coverage	% identity	Accession number
putative chalcone and stilbene synthase domain-containing protein [Colletotrichum sublineola]	*Colletotrichum sublineola*	0	100%	75.28%	KDN65911.1
chalcone and stilbene synthase domain-containing protein [Colletotrichum tofieldiae]	*Colletotrichum tofieldiae*	0	100%	75.68%	GKT65818.1
chalcone synthase [Colletotrichum spaethianum]	*Colletotrichum spaethianum*	0	100%	75.45%	GJC81220.1
chalcone and stilbene synthase domain-containing protein [Colletotrichum tofieldiae]	*Colletotrichum tofieldiae*	0	100%	75.45%	KZL74511.1
chalcone and stilbene synthase domain-containing protein [Plectosphaerella cucumerina]	*Plectosphaerella cucumerina*	0	100%	76.46%	KAH7375301.1
chalcone and stilbene synthase domain-containing protein [Colletotrichum incanum]	*Colletotrichum incanum*	0	100%	75.45%	KZL80557.1
chalcone and stilbene synthase domain-containing protein [Colletotrichum truncatum]	*Colletotrichum truncatum*	0	100%	75.28%	XP_036586176.1
putative chalcone and stilbene synthase domain-containing protein [Colletotrichum incanum]	*Colletotrichum incanum*	0	100%	75.23%	OHW96035.1
chalcone and stilbene synthase domain-containing protein [Colletotrichum graminicola M1.001]	*Colletotrichum graminicola M1.001*	0	100%	74.83%	XP_008099452.1
chalcone synthase [Colletotrichum liriopes]	*Colletotrichum liriopes*	0	100%	75.00%	GKT49639.1

*V. klebahnii*_ VRPKS-I-26_contig_NMXL01000010					
Gene name	Species of blast hit	E value	% Coverage	% identity	Accession number
hypothetical protein DL768_003915 [Monosporascus sp. mg162]	*Monosporascus sp. mg162*	0	99%	50.81%	RYP50633.1
Ketol-acid reductoisomerase [Verticillium dahliae VDG2]	*Verticillium dahliae VDG2*	0	85%	67.54%	KAF3349326.1
RecName: Full=Highly reducing polyketide synthase apmlA; Short=HRPKS apmlA; AltName: Full=Phaeospelide A biosynthesis cluster protein apmlA [Apiospora sphaerosperma]	*Apiospora sphaerosperma*	0	99%	49.55%	P0CU84.1
mycocerosic acid synthase [Verticillium dahliae VdLs.17]	*Verticillium dahliae VdLs.17*	0	99%	61.74%	XP_009654470.1
D-amino-acid oxidase [Verticillium dahliae VDG1]	*Verticillium dahliae VDG1*	0	81%	69.50%	KAF3356735.1
putative polyketide synthase [Xylariales sp. PMI_506]	*Xylariales sp. PMI_506*	0	98%	42.07%	KAH8657763.1
Fumagillin dodecapentaenoate synthase [Lachnellula willkommii]	*Lachnellula willkommii*	0	99%	42.26%	TVY89575.1
Fumagillin dodecapentaenoate synthase [Lachnellula arida]	*Lachnellula arida*	0	99%	42.20%	TVY19100.1
reducing type I polyketide synthase [Xylariomycetidae sp. FL2044]	*Xylariomycetidae sp. FL2044*	0	99%	42.24%	KAH9902283.1
Fumagillin dodecapentaenoate synthase [Lachnellula cervina]	*Lachnellula cervina*	0	98%	42.24%	TVY51056.1

*V. klebahnii*_ VRPKS-I-14_contig_NMXL01000010					
Gene name	Species of blast hit	E value	% Coverage	% identity	Accession number
hypothetical protein TruAng_007522 [Truncatella angustata]	*Truncatella angustata*	0	100%	57.03%	KAH8198320.1
putative polyketide synthase [Truncatella angustata]	*Truncatella angustata*	0	98%	56.71%	XP_045957259.1
Nonribosomal peptide synthetase-like protein [Acremonium chrysogenum ATCC 11550]	*Acremonium chrysogenum ATCC 11550*	0	98%	54.02%	KFH40504.1
hypothetical protein AJ80_00462 [Polytolypa hystricis UAMH7299]	*Polytolypa hystricis UAMH7299*	0	100%	54.32%	PGH27912.1
hypothetical protein EYB26_003290 [Talaromyces marneffei]	*Talaromyces marneffei*	0	99%	45.34%	QGA15630.1
polyketide synthase, putative [Talaromyces marneffei ATCC 18224]	*Talaromyces marneffei ATCC 18224*	0	99%	45.60%	XP_002151741.1
hypothetical protein EYB25_008228 [Talaromyces marneffei]	*Talaromyces marneffei*	0	99%	45.31%	KAE8549704.1
Highly reducing polyketide synthase AFT9-1 [Colletotrichum orbiculare MAFF 240422]	*Colletotrichum orbiculare MAFF 240422*	0	99%	45.39%	TDZ19628.1
Highly reducing polyketide synthase AFT9-1 [Colletotrichum siamense]	*Colletotrichum siamense*	0	99%	45.13%	KAF4808378.1
hypothetical protein K456DRAFT_1842648 [Colletotrichum gloeosporioides 23]	*Colletotrichum gloeosporioides 23*	0	99%	45.18%	KAH9230444.1

*V. klebahnii*_ VRPKS-I-21_contig_NMXL01000013					
Gene name	Species of blast hit	E value	% Coverage	% identity	Accession number
polyketide synthase [Colletotrichum plurivorum]	*Colletotrichum plurivorum*	0	92%	69.20%	KAF6826616.1
beta-ketoacyl synthase domain-containing protein [Colletotrichum fioriniae PJ7]	*Colletotrichum fioriniae PJ7*	0	92%	68.59%	EXF80608.1
Lovastatin diketide synthase LovF 2 [Colletotrichum chlorophyti]	*Colletotrichum chlorophyti*	0	92%	69.39%	OLN91972.1
polyketide synthase [Colletotrichum musicola]	*Colletotrichum musicola*	0	92%	68.98%	KAF6845034.1
beta-ketoacyl synthase domain-containing protein [Colletotrichum scovillei]	*Colletotrichum scovillei*	0	92%	69.14%	KAG7053867.1
polyketide synthase [Colletotrichum sojae]	*Colletotrichum sojae*	0	92%	69.07%	KAF6809610.1
polyketide synthase (beta-ketoacyl synthase domain-containing protein) [Colletotrichum tofieldiae]	*Colletotrichum tofieldiae*	0	92%	68.45%	KZL70949.1
uncharacterized protein CkaCkLH20_04708 [Colletotrichum karsti]	*Colletotrichum karsti*	0	92%	68.39%	XP_038747034.1
beta-ketoacyl synthase domain-containing protein [Colletotrichum scovillei]	*Colletotrichum scovillei*	0	92%	68.54%	XP_035330138.1
beta-ketoacyl synthase domain-containing protein [Colletotrichum simmondsii]	*Colletotrichum simmondsii*	0	92%	68.78%	KXH36687.1

*V. klebahnii_* VRPKS-I-12_contig_NMXL01000003					
Gene name	Species of blast hit	E value	% Coverage	% identity	Accession number
polyketide synthase [Sodiomyces alkalinus F11]	*Sodiomyces alkalinus F11*	0	97%	75.14%	XP_028469272.1
beta-ketoacyl synthase domain-containing protein [Colletotrichum salicis]	*Colletotrichum salicis*	0	97%	72.52%	KXH33485.1
beta-ketoacyl synthase domain-containing protein [Colletotrichum fioriniae PJ7]	*Colletotrichum fioriniae PJ7*	0	97%	72.47%	EXF80071.1
beta-ketoacyl synthase domain-containing protein [Colletotrichum nymphaeae SA-01]	*Colletotrichum nymphaeae SA-01*	0	97%	72.41%	KXH30697.1
beta-ketoacyl synthase domain-containing protein [Colletotrichum scovillei]	*Colletotrichum scovillei*	0	97%	72.37%	XP_035338903.1
beta-ketoacyl synthase domain-containing protein [Colletotrichum orchidophilum]	*Colletotrichum orchidophilum*	0	97%	72.19%	XP_022470047.1
beta-ketoacyl synthase domain-containing protein [Colletotrichum simmondsii]	*Colletotrichum simmondsii*	0	97%	72.27%	KXH41720.1
hypothetical protein G7Z17_g6123 [Cylindrodendrum hubeiense]	*Cylindrodendrum hubeiense*	0	97%	65.71%	KAF7549858.1
polyketide synthase [Pochonia chlamydosporia 170]	*Pochonia chlamydosporia 170*	0	97%	65.77%	XP_018147207.1
polyketide synthase [Thelonectria olida]	*Thelonectria olida*	0	97%	65.88%	KAH6899708.1

*V. klebahnii_* VNRPKS-I-16_contig_NMXL01000003					
Gene name	Species of blast hit	E value	% Coverage	% identity	Accession number
uncharacterized protein CkaCkLH20_00019 [Colletotrichum karsti]	*Colletotrichum karsti*	0	100%	79.23%	XP_038751444.1
hypothetical protein CGLO_05047 [Colletotrichum gloeosporioides Cg-14]	*Colletotrichum gloeosporioides Cg-14*	0	100%	78.96%	EQB55056.1
hypothetical protein CcaCcLH18_10320 [Colletotrichum camelliae]	*Colletotrichum camelliae*	0	100%	79.05%	KAH0426510.1
hypothetical protein K456DRAFT_1724748 [Colletotrichum gloeosporioides 23]	*Colletotrichum gloeosporioides 23*	0	100%	78.91%	KAH9233890.1
Conidial yellow pigment biosynthesis polyketide synthase [Colletotrichum fructicola]	*Colletotrichum fructicola*	0	100%	78.91%	KAF4937522.1
hypothetical protein GQ607_010262 [Colletotrichum asianum]	*Colletotrichum asianum*	0	100%	78.91%	KAF0322599.1
Conidial yellow pigment biosynthesis polyketide synthase [Colletotrichum gloeosporioides]	*Colletotrichum gloeosporioides*	0	100%	78.91%	XP_045268234.1
Conidial yellow pigment biosynthesis polyketide synthase [Colletotrichum viniferum]	*Colletotrichum viniferum*	0	100%	78.87%	KAF4919737.1
Conidial yellow pigment biosynthesis polyketide synthase [Colletotrichum tropicale]	*Colletotrichum tropicale*	0	100%	78.87%	KAF4827098.1
Conidial yellow pigment biosynthesis polyketide synthase [Colletotrichum siamense]	*Colletotrichum siamense*	0	100%	78.87%	KAF4840476.1

*V. klebahnii_* VRPKS-I-9_contig_NMXL01000011					
Gene name	Species of blast hit	E value	% Coverage	% identity	Accession number
AMP-binding enzyme domain-containing protein [Hirsutella rhossiliensis]	*Hirsutella rhossiliensis*	0	99%	72.49%	XP_044717051.1
lovastatin nonaketide synthase [Verticillium alfalfae VaMs.102]	*Verticillium alfalfae VaMs.102*	0	100%	70.03%	XP_003005392.1
hypothetical protein HIM_06709 [Hirsutella minnesotensis 3608]	*Hirsutella minnesotensis 3608*	0	99%	66.00%	KJZ73816.1
lovastatin nonaketide synthase [Dactylonectria macrodidyma]	*Dactylonectria macrodidyma*	0	99%	57.83%	KAH7176829.1
lovastatin nonaketide synthase [Aspergillus novofumigatus IBT 16806]	*Aspergillus novofumigatus IBT 16806*	0	100%	54.74%	XP_024677790.1
lovastatin nonaketide synthase [Hypoxylon trugodes]	*Hypoxylon trugodes*	0	99%	52.49%	KAI1387791.1
hypothetical protein DL768_008516 [Monosporascus sp. mg162]	*Monosporascus sp. mg162*	0	99%	50.94%	RYP45085.1
hypothetical protein JX265_007707 [Wardomyces moseri]	*Wardomyces moseri*	0	99%	50.58%	KAI1866406.1
hypothetical protein JN550_003285 [Wardomyces moseri]	*Wardomyces moseri*	0	99%	50.49%	KAI1873032.1
hypothetical protein JX266_000322 [Wardomyces moseri]	*Wardomyces moseri*	0	99%	50.41%	KAI1855457.1

*V. klebahnii_* VRPKS-I-2_contig_NMXL01000020					
Gene name	Species of blast hit	E value	% Coverage	% identity	Accession number
Fusaridione A synthetase fsdS [Colletotrichum fructicola]	*Colletotrichum fructicola*	0	99%	86.73%	KAF4886526.1
Fusaridione A synthetase [Colletotrichum fructicola]	*Colletotrichum fructicola*	0	99%	86.71%	XP_031875985.1
putative polyketide synthase [Nemania sp. FL0916]	*Nemania sp. FL0916*	0	99%	56.70%	KAI1177826.1
hypothetical protein ANO14919_082590 [fungal sp. No.14919]	*fungal sp. No.14919*	0	99%	56.18%	GAW18777.1
putative polyketide synthase [Xylaria grammica]	*Xylaria grammica*	0	99%	55.70%	KAI0412784.1
hypothetical protein EKO27_g3160 [Xylaria grammica]	*Xylaria grammica*	0	99%	55.58%	RWA11947.1
ChaA [Chaetomium olivaceum]	*Chaetomium olivaceum*	0	99%	53.52%	QJX57338.1
hypothetical protein UA08_04451 [Talaromyces atroroseus]	*Talaromyces atroroseus*	0	99%	54.06%	XP_020120442.1
hypothetical protein LOZ25_002353 [Ophidiomyces ophidiicola]	*Ophidiomyces ophidiicola*	0	99%	48.60%	KAI2161307.1
hypothetical protein LOZ59_004326 [Ophidiomyces ophidiicola]	*Ophidiomyces ophidiicola*	0	99%	48.62%	KAI1956291.1

*V. klebahnii_* VRPKS-I-5_contig_NMXL01000008					
Gene name	Species of blast hit	E value	% Coverage	% identity	Accession number

polyketide synthase	*Biscogniauxia mediterranea*	0	99%	64.69%	KAI1485991.1
polyketide synthase	*Biscogniauxia sp. FL1348*	0	99%	64.81%	KAI0597589.1
polyketide synthase	*Biscogniauxia mediterranea*	0	99%	64.80%	KAI1637132.1
polyketide synthase	*Rutstroemia sp. NJR-2017a BBW*	0	100%	43.74%	PQE06091.1
polyketide synthase	*Hymenoscyphus varicosporioides*	0	99%	41.14%	KAH8646967.1
polyketide synthase	*Pseudogymnoascus sp. VKM F-4520 (FW-2644)*	0	99%	41.47%	KFZ23096.1
polyketide synthase	*Pseudogymnoascus sp. VKM F-4519 (FW-2642)*	0	99%	41.40%	KFZ13457.1
polyketide synthase	*Pseudogymnoascus sp. VKM F-4514 (FW-929)*	0	99%	41.25%	KFY37827.1
polyketide synthase	*Pseudogymnoascus sp. VKM F-3557*	0	99%	41.25%	KFX98753.1
polyketide synthase	*Chaetomium sp. MPI-CAGE-AT-0009*	0	100%	67.42%	KAH6844804.1

*V. klebahnii_* VRPKS-I-6_contig_NMXL01000002					
Gene name	Species of blast hit	E value	% Coverage	% identity	Accession number
Polyketide synthase-nonribosomal peptide synthetase	*Aspergillus burnettii*	0	99%	66.66%	KAF5861957.1
Polyketide synthase-nonribosomal peptide synthetase	*Aspergillus alliaceus*	0	99%	66.50%	KAE8392342.1
Polyketide synthase-nonribosomal peptide synthetase	*Aspergillus alliaceus*	0	99%	66.47%	XP_031905223.1
Polyketide synthase-nonribosomal peptide synthetase	*Xylariaceae sp. FL0016*	0	99%	65.95%	KAI1344561.1
Polyketide synthase-nonribosomal peptide synthetase	*Monosporascus sp. GIB2*	0	99%	61.52%	RYP15707.1
Polyketide synthase-nonribosomal peptide synthetase	*Clohesyomyces aquaticus*	0	91%	65.96%	ORY13581.1
Polyketide synthase-nonribosomal peptide synthetase	*Didymosphaeria enalia*	0	91%	66.10%	KAF2268091.1
Polyketide synthase-nonribosomal peptide synthetase	*Xylariaceae sp. FL0804*	0	99%	61.31%	KAI0484820.1
Polyketide synthase-nonribosomal peptide synthetase	*Ophiobolus disseminans*	0	91%	64.61%	KAF2820849.1
Polyketide synthase-nonribosomal peptide synthetase	*Xylariomycetidae sp. FL2044*	0	99%	58.07%	KAH9905503.1

*V. longisporum_* VRPKS-I-22_contig_CVQH01005557					
Gene name	Top blast hit	E value	% Coverage	% identity	Accession number
polyketide synthase	*Fusarium mexicanum*	0	99%	70.23%	KAF5539484.1

putative polyketide synthase	*Fusarium dlaminii*	0	99%	70.38%	ALQ32831.1
polyketide synthase	*Fusarium subglutinans*	0	99%	70.16%	XP_036534733.1
polyketide synthase	*Fusarium acutatum*	0	99%	70.49%	KAF4428951.1
hypothetical protein FSARC_14608	*Fusarium sarcochroum*	0	100%	70.28%	KAF4944666.1
polyketide synthase	*Fusarium pseudoanthophilum*	0	100%	70.30%	KAF5573817.1
putative polyketide synthase	*Fusarium miscanthi*	0	99%	70.59%	ALQ32877.1
putative polyketide synthase	*Fusarium aywerte*	0	100%	69.65%	ALQ32768.1
polyketide synthase	*Fusarium mundagurra*	0	99%	70.03%	KAF5716826.1
polyketide synthase	*Fusarium denticulatum*	0	100%	70.09%	KAF5687147.1

*V. longisporum_* VRPKS-I-23_contig_CVQH01020307					
Gene name	Top blast hit	E value	% Coverage	% identity	Accession number
phenolpthiocerol synthesis polyketide synthase ppsA	*Sodiomyces alkalinus F11*	0	98%	47.31%	XP_028462492.1
phenolpthiocerol synthesis polyketide synthase ppsA	*Plectosphaerella plurivora*	0	99%	42.51%	KAH6697431.1
phenolpthiocerol synthesis polyketide synthase ppsA	*Plectosphaerella cucumerina*	0	99%	42.47%	KAH7358949.1
hypothetical protein BJF96_g3857	*Verticillium dahliae*	0	37%	94.38%	PNH33126.1
Lovastatin diketide synthase LovF 13	*Colletotrichum chlorophyti*	0	98%	41.90%	OLN85715.1
beta-ketoacyl synthase domain-containing protein	*Colletotrichum incanum*	0	99%	41.90%	OHW97630.1
Reducing polyketide synthase FUB1	*Colletotrichum gloeosporioides*	0	97%	42.17%	XP_045256339.1
Reducing polyketide synthase FUB1	*Colletotrichum aenigma*	0	97%	42.05%	XP_037174401.1
beta-ketoacyl synthase domain-containing protein	*Colletotrichum karsti*	0	97%	42.07%	XP_038741012.1
Reducing polyketide synthase	*Colletotrichum fructicola*	0	97%	42.05%	XP_031892623.1

*V. longisporum_* VRPKS-I-17_contig_CVQH01023638					
Gene name	Top blast hit	E value	% Coverage	% identity	Accession number
Lovastatin diketide synthase LovF 16	*Colletotrichum chlorophyti*	0	97%	69.51%	OLN86478.1
polyketide synthase	*Colletotrichum truncatum*	0	97%	68.54%	XP_036585096.1
Reducing polyketide synthase PKS2	*Colletotrichum trifolii*	0	97%	68.49%	TDZ28230.1
Reducing polyketide synthase PKS2	*Colletotrichum orbiculare MAFF 240422*	0	97%	68.53%	TDZ25780.1
Reducing polyketide synthase PKS2	*Colletotrichum sidae*	0	97%	68.44%	TEA18683.1
beta-ketoacyl synthase domain-containing protein	*Colletotrichum salicis*	0	97%	68.72%	KXH66496.1

polyketide synthase	*Colletotrichum musicola*	0	97%	68.84%	KAF6830768.1
beta-ketoacyl synthase domain-containing protein	*Colletotrichum fioriniae PJ7*	0	97%	68.81%	EXF85385.1
Reducing polyketide synthase PKS2	*Colletotrichum spinosum*	0	97%	68.39%	TDZ31026.1
beta-ketoacyl synthase domain-containing protein	*Colletotrichum simmondsii*	0	97%	68.81%	KXH44475.1

*V. longisporum*_ VRPKS-I-23_contig_CVQH01020607					
Gene name	Top blast hit	E value	% Coverage	% identity	Accession number
phenolpthiocerol synthesis polyketide synthase ppsA	*Sodiomyces alkalinus F11*	0	99%	49.04%	XP_028462492.1
phenolpthiocerol synthesis polyketide synthase ppsA	*Plectosphaerella plurivora*	0	100%	42.91%	KAH6697431.1
phenolpthiocerol synthesis polyketide synthase ppsA	*Plectosphaerella cucumerina*	0	99%	42.62%	KAH7358949.1
Lovastatin diketide synthase LovF 13	*Colletotrichum chlorophyti*	0	99%	42.30%	OLN85715.1
Reducing polyketide synthase FUB1	*Colletotrichum gloeosporioides*	0	98%	42.41%	XP_045256339.1
beta-ketoacyl synthase domain-containing protein	*Colletotrichum incanum*	0	99%	42.15%	OHW97630.1
acyl transferase domain-containing protein	*Hirsutella rhossiliensis*	0	99%	41.98%	XP_044721574.1
Reducing polyketide synthase FUB1	*Colletotrichum aenigma*	0	98%	42.26%	XP_037174401.1
hypothetical protein CEP54_008569	*Fusarium sp. AF-8*	0	99%	42.43%	RSL56874.1
beta-ketoacyl synthase domain-containing protein	*Colletotrichum karsti*	0	98%	41.96%	XP_038741012.1

*V. longisporum*_ VRPKS-I-17_contig_CVQH01000225					
Gene name	Top blast hit	E value	% Coverage	% identity	Accession number
Lovastatin diketide synthase LovF 16	*Colletotrichum chlorophyti*	0	99%	69.14%	OLN86478.1
hypothetical protein VDGD_08448	*Verticillium dahliae*	0	69%	98.53%	RBQ89174.1
polyketide synthase	*Colletotrichum truncatum*	0	99%	68.60%	XP_036585096.1
Reducing polyketide synthase PKS2	*Colletotrichum trifolii*	0	99%	68.33%	TDZ28230.1
Reducing polyketide synthase PKS2	*Colletotrichum orbiculare MAFF 240422*	0	99%	68.38%	TDZ25780.1
Reducing polyketide synthase PKS2	*Colletotrichum sidae*	0	99%	68.29%	TEA18683.1
Reducing polyketide synthase PKS2	*Colletotrichum spinosum*	0	99%	68.25%	TDZ31026.1
polyketide synthase	*Colletotrichum musicola*	0	99%	68.41%	KAF6830768.1
beta-ketoacyl synthase domain-containing protein	*Colletotrichum fioriniae PJ7*	0	99%	68.56%	EXF85385.1
beta-ketoacyl synthase domain-containing protein	*Colletotrichum salicis*	0	99%	68.43%	KXH66496.1

*V. longisporum_* VRPKS-I-19_contig_CVQH01002447					
Gene name	Top blast hit	E value	% Coverage	% identity	Accession number
hypothetical protein J1614_005792	*Leptosphaeria biglobosa*	0	100%	77.60%	KAH9873394.1
hypothetical protein BN1723_002883	*Verticillium longisporum*	0	64%	96.78%	CRK22444.1
Type I Iterative PKS	*Claviceps purpurea*	0	100%	61.46%	KAG6264852.1
Type I Iterative PKS	*Claviceps purpurea*	0	100%	61.51%	KAG6168136.1
Type I Iterative PKS	*Claviceps purpurea*	0	100%	61.40%	KAG6257628.1
Type I Iterative PKS	*Claviceps purpurea*	0	100%	61.30%	KAG6313294.1
Type I Iterative PKS	*Claviceps pusilla*	0	100%	62.07%	KAG6001262.1
Type I Iterative PKS	*Claviceps purpurea*	0	100%	61.40%	KAG6172346.1
Type I Iterative PKS	*Claviceps purpurea*	0	100%	61.46%	KAG6178588.1
Type I Iterative PKS	*Claviceps purpurea*	0	100%	61.40%	KAG6319079.1

*V. longisporum_* VRPKS-I-19_contig_CVQH01000001					
Gene name	Top blast hit	E value	% Coverage	% identity	Accession number
hypothetical protein J1614_005792	*Leptosphaeria biglobosa*	0	99%	78.27%	KAH9873394.1
Reducing polyketide synthase PKS2 like protein	*Verticillium longisporum*	0	73%	93.72%	KAG7126724.1
Type I Iterative PKS	*Claviceps purpurea*	0	97%	62.51%	KAG6264852.1
Type I Iterative PKS	*Claviceps purpurea*	0	97%	62.46%	KAG6168136.1
Type I Iterative PKS	*Claviceps purpurea*	0	97%	62.46%	KAG6257628.1
Type I Iterative PKS	*Claviceps purpurea*	0	97%	62.41%	KAG6178588.1
Type I Iterative PKS	*Claviceps purpurea*	0	97%	62.41%	KAG6313294.1
Type I Iterative PKS	*Claviceps purpurea*	0	97%	62.36%	KAG6172346.1
Type I Iterative PKS	*Claviceps purpurea*	0	97%	62.31%	KAG6278039.1
Type I Iterative PKS	*Claviceps purpurea*	0	97%	62.31%	KAG6152537.1

*V. longisporum_* VRPKS-I-19_contig_CVQH01000001					
Gene name	Top blast hit	E value	% Coverage	% identity	Accession number
hypothetical protein J1614_005792	Leptosphaeria biglobosa	9.00E-17	97%	85.51%	KAH9873394.1
hypothetical protein E4U41_004656	Claviceps citrina	7.00E-10	94%	71.64%	KAG6015267.1
hypothetical protein HIM_05896	Hirsutella minnesotensis 3608	2.00E-09	92%	68.18%	KJZ74779.1
hypothetical protein E4U42_001802	Claviceps africana	3.00E-09	94%	71.64%	KAG5912807.1

hypothetical protein HIM_06262	Hirsutella minnesotensis 3608	5.00E-09	88%	66.67%	KJZ74256.1
hypothetical protein E4U54_004405	Claviceps lovelessii	1.00E-08	95%	69.12%	KAG6015049.1
acyl transferase domain-containing protein	Hirsutella rhossiliensis	1.00E-08	97%	60.87%	XP_044719201.1
Type I Iterative PKS	Claviceps pusilla	4.00E-08	95%	69.12%	KAG6001262.1
Type I Iterative PKS	Claviceps cyperi	5.00E-08	94%	70.15%	KAG5972447.1
hypothetical protein CDD83_5286	Cordyceps sp. RAO-2017	5.00E-08	94%	64.18%	PHH89775.1

*V. longisporum*_ VRPKS-I-26_contig_CVQH01000001					
Gene name	Top blast hit	E value	% Coverage	% identity	Accession number
hypothetical protein F5B20DRAFT_586947	*Whalleya microplaca*	0.00E+00	99%	63.19%	KAI1073750.1
hypothetical protein DL768_003915	*Monosporascus sp. mg162*	0.00E+00	97%	62.84%	RYP50633.1
hypothetical protein BN1708_007417	*Verticillium longisporum*	0.00E+00	76%	97.21%	CRK37551.1
RecName: Full=Highly reducing polyketide synthase apmlA; Short=HRPKS apmlA; AltName: Full=Phaeospelide A biosynthesis cluster protein apmlA	*Apiospora sphaerosperma*	0.00E+00	98%	59.98%	P0CU84.1
putative polyketide synthase	*Xylariales sp. PMI_506*	0.00E+00	97%	53.15%	KAH8657763.1
hypothetical protein BN1708_001184	*Verticillium longisporum*	0.00E+00	74%	92.92%	CRK33507.1
reducing type I polyketide synthase	*Plectosphaerella cucumerina*	0.00E+00	97%	53.41%	KAH7358079.1
Lovastatin diketide synthase protein	*Rutstroemia sp. NJR-2017a WRK4*	0.00E+00	98%	53.96%	PQE16274.1
Fumagillin dodecapentaenoate synthase	*Lachnellula willkommii*	0.00E+00	98%	53.06%	TVY89575.1
polyketide synthase 2	*Didymosphaeria enalia*	0.00E+00	97%	52.43%	KAF2259783.1

*V. longisporum*_ VNRPKS-I-1_contig_CVQH01019335					
Gene name	Top blast hit	E value	% Coverage	% identity	Accession number
uncharacterized protein CkaCkLH20_00019	*Colletotrichum karsti*	0	100%	75.58%	XP_038751444.1
hypothetical protein CGLO_05047	*Colletotrichum gloeosporioides Cg-14*	0	100%	75.23%	EQB55056.1
hypothetical protein GQ607_010262	*Colletotrichum asianum*	0	100%	75.23%	KAF0322599.1
Conidial yellow pigment biosynthesis polyketide synthase	*Colletotrichum fructicola*	0	100%	75.18%	KAF4937522.1
hypothetical protein CcaCcLH18_10320	*Colletotrichum camelliae*	0	100%	75.09%	KAH0426510.1
Conidial yellow pigment biosynthesis polyketide synthase	*Colletotrichum gloeosporioides*	0	100%	75.18%	XP_045268234.1

Conidial yellow pigment biosynthesis polyketide synthase	*Colletotrichum fructicola*	0	100%	75.14%	XP_031880204.1
Conidial yellow pigment biosynthesis polyketide synthase	*Colletotrichum siamense*	0	100%	75.14%	KAF4812766.1
Conidial yellow pigment biosynthesis polyketide synthase	*Colletotrichum tropicale*	0	100%	75.27%	KAF4827098.1
hypothetical protein K456DRAFT_1724748	*Colletotrichum gloeosporioides 23*	0	100%	75.14%	KAH9233890.1

*V. longisporum_* VNRPKS-I-1_contig_CVQH01006668					
Gene name	Top blast hit	E value	% Coverage	% identity	Accession number
polyketide synthase	*Sodiomyces alkalinus F11*	2.00E-54	67%	53.94%	XP_028469272.1
beta-ketoacyl synthase domain-containing protein	*Colletotrichum fioriniae PJ7*	9.00E-54	64%	56.60%	EXF80071.1
beta-ketoacyl synthase domain-containing protein	*Colletotrichum nymphaeae SA-01*	2.00E-53	64%	57.23%	KXH30697.1
beta-ketoacyl synthase domain-containing protein	*Colletotrichum scovillei*	2.00E-53	64%	57.23%	XP_035338903.1
beta-ketoacyl synthase domain-containing protein	*Colletotrichum salicis*	2.00E-53	64%	56.60%	KXH33485.1
beta-ketoacyl synthase domain-containing protein	*Colletotrichum orchidophilum*	1.00E-50	64%	54.38%	XP_022470047.1
beta-ketoacyl synthase domain-containing protein	*Colletotrichum simmondsii*	2.00E-49	64%	55.21%	KXH41720.1
polyketide synthase	*Thelonectria olida*	2.00E-48	64%	52.47%	KAH6899708.1
Polyketide synthase-nonribosomal peptide synthetase	*Neonectria ditissima*	2.00E-48	64%	50.62%	KPM42068.1
hypothetical protein G7Z17_g6123	*Cylindrodendrum hubeiense*	3.00E-46	63%	50.00%	KAF7549858.1

*V. longisporum_* VRPKS-I-11_contig_CVQH01006891					
Gene name	Top blast hit	E value	% Coverage	% identity	Accession number
Nonribosomal peptide synthetase-like protein	*Acremonium chrysogenum ATCC 11550*	0	80%	44.24%	KFH45364.1
hypothetical protein F5Y06DRAFT_297186	*Hypoxylon sp. FL0890*	0	81%	40.81%	KAI0837758.1
polyketide synthase	*Lasiodiplodia theobromae*	0	81%	39.90%	KAF9630276.1
Fusarin C synthetase	*Lasiodiplodia theobromae*	0	81%	39.68%	KAB2573439.1
uncharacterized protein BCR38DRAFT_413069	*Pseudomassariella vexata*	0	81%	40.05%	XP_040711562.1
uncharacterized protein K452DRAFT_243204	*Aplosporella prunicola CBS 121167*	0	81%	39.24%	XP_033401020.1
Polyketide synthase	*Lasiodiplodia theobromae*	0	81%	39.12%	XP_035374836.1
hypothetical protein Egran_04668	*Elaphomyces granulatus*	0	81%	38.90%	OXV07567.1

hypothetical protein ASPCADRAFT_5570	*Aspergillus carbonarius ITEM 5010*	0	81%	36.86%	OOF95055.1
Fusarin C synthetase like protein	*Verticillium longisporum*	0	23%	100.00%	KAG7106604.1

*V. longisporum_* VRPKS-I-8_contig_CVQH01025950					
Gene name	Top blast hit	E value	% Coverage	% identity	Accession number
hypothetical protein F66182_1560	*Fusarium sp. NRRL 66182*	0	98%	69.55%	KAF5026352.1
hypothetical protein VDGD_21529	*Verticillium dahliae*	0	59%	94.22%	RBQ70014.1
polyketide synthase	*Stemphylium lycopersici*	0	84%	72.83%	KNG48001.1
putative polyketide synthase	*Stemphylium lycopersici*	0	96%	72.83%	RAR08647.1
polyketide synthase	*Stemphylium lycopersici*	0	96%	72.94%	RAR12984.1
putative polyketide synthase	*Fusarium babinda*	0	98%	65.77%	ALQ32784.1
putative polyketide synthase	*Fusarium austroafricanum*	0	98%	65.05%	KAF4442643.1
polyketide synthase	*Fusarium napiforme*	0	98%	64.19%	KAF5554444.1
polyketide synthase	*Fusarium tjaetaba*	0	98%	64.24%	XP_037202727.1
hypothetical protein IL306_006708	*Fusarium sp. DS 682*	0	98%	63.66%	KAF9775208.1

*V. longisporum_* VRPKS-I-1_contig_CVQH01027305					
Gene name	Top blast hit	E value	% Coverage	% identity	Accession number
polyketide synthase	Halenospora varia	0	99%	42.69%	KAH6673321.1
putative hybrid NRPS/PKS enzyme	Acephala macrosclerotiorum	0	99%	41.53%	KAF8856698.1
hypothetical protein BKA64DRAFT_721680	Leotiomycetes sp. MPI-SDFR-AT-0126	0	99%	40.98%	KAH7407938.1
hypothetical protein HETSPECPRED_009709	Heterodermia speciosa	0	99%	40.82%	CAF9935239.1
hypothetical protein BCR34DRAFT_634445	Clohesyomyces aquaticus	0	95%	41.71%	ORY17104.1
RecName: Full=Hybrid PKS-NRPS synthetase poxE; Short=PKS-NRPS; AltName: Full=Oxaleimides biosynthesis cluster protein E	Penicillium oxalicum 114-2	0	99%	41.08%	S7ZFK6.1
RecName: Full=Hybrid PKS-NRPS synthetase poxE; Short=PKS-NRPS; AltName: Full=Oxaleimides biosynthesis cluster protein E	Penicillium oxalicum	0	99%	41.03%	A0A1W5T1U1.1
hypothetical protein DL98DRAFT_604524	Cadophora sp. DSE1049	0	99%	40.36%	PVH74406.1
polyketide synthase	Rhexocercosporidium sp. MPI-PUGE-AT-0058	0	99%	40.33%	KAH7326983.1
putative hybrid NRPS/PKS enzyme	Aspergillus heteromorphus CBS 117.55	0	99%	41.14%	XP_025394747.1

*V. longisporum*_ VRPKS-I-1_contig_CVQH01003891					
Gene name	Top blast hit	E value	% Coverage	% identity	Accession number
putative hybrid NRPS/PKS enzyme	*Didymosphaeria enalia*	0	99%	49.05%	KAF2268646.1
polyketide synthase	*Halenospora varia*	0	94%	50.03%	KAH6673321.1
hypothetical protein KIY56_004012	*Pseudotulostoma volvatum*	0	94%	50.08%	KAG9375044.1
polyketide synthase	*Saccharata proteae CBS 121410*	0	94%	50.00%	KAF2084690.1
amino acid adenylation domain-containing protein	*Hymenoscyphus varicosporioides*	0	95%	49.76%	KAH8646967.1
Acetyl-CoA synthetase-like protein	*Glarea lozoyensis ATCC 20868*	0	99%	48.05%	XP_008078954.1
hypothetical protein HYFRA_00007390	*Hymenoscyphus fraxineus*	0	99%	47.56%	CAG8951474.1
related to polyketide synthase	*Rhynchosporium agropyri*	0	94%	47.73%	CZT02515.1
related to polyketide synthase	*Rhynchosporium commune*	0	94%	47.67%	CZS89930.1
hypothetical protein V495_06911	*Pseudogymnoascus sp. VKM F-4514 (FW-929)*	0	94%	48.66%	KFY37827.1

*V. nonalfalfae*_ VRPKS-I-22_NW_021167278					
Gene name	Species of blast hit	E value	Query Cover	Per. Ident	Accession
polyketide synthase [Fusarium mexicanum]	*Fusarium mexicanum*	0	99%	70.41%	KAF5539484.1
putative polyketide synthase [Fusarium dlaminii]	*Fusarium dlaminii*	0	99%	70.60%	ALQ32831.1
polyketide synthase [Fusarium subglutinans]	*Fusarium subglutinans*	0	99%	70.48%	XP_036534733.1
polyketide synthase [Fusarium acutatum]	*Fusarium acutatum*	0	99%	70.59%	KAF4428951.1
polyketide synthase [Fusarium pseudoanthophilum]	*Fusarium pseudoanthophilum*	0	100%	70.53%	KAF5573817.1
polyketide synthase [Fusarium denticulatum]	*Fusarium denticulatum*	0	100%	70.39%	KAF5687147.1
putative polyketide synthase [Fusarium bulbicola]	*Fusarium bulbicola*	0	99%	69.94%	KAF5977424.1
putative polyketide synthase [Fusarium sacchari]	*Fusarium sacchari*	0	99%	70.69%	ALQ32937.1
putative polyketide synthase [Fusarium succisae]	*Fusarium succisae*	0	99%	69.93%	ALQ33023.1
polyketide synthase [Fusarium mundagurra]	*Fusarium mundagurra*	0	99%	69.81%	KAF5716826.1

*V. nonalfalfae*_ VRPKS-I-17_NW_021167382					
Gene name	Species of blast hit	E value	Query Cover	Per. Ident	Accession
Lovastatin diketide synthase LovF 16 [Colletotrichum chlorophyti]	*Colletotrichum chlorophyti*	0	93%	70.29%	OLN86478.1

polyketide synthase [Colletotrichum truncatum]	*Colletotrichum truncatum*	0	93%	69.53%	XP_036585096.1
Reducing polyketide synthase PKS2 [Colletotrichum orbiculare MAFF 240422]	*Colletotrichum orbiculare MAFF 240422*	0	93%	69.57%	TDZ25780.1
Reducing polyketide synthase PKS2 [Colletotrichum sidae]	*Colletotrichum sidae*	0	93%	69.49%	TEA18683.1
Reducing polyketide synthase PKS2 [Colletotrichum trifolii]	*Colletotrichum trifolii*	0	93%	69.53%	TDZ28230.1
Reducing polyketide synthase PKS2 [Colletotrichum spinosum]	*Colletotrichum spinosum*	0	93%	69.44%	TDZ31026.1
beta-ketoacyl synthase domain-containing protein [Colletotrichum salicis]	*Colletotrichum salicis*	0	93%	69.44%	KXH66496.1
polyketide synthase [Colletotrichum musicola]	*Colletotrichum musicola*	0	93%	69.59%	KAF6830768.1
beta-ketoacyl synthase domain-containing protein [Colletotrichum fioriniae PJ7]	*Colletotrichum fioriniae PJ7*	0	93%	69.53%	EXF85385.1
beta-ketoacyl synthase domain-containing protein [Colletotrichum scovillei]	*Colletotrichum scovillei*	0	93%	69.53%	XP_035338972.1

*V. nonalfalfae*_ VRPKS-I-19_NW_021167282					
Gene name	Species of blast hit	E value	Query Cover	Per. Ident	Accession
hypothetical protein J1614_005792 [Leptosphaeria biglobosa]	*Leptosphaeria biglobosa*	0	99%	78.94%	KAH9873394.1
Reducing polyketide synthase PKS2 like protein [Verticillium longisporum]	*Verticillium longisporum*	0	76%	94.62%	KAG7126724.1
Type I Iterative PKS [Claviceps purpurea]	*Claviceps purpurea*	0	99%	62.24%	KAG6264852.1
Type I Iterative PKS [Claviceps purpurea]	*Claviceps purpurea*	0	99%	62.24%	KAG6168136.1
Type I Iterative PKS [Claviceps purpurea]	*Claviceps purpurea*	0	99%	62.15%	KAG6172346.1
Type I Iterative PKS [Claviceps purpurea]	*Claviceps purpurea*	0	99%	62.19%	KAG6257628.1
Type I Iterative PKS [Claviceps purpurea]	*Claviceps purpurea*	0	99%	62.19%	KAG6178588.1
Type I Iterative PKS [Claviceps purpurea]	*Claviceps purpurea*	0	99%	62.19%	KAG6152537.1
Type I Iterative PKS [Claviceps purpurea]	*Claviceps purpurea*	0	99%	62.10%	KAG6313294.1
Type I Iterative PKS [Claviceps purpurea]	*Claviceps purpurea*	0	99%	62.15%	KAG6319079.1

*V. nonalfalfae*_ VRPKS-I-23_NW_021167434					
Gene name	Species of blast hit	E value	Query Cover	Per. Ident	Accession
phenolpthiocerol synthesis polyketide synthase ppsA [Sodiomyces alkalinus F11]	*Sodiomyces alkalinus F11*	0	98%	50.04%	XP_028462492.1
phenolpthiocerol synthesis polyketide synthase ppsA [Plectosphaerella plurivora]	*Plectosphaerella plurivora*	0	99%	44.46%	KAH6697431.1
phenolpthiocerol synthesis polyketide synthase ppsA [Plectosphaerella cucumerina]	*Plectosphaerella cucumerina*	0	99%	44.51%	KAH7358949.1
beta-ketoacyl synthase domain-containing protein [Colletotrichum incanum]	*Colletotrichum incanum*	0	99%	42.83%	OHW97630.1

hypothetical protein BJF96_g3857 [Verticillium dahliae]	*Verticillium dahliae*	0	38%	94.05%	PNH33126.1
hypothetical protein G7046_g6445 [Stylonectria norvegica]	*Stylonectria norvegica*	0	99%	41.66%	KAF7555862.1
polyketide synthase (beta-ketoacyl synthase domain-containing protein) [Colletotrichum tofieldiae]	*Colletotrichum tofieldiae*	0	97%	42.90%	KZL67886.1
Lovastatin diketide synthase LovF 13 [Colletotrichum chlorophyti]	*Colletotrichum chlorophyti*	0	98%	42.52%	OLN85715.1
Reducing polyketide synthase FUB1 [Colletotrichum orbiculare MAFF 240422]	*Colletotrichum orbiculare MAFF 240422*	0	97%	43.44%	TDZ22788.1
beta-ketoacyl synthase domain-containing protein [Colletotrichum incanum]	*Colletotrichum incanum*	0	97%	42.83%	KZL83622.1

*V. nonalfalfae_* VRPKS-I-26_NW_021167247					
Gene name	Species of blast hit	E value	Query Cover	Per. Ident	Accession
hypothetical protein F5B20DRAFT_586947 [Whalleya microplaca]	*Whalleya microplaca*	0	97%	54.85%	KAI1073750.1
hypothetical protein DL768_003915 [Monosporascus sp. mg162]	*Monosporascus sp. mg162*	0	97%	54.66%	RYP50633.1
RecName: Full=Highly reducing polyketide synthase apmlA; Short=HRPKS apmlA; AltName: Full=Phaeospelide A biosynthesis cluster protein apmlA [Apiospora sphaerosperma]	*Apiospora sphaerosperma*	0	97%	50.20%	P0CU84.1
putative polyketide synthase [Xylariales sp. PMI_506]	*Xylariales sp. PMI_506*	0	93%	46.85%	KAH8657763.1
Fumagillin dodecapentaenoate synthase [Lachnellula willkommii]	*Lachnellula willkommii*	0	95%	45.68%	TVY89575.1
Fumagillin dodecapentaenoate synthase [Lachnellula arida]	*Lachnellula arida*	0	95%	45.54%	TVY19100.1
polyketide synthase 2 [Didymosphaeria enalia]	*Didymosphaeria enalia*	0	95%	45.61%	KAF2259783.1
hypothetical protein ACN38_g10438 [Penicillium nordicum]	*Penicillium nordicum*	0	96%	45.84%	KOS38742.1
Fumagillin dodecapentaenoate synthase [Lachnellula cervina]	*Lachnellula cervina*	0	95%	45.54%	TVY51056.1
hypothetical protein ACN42_g5244 [Penicillium freii]	*Penicillium freii*	0	95%	45.69%	KUM61888.1

*V. nonalfalfae_* VNRPKS-I-1_NW_021167324					
Gene name	Species of blast hit	E value	Query Cover	Per. Ident	Accession
Conidial yellow pigment biosynthesis polyketide synthase [Colletotrichum gloeosporioides]	*Colletotrichum gloeosporioides*	0	100%	75.72%	XP_045268234.1
beta-ketoacyl synthase [Colletotrichum sojae]	*Colletotrichum sojae*	0	100%	76.16%	KAF6807537.1
Conidial yellow pigment biosynthesis polyketide synthase [Colletotrichum tropicale]	*Colletotrichum tropicale*	0	100%	75.81%	KAF4827098.1
uncharacterized protein CkaCkLH20_00019 [Colletotrichum karsti]	*Colletotrichum karsti*	0	100%	75.93%	XP_038751444.1
hypothetical protein GQ607_010262 [Colletotrichum asianum]	*Colletotrichum asianum*	0	100%	75.76%	KAF0322599.1
hypothetical protein CcaCcLH18_10320 [Colletotrichum camelliae]	*Colletotrichum camelliae*	0	100%	75.49%	KAH0426510.1

Conidial yellow pigment biosynthesis polyketide synthase [Colletotrichum siamense]	*Colletotrichum siamense*	0	100%	75.67%	KAF4812766.1
Conidial yellow pigment biosynthesis polyketide synthase [Colletotrichum fructicola]	*Colletotrichum fructicola*	0	100%	75.72%	KAF4937522.1
Conidial yellow pigment biosynthesis polyketide synthase [Colletotrichum viniferum]	*Colletotrichum viniferum*	0	100%	75.72%	KAF4919737.1
hypothetical protein K456DRAFT_1724748 [Colletotrichum gloeosporioides 23]	*Colletotrichum gloeosporioides 23*	0	100%	75.67%	KAH9233890.1

*V. nonalfalfae*_ VNRPKS-I-3_NW_021167257					
Gene name	Species of blast hit	E value	Query Cover	Per. Ident	Accession
phenolpthiocerol synthesis polyketide synthase ppsA [Sodiomyces alkalinus F11]	*Sodiomyces alkalinus F11*	4.00E-131	87%	55.50%	XP_028463040.1
putative sterigmatocystin biosynthesis polyketide synthase [Tolypocladium ophioglossoides CBS 100239]	*Tolypocladium ophioglossoides CBS 100239*	6.00E-117	91%	47.20%	KND89106.1
Polyketide synthase [Tolypocladium paradoxum]	*Tolypocladium paradoxum*	2.00E-114	91%	46.47%	POR35294.1
hypothetical protein F4677DRAFT_278704 [Hypoxylon crocopeplum]	*Hypoxylon crocopeplum*	2.00E-100	92%	45.73%	KAI1372052.1
hypothetical protein F4802DRAFT_239318 [Xylaria palmicola]	*Xylaria palmicola*	2.00E-99	92%	48.00%	KAI0409182.1
hypothetical protein VDGD_20147 [Verticillium dahliae]	*Verticillium dahliae*	4.00E-99	35%	94.04%	RBQ97855.1
Beta-ketoacyl synthase [Macrophomina phaseolina MS6]	*Macrophomina phaseolina MS6*	3.00E-98	90%	42.56%	EKG13225.1
hypothetical protein GGR53DRAFT_500984 [Hypoxylon sp. FL1150]	*Hypoxylon sp. FL1150*	8.00E-96	83%	46.54%	KAI1762632.1
hypothetical protein B0I35DRAFT_407015 [Stachybotrys elegans]	*Stachybotrys elegans*	2.00E-95	91%	43.86%	KAH7322541.1
hypothetical protein F4680DRAFT_463425 [Xylaria scruposa]	*Xylaria scruposa*	5.00E-95	91%	44.19%	KAI1746119.1

*V. nonalfalfae*_PKS-III_NW_021167286					
Gene name	Species of blast hit	E value	Query Cover	Per. Ident	Accession
putative stilbene synthase 2 [Colletotrichum chlorophyti]	*Colletotrichum chlorophyti*	0	100%	72.91%	OLN95986.1
chalcone and stilbene synthase domain-containing protein [Colletotrichum graminicola M1.001]	*Colletotrichum graminicola M1.001*	0	100%	72.23%	XP_008099452.1
chalcone and stilbene synthase domain-containing protein [Colletotrichum tofieldiae]	*Colletotrichum tofieldiae*	0	100%	72.40%	GKT65818.1
chalcone and stilbene synthase domain-containing protein [Plectosphaerella cucumerina]	*Plectosphaerella cucumerina*	0	100%	73.08%	KAH7375301.1
Chalcone synthase [Colletotrichum tanaceti]	*Colletotrichum tanaceti*	0	100%	72.62%	TKW57803.1
chalcone synthase [Colletotrichum spaethianum]	*Colletotrichum spaethianum*	0	100%	72.17%	GJC81220.1
chalcone and stilbene synthase domain-containing protein [Colletotrichum tofieldiae]	*Colletotrichum tofieldiae*	0	100%	72.40%	KZL74511.1

chalcone synthase [Colletotrichum liriopes]	*Colletotrichum liriopes*	0	100%	72.17%	GKT49639.1
putative chalcone and stilbene synthase domain-containing protein [Colletotrichum sublineola]	*Colletotrichum sublineola*	0	100%	71.49%	KDN65911.1
chalcone and stilbene synthase domain-containing protein [Colletotrichum incanum]	*Colletotrichum incanum*	0	100%	72.17%	KZL80557.1

*V. nonalfalfae_* VRPKS-I-11_NW_021167275					
Gene name	Species of blast hit	E value	Query Cover	Per. Ident	Accession
Nonribosomal peptide synthetase-like protein [Acremonium chrysogenum ATCC 11550]	*Acremonium chrysogenum ATCC 11550*	0	100%	47.99%	KFH45364.1
hypothetical protein F5Y06DRAFT_297186 [Hypoxylon sp. FL0890]	*Hypoxylon sp. FL0890*	0	99%	46.42%	KAI0837758.1
uncharacterized protein BCR38DRAFT_413069 [Pseudomassariella vexata]	*Pseudomassariella vexata*	0	100%	44.67%	XP_040711562.1
polyketide synthase [Lasiodiplodia theobromae]	*Lasiodiplodia theobromae*	0	99%	43.48%	KAF9630276.1
Fusarin C synthetase [Lasiodiplodia theobromae]	*Lasiodiplodia theobromae*	0	99%	43.52%	KAB2573439.1
Polyketide synthase [Lasiodiplodia theobromae]	*Lasiodiplodia theobromae*	0	99%	43.27%	XP_035374836.1
uncharacterized protein K452DRAFT_243204 [Aplosporella prunicola CBS 121167]	*Aplosporella prunicola CBS 121167*	0	99%	42.98%	XP_033401020.1
hypothetical protein Egran_04668 [Elaphomyces granulatus]	*Elaphomyces granulatus*	0	99%	42.09%	OXV07567.1
polyketide synthase, putative [Talaromyces stipitatus ATCC 10500]	*Talaromyces stipitatus ATCC 10500*	0	99%	40.75%	XP_002478535.1
putative polyketide synthase [Aspergillus sclerotioniger CBS 115572]	*Aspergillus sclerotioniger CBS 115572*	0	99%	41.77%	XP_025471949.1

*V. nonalfalfae_* VRPKS-I-9_NW_021167275					
Gene name	Species of blast hit	E value	Query Cover	Per. Ident	Accession
AMP-binding enzyme domain-containing protein [Hirsutella rhossiliensis]	*Hirsutella rhossiliensis*	0	99%	70.68%	XP_044717051.1
hypothetical protein HIM_06709 [Hirsutella minnesotensis 3608]	*Hirsutella minnesotensis 3608*	0	99%	63.63%	KJZ73816.1
lovastatin nonaketide synthase [Mollisia scopiformis]	*Mollisia scopiformis*	0	99%	48.67%	XP_018063373.1
lovastatin nonaketide synthase [Hypoxylon cercidicola]	*Hypoxylon cercidicola*	0	99%	45.81%	KAI1772910.1
lovastatin nonaketide synthase [Dactylonectria macrodidyma]	*Dactylonectria macrodidyma*	0	99%	53.69%	KAH7176829.1
polyketide synthase 3 [Aspergillus steynii IBT 23096]	*Aspergillus steynii IBT 23096*	0	91%	45.41%	XP_024699799.1
lovastatin nonaketide synthase [Phaeosphaeria sp. MPI-PUGE-AT-0046c]	*Phaeosphaeria sp. MPI-PUGE-AT-0046c*	0	99%	43.27%	KAH7408173.1
putative Hybrid PKS-NRPS biosynthetic cluster [Verticillium nonalfalfae]	*Verticillium nonalfalfae*	0	94%	97.35%	XP_028495009.1
lovastatin nonaketide synthase [Aspergillus novofumigatus IBT 16806]	*Aspergillus novofumigatus IBT 16806*	0	99%	52.28%	XP_024677790.1

lovastatin nonaketide synthase [Hypoxylon trugodes]	*Hypoxylon trugodes*	0	99%	49.95%	KAI1387791.1

*V. nubilum_* VRPKS-I-17_contig_NMXI01000001					
Gene name	E value	% Coverage	% identity	Top blast hit	Accession number
Lovastatin diketide synthase LovF 16	0	99%	70.16%	*Colletotrichum chlorophyti*	OLN86478.1
polyketide synthase	0	99%	69.63%	*Colletotrichum truncatum*	XP_036585096.1
Reducing polyketide synthase PKS2	0	99%	69.49%	*Colletotrichum orbiculare MAFF 240422*	TDZ25780.1
Reducing polyketide synthase PKS2	0	99%	69.45%	*Colletotrichum sidae*	TEA18683.1
Reducing polyketide synthase PKS2	0	99%	69.55%	*Colletotrichum trifolii*	TDZ28230.1
Reducing polyketide synthase PKS2	0	99%	69.41%	*Colletotrichum spinosum*	TDZ31026.1
polyketide synthase	0	99%	69.85%	*Colletotrichum musicola*	KAF6830768.1
polyketide synthase	0	99%	69.85%	*Colletotrichum sojae*	KAF6806435.1
beta-ketoacyl synthase domain-containing protein	0	99%	69.77%	*Colletotrichum salicis*	KXH66496.1
polyketide synthase	0	99%	69.85%	*Colletotrichum plurivorum*	KAF6821754.1

*V. nubilum_* VRPKS-I-10_contig_NMXI01000076					
Gene name	E value	% Coverage	% identity	Top blast hit	Accession number
beta-ketoacyl synthase domain-containing protein	0	99%	54.33%	*Coniochaeta sp. PMI_546*	KAH8906691.1
beta-ketoacyl synthase domain-containing protein	0	99%	54.00%	*Coniochaeta ligniaria NRRL 30616*	OIW27845.1
putative polyketide synthase	0	99%	53.11%	*Coniochaeta sp. 2T2.1*	KAB5585722.1
putative polyketide synthase	0	99%	53.19%	*Coniochaeta sp. 2T2.1*	KAB5583401.1
uncharacterized protein SMAC_05695	0	98%	52.35%	*Sordaria macrospora k-hell*	XP_003348600.1
putative polyketide synthase	0	98%	52.27%	*Sordaria sp. MPI-SDFR-AT-0083*	KAH7628050.1
hypothetical protein NEUTE1DRAFT_85360	0	98%	51.78%	*Neurospora tetrasperma FGSC 2508*	XP_009853076.1
polyketide synthase 4	0	98%	51.67%	*Neurospora crassa OR74A*	XP_011395279.1
hypothetical protein M426DRAFT_261564	0	99%	50.77%	*Hypoxylon sp. CI-4A*	OTB04920.1
beta-ketoacyl synthase domain-containing protein	0	99%	51.69%	*Hypoxylon sp. FL0890*	KAI0834000.1

*V. nubilum_* VNRPKS-I-4_contig_NMXI01000091					
Gene name	E value	% Coverage	% identity	Top blast hit	Accession number
putative FSP1	0	99%	68.09%	*Dactylonectria macrodidyma*	KAH7133936.1
putative FSP1	0	99%	66.55%	*Ilyonectria destructans*	KAH7019732.1
putative polyketide synthase	0	99%	61.40%	*Fusarium sacchari*	ALQ32938.1

polyketide synthase	0	99%	61.68%	*Fusarium avenaceum*	KIL88308.1
beta-ketoacyl synthase domain-containing protein	0	99%	61.80%	*Fusarium proliferatum*	KAG4291688.1
polyketide synthase	0	99%	61.11%	*Fusarium subglutinans*	XP_036537074.1
putative polyketide synthase	0	99%	61.09%	*Fusarium anthophilum*	ALQ32742.1
polyketide synthase	0	99%	60.89%	*Fusarium coicis*	KAF5983031.1
putative type I polyketide synthase	0	99%	61.47%	*Fusarium fujikuroi*	KLP04343.1
hypothetical protein LB506_008106	0	99%	61.65%	*Fusarium annulatum*	KAI1066549.1

*V. nubilum_* VRPKS-I-15_contig_NMXI01000091					
Gene name	E value	% Coverage	% identity	Top blast hit	Accession number
hypothetical protein EDB80DRAFT_632905	0	87%	68.74%	*Ilyonectria destructans*	KAH7019735.1
hypothetical protein F5Y06DRAFT_304448	0	88%	65.45%	*Hypoxylon sp. FL0890*	KAI0842739.1
hypothetical protein F5Y05DRAFT_417605	0	88%	64.94%	*Hypoxylon sp. FL0543*	KAI1134063.1
hypothetical protein F5Y13DRAFT_179997	0	88%	64.96%	*Hypoxylon sp. FL1857*	KAI1412489.1
hypothetical protein IL306_014406	0	87%	62.92%	*Fusarium sp. DS 682*	KAF9768315.1
polyketide synthase	0	87%	62.15%	*Fusarium avenaceum*	KIL88312.1
hypothetical protein DER45DRAFT_636326	0	87%	61.93%	*Fusarium avenaceum*	KAH6952055.1
lijE	0	87%	62.58%	*Ascomycota sp. F53*	AMJ52084.1
Type I Iterative Polyketide synthase (PKS)	0	87%	60.74%	*Pseudogymnoascus sp. WSF 3629*	OBT39041.1
Type I Iterative Polyketide synthase (PKS)	0	87%	60.12%	*Pseudogymnoascus destructans*	XP_024319559.1

*V. nubilum_* VRPKS-I-19_contig_NMXI01000024					
Gene name	E value	% Coverage	% identity	Top blast hit	Accession number
hypothetical protein J1614_005792	0	100%	85.90%	*Leptosphaeria biglobosa*	KAH9873394.1
Type I Polyketide synthase	0	99%	81.78%	*Verticillium nonalfalfae*	XP_028494609.1
Reducing polyketide synthase PKS2 like protein	0	99%	81.74%	*Verticillium longisporum*	KAG7143635.1
fatty acid synthase S-acetyltransferase	0	99%	81.65%	*Verticillium dahliae*	KAH6703869.1
hypothetical protein VD0004_g7884	0	99%	81.37%	*Verticillium dahliae*	PNH38983.1
fatty acid synthase S-acetyltransferase	0	99%	80.96%	*Verticillium dahliae VdLs.17*	XP_009655626.1
hypothetical protein BN1708_003179	0	99%	81.10%	*Verticillium longisporum*	CRK18834.1
Inositol 2-dehydrogenase 2	0	96%	81.33%	*Verticillium dahliae VDG2*	KAF3346614.1
hypothetical protein BN1708_009954	0	88%	81.02%	*Verticillium longisporum*	CRK10893.1
hypothetical protein VDGD_03466	0	87%	81.00%	*Verticillium dahliae*	RBQ87991.1

*V. nubilum_* VRPKS-I-21_contig_NMXI01000024					
Gene name	E value	% Coverage	% identity	Top blast hit	Accession number
Lovastatin diketide synthase LovF 2	0	100%	70.18%	*Colletotrichum chlorophyti*	OLN91972.1
beta-ketoacyl synthase domain-containing protein	0	100%	69.48%	*Colletotrichum fioriniae PJ7*	EXF80608.1
polyketide synthase	0	99%	69.99%	*Colletotrichum plurivorum*	KAF6826616.1
beta-ketoacyl synthase domain-containing protein	0	100%	70.03%	*Colletotrichum scovillei*	KAG7053867.1
polyketide synthase	0	99%	69.91%	*Colletotrichum musicola*	KAF6845034.1
polyketide synthase	0	99%	69.95%	*Colletotrichum sojae*	KAF6809610.1
beta-ketoacyl synthase domain-containing protein	0	100%	69.75%	*Colletotrichum simmondsii*	KXH36687.1
beta-ketoacyl synthase domain-containing protein	0	100%	69.50%	*Colletotrichum scovillei*	XP_035330138.1
polyketide synthase (beta-ketoacyl synthase domain-containing protein)	0	100%	69.18%	*Colletotrichum tofieldiae*	KZL70949.1

*V. nubilum_* VRPKS-I-26_contig_NMXI01000023					
Gene name	E value	% Coverage	% identity	Top blast hit	Accession number
hypothetical protein F5B20DRAFT_586947	0	99%	51.02%	*Whalleya microplaca*	KAI1073750.1
hypothetical protein BN1708_001184	0	85%	62.42%	*Verticillium longisporum*	CRK33507.1
hypothetical protein DL768_003915	0	99%	50.15%	*Monosporascus sp. mg162*	RYP50633.1
mycocerosic acid synthase	0	97%	65.61%	*Verticillium dahliae VdLs.17*	XP_009654470.1
RecName: Full=Highly reducing polyketide synthase apmlA; Short=HRPKS apmlA; AltName: Full=Phaeospelide A biosynthesis cluster protein apmlA	0	99%	49.11%	*Apiospora sphaerosperma*	P0CU84.1
D-amino-acid oxidase	0	80%	73.86%	*Verticillium dahliae VDG1*	KAF3356735.1
Highly reducing polyketide synthase apmlA like protein	0	74%	80.72%	*Verticillium longisporum*	KAG7100701.1
Highly reducing polyketide synthase apmlA like protein	0	45%	81.65%	*Verticillium longisporum*	KAG7141377.1
hypothetical protein VDGD_07270	0	46%	78.75%	*Verticillium dahliae*	RBQ99134.1
hypothetical protein ACN42_g5244	0	99%	41.93%	*Penicillium freii*	KUM61888.1

*V. nubilum_* VRPKS-I-23_contig_NMXI01000025					
Gene name	E value	% Coverage	% identity	Top blast hit	Accession number
phenolpthiocerol synthesis polyketide synthase ppsA	0	99%	50.96%	*Sodiomyces alkalinus F11*	XP_028462492.1
polyketide synthase	0	95%	67.90%	*Verticillium nonalfalfae*	XP_028490525.1
lovastatin nonaketide synthase	0	86%	68.52%	*Verticillium alfalfae VaMs.102*	XP_003007505.1
phenolpthiocerol synthesis polyketide synthase ppsA	0	99%	45.67%	*Plectosphaerella plurivora*	KAH6697431.1
phenolpthiocerol synthesis polyketide synthase ppsA	0	99%	46.03%	*Plectosphaerella cucumerina*	KAH7358949.1

beta-ketoacyl synthase domain-containing protein	0	99%	43.74%	*Colletotrichum incanum*	OHW97630.1
beta-ketoacyl synthase domain-containing protein	0	97%	43.76%	*Colletotrichum graminicola M1.001*	XP_008090783.1
polyketide synthase (beta-ketoacyl synthase domain-containing protein)	0	97%	43.93%	*Colletotrichum tofieldiae*	KZL67886.1
Reducing polyketide synthase FUB1	0	97%	43.65%	*Colletotrichum gloeosporioides*	XP_045256339.1
beta-ketoacyl synthase domain-containing protein	0	97%	42.96%	*Colletotrichum karsti*	XP_038741012.1

*V. nubilum*_PKS-III_contig_NMXI01000041					
Gene name	E value	% Coverage	% identity	Top blast hit	Accession number
chalcone and stilbene synthase domain-containing protein	0	100%	75.45%	*Plectosphaerella cucumerina*	KAH7375301.1
type iii polyketide synthase	0	99%	75.79%	*Colletotrichum camelliae*	KAH0440190.1
chalcone and stilbene synthase domain-containing protein	0	100%	74.43%	*Colletotrichum graminicola M1.001*	XP_008099452.1
Stilbene synthase 3	0	99%	75.79%	*Colletotrichum siamense*	KAF4811812.1
putative stilbene synthase 2	0	100%	74.43%	*Colletotrichum chlorophyti*	OLN95986.1
Type III polyketide synthase B	0	98%	75.97%	*Colletotrichum gloeosporioides*	XP_045260893.1
putative chalcone and stilbene synthase domain-containing protein	0	100%	74.43%	*Colletotrichum sublineola*	KDN65911.1
hypothetical protein CGLO_04899	0	98%	75.97%	*Colletotrichum gloeosporioides Cg-14*	EQB55203.1
Stilbene synthase 3	0	100%	73.87%	*Colletotrichum aenigma*	XP_037171847.1
Stilbene synthase 3	0	99%	75.34%	*Colletotrichum siamense*	XP_036488664.1

*V. nubilum*_ VRPKS-I-4_contig_NMXI01000015					
Gene name	E value	% Coverage	% identity	Top blast hit	Accession number
hypothetical protein F4775DRAFT_593279	0	99%	58.01%	*Biscogniauxia sp. FL1348*	KAI0597589.1
hypothetical protein F5X96DRAFT_674085	0	99%	58.07%	*Biscogniauxia mediterranea*	KAI1485991.1
hypothetical protein F4809DRAFT_640890	0	99%	57.96%	*Biscogniauxia mediterranea*	KAI1637132.1
hypothetical protein BOTCAL_0139g00080	0	99%	56.29%	*Botryotinia calthae*	TEY65541.1
hypothetical protein BTUL_0001g00530	0	99%	56.11%	*Botrytis tulipae*	TGO20046.1
hypothetical protein BGAL_0408g00010	0	99%	55.70%	*Botrytis galanthina*	THV46179.1
hypothetical protein F5144DRAFT_655621	0	99%	54.94%	*Chaetomium globosum*	KAH6628919.1
hypothetical protein B7494_g1325	0	99%	52.28%	*Chlorociboria aeruginascens*	TAQ90374.1
Acyl transferase acyl hydrolase lysophospholipase protein	0	99%	45.98%	*Rutstroemia sp. NJR-2017a BVV2*	PQE27755.1
hypothetical protein CJF32_00005378	0	99%	46.24%	*Rutstroemia sp. NJR-2017a WRK4*	PQE13487.1

*V. tricorpus*_ VRPKS-I-17_contig_JPET01000002					
Gene name	Species of BLAST hit	E-value	% coverage	% identity	Accession number
Lovastatin diketide synthase LovF 16	*Colletotrichum chlorophyti*	0	92%	71.98%	OLN86478.1
polyketide synthase	*Colletotrichum truncatum*	0	92%	71.55%	XP_036585096.1
Reducing polyketide synthase PKS2	*Colletotrichum trifolii*	0	92%	71.46%	TDZ28230.1
Reducing polyketide synthase PKS2	*Colletotrichum orbiculare MAFF 240422*	0	92%	71.42%	TDZ25780.1
Reducing polyketide synthase PKS2	*Colletotrichum sidae*	0	92%	71.37%	TEA18683.1
Reducing polyketide synthase PKS2	*Colletotrichum spinosum*	0	92%	71.33%	TDZ31026.1
polyketide synthase	*Colletotrichum sojae*	0	92%	71.47%	KAF6806435.1
polyketide synthase	*Colletotrichum musicola*	0	92%	71.68%	KAF6830768.1
fatty acid synthase S-acetyltransferase	*Verticillium alfalfae VaMs.102*	0	91%	82.56%	XP_003008481.1
beta-ketoacyl synthase domain-containing protein	*Colletotrichum nymphaeae SA-01*	0	92%	71.14%	KXH49587.1

*V. tricorpus*_ VRPKS-I-14_contig_JPET01000002					
Gene name	Species of BLAST hit	E-value	% coverage	% identity	Accession number
putative polyketide synthase	*Truncatella angustata*	0	100%	57.90%	XP_045957259.1
hypothetical protein AJ80_00462	*Polytolypa hystricis UAMH7299*	0	100%	55.29%	PGH27912.1
Nonribosomal peptide synthetase-like protein	*Acremonium chrysogenum ATCC 11550*	0	99%	54.23%	KFH40504.1
uncharacterized protein B0I36DRAFT_397789	*Microdochium trichocladiopsis*	0	98%	49.14%	XP_046005243.1
hypothetical protein TruAng_007522	*Truncatella angustata*	0	97%	54.99%	KAH8198320.1
Polyketide synthase	*Penicillium sp. RFL-2021a*	0	99%	47.71%	KAF7713846.1
hypothetical protein PDE_03926	*Penicillium oxalicum 114-2*	0	100%	47.78%	EPS28980.1
Compactin diketide synthase mokB	*Penicillium rolfsii*	0	99%	47.57%	KAF3388919.1
hypothetical protein PMG11_09333	*Penicillium brasilianum*	0	99%	47.54%	CEJ60771.1
Lovastatin diketide synthase LovF	*Penicillium subrubescens*	0	99%	47.83%	OKP11696.1

*V. tricorpus*_ VRPKS-I-16_contig_JPET01000001					
Gene name	Species of BLAST hit	E-value	% coverage	% identity	Accession number
putative polyketide synthase	*Plectosphaerella cucumerina*	0	99%	72.03%	KAH7369281.1
putative polyketide synthase	*Plectosphaerella plurivora*	0	99%	71.33%	KAH6673997.1
hypothetical protein K402DRAFT_461387	*Aulographum hederae CBS 113979*	0	99%	49.89%	KAF1988946.1
uncharacterized protein K444DRAFT_592521	*Hyaloscypha bicolor E*	0	99%	47.85%	XP_024734639.1

hypothetical protein IFR05_000103	*Cadophora sp. M221*	0	99%	47.63%	KAG4444512.1
putative polyketide synthase	*Tothia fuscella*	0	99%	47.70%	KAF2419578.1
hypothetical protein CNMCM7691_001823	*Aspergillus felis*	0	99%	46.16%	KAF7182343.1
hypothetical protein CDV56_109033	*Aspergillus thermomutatus*	0	99%	45.61%	XP_026617693.1
hypothetical protein IFM61606_05003	*Aspergillus udagawae*	0	99%	45.39%	GFG25074.1
hypothetical protein CNMCM5623_002475	*Aspergillus felis*	0	99%	45.58%	KAF7169924.1

*V. tricorpus_* VRPKS-I-25_contig_JPET01000001					
Gene name	Species of BLAST hit	E-value	% coverage	% identity	Accession number
hypothetical protein S40293_03578	*Stachybotrys chartarum IBT 40293*	0	99%	89.27%	KFA46917.1
hypothetical protein S40288_08039	*Stachybotrys chartarum IBT 40288*	0	99%	87.31%	KFA72420.1
hypothetical protein S7711_03851	*Stachybotrys chartarum IBT 7711*	0	86%	88.90%	KEY68922.1
hypothetical protein DL764_002272	*Monosporascus ibericus*	0	99%	56.31%	RYP07817.1
hypothetical protein B7494_g6164	*Chlorociboria aeruginascens*	0	99%	56.44%	TAQ85507.1
hypothetical protein DL770_006186	*Monosporascus sp. CRB-9-2*	0	99%	56.73%	RYP80513.1
type I iterative PKS	*Aspergillus luchuensis*	0	99%	55.87%	XP_041547082.1
polyketide synthase	*Aspergillus costaricaensis CBS 115574*	0	99%	55.54%	XP_025542753.1
polyketide synthase	*Aspergillus niger*	0	99%	55.38%	GAQ46573.1
polyketide synthase	*Aspergillus neoniger CBS 115656*	0	99%	55.54%	XP_025484624.1

*V. tricorpus_* VRPKS-I-19_contig_JPET01000001					
Gene name	Species of BLAST hit	E-value	% coverage	% identity	Accession number
hypothetical protein J1614_005792	*Leptosphaeria biglobosa*	0	99%	78.03%	KAH9873394.1
Type I Polyketide synthase	*Verticillium nonalfalfae*	0	100%	77.17%	XP_028494609.1
Reducing polyketide synthase PKS2 like protein	*Verticillium longisporum*	0	100%	77.12%	KAG7143635.1
hypothetical protein VD0004_g7884	*Verticillium dahliae*	0	100%	77.08%	PNH38983.1
fatty acid synthase S-acetyltransferase	*Verticillium dahliae*	0	100%	77.17%	KAH6703869.1
fatty acid synthase S-acetyltransferase	*Verticillium dahliae VdLs.17*	0	100%	76.48%	XP_009655626.1
hypothetical protein BN1708_003179	*Verticillium longisporum*	0	100%	76.56%	CRK18834.1
Inositol 2-dehydrogenase 2	*Verticillium dahliae VDG2*	0	96%	76.72%	KAF3346614.1
hypothetical protein BN1708_009954	*Verticillium longisporum*	0	88%	76.32%	CRK10893.1
hypothetical protein VDGD_03466	*Verticillium dahliae*	0	87%	76.66%	RBQ87991.1

*V. tricorpus_* VRPKS-I-13_contig_JPET01000003					
Gene name	Species of BLAST hit	E-value	% coverage	% identity	Accession number
polyketide synthase	*Fusarium avenaceum*	0	99%	55.19%	KIL85244.1
hypothetical protein BKA59DRAFT_387557	*Fusarium tricinctum*	0	99%	55.01%	KAH7261591.1
hypothetical protein DER45DRAFT_513901	*Fusarium avenaceum*	0	99%	54.98%	KAH6968136.1
putative polyketide synthase	*Fusarium redolens*	0	99%	54.93%	ALQ32917.1
unnamed protein product	*Fusarium equiseti*	0	99%	55.00%	CAG7561821.1
polyketide synthase	*Fusarium beomiforme*	0	99%	54.58%	KAF4341752.1
hypothetical protein FGRMN_6861	*Fusarium graminum*	0	99%	54.51%	KAF4992917.1
uncharacterized protein FVRRES_09355	*Fusarium venenatum*	0	99%	54.52%	XP_025592993.1
hypothetical protein EDB82DRAFT_480382	*Fusarium venenatum*	0	99%	54.49%	KAH6966040.1
uncharacterized protein BKA55DRAFT_692368	*Fusarium redolens*	0	99%	54.74%	XP_046047791.1

*V. tricorpus_* VRPKS-I-26_contig_JPET01000004					
Gene name	Species of BLAST hit	E-value	% coverage	% identity	Accession number
hypothetical protein PENARI_c014G02915	*Penicillium arizonense*	0.00E+00	99%	67.08%	XP_022486633.1
uncharacterized protein BGW36DRAFT_428643	*Talaromyces proteolyticus*	0	99%	52.82%	XP_046071584.1
hypothetical protein ABOM_007875	*Aspergillus bombycis*	0.00E+00	99%	51.68%	XP_022386052.1
hypothetical protein BDV39DRAFT_200138	*Aspergillus sergii*	0	99%	51.14%	KAE8332518.1
Type I Iterative PKS	*Purpureocillium takamizusanense*	0	98%	52.22%	UNI20497.1
hypothetical protein CHU98_g7784	*Xylaria longipes*	0	99%	51.02%	RYC58429.1
KR domain protein	*Aspergillus parasiticus SU-1*	0	99%	50.88%	KJK67167.1
hypothetical protein BDV41DRAFT_584626	*Aspergillus transmontanensis*	0	99%	50.69%	KAE8317099.1
hypothetical protein G6O67_003088	*Ophiocordyceps sinensis*	0	98%	51.97%	KAF4511277.1
Type I Iterative PKS	*Eutypa lata*	0	99%	49.18%	KAI1254450.1

*V. tricorpus_*PKS-III_contig_JPET01000004					
Gene name	Species of BLAST hit	E-value	% coverage	% identity	Accession number
putative chalcone and stilbene synthase domain-containing protein	*Colletotrichum sublineola*	0	100%	73.65%	KDN65911.1
chalcone and stilbene synthase domain-containing protein	*Plectosphaerella cucumerina*	0	100%	75.45%	KAH7375301.1
chalcone and stilbene synthase domain-containing protein	*Colletotrichum tofieldiae*	0	100%	73.65%	GKT65818.1

chalcone synthase	*Colletotrichum spaethianum*	0	100%	73.42%	GJC81220.1
chalcone and stilbene synthase domain-containing protein	*Colletotrichum graminicola M1.001*	0	100%	73.20%	XP_008099452.1
chalcone and stilbene synthase domain-containing protein	*Colletotrichum tofieldiae*	0	100%	73.42%	KZL74511.1
chalcone and stilbene synthase	*Colletotrichum simmondsii*	0	100%	72.75%	KXH46773.1
chalcone and stilbene synthase domain-containing protein	*Colletotrichum incanum*	0	100%	73.42%	KZL80557.1
chalcone and stilbene synthase	*Colletotrichum scovillei*	0	100%	72.52%	XP_035333635.1
putative stilbene synthase 2	*Colletotrichum chlorophyti*	0	100%	72.07%	OLN95986.1

*V. tricorpus_* VRPKS-I-23_contig_JPET01000002					
Gene name	Species of BLAST hit	E-value	% coverage	% identity	Accession number
phenolpthiocerol synthesis polyketide synthase ppsA	*Sodiomyces alkalinus F11*	0	99%	51.56%	XP_028462492.1
polyketide synthase	*Verticillium nonalfalfae*	0	95%	64.92%	XP_028490525.1
phenolpthiocerol synthesis polyketide synthase ppsA	*Plectosphaerella plurivora*	0	99%	46.02%	KAH6697431.1
phenolpthiocerol synthesis polyketide synthase ppsA	*Plectosphaerella cucumerina*	0	99%	45.54%	KAH7358949.1
beta-ketoacyl synthase domain-containing protein	*Colletotrichum incanum*	0	99%	44.85%	OHW97630.1
Lovastatin diketide synthase LovF 13	*Colletotrichum chlorophyti*	0	99%	44.06%	OLN85715.1
beta-ketoacyl synthase domain-containing protein	*Colletotrichum karsti*	0	98%	44.21%	XP_038741012.1
beta-ketoacyl synthase domain-containing protein	*Colletotrichum graminicola M1.001*	0	98%	45.27%	XP_008090783.1
polyketide synthase (beta-ketoacyl synthase domain-containing protein)	*Colletotrichum tofieldiae*	0	98%	44.92%	KZL67886.1
Reducing polyketide synthase FUB1	*Colletotrichum orbiculare MAFF 240422*	0	98%	44.64%	TDZ22788.1

*V. tricorpus_* VRPKS-I-12_contig_JPET01000002					
Gene name	Species of BLAST hit	E-value	% coverage	% identity	Accession number
polyketide synthase	*Sodiomyces alkalinus F11*	0	96%	75.43%	XP_028469272.1
beta-ketoacyl synthase domain-containing protein	*Colletotrichum fioriniae PJ7*	0	96%	72.70%	EXF80071.1
beta-ketoacyl synthase domain-containing protein	*Colletotrichum salicis*	0	96%	72.61%	KXH33485.1
beta-ketoacyl synthase domain-containing protein	*Colletotrichum nymphaeae SA-01*	0	96%	72.56%	KXH30697.1
beta-ketoacyl synthase domain-containing protein	*Colletotrichum scovillei*	0	96%	72.52%	XP_035338903.1
beta-ketoacyl synthase domain-containing protein	*Colletotrichum orchidophilum*	0	96%	72.49%	XP_022470047.1
beta-ketoacyl synthase domain-containing protein	*Colletotrichum simmondsii*	0	96%	72.52%	KXH41720.1
polyketide synthase	*Pochonia chlamydosporia 170*	0	96%	65.91%	XP_018147207.1
hypothetical protein G7Z17_g6123	*Cylindrodendrum hubeiense*	0	96%	65.64%	KAF7549858.1

polyketide synthase	*Thelonectria olida*	0	96%	65.90%	KAH6899708.1

*V. tricorpus_* VNRPKS-I-1_contig_JPET01000002					
Gene name	Species of BLAST hit	E-value	% coverage	% identity	Accession number
uncharacterized protein CkaCkLH20_00019	*Colletotrichum karsti*	0	100%	79.19%	XP_038751444.1
hypothetical protein CGLO_05047	*Colletotrichum gloeosporioides Cg-14*	0	100%	79.05%	EQB55056.1
hypothetical protein GQ607_010262	*Colletotrichum asianum*	0	100%	79.01%	KAF0322599.1
hypothetical protein K456DRAFT_1724748	*Colletotrichum gloeosporioides 23*	0	100%	79.01%	KAH9233890.1
Conidial yellow pigment biosynthesis polyketide synthase	*Colletotrichum fructicola*	0	100%	79.01%	KAF4937522.1
hypothetical protein CcaCcLH18_10320	*Colletotrichum camelliae*	0	100%	79.05%	KAH0426510.1
Conidial yellow pigment biosynthesis polyketide synthase	*Colletotrichum gloeosporioides*	0	100%	79.01%	XP_045268234.1
Conidial yellow pigment biosynthesis polyketide synthase	*Colletotrichum fructicola*	0	100%	78.96%	XP_031880204.1
Conidial yellow pigment biosynthesis polyketide synthase	*Colletotrichum viniferum*	0	100%	78.96%	KAF4919737.1
Conidial yellow pigment biosynthesis polyketide synthase	*Colletotrichum siamense*	0	100%	78.91%	KAF4812766.1

*V. tricorpus_* VRPKS-I-5_contig_JPET01000008					
Gene name	Species of BLAST hit	E-value	% coverage	% identity	Accession number
AMP-binding enzyme domain-containing protein	*Hirsutella rhossiliensis*	0	99%	71.29%	XP_044717051.1
lovastatin nonaketide synthase	*Verticillium alfalfae VaMs.102*	0	100%	68.07%	XP_003005392.1
hypothetical protein HIM_06709	*Hirsutella minnesotensis 3608*	0	99%	64.00%	KJZ73816.1
lovastatin nonaketide synthase	*Aspergillus novofumigatus IBT 16806*	0	100%	53.29%	XP_024677790.1
lovastatin nonaketide synthase	*Hypoxylon trugodes*	0	99%	51.85%	KAI1387791.1
lovastatin nonaketide synthase	*Dactylonectria macrodidyma*	0	98%	55.88%	KAH7176829.1
hypothetical protein DL768_008516	*Monosporascus sp. mg162*	0	99%	50.60%	RYP45085.1
hypothetical protein JX265_007707	*Wardomyces moseri*	0	99%	49.76%	KAI1866406.1
lovastatin nonaketide synthase	*Mollisia scopiformis*	0	99%	47.61%	XP_018063373.1
hypothetical protein DSL72_003306	*Monilinia vaccinii-corymbosi*	0	90%	45.46%	QSZ28801.1

*V. tricorpus_* VRPKS-I-2_contig_JPET01000003					
Gene name	Species of BLAST hit	E-value	% coverage	% identity	Accession number
Fusaridione A synthetase fsdS	*Colletotrichum fructicola*	0	90%	85.90%	KAF4886526.1
Fusaridione A synthetase	*Colletotrichum fructicola*	0	90%	85.88%	XP_031875985.1

ChaA	*Chaetomium olivaceum*	0	90%	54.35%	QJX57338.1
hypothetical protein UA08_04451	*Talaromyces atroroseus*	0	89%	54.59%	XP_020120442.1
polyketide synthase	*Saccharata proteae CBS 121410*	0	89%	49.03%	KAF2084690.1
polyketide synthetase	*Mollisia scopiformis*	0	89%	47.73%	XP_018074800.1
XenE	*Xenoacremonium sp. BF-2018a*	0	89%	46.80%	QOJ72663.1
hypothetical protein PENSTE_c011G08446	*Penicillium steckii*	0	89%	46.94%	OQE21884.1
polyketide synthetase	*Xylaria acuta*	0	89%	46.56%	KAI0452050.1
fusaridione A synthetase fsdS	*Colletotrichum spaethianum*	0	89%	45.75%	GJC90835.1

*V. tricorpus_* VRPKS-I-6_contig_JPET01000003					
Gene name	Species of BLAST hit	E-value	% coverage	% identity	Accession number
hypothetical protein BDV25DRAFT_139560	*Aspergillus avenaceus*	0	99%	66.86%	KAE8150730.1
hypothetical protein ETB97_012280	*Aspergillus burnettii*	0	99%	66.24%	KAF5861957.1
uncharacterized protein BDW43DRAFT_306247	*Aspergillus alliaceus*	0	99%	66.27%	XP_031905223.1
hypothetical protein BDV23DRAFT_181744	*Aspergillus alliaceus*	0	99%	66.31%	KAE8392342.1
hypothetical protein DL764_009059	*Monosporascus ibericus*	0	99%	61.80%	RYO86086.1
hypothetical protein DL769_003084	*Monosporascus sp. CRB-8-3*	0	99%	61.31%	RYP78994.1
hypothetical protein DL765_005547	*Monosporascus sp. GIB2*	0	99%	61.50%	RYP15707.1
hypothetical protein BCR34DRAFT_599754	*Clohesyomyces aquaticus*	0	91%	65.92%	ORY13581.1
hypothetical protein GGR56DRAFT_680577	*Xylariaceae sp. FL0804*	0	99%	61.16%	KAI0484820.1
hypothetical protein CC78DRAFT_565861	*Didymosphaeria enalia*	0	91%	65.77%	KAF2268091.1

*V.tricorpus_* VRPKS-I-5_contig_JPET01000007					
Gene name	Species of BLAST hit	E-value	% coverage	% identity	Accession number
hypothetical protein F4775DRAFT_593279	*Biscogniauxia sp. FL1348*	0	96%	65.60%	KAI0597589.1
hypothetical protein F5X96DRAFT_674085	*Biscogniauxia mediterranea*	0	96%	65.14%	KAI1485991.1
hypothetical protein F4809DRAFT_640890	*Biscogniauxia mediterranea*	0	96%	65.29%	KAI1637132.1
hypothetical protein B0I37DRAFT_446777	*Chaetomium sp. MPI-CAGE-AT-0009*	0	96%	64.22%	KAH6844804.1
hypothetical protein F5144DRAFT_655621	*Chaetomium globosum*	0	96%	64.06%	KAH6628919.1
hypothetical protein BTUL_0001g00530	*Botrytis tulipae*	0	96%	61.55%	TGO20046.1
hypothetical protein BOTCAL_0139g00080	*Botryotinia calthae*	0	96%	61.02%	TEY65541.1

hypothetical protein BGAL_0408g00010	*Botrytis galanthina*	0	96%	60.90%	THV46179.1
hypothetical protein B7494_g1325	*Chlorociboria aeruginascens*	0	96%	48.64%	TAQ90374.1
hypothetical protein CJF32_00005378	*Rutstroemia sp. NJR-2017a WRK4*	0	96%	43.81%	PQE13487.1

*V. zaregamsianum_* VRPKS-I-17_NMXM01000005					
Gene name	Species of blast hit	% coverage	% identity	E value	Accession number
Lovastatin diketide synthase LovF 16	*Colletotrichum chlorophyti*	99%	72.06%	0	OLN86478.1
polyketide synthase	*Colletotrichum truncatum*	99%	71.59%	0	XP_036585096.1
Reducing polyketide synthase PKS2	*Colletotrichum trifolii*	99%	71.63%	0	TDZ28230.1
Reducing polyketide synthase PKS2	*Colletotrichum orbiculare MAFF 240422*	99%	71.58%	0	TDZ25780.1
Reducing polyketide synthase PKS2	*Colletotrichum sidae*	99%	71.54%	0	TEA18683.1
Reducing polyketide synthase PKS2	*Colletotrichum spinosum*	99%	71.50%	0	TDZ31026.1
polyketide synthase	*Colletotrichum musicola*	99%	71.69%	0	KAF6830768.1
polyketide synthase	*Colletotrichum sojae*	99%	71.48%	0	KAF6806435.1
beta-ketoacyl synthase domain-containing protein	*Colletotrichum nymphaeae SA-01*	99%	71.34%	0	KXH49587.1
beta-ketoacyl synthase domain-containing protein	*Colletotrichum simmondsii*	99%	71.27%	0	KXH44475.1

*V. zaregamsianum_* VRPKS-I-23_NMXM01000005					
Gene name	Species of blast hit	% coverage	% identity	E value	Accession number
phenolpthiocerol synthesis polyketide synthase ppsA	*Sodiomyces alkalinus F11*	99%	51.93%	0	XP_028462492.1
phenolpthiocerol synthesis polyketide synthase ppsA	*Plectosphaerella plurivora*	99%	45.93%	0	KAH6697431.1
phenolpthiocerol synthesis polyketide synthase ppsA	*Plectosphaerella cucumerina*	99%	44.91%	0	KAH7358949.1
Lovastatin diketide synthase LovF 13	*Colletotrichum chlorophyti*	99%	44.03%	0	OLN85715.1
beta-ketoacyl synthase domain-containing protein	*Colletotrichum incanum*	99%	44.29%	0	OHW97630.1
beta-ketoacyl synthase domain-containing protein	*Colletotrichum karsti*	98%	44.46%	0	XP_038741012.1
Reducing polyketide synthase FUB1	*Colletotrichum orbiculare MAFF 240422*	98%	44.52%	0	TDZ22788.1
beta-ketoacyl synthase domain-containing protein	*Colletotrichum graminicola M1.001*	98%	44.89%	0	XP_008090783.1

Reducing polyketide synthase FUB1	*Colletotrichum trifolii*	98%	44.32%	0	TDZ29218.1
polyketide synthase (beta-ketoacyl synthase domain-containing protein)	*Colletotrichum tofieldiae*	98%	44.47%	0	KZL67886.1

*V. zaregamsianum_* VRPKS-I-16_NMXM01000004					
Gene name	Species of blast hit	% coverage	% identity	E value	Accession number
putative polyketide synthase	*Plectosphaerella cucumerina*	99%	72.07%	0	KAH7369281.1
putative polyketide synthase	*Plectosphaerella plurivora*	99%	71.59%	0	KAH6673997.1
hypothetical protein K402DRAFT_461387	*Aulographum hederae CBS 113979*	99%	50.23%	0	KAF1988946.1
hypothetical protein L207DRAFT_422368	*Hyaloscypha variabilis F*	99%	48.85%	0	PMD43053.1
hypothetical protein DL98DRAFT_477463	*Cadophora sp. DSE1049*	99%	48.15%	0	PVH88801.1
uncharacterized protein K444DRAFT_592521	*Hyaloscypha bicolor E*	99%	47.94%	0	XP_024734639.1
hypothetical protein IFR05_000103	*Cadophora sp. M221*	99%	47.58%	0	KAG4444512.1
putative polyketide synthase	*Tothia fuscella*	99%	48.01%	0	KAF2419578.1
hypothetical protein NA56DRAFT_673334	*Hyaloscypha hepaticicola*	99%	46.19%	0	PMD16306.1
putative polyketide synthase	*Phaeosphaeria sp. MPI-PUGE-AT-0046c*	98%	45.29%	0	KAH7402395.1
hypothetical protein CNMCM7691_001823	*Aspergillus felis*	99%	45.76%	0	KAF7182343.1

*V. zaregamsianum_* VRPKS-I-25_NMXM01000004					
Gene name	Species of blast hit	% coverage	% identity	E value	Accession number
hypothetical protein S40293_03578	*Stachybotrys chartarum IBT 40293*	99%	91.30%	0	KFA46917.1
hypothetical protein S40288_08039	*Stachybotrys chartarum IBT 40288*	99%	89.17%	0	KFA72420.1
hypothetical protein S7711_03851	*Stachybotrys chartarum IBT 7711*	86%	90.99%	0	KEY68922.1
hypothetical protein DL770_006186	*Monosporascus sp. CRB-9-2*	99%	57.05%	0	RYP80513.1
hypothetical protein B7494_g6164	*Chlorociboria aeruginascens*	99%	56.56%	0	TAQ85507.1
hypothetical protein DL764_002272	*Monosporascus ibericus*	99%	56.64%	0	RYP07817.1
type I iterative PKS	*Aspergillus luchuensis*	99%	55.86%	0	XP_041547082.1
polyketide synthase	*Aspergillus neoniger CBS 115656*	99%	55.72%	0	XP_025484624.1

polyketide synthase	*Aspergillus costaricaensis CBS 115574*	99%	55.59%	0	XP_025542753.1
polyketide synthase	*Aspergillus niger*	99%	55.55%	0	GAQ46573.1

*V. zaregamsianum_* VRPKS-I-18_contig_NMXM01000001					
Gene name	Species of blast hit	% coverage	% identity	E value	Accession number
Reducing polyketide synthase PKS2	*Fulvia fulva*	79%	77.41%	0	UJO18003.1
Polyketide synthase	*Tolypocladium paradoxum*	78%	68.05%	0	POR36838.1
Polyketide synthase	*Tolypocladium capitatum*	78%	68.22%	0	PNY26863.1
polyketide synthase	*Thelonectria olida*	78%	65.50%	0	KAH6887502.1
hypothetical protein FDECE_8493	*Fusarium decemcellulare*	78%	64.87%	0	KAF5005076.1
Lovastatin diketide synthase mokB	*Trichoderma lentiforme*	78%	64.94%	0	KAF3065508.1
fatty acid synthase S-acetyltransferase	*Trichoderma harzianum*	78%	64.98%	0	KKO98752.1
hypothetical protein THARTR1_06994	*Trichoderma harzianum*	78%	65.12%	0	PNP52390.1
hypothetical protein CONLIGDRAFT_690934	*Coniochaeta ligniaria NRRL 30616*	78%	64.48%	0	OIW25172.1
hypothetical protein VHEMI00830	*Torrubiella hemipterigena*	78%	62.90%	0	CEJ80659.1

*V. zaregamsianum*_VRPKS-I-6_contig_NMXM01000001					
Gene name	Species of blast hit	% coverage	% identity	E value	Accession number
hypothetical protein BDV25DRAFT_139560	*Aspergillus avenaceus*	99%	67.20%	0	KAE8150730.1
hypothetical protein ETB97_012280	*Aspergillus burnettii*	99%	66.88%	0	KAF5861957.1
hypothetical protein BDV23DRAFT_181744	*Aspergillus alliaceus*	99%	66.76%	0	KAE8392342.1
uncharacterized protein BDW43DRAFT_306247	*Aspergillus alliaceus*	99%	66.74%	0	XP_031905223.1
hypothetical protein DL764_009059	*Monosporascus ibericus*	99%	61.94%	0	RYO86086.1
hypothetical protein DL769_003084	*Monosporascus sp. CRB-8-3*	99%	61.35%	0	RYP78994.1
hypothetical protein DL765_005547	*Monosporascus sp. GIB2*	99%	61.53%	0	RYP15707.1
hypothetical protein BCR34DRAFT_599754	*Clohesyomyces aquaticus*	91%	66.30%	0	ORY13581.1
hypothetical protein GGR56DRAFT_680577	*Xylariaceae sp. FL0804*	99%	61.37%	0	KAI0484820.1
hypothetical protein CC78DRAFT_565861	*Didymosphaeria enalia*	91%	66.23%	0	KAF2268091.1

*V. zaregamsianum_* VRPKS-I-7_contig_NMXM01000002					

Gene name	Species of blast hit	% coverage	% identity	E value	Accession number
polyketide synthase dehydratase domain-containing protein	*Sarocladium implicatum*	96%	78.19%	0	KAH8169122.1
hypothetical protein BDV25DRAFT_168001	*Aspergillus avenaceus*	96%	58.44%	0	KAE8154018.1
hypothetical protein F5B20DRAFT_519373	*Whalleya microplaca*	96%	57.63%	0	KAI1085697.1
uncharacterized protein ASPWEDRAFT_42595	*Aspergillus wentii DTO 134E9*	96%	58.29%	0	XP_040688298.1
putative polyketide synthase	*Hypoxylon sp. FL1284*	96%	56.15%	0	KAI0169670.1
hypothetical protein ACN38_g5709	*Penicillium nordicum*	96%	56.06%	0	KOS43405.1
Beta-ketoacyl synthase	*Penicillium camemberti*	96%	56.15%	0	CRL31206.1
hypothetical protein HAV15_003755	*Penicillium sp. str. #12*	96%	56.03%	0	KAF4765052.1
hypothetical protein ETB97_004590	*Aspergillus burnettii*	96%	56.11%	0	KAF5858310.1
hypothetical protein BDV23DRAFT_185642	*Aspergillus alliaceus*	96%	55.88%	0	KAE8388153.1

*V. zaregamsianum_* VRPKS-I-26_contig_NMXM01000003					
Gene name	Species of blast hit	% coverage	% identity	E value	Accession number
hypothetical protein F5B20DRAFT_586947	*Whalleya microplaca*	99%	52.38%	0	KAI1073750.1
hypothetical protein DL768_003915	*Monosporascus sp. mg162*	99%	51.63%	0	RYP50633.1
RecName: Full=Highly reducing polyketide synthase apmlA; Short=HRPKS apmlA; AltName: Full=Phaeospelide A biosynthesis cluster protein apmlA	*Apiospora sphaerosperma*	99%	50.46%	0	P0CU84.1
hypothetical protein ACN38_g10438	*Penicillium nordicum*	98%	43.83%	0	KOS38742.1
hypothetical protein ACN42_g5244	*Penicillium freii*	98%	43.04%	0	KUM61888.1
hypothetical protein PENPOL_c017G09878	*Penicillium polonicum*	98%	43.27%	0	OQD61348.1
reducing type I polyketide synthase	*Xylariomycetidae sp. FL2044*	99%	42.58%	0	KAH9902283.1
reducing type I polyketide synthase	*Annulohypoxylon maeteangense*	98%	42.68%	0	KAI0883761.1
Fumagillin dodecapentaenoate synthase	*Lachnellula willkommii*	99%	42.62%	0	TVY89575.1
Fumagillin dodecapentaenoate synthase	*Lachnellula arida*	99%	42.58%	0	TVY19100.1

*V. zaregamsianum_* VNRPKS-I-2_contig_NMXM01000003					
Gene name	Species of blast hit	% coverage	% identity	E value	Accession number
hypothetical protein DL764_006210	*Monosporascus ibericus*	100%	84.68%	0	RYP01440.1
uncharacterized protein BGZ61DRAFT_593032	*Ilyonectria robusta*	99%	79.46%	0	XP_046097594.1

PKS16 protein	*Ilyonectria destructans*	98%	79.36%	0	KAH7000898.1
hypothetical protein BKA56DRAFT_558919	*Ilyonectria sp. MPI-CAGE-AT-0026*	99%	79.01%	0	KAH6970569.1
PKS16 protein	*Dactylonectria estremocensis*	99%	79.36%	0	KAH7155810.1
PKS16 protein	*Dactylonectria macrodidyma*	98%	77.81%	0	KAH7120608.1
PKS16 protein	*Corynespora cassiicola Philippines*	99%	59.38%	0	PSN68069.1
uncharacterized protein INS49_005730	*Diaporthe citri*	85%	59.40%	0	XP_043027652.1
PKS16 protein	*Rhexocercosporidium sp. MPI-PUGE-AT-0058*	99%	52.10%	0	KAH7357101.1
polyketide synthase	*Leptodontidium sp. 2 PMI_412*	99%	52.31%	0	KAH9225058.1

*V. zaregamsianum_* VRPKS-I-14_contig_NMXM01000009					
Gene name	Species of blast hit	% coverage	% identity	E value	Accession number
putative polyketide synthase	*Truncatella angustata*	100%	56.98%	0	XP_045957259.1
hypothetical protein TruAng_007522	*Truncatella angustata*	100%	56.47%	0	KAH8198320.1
Nonribosomal peptide synthetase-like protein	*Acremonium chrysogenum ATCC 11550*	99%	53.95%	0	KFH40504.1
hypothetical protein AJ80_00462	*Polytolypa hystricis UAMH7299*	100%	54.29%	0	PGH27912.1
uncharacterized protein B0I36DRAFT_397789	*Microdochium trichocladiopsis*	97%	48.37%	0	XP_046005243.1
Polyketide synthase	*Penicillium sp. RFL-2021a*	99%	46.52%	0	KAF7713846.1
hypothetical protein PDE_03926	*Penicillium oxalicum 114-2*	99%	46.08%	0	EPS28980.1
Compactin diketide synthase mokB	*Penicillium rolfsii*	99%	46.40%	0	KAF3388919.1
Lovastatin diketide synthase LovF	*Penicillium subrubescens*	99%	46.34%	0	OKP11696.1
hypothetical protein PMG11_09333	*Penicillium brasilianum*	99%	46.26%	0	CEJ60771.1

*V. zaregamsianum_* VRPKS-I-21_contig_NMXM01000006					
Gene name	Species of blast hit	% coverage	% identity	E value	Accession number
Lovastatin diketide synthase LovF 2	*Colletotrichum chlorophyti*	100%	74.50%	0	OLN91972.1

polyketide synthase	*Colletotrichum plurivorum*	100%	74.12%	0	KAF6826616.1
polyketide synthase	*Colletotrichum musicola*	100%	74.08%	0	KAF6845034.1
beta-ketoacyl synthase domain-containing protein	*Colletotrichum fioriniae PJ7*	100%	73.52%	0	EXF80608.1
polyketide synthase (beta-ketoacyl synthase domain-containing protein)	*Colletotrichum tofieldiae*	100%	73.65%	0	KZL70949.1
beta-ketoacyl synthase domain-containing protein	*Colletotrichum scovillei*	100%	73.90%	0	KAG7053867.1
polyketide synthase	*Colletotrichum sojae*	100%	74.16%	0	KAF6809610.1
beta-ketoacyl synthase domain-containing protein	*Colletotrichum simmondsii*	100%	73.77%	0	KXH36687.1
beta-ketoacyl synthase domain-containing protein	*Colletotrichum scovillei*	100%	73.29%	0	XP_035330138.1
beta-ketoacyl synthase domain-containing protein	*Colletotrichum nymphaeae* SA-01	100%	73.94%	0	KXH46086.1

*V. zaregamsianum_* VRPKS-I-19_contig_NMXM01000013					
Gene name	Species of blast hit	% coverage	% identity	E value	Accession number
hypothetical protein J1614_005792	*Leptosphaeria biglobosa*	99%	75.93%	0	KAH9873394.1
polyketide synthase	*Metarhizium rileyi RCEF 4871*	99%	62.67%	0	OAA35905.1
Type I Iterative PKS	*Claviceps pusilla*	99%	63.13%	0	KAG6001262.1
Type I Iterative PKS	*Claviceps purpurea*	99%	62.95%	0	KAG6313294.1
Type I Iterative PKS	*Claviceps purpurea*	98%	62.74%	0	KAG6145386.1
Type I Iterative PKS	*Claviceps purpurea*	99%	62.90%	0	KAG6319079.1
Type I Iterative PKS	*Claviceps aff. purpurea*	99%	62.95%	0	KAG6303654.1
Type I Iterative PKS	*Claviceps sp. LM454 group G7*	99%	62.93%	0	KAG6106452.1
Type I Iterative PKS	*Claviceps purpurea*	99%	62.86%	0	KAG6144460.1
Type I Iterative PKS	*Claviceps purpurea*	99%	62.86%	0	KAG6278039.1

*V. zaregamsianum_* VNRPKS-I-1_contig_NMXM01000005					
Gene name	Species of blast hit	% coverage	% identity	E value	Accession number
hypothetical protein CcaCcLH18_10320	*Colletotrichum camelliae*	100%	79.05%	0	KAH0426510.1
uncharacterized protein CkaCkLH20_00019	*Colletotrichum karsti*	100%	79.37%	0	XP_038751444.1
hypothetical protein CGLO_05047	*Colletotrichum gloeosporioides Cg-14*	100%	79.23%	0	EQB55056.1
hypothetical protein GQ607_010262	*Colletotrichum asianum*	100%	79.19%	0	KAF0322599.1

Conidial yellow pigment biosynthesis polyketide synthase	*Colletotrichum gloeosporioides*	100%	79.19%	0	XP_045268234.1
hypothetical protein K456DRAFT_1724748	*Colletotrichum gloeosporioides 23*	100%	79.19%	0	KAH9233890.1
Conidial yellow pigment biosynthesis polyketide synthase	*Colletotrichum fructicola*	100%	79.19%	0	KAF4937522.1
Conidial yellow pigment biosynthesis polyketide synthase	*Colletotrichum fructicola*	100%	79.14%	0	XP_031880204.1
Conidial yellow pigment biosynthesis polyketide synthase	*Colletotrichum siamense*	100%	79.14%	0	KAF4840476.1
Conidial yellow pigment biosynthesis polyketide synthase	*Colletotrichum fructicola*	100%	79.14%	0	KAF4912855.1

*V. zaregamsianum_* VRPKS-I-5_contig_NMXM01000008					
Gene name	Species of blast hit	% coverage	% identity	E value	Accession number
hypothetical protein F4775DRAFT_593279	*Biscogniauxia sp. FL1348*	99%	65.91%	0	KAI0597589.1
hypothetical protein F5144DRAFT_655621	*Chaetomium globosum*	99%	64.38%	0	KAH6628919.1
hypothetical protein B7494_g1325	*Chlorociboria aeruginascens*	99%	48.92%	0	TAQ90374.1
hypothetical protein BTUL_0001g00530	*Botrytis tulipae*	98%	63.97%	0	TGO20046.1
hypothetical protein BOTCAL_0139g00080	*Botryotinia calthae*	98%	63.26%	0	TEY65541.1
hypothetical protein BGAL_0408g00010	*Botrytis galanthina*	98%	62.99%	0	THV46179.1
Acyl transferase acyl hydrolase lysophospholipase protein	*Rutstroemia sp. NJR-2017a BVV2*	100%	43.84%	0	PQE27755.1
hypothetical protein CJF32_00005378	*Rutstroemia sp. NJR-2017a WRK4*	100%	44.00%	0	PQE13487.1
Acyl transferase/acyl hydrolase/lysophospholipase	*Penicillium italicum*	99%	42.86%	0	KGO65245.1
hypothetical protein F5Y04DRAFT_286173	*Hypomontagnella monticulosa*	99%	42.62%	0	KAI0376194.1

*V. zaregamsianum_* VRPKS-I-1_contig_NMXM01000001					
Gene name	Species of blast hit	% coverage	% identity	E value	Accession number
putative hybrid NRPS/PKS enzyme	*Didymosphaeria enalia*	99%	44.80%	0	KAF2268646.1
related to polyketide synthase	*Phialocephala subalpina*	99%	44.15%	0	CZR58354.1
hypothetical protein V492_07950	*Pseudogymnoascus sp. VKM F-4246*	99%	43.67%	0	KFY06571.1
Acetyl-CoA synthetase-like protein	*Glarea lozoyensis ATCC 20868*	99%	43.59%	0	XP_008078954.1
polyketide synthase	*Halenospora varia*	99%	43.55%	0	KAH6673321.1
polyketide synthase	*Saccharata proteae CBS 121410*	99%	43.33%	0	KAF2084690.1

amino acid adenylation domain-containing protein	*Hymenoscyphus varicosporioides*	95%	44.64%	0	KAH8646967.1
hypothetical protein UREG_03815	*Uncinocarpus reesii 1704*	99%	42.07%	0	XP_002544298.1
uncharacterized protein BP5553_06488	*Venustampulla echinocandica*	99%	42.97%	0	XP_031868532.1
hypothetical protein IFR04_013564	*Cadophora malorum*	99%	41.74%	0	KAG4413304.1

*V. zaregamsianum_* VRPKS-I-6_contig_NMXM01000001					
Gene name	Species of blast hit	% coverage	% identity	E value	Accession number
polyketide synthase	*Fusarium avenaceum*	99%	55.53%	0	KIL85244.1
hypothetical protein BKA59DRAFT_387557	*Fusarium tricinctum*	99%	55.28%	0	KAH7261591.1
hypothetical protein DER45DRAFT_513901	*Fusarium avenaceum*	99%	55.22%	0	KAH6968136.1
putative polyketide synthase	*Fusarium redolens*	99%	55.26%	0	ALQ32917.1
unnamed protein product	*Fusarium equiseti*	99%	55.07%	0	CAG7561821.1
unnamed protein product	*Fusarium graminearum*	99%	54.73%	0	VTO93732.1
polyketide synthase	*Fusarium beomiforme*	99%	55.14%	0	KAF4341752.1
unnamed protein product	*Fusarium graminearum*	99%	54.76%	0	CZS84488.1
unnamed protein product	*Fusarium graminearum*	99%	54.73%	0	CAF3461387.1
unnamed protein product	*Fusarium graminearum*	99%	54.73%	0	CAG2002135.1

*V. zaregamsianum*_PKS-III_contig_NMXM01000003					
Gene name	Species of blast hit	% coverage	% identity	E value	Accession number
putative chalcone and stilbene synthase domain-containing protein	*Colletotrichum sublineola*	100%	75.06%	0	KDN65911.1
chalcone and stilbene synthase domain-containing protein	*Colletotrichum tofieldiae*	100%	75.45%	0	GKT65818.1
chalcone synthase	*Colletotrichum spaethianum*	100%	75.23%	0	GJC81220.1
chalcone and stilbene synthase domain-containing protein	*Plectosphaerella cucumerina*	100%	76.46%	0	KAH7375301.1
chalcone and stilbene synthase domain-containing protein	*Colletotrichum tofieldiae*	100%	75.23%	0	KZL74511.1
chalcone and stilbene synthase domain-containing protein	*Colletotrichum incanum*	100%	75.23%	0	KZL80557.1
chalcone and stilbene synthase domain-containing protein	*Colletotrichum truncatum*	100%	75.28%	0	XP_036586176.1
putative chalcone and stilbene synthase domain-containing protein	*Colletotrichum incanum*	100%	75.00%	0	OHW96035.1
chalcone synthase	*Colletotrichum liriopes*	100%	74.77%	0	GKT49639.1
chalcone and stilbene synthase domain-containing protein	*Colletotrichum graminicola M1.001*	100%	74.61%	0	XP_008099452.1
